# Direct and indirect responses of the Arabidopsis transcriptome to an induced increase in trehalose 6-phosphate

**DOI:** 10.1093/plphys/kiae196

**Published:** 2024-04-09

**Authors:** Omri Avidan, Marina C M Martins, Regina Feil, Marc Lohse, Federico M Giorgi, Armin Schlereth, John E Lunn, Mark Stitt

**Affiliations:** Max Planck Institute of Molecular Plant Physiology, Am Muehlenberg 1, 14476 Potsdam-Golm, Germany; Max Planck Institute of Molecular Plant Physiology, Am Muehlenberg 1, 14476 Potsdam-Golm, Germany; Max Planck Institute of Molecular Plant Physiology, Am Muehlenberg 1, 14476 Potsdam-Golm, Germany; Max Planck Institute of Molecular Plant Physiology, Am Muehlenberg 1, 14476 Potsdam-Golm, Germany; Max Planck Institute of Molecular Plant Physiology, Am Muehlenberg 1, 14476 Potsdam-Golm, Germany; Max Planck Institute of Molecular Plant Physiology, Am Muehlenberg 1, 14476 Potsdam-Golm, Germany; Max Planck Institute of Molecular Plant Physiology, Am Muehlenberg 1, 14476 Potsdam-Golm, Germany; Max Planck Institute of Molecular Plant Physiology, Am Muehlenberg 1, 14476 Potsdam-Golm, Germany

## Abstract

Trehalose 6-phosphate (Tre6P) is an essential signal metabolite that regulates the level of sucrose, linking growth and development to the metabolic status. We hypothesized that Tre6P plays a role in mediating the regulation of gene expression by sucrose. To test this, we performed transcriptomic profiling on Arabidopsis (*Arabidopsis thaliana*) plants that expressed a bacterial TREHALOSE 6-PHOSPHATE SYNTHASE (TPS) under the control of an ethanol-inducible promoter. Induction led to a 4-fold rise in Tre6P levels, a concomitant decrease in sucrose, significant changes (FDR ≤ 0.05) of over 13,000 transcripts, and 2-fold or larger changes of over 5,000 transcripts. Comparison with nine published responses to sugar availability allowed some of these changes to be linked to the rise in Tre6P, while others were probably due to lower sucrose or other indirect effects. Changes linked to Tre6P included repression of photosynthesis-related gene expression and induction of many growth-related processes including ribosome biogenesis. About 500 starvation-related genes are known to be induced by SUCROSE-NON-FERMENTING-1-RELATED KINASE 1 (SnRK1). They were largely repressed by Tre6P in a manner consistent with SnRK1 inhibition by Tre6P. SnRK1 also represses many genes that are involved in biosynthesis and growth. These responded to Tre6P in a more complex manner, pointing toward Tre6P interacting with other C-signaling pathways. Additionally, elevated Tre6P modified the expression of genes encoding regulatory subunits of the SnRK1 complex and TPS class II and FCS-LIKE ZINC FINGER proteins that are thought to modulate SnRK1 function and genes involved in circadian, TARGET OF RAPAMYCIN, light, abscisic acid, and other hormone signaling.

## Teaser

Analysis of the response of the transcriptome to induced elevation of trehalose 6-phosphate separates direct from indirect responses, identifies regulated processes, and assesses molecular mechanisms.

## Introduction

Trehalose 6-phosphate (Tre6P) is an essential signal metabolite in plants (Fichtner and Lunn 2021). Tre6P is synthesized by TREHALOSE-6-PHOSPHATE SYNTHASE (TPS) from UDP-glucose (UDPGlc) and glucose 6-phosphate (Glc6P) and dephosphorylated by TREHALOSE-6-PHOSPHATE PHOSPHATASE (TPP) to produce trehalose (Cabib and Leloir 1958). In addition to catalytically active TPS proteins like AtTPS1 in Arabidopsis (*Arabidopsis thaliana*), plants possess a family of catalytically inactive TPS proteins (class II TPS proteins) (Leyman et al. 2001; Lunn 2007; Ramon et al. 2009; Vandesteene et al. 2010; Lunn et al. 2014; Delorge et al. 2015) and a family of TPP proteins (Leyman et al. 2001; Vandesteene et al. 2012; Kretzschmar et al. 2015). The embryo-lethal phenotype of Arabidopsis *tps1* mutants (Eastmond et al 2002), the phenotype of Arabidopsis overexpressing bacterial TPS and TPP (Schluepmann et al. 2003) and information about Tre6P levels (Lunn et al. 2006) established that Tre6P acts as a signal and is essential for growth. Tre6P profoundly influences metabolism (Figueroa and Lunn 2016; Fichtner and Lunn 2021) and development including flowering (Wahl et al. 2013), inflorescence structure (Satoh-Nagasawa et al. 2006; Claeys et al. 2019, Klein et al. 2022), shoot branching (Fichtner et al. 2017, 2021), and lateral root formation (Morales-Herrera et al. 2023). Modification of Tre6P levels can influence crop yield (Nuccio et al. 2015; Griffiths et al. 2016; Paul et al. 2020). However, questions remain concerning how Tre6P acts. In the following, we investigate the short-term response of the transcriptome to an induced increase in Tre6P.

It has been proposed that Tre6P is a signal for sucrose availability (Lunn et al. 2006). Tre6P levels correlate strongly with sucrose in Arabidopsis during recurring and perturbed diel cycles (Lunn et al. 2006; Carillo et al. 2013; Martins et al. 2013; Sulpice et al. 2014; Figueroa et al. 2016; Annunziata et al. 2017), after sugar feeding (Lunn et al. 2006; Nunes et al. 2013; Yadav et al. 2014) and in response to genetic interventions (Lunn et al. 2006; dos Anjos et al. 2018). Sucrose and Tre6P also correlate in other species (Debast et al. 2011; Martínez-Barajas et al. 2011; Henry et al. 2014; Zhang et al. 2015). The response to sucrose appears to be specific; responses to manipulation of other sugars or nitrogen (N) are explained by concomitant changes in sucrose (Yadav et al. 2014). The mechanism linking Tre6P to sucrose is unknown, but depends on de novo protein synthesis (Yadav et al. 2014) and features of AtTPS1 protein (Fichtner et al. 2021).

Genetic interventions that alter TPS or TPP expression result in reciprocal changes of Tre6P and sucrose. This occurs in response to constitutive overexpression (Gomez et al. 2010; Yadav et al. 2014; Nuccio et al. 2015), vascular tissue-specific overexpression (Fichtner et al. 2020, 2021), and induced expression (Martins et al. 2013; Yadav et al. 2014; Figueroa et al. 2016). This reciprocal response implies that Tre6P inhibits sucrose production and/or stimulates sucrose consumption. These observations led to proposal of the sucrose:Tre6P nexus hypothesis, according to which Tre6P has a dual function as a signal of and negative feedback regulator of sucrose levels (Lunn et al. 2014; Figueroa and Lunn 2016). The relationship between sucrose and Tre6P depends on the tissue, developmental stage, and environmental conditions (Fichtner and Lunn 2021).

The role of Tre6P may vary between source tissues that produce and export sucrose, and sink tissues that utilize sucrose. In source leaves in the light, induced increases in Tre6P post-translationally stimulate phospho*enol*pyruvate carboxylase and nitrate reductase, increase synthesis of organic acids and amino acids and decrease synthesis of sucrose (Figueroa et al. 2016). At night, increased Tre6P restricts starch mobilization (Martins et al. 2013; dos Anjos et al. 2018). *AtTPS1* is mainly expressed in the phloem parenchyma and the companion cell-sieve element complex (Fichtner et al. 2020). Presumably, Tre6P formed in the phloem parenchyma moves via plasmodesmata into mesophyll cells to regulate metabolism, whilst Tre6P produced in the companion cells provides a signal linked to sucrose movement in the phloem. Tre6P modulates long-distance signaling that controls developmental transitions which set up a future demand for sucrose, including flowering (Wahl et al. 2013) and shoot branching (Fichtner et al. 2017, 2021). In sink tissues, Tre6P regulates sucrose mobilization by inhibiting sucrose synthase (Fedosejevs et al. 2018) and modifying expression of sucrolytic enzymes and SWEETs (Bledsoe et al. 2017; Oszvald et al. 2018; Fichtner et al. 2021). Tre6P stimulates metabolism in axillary buds (Fichtner et al. 2017, 2021) and promotes storage product accumulation in Arabidopsis seeds by stabilizing WRINKLED1 (Zhai et al. 2018) and in pea seeds by inducing an auxin biosynthesis gene (Meitzel et al. 2021).

Tre6P can act by inhibiting SUCROSE-NON-FERMENTING-1-RELATED KINASE 1 (SnRK1) (Zhang et al. 2009; Paul et al. 2020). SnRK1 is the plant homolog of yeast SUCROSE-NON-FERMENTING1 (SNF1) and mammalian AMP-ACTIVATED PROTEIN KINASE (AMPK) that play a key role in low-energy signaling (Jossier et al. 2009; Hulsmans et al. 2016; Crepin and Rolland 2019; Baena-González and Lunn 2020). One line of evidence is that Tre6P, along with other sugar phosphates like Glc6P, inhibits in vitro SnRK1 activity (Zhang et al. 2009; Debast et al. 2011; Delatte et al. 2011; Nunes et al. 2013; Coello and Martínez-Barajas 2014; Emanuelle et al. 2015). Inhibition requires an unidentified protein and has only been observed in extracts from sink tissues (Zhang et al. 2009; Emmanuelle et al. 2015). Tre6P interferes with binding of SnRK1-activating kinases (SnAK1/GRIK1, SnAK2/GRIK2) to SnRK1 α subunit, leading to inhibition of SnRK1 activity (Glab et al. 2017; Zhai et al. 2018; Hwang et al. 2019). However, SnAK1 and SnAK2 are expressed ubiquitously, indicating that this is a separate mechanism to that reported by Zhang et al. (2009). The second line of evidence is that changes of Tre6P levels in vivo often associate negatively with the abundance of a set of C-starvation-induced transcripts (Zhang et al. 2009; Paul et al. 2010; Henry et al. 2015; Bledsoe et al. 2017: Peixoto et al. 2021) that are induced by transient SnRK1 overexpression in mesophyll protoplasts (Baena-Gonzalez et al. 2007). Many of these genes are repressed by constitutive overexpression of bacterial TPS (Zhang et al. 2009; Oszvald et al. 2018). A subset also responds after application of permeable Tre6P analogs (Griffiths et al. 2016; Morales-Herrera et al. 2023). Two recent findings provide further evidence for interactions between Tre6P and SnRK1. One is that TPS class II proteins physically interact with SnRK1 and can inhibit its activity (van Leene et al. 2022). The other is that whilst the positive correlation of Tre6P with sucrose was retained in Arabidopsis lines with altered SnRK1 expression, the response was damped as SnRK1 expression increased (Peixoto et al. 2021).

However, changes of SnRK1 downstream target transcripts are seen in plant material that is dominated by mature source leaves, whereas in vitro inhibition of SnRK1 by Tre6P is only observed in extracts from sink tissues (see Baena-Gonzalez and Lunn 2020, for discussion). Furthermore, diel changes in SnRK1 activity, based on phosphorylation of an in vivo reporter protein, did not always correspond with Tre6P levels and varied independently of the abundance of SnRK1 downstream target transcripts, indicating that the latter is not always a faithful readout of SnRK1 activity (Avidan et al. 2023). Thus, whilst it has been established that Tre6P can act, at least in part, via inhibition of SnRK1 activity, open questions remain concerning the molecular mechanism and whether this interaction plays a major role in Tre6P and SnRK1 signaling.

Except for a recent study that investigated the impact of an induced increase of Tre6P on expression of a small subset of SnRK1 targets (Peixoto et al. 2021), previous genetic studies of the impact of Tre6P on transcript abundance have used constitutive overexpression of TPS or TPP. Genetic interventions generate reciprocal changes in the levels of Tre6P and sucrose and other sugars, and major changes in metabolism, growth, and development. This makes it difficult to distinguish between direct responses to Tre6P, indirect responses due to changes in sucrose and other metabolites, and pleiotropic effects. In the following, we analyze the short-term response of transcript abundance to an induced increase in Tre6P. We identify, at a global level, genes whose expression is regulated by Tre6P, ask what biological functions are impacted and assess to what extent the response can be explained via inhibition of SnRK1.

## Results

### Response to elevated Tre6P over an entire light or dark period

In an initial experiment, two iTPS lines (29.2, 31.3) and control alcR plants were sprayed with ethanol or water at dawn and harvested 12 h later at the end of the day (ED) or sprayed at dusk and harvested 12 h later at the end of the night (EN), and profiled using Affymetrix ATH1 arrays (see Supplementary Text for details; Supplementary Table S1 lists all utilized transcriptome datasets). Differentially expressed genes (DEGs) were identified using a false discovery rate (FDR) < 0.05. We termed the response to induction the “iTPS response”. Whilst line 29.1 showed a stronger response than line 31, their responses were strongly correlated (*R*^2^ = 0.96 and 0.98 at ED and EN, respectively; Supplementary Fig. S1, Supplementary Table S2, Supplementary Data Set S1).

### Deconvolution of the iTPS response using the carbon response factor

Given that Tre6P is a sucrose signal, it might be expected that the iTPS response would qualitatively resemble the response to elevated sugar. We calculated a carbon response factor (CRF) for each gene, based on transcriptomics data from nine published experiments that focused on short-term responses and minimized side-effects due to circadian- or light-signaling (Supplementary Fig. S2, Supplementary Data Set S2). We assigned transcripts to three CRF groups: G_1_ contained transcripts that responded in iTPS in the same direction as to increased sugar, G_2_ contained transcripts that responded in the opposite direction, and G_0_ contained transcripts that responded in iTPS but did not respond to sugar. A relaxed filter (log_2_ CRF > 0.1) was used to maximize assignment to G_1_ or G_2_.

There was little similarity between the CRF and the overall iTPS response at ED and even less at EN (Supplementary Fig. S3, A and B). Transcripts assigned to G_1_ (48% and 24% of DEGs at ED and EN, respectively) showed a positive correlation between their CRF and iTPS response, consistent with them responding to elevated Tre6P. Transcripts assigned to G_2_ (23% and 57% at ED and EN) showed a negative correlation between their CRF and iTPS response, consistent with them responding to the decrease in sugars (Supplementary Fig. S3, C and D, Supplementary Table S2, Supplementary Data Set S2, and Supplementary text for details). Some transcripts (29% and 19% at ED and EN, respectively) were assigned to G_0_. Thus, the 12-h post-induction response includes many indirect effects at ED, and these predominate at EN. We decided to focus on induction in the light and to harvest at earlier times.

### Early response to induction of TPS at the beginning of the day

**Figure 1.**
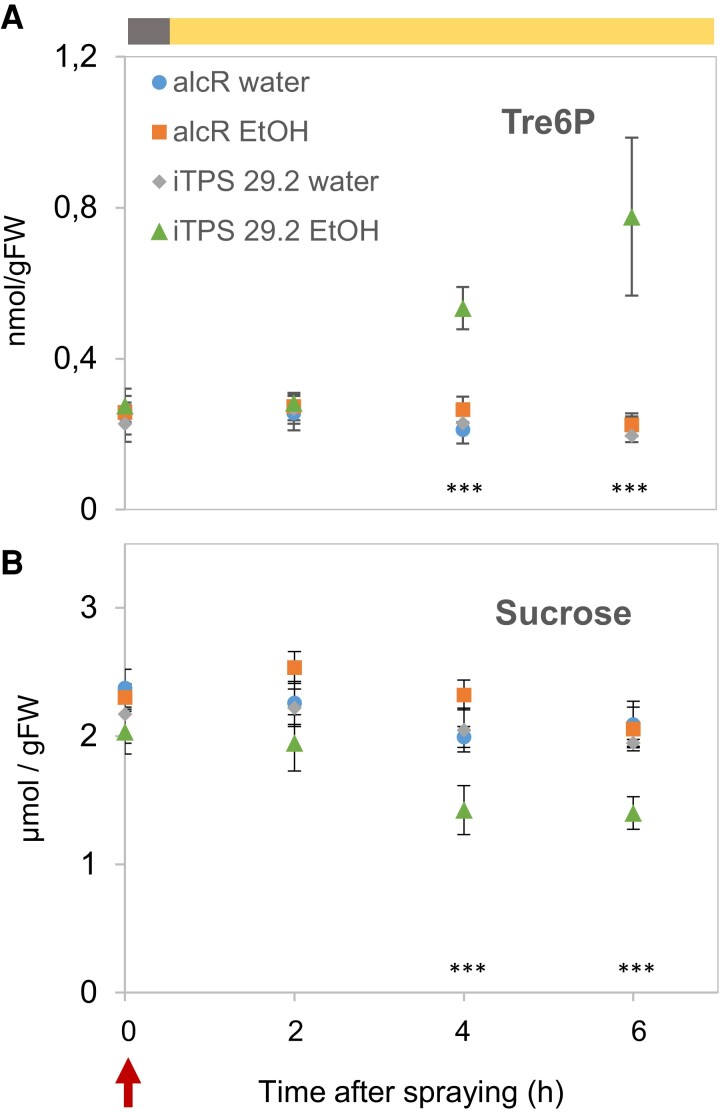
Changes of Tre6P and sucrose after induction of bacterial TPS in the light. Arabidopsis iTPS29.2 and alcR plants (for details see Materials and Methods and Supplementary text) were grown in long-day conditions (16 h light/8 h dark, 160 *µ*mol m^−2^ s^−1^ irradiance) for 22 d and were then sprayed at 0.5 h after dawn (Zeitgeber time 0.5, ZT0.5, red arrow) with either 2% (v/v) ethanol (EtOH) or water, and harvested 2, 4 and 6 h later (ZT2.5, 4.5, 6.5). The light period is indicated in the upper gray (dark) and orange (light) bar. Measurements were carried out on three controls (alcR and iTPS sprayed with water and alcR sprayed with 2% (v/v) ethanol) and on iTPS29.2 sprayed with 2% (v/v) ethanol to induce bacterial TPS. **A)** Tre6P, **B)** Sucrose, nmol or µmol per g fresh weight (FW). The results are plotted as mean ± Sd. (*n* = 4) (each replicate contained four to five whole rosettes). Significant differences (one-way ANOVA, Holm-Sidak) are denoted by asterisks (*P < 0.05, **P < 0.01, ***P < 0.001) when the ethanol-induced iTPS samples were significantly different from all three controls in pairwise comparisons. Data for additional metabolites are shown in Supplementary Fig. S4 and the original data are provided in Supplementary Data Set S3.

Twenty-two-day-old iTPS29.2 and alcR plants were sprayed with ethanol or water 0.5 h after dawn and rosettes harvested 2, 4 and 6 h later. Bacterial TPS protein was detectable by 2 h and abundance rose further at 4 and 6 h (Supplementary Fig. S4A). Tre6P levels were unaltered at 2 h, increased significantly at 4 h, and rose further at 6 h to 4-fold higher levels than in controls (Fig. 1A; Supplementary Data Set S3). Sucrose levels decreased significantly to 70% of those in controls by 4 h (Fig. 1B). Tre6P and sucrose levels were positively correlated in controls but negatively correlated from 2 h onwards in ethanol-sprayed line 29.2 (Supplementary Fig. S4B). The increase in Tre6P was accompanied by a decrease of Glc6P, Fru6P, and PEP, and an increase of pyruvate, malate, fumarate, citrate, aconitate, 2-oxoglutarate, and shikimate (Supplementary Fig. S4C). This resembled previous studies (Figueroa et al. 2016; Avidan et al. 2023), which also documented a widespread increase of amino acids.

**Figure 2.**
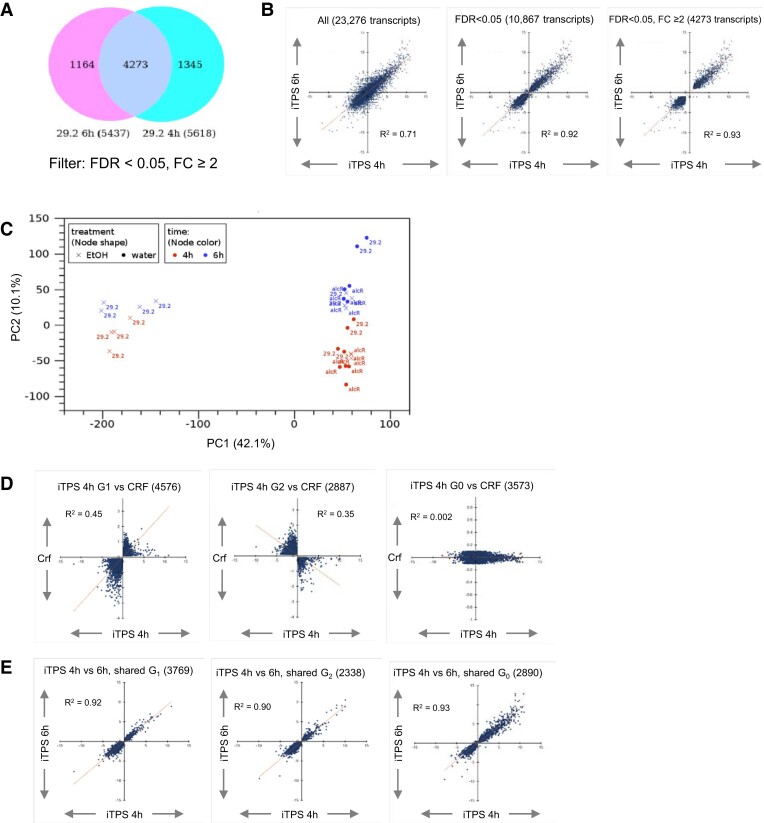
Changes of transcript abundance 4 and 6 h after induction of bacterial TPS in the light. Arabidopsis iTPS29.2 and alcR plants (for details see Materials and Methods, Supplementary text) were grown and treated with ethanol (EtOH) or water as described in Fig. 1 and RNA was extracted for RNAseq analysis. **A)** DEGs were identified by comparing the transcript abundance in the ethanol-sprayed samples to their water-sprayed control, using RPKM values (see Materials and Methods). The VENN diagram compares DEGs that passed FDR < 0.05 and FC ≥ 2 filters (FDR, false discovery rate; FC, fold change) in the 4 and 6 h datasets; the numbers at the bottom represent total DEGs, while numbers located within circles represent shared and nonshared responses. **B)** Comparison of transcript responses at 4 and 6 h for all 23.8K detected genes (left), the 10,867 transcripts that passed the FDR < 0.05 filter (middle), and the 4,273 transcripts that passed the FDR < 0.05 and FC ≥ 2 filter (right). **C)** PC analysis performed on all detected transcripts. Genotype (29.2, alcR) is indicated in the figure, red and blue are the 4 and 6 h treatments, circles and crosses are water- and ethanol-sprayed (see insert). **D)** Deconvoluted response to elevated Tre6P plotted against the CRF for iTPS 4 h (analogous plots for the response at 6 h are provided in Supplementary Fig. S5B). The CRF summarizes the response of a given Arabidopsis gene transcript to a change in sugar levels across a set of treatments. They included addition of exogenous glucose or sucrose to starved seedlings in liquid culture under continuous low light (Bläsing et al. 2005, Osuna et al. 2007), comparison of the starchless *pgm* mutant with wild-type plants at four times in the diel cycle (Gibon et al. 2004; Bläsing et al. 2005; Usadel et al. 2008), and illumination of wild-type plants for 4 h with ambient or low CO_2_ (Bläsing et al. 2005). An increasingly positive sign denotes an increasingly large average increase in abundance, an increasingly negative sign denotes an increasingly large average decrease in abundance and a value around zero indicates that average transcript abundance does not respond to sugar status. Group 1 (G_1_) denotes transcripts where the iTPS response and CRF are qualitatively the same and, by inference, the iTPS response may be a direct response to elevated Tre6P. Group 2 (G_2_) denotes transcripts where the iTPS response and CRF are qualitatively opposed and by inference the iTPS response is unlikely to be a direct response to elevated Tre6P. Group 0 (G_0_) denotes transcripts that respond in the iTPS response but cannot be assigned to G1 or G2 because they do not show a consistent response to changes in sugars (for details see Supplementary Fig. S2 and Supplementary Data Set S2). **E)** Comparability of the response of transcript assigned to G_1_, G_2_ and G_0_ in the 4 and 6 h data set.

RNA sequencing (RNAseq) was performed on quadruplicate samples harvested 4 and 6 h after spraying. Ethanol- and water-sprayed quadruplicates were used to calculate the FDR (Benjamini and Hochberg 1995) and average fold change (FC) in abundance. Of 23.8K detected transcripts, >13K passed an FDR < 0.05 filter at both time points (>55% of detected transcripts) and 5.6 and 5.4K passed a combined filter (FDR < 0.05, FC ≥ 2) at 4 and 6 h (∼23% of detected transcripts) (Fig. 2A; Supplementary Data Set S4). The 4 and 6 h responses were highly correlated when compared for all detected transcripts, transcripts that passed the FDR-only filter, or transcripts that passed the combined filter (*R*^2^ = 0.71, 0.92, and 0.93, respectively; Fig. 2B, Supplementary Table S2).

To eliminate a possible effect of ethanol and off-target effects of alcR (Randall 2021) we inspected the response of alcR plants to ethanol induction. The alcR line anyway contains an empty alcA promoter:OCT terminator cassette, providing a natural binding site (i.e. the alcA promoter) for the alcR protein, to minimize off-target binding to endogenous genes (Supplementary text). The effect was negligible; the number of shared DEGs between alcR and iTPS at 4 and 6 h was 34 (22 in the same direction) and 12 (all in the same direction) (Supplementary Data Set S4). Genes with similar changes were omitted from further analyses.

Principal component (PC) analysis (Fig. 2C) revealed strong separation of ethanol-sprayed iTPS lines from control treatments along the major PC1 axis (42% of variance). PC2 (10% of variance) separated 4 and 6 h samples. Thus, induction of TPS led to rapid widespread changes in transcript abundance.

### Dissection of the iTPS response into CRF groups reveals a mix of direct and indirect responses

Despite earlier harvesting, the overall iTPS response remained unrelated to the CRF (Supplementary Fig. S5, A and B). We again assigned transcripts to CRF groups G_1_, G_2_, and G_0_ (see Supplementary Data Set S4 for assignments). The published experiments that we used to estimate CRF had been analyzed using ATH1 arrays. Further analysis of the RNAseq dataset therefore focused on genes shared with the ATH1 array. The largest subset of transcripts was assigned to G_1_, but many were assigned to G_2_ and G_0_ (4576-4470, 2887-2969 and 3573-3653 at 4 and 6 h, respectively; Supplementary Table S3). Positive correlations to CRF emerged for G_1_ and negative correlations for G_2_ (Fig. 2D; Supplementary Fig. S5B). There was strong agreement between the 4 h and 6 h response for each group (Fig. 2E, *R*^2^ = 0.92, 0.90, and 0.93 for G_1_, G_2_, and G_0_, respectively). Thus, even at early times, whilst many transcripts showed a response consistent with them responding to elevated Tre6P, many changed in a manner indicating they were indirect responses.

Many iTPS-responsive transcripts were assigned to G_0_ (i.e. apparently unresponsive to sugar). We investigated two technical explanations for this unexpected result. One is that their response to sugar is context-dependent (i.e. transcript abundance responds in opposing ways in the nine treatments used to calculate the CRF). Supplementary Fig. S6, A and B shows the individual responses of the top 10 and 100 upregulated and downregulated transcripts in the nine treatments. Most were nonresponsive across all treatments. A second explanation is that ATH1 arrays can underestimate responses for lowly expressed genes, especially in families where multiple members may cross-hybridize, masking members that do respond. Inspection of transcripts assigned to G_0_ (Supplementary Fig. S6C) revealed that the majority have abundancies comparable to transcripts in G_1_ or G_2_.

### Elevated Tre6P represses genes involved in photosynthesis and gluconeogenesis and induces nucleotide biosynthesis and ribosome assembly

**Figure 3.**
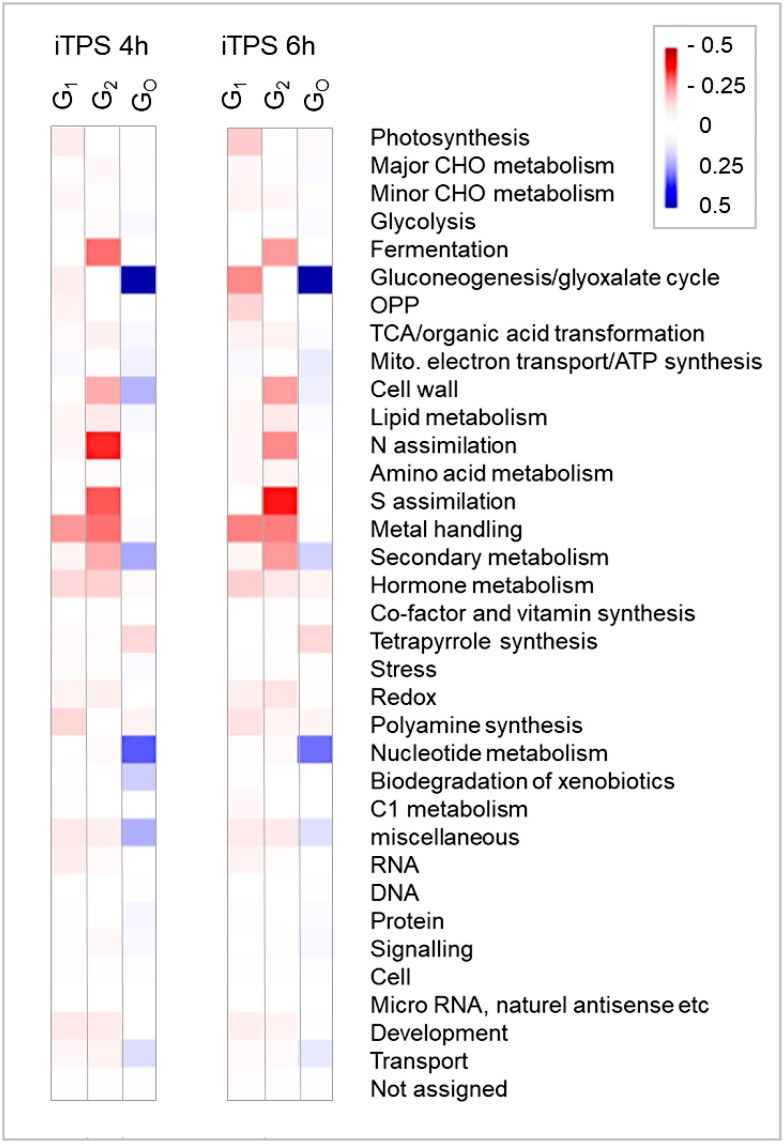
Enrichment analysis of responses at 4 and 6 h after induction of TPS. The analysis was conducted using PageMan (Usadel et al. 2006) and MapMan software (version 3.6.0RC1; https://mapman.gabipd.org/; Ath_AGI_LOCUS_TAIR10_Aug2012). The analysis was performed separately for the sets of genes that were assigned to the CRF groups G_1_, G_2_, and G_0_ (see Supplementary Fig. S2) and for the responses at 4 and 6 h after spraying. The CFR groups are shown from left to right in the block in which the 4 and 6 h response is displayed. The analyses used the log_2_FC values for all genes in a given category. These were filtered (FDR < 0.05, FC ≥ 2; all values that did not pass the filter were set to zero) and all individual FC values in a given BIN (including the values set to zero) were then averaged. The average log_2_FC values for each BIN (the upper category in the MapMan ontology) are displayed as a heat map (for scale see insert). An analysis in which a lower FC filter was used and analyses in which several higher-level categories (photosynthesis, gluconeogenesis/glyoxylate, N metabolism, nucleotide metabolism, secondary metabolism, protein, cell wall) are broken down into subcategories (subBINS) are provided in Supplementary Fig. S7. iTPS, induction of TPS; CHO, carbohydrate, OPP, oxidative pentose phosphate pathway; TCA, tricarboxylic acid cycle; N, nitrogen; S, sulfur.

We explored whether assignment of genes to G_1_, G_2_, and G_0_ allows areas of metabolism or cellular function to be identified that respond to elevated Tre6P, as opposed to indirect effects. We first used the PageMan tool (Usadel et al. 2006; https://mapman.gabipd.org/pageman) to visualize the response of genes assigned to different categories (BINs) in the MapMan ontology (Thimm et al. 2004; Schwache et al. 2019). All transcripts that failed to pass a filter of FDR < 0.05 and FC ≥ 2 were set as zero before averaging FC in each category. When we performed the analysis at the highest level of the MapMan ontology, different patterns emerged for G_1_, G_2_, and G_0_ (Fig. 3). For example, in G_1_ genes involved in photosynthesis and gluconeogenesis were repressed, whereas in G_2_ genes involved in fermentation, cell wall, lipid metabolism, N-assimilation, S-assimilation, and specialized metabolism were repressed.

**Figure 4.**
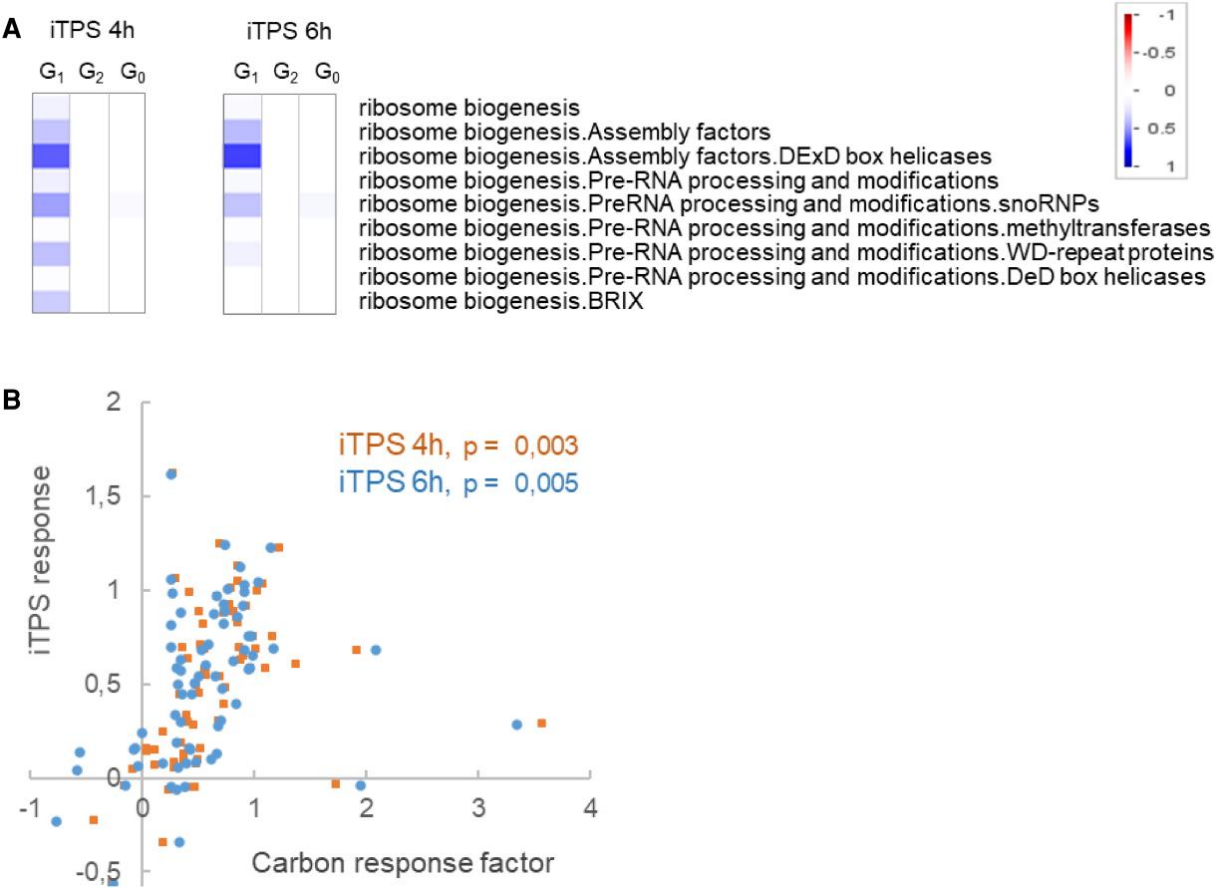
Induction of TPS leads to upregulation of ribosome biogenesis at the transcript level. The plots show changes in transcript abundance after induction of TPS (iTPS) for genes assigned to ribosomal proteins, ribosome biogenesis, and ribosomal RNA in the MapMan ontology. For each transcript, the response was calculated as the average change in ethanol-sprayed iTPS plants (induced) compared to water-sprayed iTPS plants (control) at 4 or 6 h after spraying. **A)** Coordinated responses in subBINs associated with ribosome biogenesis. The analysis was performed using PageMan (Usadel et al. 2006); the shading indicates the average change in transcript abundance for genes assigned to a given subBIN. As in Fig. 3, for genes that did not pass the combined FDR < 0.05 and log_2_FC ≥ 2 the FC value was set as zero before calculating the average response. An analysis using a lower FC filter is provided in Supplementary Fig. S7G. **B)** Comparison of iTPS response for genes assigned to ribosome biogenesis compared to their CRF (see Supplementary Fig. S2), both on a log_2_ scale. The iTPS responses at 4 and at 6 h after spraying are shown in brown and blue, respectively.

MapMan BINs group genes involved in a given process, irrespective of their precise function. We inspected selected BINs at higher resolution, using a relaxed filter (FDR < 0.05, log_2_ FC ≥ 0.2, Supplementary Fig. S7, see Supplementary text for details). In the G_1_ response, elevated Tre6P repressed photosynthesis and related functions like tetrapyrrole, tocopherol, and carotenoid biosynthesis and plastid ribosome biogenesis, repressed gluconeogenesis, and anthocyanin biosynthesis, and induced genes for nucleotide biosynthesis. Elevated Tre6P induced cytosolic and mitochondrial ribosomal proteins and ribosome assembly factors (Fig. 4; Supplementary Fig. S7G). There were widespread changes in transcript abundance for genes involved in sucrose transport and metabolism, flowering and the circadian clock, with many being assigned to G_1_ (Supplementary Figs. S8 to S10). In contrast, many genes involved in nitrate and ammonium assimilation, large sectors of specialized metabolism like phenylpropanoid, flavonoid, and glucosinolate biosynthesis and cell wall modification were assigned to G_2_. These responses were presumably indirect.

We performed gene ontology (GO) analysis after filtering by FDR < 0.05 and FC ≥ 2 (Supplementary Fig. S11, Supplementary Dataset S5). This confirmed responses identified by PageMan analysis, and highlighted further responses. In the G_1_ gene set, enriched upregulated categories included mitochondrial RNA modification and gene expression. Enriched downregulated categories included clock entrainment, light responses, and several hormone-related responses. In the G_2_ gene set, enriched upregulated categories were related mainly to stress, and enriched downregulated categories included nitrate assimilation, nucleotide salvage, glucosinolate biosynthesis, flavonoid metabolism, cell wall loosening, pectin synthesis, cutin and wax biosynthesis, indole acetic acid biosynthesis, brassinosteroid biosynthesis, auxin transport, gibberellic, and jasmonic acid signaling.

**Figure 5.**
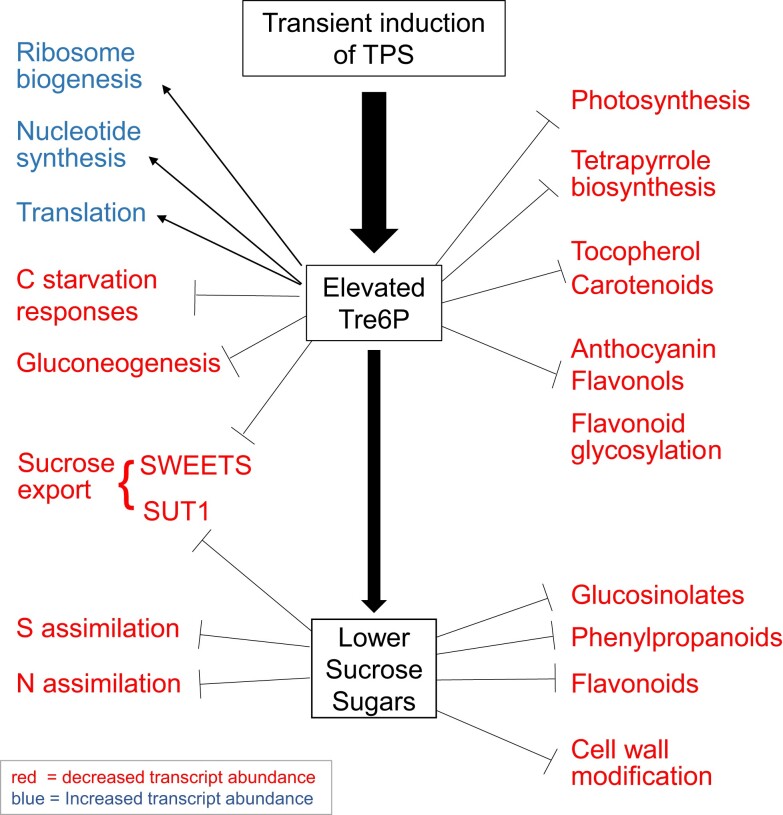
Summary of the metabolic and growth-related responses to an increase in Tre6P. Categories of genes that respond directly to elevated Tre6P were inferred from their assignment to CRF G_1_, while categories of genes that respond to the concomitant fall in sucrose were inferred from their assignment to CRF G_2_. Upregulated and downregulated gene categories are shown in blue and red, respectively (see Supplementary Fig. S2 and Supplementary Data Set S4).

As summarized in Fig. 5, elevated Tre6P leads to repression of the photosynthetic machinery, repression of gluconeogenesis, complex changes in sucrose metabolism and transport, stimulation of nucleotide biosynthesis and stimulation of ribosome biogenesis in the cytosol and mitochondria. The increase of Tre6P is accompanied by a decrease of sucrose and glycolytic intermediates, like Glc6P, whereas many organic acids and amino acids were increased (see above). These probably trigger widespread secondary changes including repression of genes for N and S metabolism, specialized metabolism, and cell wall loosening.

### Comparison with the response to constitutive overexpression of bacterial TPS

**Figure 6.**
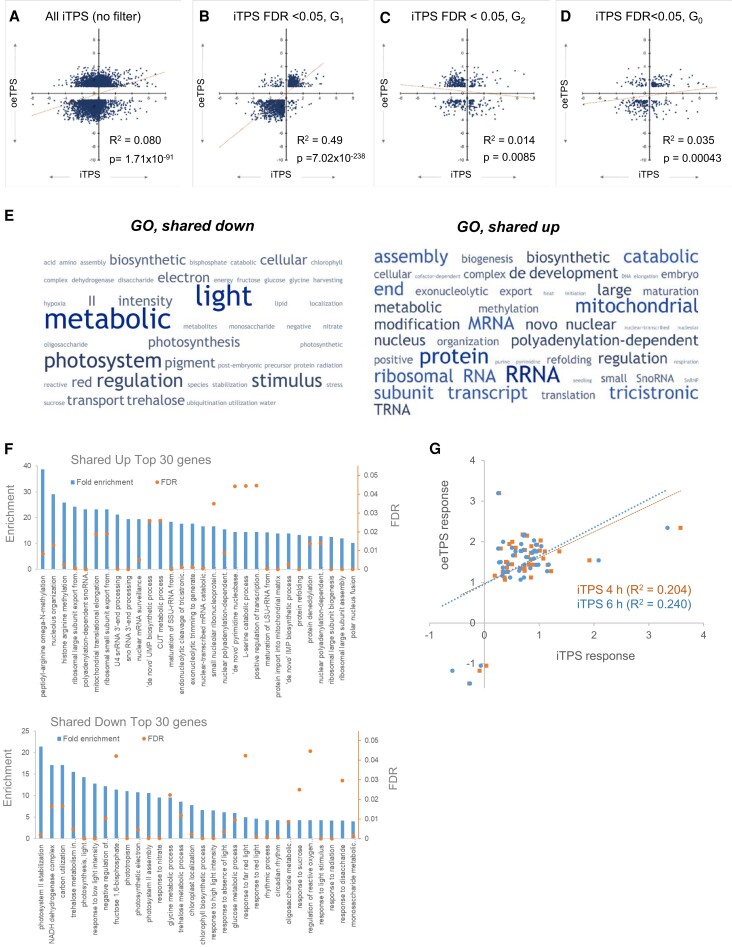
Comparison of the response to an induced increase in Tre6P and constitutive overexpression of a bacterial TPS. The response of transcript abundance to constitutive overexpression of a bacterial TPS (oeTPS) is taken from Zhang et al. (2009) who harvested 7-d-old seedlings growing in liquid culture under continuous light. **A)** oeTPS response plotted against the iTPS response (response to an induced increase in Tre6P) at 4 h after induction of TPS. Of the 5.2K responsive transcripts reported by Zhang et al. (2009), 4,966 were found in the iTPS response data set. No FDR filter was applied to the iTPS dataset for this plot. **B to D)** oeTPS response plotted against the iTPS response for the 2,437 transcripts that responded significantly (FDR < 0.05) at both 4 and 6 h after spraying (termed “iTPS 4-6h” in the display). Data were plotted separately for each CRF group of genes: (B) 1,596 transcripts assigned to CRF G_1_, (C) 494 transcripts that were assigned to CRF G_2_, and (D) 347 transcripts that were assigned to CRF G_0_. Transcripts were assigned to CRF G_1_, G_2_, and G_0_ as explained in Supplementary Fig. S2. The iTPS response of transcripts in G_1_ is probably a direct response to elevated Tre6P, in G_2_ to lower sugar and G_O_ to more complex interactions. Plots of oeTPSA against the individual 4 and 6 h iTPS responses are provided in Supplementary Fig. S12, A and B. **E, F)** Enriched pathways based on GO. The analysis was performed for DEGs from the oeTPS data set of Zhang et al. (2009) that were assigned to G_1_ in both iTPS datasets (4 and 6 h). **E)** This shared set of transcripts was analyzed using the TagCrowd online tool (https://tagcrowd.com/) to identify frequently occurring terms among the gene names and descriptions and are shown in a word map with the font size representing the frequency. **F)** Histogram depicting the fold enrichment (left *y*-axis) and *P*-value (right *y*-axis) of the top 30 enriched processes. An analysis of all enriched processes is provided in Supplementary Fig. S12D. **G)** Comparison of the oeTPS (Zhang et al. 2009) and iTPS responses for genes assigned to ribosome biogenesis, both plotted on a log_2_ scale. The plot shows the iTPS response at 4 and at 6 h after spraying. The number of genes shown in this display is less that in panel B because not all of the genes in the iTPS response were present in the data set of Zhang et al. (2009). Although the oeTPS data of Zhang et al. (2009) showed the strong response of ribosome biogenesis, this was not explicitly noted at the time because assignment of genes to the ribosome biogenesis category was very incomplete in the ontology that they used.

We compared the iTPS response with a published response to constitutive overexpression of bacterial TPS in seedlings (termed hereafter the oeTPS response) (Zhang et al. 2009; Paul et al. 2010). This earlier study profiled transcript abundance with the CATMA array, which uses cDNA probes (Allemeersch et al. 2005). It shortlisted 5,273 genes (FDR < 0.05, FC ≥ 2) of which 4,966 were found in the iTPS dataset. A scatter plot of the oeTPS and iTPS responses revealed poor agreement (Fig. 6A; Supplementary Fig. S12A). About half (2,559) did not pass the FDR < 0.05 filter in the iTPS dataset, 1,596 were assigned to G_1_, 484 to G_2_, and 347 to G_0_ (Supplementary Table S5). Genes assigned to G_1_ showed good agreement between the oeTPS and iTPS responses (*R*^2^ = 0.49, *P* = 7.02 × 10^−238^; Fig. 6B, Supplementary Fig. S12B, Supplementary Table S5; only 72 (4.5%) showed reciprocal responses). There was little agreement between oeTPS and iTPS for genes assigned to G_2_ or G_O_ (Fig. 6, C and D; Supplementary Fig. S12B, Supplementary Table S5). We conclude about 30% of responses to constitutive oeTPS were to elevated Tre6P whilst the rest were probably indirect.

oeTPS repressed genes involved in photosynthesis, the glyoxylate cycle and gluconeogenesis, and induced genes involved in mitochondrial electron transport, amino acid synthesis, nucleotide synthesis and protein synthesis (Zhang et al. 2009). Our re-analysis shows many of these responses are due to Tre6P signaling. Paul et al. (2010) noted that seedlings with constitutive overexpression of TPS were stunted, leading them to investigate transcripts for light- and auxin signaling and cell wall biosynthesis. The responses were partly confirmed in the iTPS response. Many genes involved in light signaling were repressed in both oeTPS and iTPS and assigned to G_1_ (Supplementary Fig. S12C, Supplementary Text). Responses in auxin signaling and cell wall modification included many indirect effects (Supplementary Fig. S7H, S11, and S12, D and E, Supplementary text).

Genes that respond to short-term elevation of Tre6P are assigned to G_1_, and respond in a qualitatively similar manner to constitutive oeTPS represent a robust set of Tre6P-regulated genes (listed in Supplementary Data Set S6). They were used for GO analysis (Fig. 6, E and F, Supplementary Fig. S12D, Supplementary Data Set S7). Downregulated processes included photosynthesis, chlorophyll, and pigment metabolism, C-utilization, monosaccharide metabolism, generation of precursor metabolites and energy, amino acid catabolism, various light responses and the circadian clock. The most enriched upregulated process was cellular component organization/biogenesis, which includes ribosome biogenesis and mitochondrial biogenesis.

### Impact on trehalose metabolism

**Figure 7.**
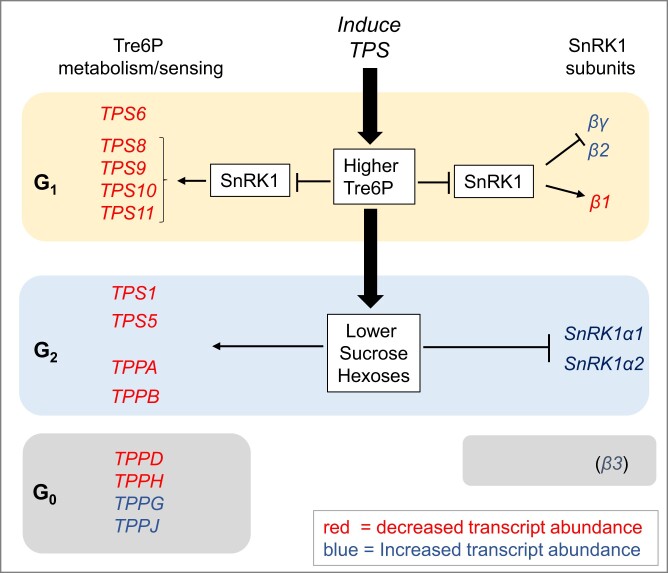
Schematic overview summarizing responses of transcript encoding proteins involved in Tre6P metabolism and subunits of the SnRK1 complex. *TPS* and *TPP* genes and SnRK1 subunit genes whose transcripts responded to elevated Tre6P were assigned to CRF groups G_1_, G_2_, and G_0_. All showed a significant change (FDR < 0.05), transcripts showing a FC ≥ 2 are highlighted as bold. Based on responses to transient overexpression of SnRK1α1 (Baena-González et al. 2007), it could be inferred that most of the genes in CRF group G_1_ were responding due to inhibition of SnRK1 by Tre6P. The genes in CRF group G_2_ are probably responding to the decrease in sucrose and other sugars that follows an induced rise in Tre6P levels, rather than the rise in Tre6P per se. Genes in CRF group G_0_ respond to Tre6P but their expression appears not to be regulated by sugars. The response of SnRK1β3 is shown in brackets because it is inconsistent at 4 and 6 h. Upregulated and downregulated genes are shown in blue and red, respectively. The display is based on data provided in Supplementary Figs. S13 and S14A.

We investigated C-signaling pathways that might be involved in the response to elevated Tre6P. Tre6P was elevated by induced expression of a bacterial TPS. We asked how the endogenous pathway responds to this suddenly imposed increase in Tre6P (Fig. 7; Supplementary Fig. S13). TPS1 is responsible for synthesis of Tre6P. *TPS1* was repressed >2-fold but assigned to G_2_ indicating this was an indirect response. Class II TPSs (TPS5-11) are thought to have a regulatory function. *TPS8-11* were repressed >2-fold and assigned to G_1_. Several *TPP*s were assigned to G_2_ or G_0_, with five being repressed >2-fold. These observations point an imposed increase in Tre6P leading to rewiring of Tre6P metabolism (see Supplementary text for details).

### Impact on SnRK1 expression

Tre6P is known to inhibit SnRK1 activity in vitro and this interaction can underlie at least some SnRK1 signaling functions (see Introduction). We inspected the relationship between the iTPS response and SnRK1 signaling. We first asked if Tre6P modifies SnRK1 expression (Fig. 7; Supplementary Fig. S14A). SnRK1 is a heterotrimer containing one of two alternative catalytic subunits (SnRK1α1, SnRK1α2), one of three alternative regulatory β-subunits (SnRK1β1, SnRK1β2, SnRK1β3) and a regulatory SnRK1βγ subunit (Polge et al. 2008; Broeckx et al. 2016; Nietzsche et al. 2016; Wang et al. 2020). In the iTPS response, three regulatory subunits were assigned to G_1_, with SnRK1β1 transcript decreasing >2-fold by 6 h and SnRK1β2 and SnRK1βγ showing a weak increase. Thus, Tre6P modifies the relative expression of SnRK1 regulatory subunits.

### Comparison of the iTPS response with the response to transient SnRK1 overexpression

We asked how much of the iTPS response can be explained as a consequence of inhibition of SnRK1 by Tre6P. The published response to transient *SnRK1α1* overexpression in protoplasts (Baena-Gonzalez et al. 2007; hereafter termed the tSnRK1α1 response) has been widely used to define transcriptional events downstream of SnRK1. Supplementary Fig. S14B summarizes the iTPS response of the top 25 responders from Baena-Gonzalez et al. (2007) plus four genes widely used as SnRK1 markers (*DIN1*, *DIN3*) or studied as players in C-starvation responses (*bZIP11*, *bZIP63*) (Ma et al. 2011; Mair et al. 2015). Of these 29 genes, 14 are induced by tSnRK1α1 and 12 of these were repressed by iTPS and assigned to G_1_, consistent with Tre6P inhibiting SnRK1. A less consistent picture emerged for the 15 genes that are repressed by tSnRK1α1. Five were assigned to G_1_ and induced, consistent with Tre6P inhibiting SnRK1. Five were assigned to G_2_ and repressed, consistent with inhibition of SnRK1 by signals deriving from low sugars or other side-effects. One was assigned to G_0_, and three did not show a significant response. Despite the mixed response of SnRK1-repressed genes, the main conclusion is that 17 of 29 genes were assigned to G_1_ and responded in the direction predicted if Tre6P inhibits SnRK1 in vivo.

**Figure 8.**
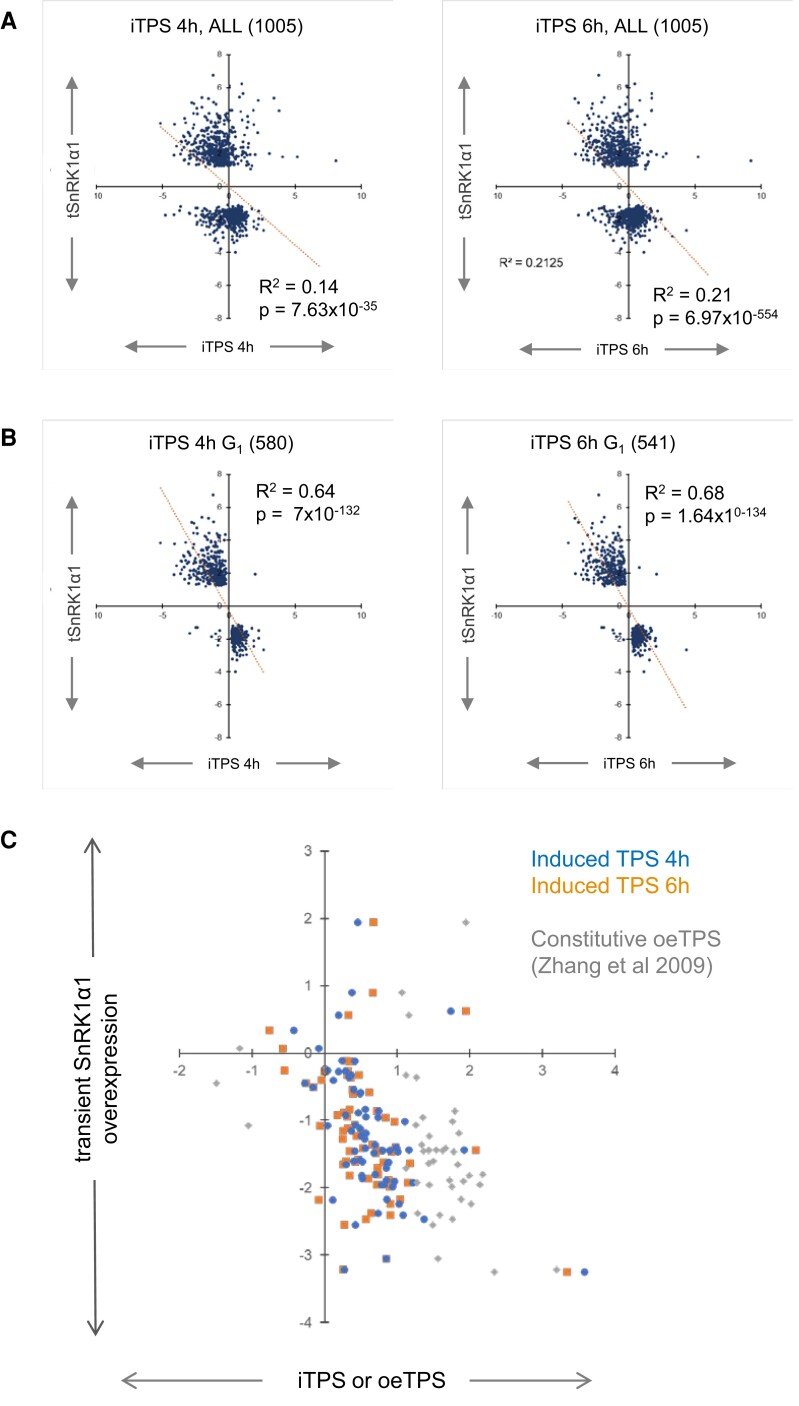
Response of SnRK1-regulated transcripts to elevation of Tre6P levels. A list of SnRK1-regulated transcripts was drawn up based on the data for the response to transient overexpression of SnRK1α1 in Arabidopsis mesophyll protoplasts (Baena-Gonzalez et al. 2007, here termed the tSnRK1 α1 response). A total of 1,001 of these transcripts was retrieved in the unfiltered iTPS data set. **A)** Regression plot for all 1001 genes of the tSnRK1α1 response versus the response at 4 h (left and 6 h (right) after spraying iTPS29.2 with ethanol (iTPS response). In the 4 and 6 h iTPS samples, 763 and 762 transcripts, respectively, showed a qualitatively opposite response to their tSnRKα1 response, whilst 242 and 243 transcripts, respectively, showed a qualitatively similar response to their tSnRK1α1 response. **B)** Regression plot for all 1,001 DEGs of the tSnRK1α1 response versus the filtered G_1_ iTPS response. Transcripts were filtered (FDR > 0.05, log_2_FC ≥ 0.2) and then compared with the CRF (Supplementary Fig. S2) to assign transcripts to G_1_ (i.e. transcripts whose iTPS response is qualitatively similar to their CRF and probably a direct effect of elevated Tre6P). A total of 580 and 541 transcripts were assigned to G_1_ in the iTPS 4 and iTPS 6 h data sets, respectively. Of these transcripts, at 4 and 6 h iTPS, the vast majority (571 and 532, respectively) showed a qualitatively opposite response to their tSnRK1α1 response, whilst at both times only nine transcripts showed a qualitatively similar response to their tSnRK1α1 response. Further information about these analyses and the correlations between tSnRK1α1 response and transcripts assigned to iTPS CRF groups G_2_ and G_O_ is provided in Supplementary Fig. S14 and Supplementary Table S6. **C)** Regression plots of the tSnRK1α1 response and the iTPS and oeTPS1 response (response to constitutive overexpression of TPS, see Fig. 6) of genes encoding ribosome assembly factor. The plot shows all 74 genes assigned to the subBIN “ribosome biogenesis” in the MapMan TAIR10 ontology. Of these, 54 were assigned to CRF group G_1_ and 10 to CRF group G_0_, respectively, in at least one of the two iTPS treatments, and only four were unassigned. The responses in the 4 and 6 h iTPS treatments were similar and those in the oeTPS response were qualitatively similar but stronger than in the induced treatments. As reported in Baena-González et al. (2007), tSnRK1α1 represses ribosome assembly genes (see also Supplementary Fig. S14I). The vast majority of the changes in response to overexpression of TPS were therefore reciprocal to the response to tSnRK1α1.

Baena-González et al. (2007) listed 1,021 potential SnRK1 downstream targets, of which 1,004 were present in the iTPS dataset. Comparison of the tSnRK1α1 response with the complete iTPS response revealed a weak negative trend (*R*^2^ = 0.14 and 0.21 at 4 and 6 h, respectively, Supplementary Table S6). Many transcripts showed a qualitatively similar rather than the expected reciprocal response (Fig. 8A). For transcripts assigned to G_1_, a very strong negative correlation emerged (580 and 542 genes, *R*^2^ = 0.64 and 0.68, *P* = 7.6 × 10^−132^ and 7.0 × 10^−54^ in the 4 and 6 h iTPS response, respectively) (Fig. 8B; Supplementary Table S6). A similar picture held for the top 100 tSnRK1α1 responders (Supplementary Fig. S14C). These genes presumably represent downstream targets where SnRK1 signaling is inhibited by elevated Tre6P. An analogous analysis for G_2_ yielded a relatively good positive regression (144 and 151 genes, *R*^2^ = 0.41 and 0.29, *P* = 2.9 × 10^−18^ and 1.44 × 10^−12^, respectively, Supplementary Table S6, Supplementary Fig. S14D). These presumably represent genes that are downstream of SnRK1 and whose response is not counteracted by elevated Tre6P but is instead promoted by low sugars or other side effects. Transcripts assigned to G_0_ yielded a very weak relationship (22 and 22 genes, *R*^2^ = 0.042 and 0.074, *P* = 0.36 and 0.22, respectively; Supplementary Table S6, Supplementary Fig. S14D).

Global analysis with the >500 genes assigned to G_1_ revealed a higher proportion of expected responses (i.e. iTPS qualitatively opposite to tSnRK1α1) for genes that are induced by tSnRK1α1 than for genes that are repressed by tSnRK1α1 (Supplementary Fig. S14E). This points to Tre6P playing a large role in SnRK1 signaling that induces genes, but a smaller role in SnRK1 signaling that represses genes. We compared the iTPS and tSnRK1α1 responses for genes assigned to photosynthesis, light signaling, or cytosolic ribosomal proteins (Supplementary Fig. S14, F to H). The responses tended to be reciprocal, but there were large differences in magnitude resulting in low correlation coefficients. The strongest correlation (*R*^2^ = 0.3 to 0.42) was for cytosolic ribosomal proteins. Many ribosome assembly factors are induced by sugars and repressed by tSnRK1α1 (Supplementary Fig. S14I). Ribosome assembly factors were induced by transient and constitutive elevation of Tre6P, and responded reciprocally to tSnRK1α1 (Fig. 8C) consistent with Tre6P inhibition of SnRK1. As already mentioned, increased C-availability leads to repression of *TPS8 to TPS11* and a switch from the β1 to the β2 subunit of SnRK1. Five TPS class II genes (*TPS6*, *TPS8 to TPS11*) and the β1 and β2 subunits of SnRK1 responded in a way consistent with Tre6P inhibiting SnRK1 (Supplementary Fig. S14J).

### Interactions with TARGET OF RAPAMYCIN COMPLEX

TARGET OF RAPAMYCIN COMPLEX (TORC) acts as a counterpart to AMPK/SNF1/SnRK1 to promote ribosome biogenesis and growth in eukaryotes (Sabatini 2017; Ryabova et al. 2019; Wu et al. 2019; Meng et al. 2022). Emerging evidence points to multiple interactions between TORC and SnRK1 in plants (Nukarinen et al. 2016, Wang et al. 2018; Belda-Palazón et al. 2020, 2022; Morales-Herrera et al. 2023). Tre6P had little effect on expression of TORC (Supplementary Fig. S15A, Supplementary text). Inspection of the responses of known post-translational targets of TORC (Supplementary Fig. 15, B to E) revealed that Tre6P repressed several members of the ABA-receptor family.

We inspected two published transcriptional responses (Caldana et al. 2013; Dong et al. 2015) to inactivation of TORC (Supplementary Fig. S15, F to J, Supplementary text). If TORC acts in a similar manner to Tre6P and reciprocally to SnRK1, the response to TORC inactivation should be qualitatively reciprocal to that to elevated Tre6P and similar to that to tSnRK1α1. The response to TORC inactivation partly followed this pattern but was partly unrelated and partly opposite. Furthermore, comparison of the TOR-inactivation response with the CRF indicated that C-supply is just one of many inputs to TORC signaling.

### FCS-LIKE ZINC FINGER (FLZ) proteins

FLZ proteins are negative regulators of SnRK1 and implicated in its interaction with TORC (Jamsheer et al. 2015, 2022; Nietzsch et al. 2016). Comparison of their CRFs and iTPS and tSnRK1α1 responses confirmed their expression is regulated by C-status and pointed to Tre6P-inhibition of SnRK1 regulating a subset that is induced in high sugar (Supplementary Fig. S16, Supplementary text).

### Comparison with bZIP11 signaling

S_1_ and C class bZIP proteins play an important role in low energy signaling (Dröge-Laser and Weiste 2018). S_1_ bZIPs are translationally regulated by sucrose, which binds at upstream open reading frames (uORFs) to stall ribosome progression (Wiese et al. 2004; Rahmani et al. 2009). When sucrose falls, S_1_ bZIPs are translated and dimerize with C bZIPs to transcriptionally activate starvation responses and inhibit growth (Hanson et al. 2008; Ma et al. 2011, Dröge-Laser and Weiste 2018). We compared the iTPS response with the response to constitutive overexpression of bZIP11 (termed oebZIP11, data from Ma et al. 2011) (Supplementary Fig. S17). Many genes that respond to oebZIP11 also responded to iTPS, with some being assigned to G_1_ (see Supplementary text for details).

### Transcription factors

Over 400 transcription factors (TFs) showed a FC ≥ 2, with 141 being assigned to G_1_ (Supplementary Fig. S18). These were analyzed using GO and STRING (Supplementary Fig. S19). STRING utilizes available datasets, text mining, and computational predictions from various organisms to score the likelihood of association between proteins (Szklarczyk et al. 2021). The analyses highlighted TFs that regulate carbohydrate and C-signaling, biosynthesis (chlorophyll, anthocyanin), the circadian clock, and signaling related to light (shade avoidance, red/far-red, blue), hormones (auxin, gibberellic acid, ethylene, ABA, jasmonic acid), and development (flowering, phloem or xylem development) (see Supplementary text). Many C and S_1_ bZIP family members including *bZIP1*, *bZIP2*, *bZIP9*, *bZIP25*, and *bZIP63* showed >2-fold changes in transcript abundance (Supplementary Fig. S20, A and B). *bZIP1 bZIP25* and *bZIP63* showed a reciprocal response to tSnRK1α1, consistent with Tre6P-inhibition of SnRK1. This pattern was seen for many other TFs (Supplementary Fig. S21). Analyses of the G_2_ response highlighted TFs assigned to processes like water relations, N metabolism, S starvation, and specialized metabolism (Supplementary Figs. S21, S22, and S23, Supplementary Data Set S8, Supplementary text).

## Discussion

### Elevation of Tre6P leads to widespread changes in transcript abundance

Tre6P is a sucrose signal with a central role in the regulation of plant metabolism, growth, and development (Fichtner and Lunn 2021). Insights into the transcriptional response to Tre6P were provided by analyses of plants with constitutive TPS overexpression (Zhang et al. 2009; Paul et al. 2010). However, these plants showed strong growth and developmental phenotypes (see also Schleupmann et al. 2003; Yadav et al. 2014). Furthermore, elevated Tre6P leads to major changes in metabolism including decreased levels of sugars and glycolytic intermediates and increased organic acids and amino acids (Figueroa et al. 2016; Ishihara et al. 2022) that may trigger secondary changes in expression. We have used Arabidopsis plants expressing TPS under the control of an ethanol-inducible promoter to investigate the short-term response of the transcriptome to elevation of Tre6P.

The induction system was developed to avoid artifacts due to unphysiologically high levels of Tre6P (Martins et al. 2013). However, TPS protein is induced in a wide range of cell types. AtTPS1, the endogenous protein that synthesizes Tre6P in Arabidopsis, is mainly expressed in the companion cells and phloem parenchyma (the phloem-loading zone) and guard cells in leaves (Fichtner et al. 2020). Although it is likely that Tre6P moves via plasmodesmata into neighboring cell types, there might be spatial gradients that are overridden in our induction system. Despite this caveat, our inducible system enables us to investigate primary responses more easily than in plants with constitutively elevated Tre6P. Future studies might combine inducible and cell-specific expression to gain insights into spatial aspects of Tre6P signalling.

Almost half the detected transcripts showed significant changes and >5,000 showed >2-fold changes in abundance 4 h after ethanol spraying (Fig. 2A), i.e. within 2 h of the first detectable rise in Tre6P (Fig. 1). The increase in Tre6P was accompanied by a decline in sucrose (Fig. 1) and changes in the levels of other metabolites (Supplementary Fig. S4) that might themselves lead to changes in gene expression. Thus, the inducible system and rapid sampling did not entirely remove complications due to secondary changes. This may be unavoidable, due to rapid post-translational regulation of metabolism by Tre6P (Figueroa et al. 2016).

### Deconvolution of Tre6P-dependent and indirect responses

To distinguish responses to elevated Tre6P from responses to lower sucrose and other indirect effects, we compared the response to induction of TPS (iTPS) with the response to increased sugar availability. To do this we calculated an average response, which we termed a CRF, across nine published treatments that modified sugar levels whilst minimizing confounding changes in light- or circadian-signaling (Supplementary Fig. S2). We considered transcripts that showed a qualitatively similar response to iTPS and elevated sugar to be targets of Tre6P-signaling (CRF-group G_1_), whilst transcripts that showed opposite responses were more likely to be responding to indirect effects like the decline in sucrose (G_2_). Assignment to G_1_ or G_2_ does not mean that a gene is regulated only by Tre6P or only by signals from sucrose or other indirect effects; it is possible that in some cases expression is regulated by both, and the observed change depends on the relative strengths of the responses to 3- to 4-fold elevated Tre6P compared to a 30% decrease in sucrose or other secondary effects. Overall, of the responding transcripts, about 40% responded in a manner consistent with a response to elevated Tre6P and about 27% in a manner consistent with them responding to lower sucrose or other indirect effects. About 33% could not be assigned to either response type because they did not show an obvious response to a change in sugar levels. We conclude that there are massive transcriptional responses within 2 h of Tre6P levels starting to rise, but also massive indirect effects.

It was unexpected that many transcripts responded to iTPS but did not respond to elevated sugar. This is partly due to two technical issues. First, some transcripts responded to sugars in a context-dependent manner, rising in some and falling in other treatments. Second, some responses may have been missed because the CRF was calculated using data from ATH1 arrays, which can be insensitive to changes of low-abundance transcripts. However, these technical factors explained only a small proportion of the unexpected responses (Supplementary Fig. S6). It remains possible that some genes respond to sugars in other conditions. It is also possible that part of the iTPS response is due to changes in organic acids or amino acids, rather than sugars. Another explanation is that their expression is regulated in an opposing manner by Tre6P and by signals that change in concert with sucrose. In most situations in wild-type plants, such genes would not respond strongly or consistently because sucrose and Tre6P usually change in parallel (Lunn et al. 2014; Figueroa et al. 2016; see also Introduction). This idea is supported by the observation that some genes assigned to G_0_ respond to other perturbations of the C-signaling network, like transient overexpression of SnRK1α1, inactivation of TORC or overexpression of bZIP11 (Supplementary Figs. S14D, S15J and S17C).

We revisited a published microarray study of Arabidopsis with constitutive TPS overexpression (Zhang et al. 2009) (Fig. 6, Supplementary Fig. S12). In this study, ∼5,000 transcripts showed >2-fold changes in abundance. In our study, about 30% of them responded directly to elevated Tre6P (i.e. were assigned to G_1_ and showed qualitatively similar changes in constitutive and induced responses) (Fig. 6B). This agreement is striking as different tissues (seedlings versus rosettes), growth conditions, and RNA analysis techniques were employed. There was little overlap between the induced and constitutive response G_2_ and G_0_; these contained only 10% and 7%, respectively, of the genes listed in Zhang et al. (2009) and many changed in opposite directions in the induced and constitutive responses (Fig. 6, C and D). Over 50% of the transcripts that responded to constitutive overexpression of TPS did not show significant changes in the induced response and presumably reflect indirect responses to long-term elevation of Tre6P. Overall, this comparison identified about 1,500 genes that respond robustly to short- and long-term elevation of Tre6P, but also highlighted the importance of studying short-term responses and of distinguishing direct from indirect responses.

### When C availability increases, Tre6P-dependent and -independent signaling act collectively to promote biosynthesis, growth, and defense

Genes whose expression was regulated by Tre6P were from different functional categories to those that showed an indirect response. Metabolic processes predicted to be regulated by Tre6P included repression of photosynthesis and associated processes like chlorophyll, tocopherol and flavanol biosynthesis, repression of gluconeogenesis, induction of nucleotide synthesis, repression of anthocyanin biosynthesis, and repression of sucrose export especially *SWEET*s (Figs. 3 and 5;Supplementary Figs. S7, B to F and S11). In contrast, N-assimilation, S-assimilation, and large sectors of specialized metabolism, including glucosinolate, phenylpropanoid, and flavonoid biosynthesis, were predicted to be regulated by indirect effects (Fig. 5; Supplementary Fig. S7, C to F). Growth-related processes that were induced by Tre6P included mitochondrial biogenesis and ribosome biogenesis (Figs. 4 and 6;Supplementary Fig. S7G), whereas indirect responses included repression of cell wall modification (Supplementary Figs. S7, H, S11 and S12).

In a wild-type plant, Tre6P and sucrose usually change in parallel (see Introduction). For transcripts assigned to G_1_, the response of wild-type plants to rising C availability is predicted to resemble their iTPS response. For transcripts assigned to G_2_, the response of wild-type plants to rising C availability will be qualitatively opposite to their iTPS response. This is illustrated in Supplementary Fig. S24A, which is derived from Fig. 5 but with the direction-of-change reversed for processes assigned to G_2_. As C-availability rises, Tre6P-mediated and Tre6P-independent sugar-signaling act in concert to decrease investment in the photosynthetic apparatus and promote nutrient assimilation, biosynthesis, protein synthesis and cellular growth, as well as synthesis of specialized metabolites for defense purposes (see Supplementary text for discussion of examples).

Tre6P also acts transcriptionally on many signaling functions including the circadian clock, light-, ABA- and auxin-signaling and floral induction. These categories were highlighted in our analyses both of the total transcriptome and of TFs (Fig. 6, E and F, Supplementary Figs. S9 to S12, S15 and S18 to S19, for details see Supplementary text.

### Impact on the Tre6P biosynthesis and signaling pathway

Introduction of a heterologous TPS will disturb the network that regulates and balances Tre6P levels with the production and use of sucrose. *TPS1* encodes the main enzyme responsible for synthesizing Tre6P (see Introduction). *TPS1* was repressed in the iTPS response but assigned to G_2_, indicating an indirect response possibly due to the decline in sucrose. Several class II TPSs (*TPS8-11*) were repressed >2-fold by elevated Tre6P, probably acting via inhibition of SnRK1 (Fig. 7; Supplementary Figs. S13 and S14). Several *TPPs* were also repressed, but often by indirect effects (Fig. 7). Overall, these observations point to large-scale rewiring of Tre6P metabolism.

In the context of the iTPS response, these transcriptional responses partly counteract the imposed increase in Tre6P. In the context of rising C-availability in wild-type plants, cooperation between Tre6P-mediated and Tre6P-independent signaling is predicted to induce *TPS1* and promote Tre6P synthesis, repress many *TPS* class II genes and induce many *TPP*s (Supplementary Fig. 24B). Based on emerging evidence that TPS class II proteins interact with and inhibit SnRK1 (van Leene et al. 2022), their repression might provide feedforward amplification within the Tre6P-SnRK1 network. Repression involves mainly *TPS8-11*, which are induced under C-starvation (Usadel et al. 2008; Cookson et al. 2016). *TPS5*, *TPS6*, and *TPS7*, which are expressed under more benign conditions, showed smaller and more varied responses (see Supplementary Fig. S8).

### Interaction between Tre6P- and SnR1K-signaling

**Figure 9.**
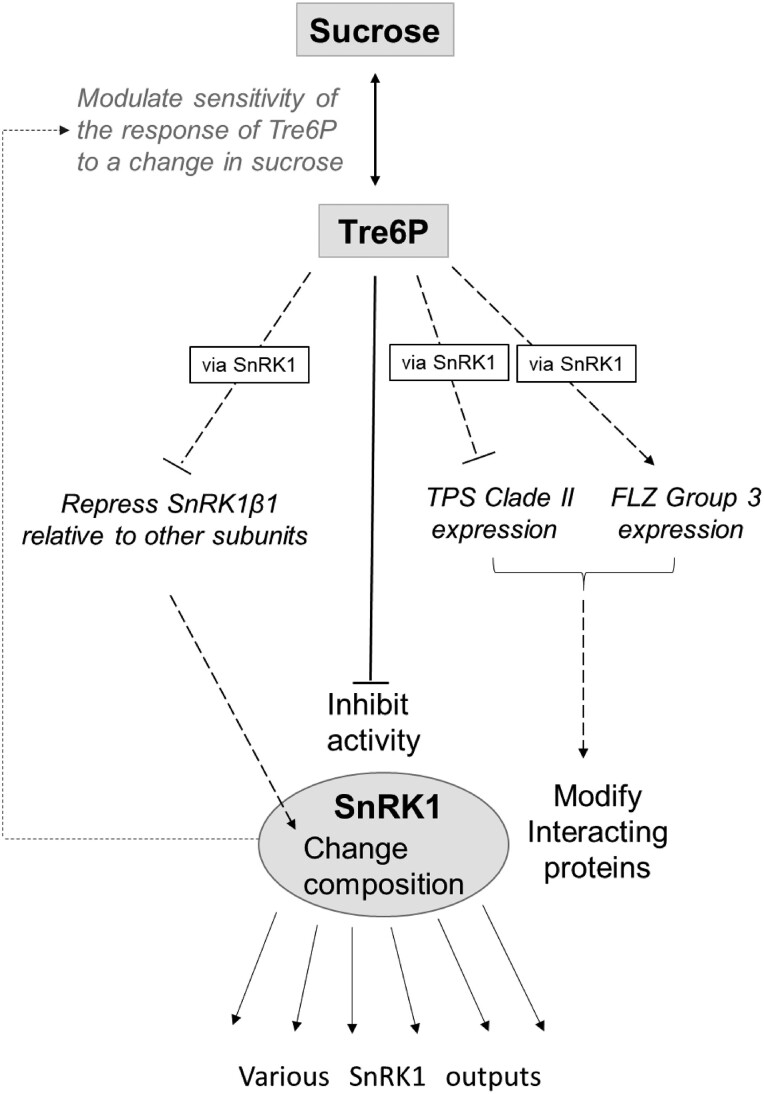
Interactions between Tre6P and SnRK1 signaling. The known inhibitory effect of Tre6P in SnRK1 activity is shown as a solid black line. In addition, Tre6P regulates the expression of the two regulatory β-subunits of SnRK1, repressing *SnRK1*β*1* and inducing *SnRK1*β*2*, which will probably lead to changes in SnRK1 complex composition. Tre6P also represses expression of several TPS Class II genes, in particular clade 2 (*TPS8*, *TPS9*, *TPS10*, *TPS11*) that were shown to physically interact with and at least in some cases may inhibit SnRK1 activity (van Leene et al. 2022). Furthermore, Tre6P induces FLZ group 3 genes, which also regulate SnRK1 activity (see Jamsheer et al. 2015, 2022; Nietzsch et al. 2016). These interactions are shown as dotted lines. The action of Tre6P on the SnRK1 β-subunits, TPS class II and FLZ group 3 expression may be mediated via inhibition of SnRK1 activity. The arrows to “various SnRK1 outputs” indicate that the changes in SnRK1 composition and interacting proteins may modify if and how they operate. In addition, it has been shown that increased expression of tSnRK1α1 dampens the response of Tre6P to sucrose (Peixoto et al. 2021) (gray line).

**Figure 10.**
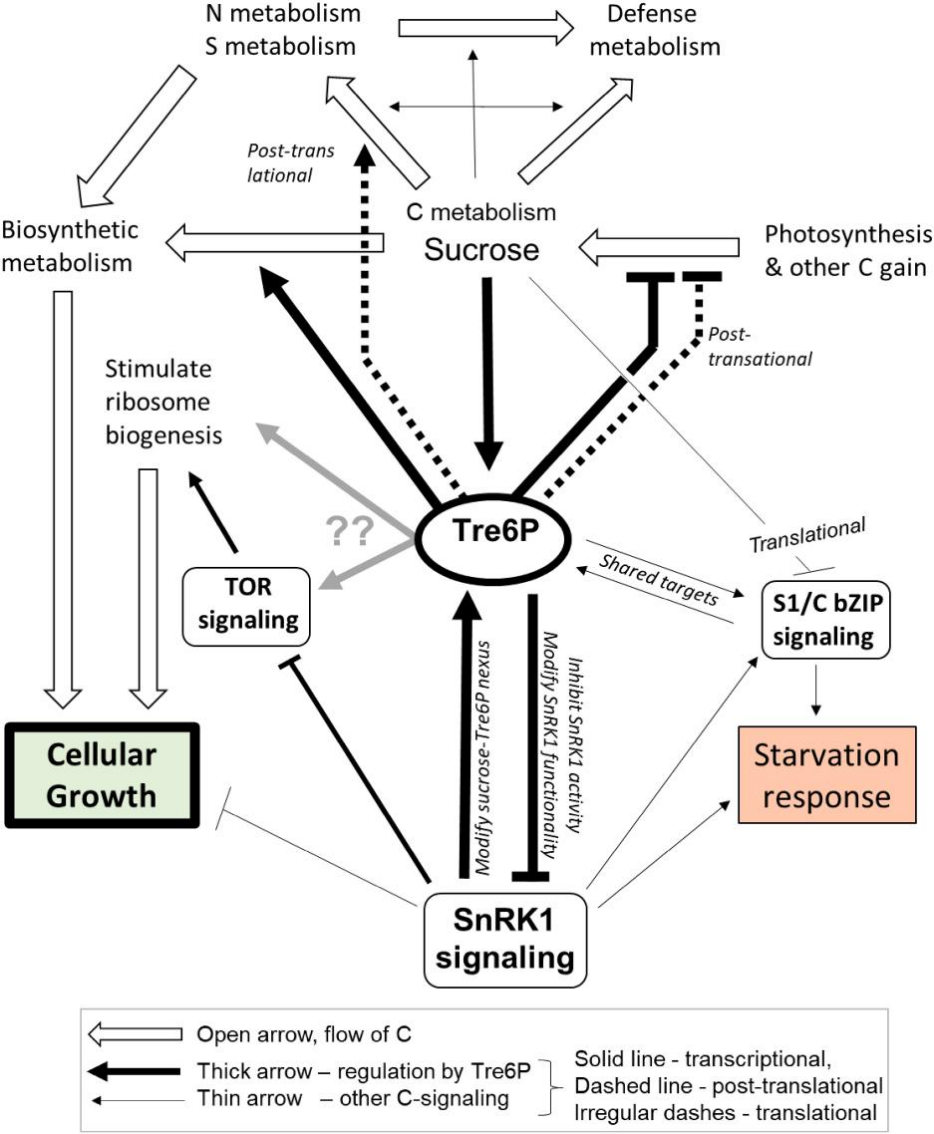
Regulation of metabolism and growth by Tre6P. Flows of C, N, and S are indicated by open arrows, transcriptional regulation, and post-translational regulation by Tre6P and thick solid arrows and thick dotted arrows, respectively. Thin solid arrows denote further C signaling. Tre6P represses expression of genes encoding components of the photosynthetic machinery and post-translationally modified starch and sucrose breakdown. Tre6P exerts positive transcriptional regulation on biosynthesis- and growth-related processes, in part by action via inhibition of SnRK1 and via links to TOR. Tre6P inhibits starvation responses via inhibition of SnRK1. The action of Tre6P on SnRK1 involves not only inhibition but also alteration of SnRK1 composition, and modification of the expression of genes encoding TPS Class II proteins and S_1_/C FLZ proteins that interact with SnRK1 and presumably modify its activity and functionality (see also Fig. 9) as well as links and overlap with type S_1_/C bZIP signaling. The induction of ribosome biogenesis may be at least partly explained by inhibition of SnRK1 by elevated Tre6P, but other mor direct links to TOR (TARGET OF RIFAMYCIN) cannot be excluded (indicated by gray arrows) The action of Tre6P on C metabolism is probably reinforced by other sugar-signaling pathways. Tre6P is not directly involved in the transcriptional regulation of N and S assimilation, but acts at a post-translational level to promote C flux to organic acids (OA) and amino acids (AA). The synthesis of major sets of specialized metabolites like glucosinolates, phenylpropanoids, and flavonoids appears to be regulated by sugar-signaling pathways other than Tre6P, which may nevertheless make a small contribution (not depicted in this summary display). Links from sugar-signaling (largely Tre6P-independent) to cell wall modification and expansion growth, and links from Tre6P to light-signaling, the circadian clock and various hormone-signaling pathways are not depicted in this summary display.

Plants possess a plethora of sugar-signaling pathways including SnRK1, TORC, and S_1_ type bZIP TFs (Rolland et al. 2006; Lastdrager et al. 2014; Crepin and Rolland 2019; Baena-Gonzalez and Lunn 2020; Meng et al. 2022). We compared the iTPS response with published datasets to provide insights into interactions between Tre6P and these sugar-signaling pathways (summarized in Figs. 9 and 10).

It is well established that Tre6P inhibits SnRK1 activity in vitro, but open questions remain regarding the molecular mechanism and the general importance of this interaction (see Introduction). We used our inducible system to re-examine the relation between Tre6P- and SnRK1-signaling. We recently reported that induced elevation of Tre6P in the light period inhibits in vivo SnRK1 activity, measured as phosphorylation of a chimeric ACETYL COA CARBOXYLASE 1 peptide (Avidan et al. 2023). If Tre6P acts via inhibition of SnRK1, we would expect a reciprocal relationship between the transcriptional responses to induced overexpression of TPS and transient overexpression of SnRK1α1 (Baena-González et al. 2007). When we made this comparison for all ∼1,000 transcripts that showed a tSnRK1α1 response, there was only a weak negative correlation (*R*^2^ = 0.14 to 0.21) with many transcripts showing a similar rather than a reciprocal response (Fig. 6A). There was a strong negative correlation (*R*^2^ = 0.64 to 0.68) for the ∼500 transcripts that were probably responding directly to elevated Tre6P (i.e. the G_1_ component, Fig. 6B, Supplementary Fig. S14B). Morales-Herrera et al. (2023) recently reported that a small set of SnRK1 reporter transcripts showed the expected changes after applying small amounts of a membrane-permeable Tre6P analog to seedlings.

The reciprocal relationship between the response to elevated Tre6P and tSnRK1α1 was much stronger for tSnRK1α1-induced transcripts than tSnRK1α1-repressed transcripts (Supplementary Fig. S14, B and E). The former is mainly related to the C-starvation response, and the latter to biosynthesis and growth. The implication is that rising Tre6P inhibits the SnRK1 starvation response in a rather consistent manner, whereas the interaction is more complex for genes that are involved in biosynthesis and growth. Analyses of four sets of genes related to metabolism, growth and signaling (photosynthesis, light signaling, cytoplasmic ribosomal proteins, ribosome assembly factors) confirmed that, although the responses to elevated Tre6P and SnRK1α1 overexpression tended to be reciprocal, there were differences in the consistency of the relationship (Fig. 8C; Supplementary Fig. S14F). A strong correlation was found for ribosome assembly factors and ribosomal proteins (Fig. 8C; Supplementary Fig. S14H). The differentiated responses of SnRK1α1-repressed genes might indicate that the Tre6P and SnRK1 responses are partly independent. Alternatively, Tre6P might always act to inhibit SnRK1 but with further inputs may act downstream to modulate SnRK1 outputs (see also Avidan et al. 2023).

Tre6P also exerts transcriptional control over SnRK1 expression, decreasing expression of *SnRK1β1* relative to other regulatory subunits and the catalytic subunits (Supplementary Fig. S14A). This is reciprocal to the response to transient overexpression of SnRK1α1 (Baena-González et al. 2007, see also Supplementary Fig. S14J). These observations are consistent with Tre6P acting via SnRK1 to modify SnRK1 composition. In wild-type plants, *SnRK1β1* is induced under C-starvation (Usadel et al. 2008; Broeckx et al. 2016; Cookson et al. 2016; Peixoto and Baena-González 2022). Our analyses indicate this is due to a decline in Tre6P and activation of SnRK1 (Supplementary Fig. S24B).

TPS class II proteins were recently shown to bind SnRK1 and, in at least some cases, inhibit its activity (van Leene et al. 2022). Tre6P-repression of many TPS class IIs (see above) provides a potential loop to modify SnRK1 activity. FLZ family proteins are also emerging as important modulators of SnRK1-signaling (Nietzsch et al. 2016; Bortlik et al. 2022; Jamsheer et al. 2022). Analysis of their response to induced overexpression of TPS points to Tre6P-inhibition of SnRK1 regulating expression of a subset that is induced in C-replete conditions (Supplementary Fig. S16).

Overall, Tre6P signaling and SnRK1 function are closely intermeshed (Fig. 9), with Tre6P acting (i) to inhibit SnRK1 activity, with a broad impact on global transcript abundance, and (ii) transcriptionally to modify SnRK1 composition and the expression of two classes of proteins that interact with SnRK1. Thus, Tre6P not only modulates SnRK1 activity per se but may also adjust its functionality to the prevailing conditions. In addition, increased SnRK1 expression dampens the response of Tre6P to sucrose (Peixoto et al. 2021).

### Interaction with TORC signaling

TORC is a canonical positive regulator of ribosome biogenesis (Sabatini 2017; Ryabova et al. 2019; Wu et al. 2019; Meng et al. 2022). The most parsimonious explanation for the induction of ribosome biogenesis by Tre6P is that Tre6P acts via SnRK1 to regulate TORC activity. It has previously been observed that transient overexpression of SnRK1α1 leads to repression of many ribosomal proteins and ribosome assembly factors (Baena-González et al. (2007); see also Fig. 8C, Supplementary Figs. S14, H and I). Nukarinen et al. (2016) reported that SnRK1 phosphorylates the RAPTORB subunit of TOR and that loss of SnRK1α1 leads to increased phosphorylation of the canonical TORC target RPS6K.

We compared the transcriptional responses to elevated Tre6P and to TORC-inactivation (Supplementary text, Supplementary Figs. S15, F to J). There was no consistent relationship between the response to TORC inactivation and the direct response to elevated Tre6P (i.e. the G_1_ component of iTPS). This contrasts with the strong fingerprint of SnRK1 signaling in the direct response to elevated Tre6P and points to Tre6P acting by modifying SnRK1 signaling rather than TORC signaling. Further, whereas C-supply was a major input to Tre6P and SnRK1 signaling, the impact on TORC signaling was less obvious (Supplementary Fig. 15G). This is expected, as TORC is regulated by many other nutrient and hormonal signals (see Wu et al. 2019; Meng et al. 2022). Correspondingly, whilst part of the global transcriptional response to TORC resembles that of Tre6P and is reciprocal to that of SnRK1, part is unrelated or shows a reversed pattern. This observation indicates that Tre6P and SnRK1 tune part of the broad TORC output (see Supplementary text).

Elevated Tre6P modified expression of some known TORC phosphorylation targets (Supplementary Fig. S15, B to E, Supplementary text). This included weak induction of two LARP kinases, NAP1;1, RPS6 and some initiation and elongation factors. LARPs are involved in the TORC-LARP1-5´TOP signaling pathway that induces ribosomal proteins and assembly factors (Scarpin et al. 2020, 2022), whilst NAP1;1 and RPS6 promote rRNA transcription (Son et al. 2015). Their induction may contribute to the induction of ribosome biogenesis by elevated Tre6P. Notably, Tre6P repressed several members of the PYR/PYL family, which are phosphorylated and inhibited by TOR, pointing to concerted action of Tre6P and TORC to inhibit ABA sensing and signaling in C-replete conditions (Supplementary Fig. 15D, Supplementary text).

### Interaction with S_1_ bZIP signaling

S_1_-type bZIP proteins like bZIP11 dimerize with C-type bZIPs to orchestrate C-starvation responses, and this action is inhibited in C-replete conditions when sucrose translationally inhibits their synthesis (Wiese et al. 2004; Rahmani et al. 2009; Dröge-Laser and Weiste 2018). There was considerable overlap between the iTPS response and the published response to bZIP11 overexpression (Ma et al. 2011) (Supplementary Fig. 1^7^). This was partly indirect, possibly because the decrease in sucrose allows translation of bZIP11 protein. However, many bZIP11 targets were assigned to G_1_, which probably respond directly to elevated Tre6P. For most of the shared transcripts, the response to elevated Tre6P was opposite to the response to bZIP11 overexpression, and in many cases also opposite to the response to transient overexpression of SnRK1α1 (Supplementary Fig. S17E; see Supplementary text for genes in this subset). This dual layer of regulation presumably enhances responsiveness to low C. Another set of transcripts showed qualitatively similar responses to elevated Tre6P and bZIP11 overexpression (Supplementary Fig. S17E, Supplementary text). In this case, opposing regulation may stabilize expression or, alternatively, allow changes in expression in conditions where SnRK1 activity and sucrose levels change independently of each other.

### Tre6P as a component in an integrated network that processes internal and external information

In conclusion, inducible overexpression of TPS leads to widespread changes in transcript abundance, with significant changes for almost half the genome, and >2-fold changes for about 5,000 genes. Whilst about 40% are probably a direct response to elevated Tre6P, there is a high proportion of indirect responses. This mirrors the dual action of Tre6P on transcriptional regulation and on post-translational regulation, with the latter leading to changes in the levels of sucrose and other metabolites that trigger indirect transcriptional responses. Tre6P transcriptionally regulates important aspects of metabolism and growth, including repression of photosynthesis and enhancement of ribosome assembly and translation (Figs. 9 and 10) and interacts with other signaling pathways including the circadian clock, light- and ABA-signaling. Mechanistically, our global analysis provides strong support for the idea that a key function of Tre6P is to inhibit SnRK1 activity and prevent starvation responses when C availability is high. Tre6P acts via inhibition of SnRK1 to promote biosynthesis and growth, but in this case Tre6P also acts via additional pathways and/or the response is modulated by other factors. Furthermore, Tre6P modifies expression of regulatory subunits of SnRK1 and of proteins that interact with SnRK1. Over half the genes in Arabidopsis exhibit diel changes in transcript abundance, driven by changes in C-, light- and circadian-signaling (Bläsing et al. 2005; Usadel et al. 2008; Flis et al 2016). Perturbation of Tre6P, which is just one component of the C-signaling network, generates equally large perturbations. This highlights the sensitivity of signaling networks that plants use to integrate information about their internal metabolic status, the external conditions and the time-of-day, as well as the robustness provided by multiple connections within this network.

## Materials and methods

### Plant material, growth, and induction

Arabidopsis (*A. thaliana* (L.) Heyhn. accession Columbia-0) *iTPS lines 29.2* and *31.3* carrying the *p35S:alcR*/*pAlcA:otsA* construct for ethanol-induction of *Escherichia coli* TPS and the alcR empty vector control line (Caddick et al. 1998; Martins et al. 2013) were used in two experiments. For ATH1 microarray analysis, 29.2, 31.3 and alcR were grown as in Martins et al. (2013). For details see Supplementary text. Four-wk-old plants were sprayed to runoff with water (control) or 2% (v/v) ethanol at dawn or dusk and rosettes harvested 12 h later at ED or EN, respectively, by quenching in liquid nitrogen. Material was pooled from one or two pots (five or 10 rosettes), ground at −70°C using a robotized ball mill (Sulpice et al. 2014) and stored at −80°C. For RNAseq, 29.2 and AlcR were sown on a 1:1 mixture of soil (Stender AG, Schermbeck, Germany; https://www.stender.de) and vermiculite in 6-cm diameter pots, covered and stratified at 4°C in the dark for 48 h, germinated in a controlled environment chamber (Percival E-36 L chamber model AR66-cL2-cLED), CLF Plant Climatics GmbH, Weringen, Germany; (https://www.percival-scientific.com) with a 16 h photoperiod (white LEDs, 160 *µ*mol m^−2^ s^−1^), day/night temperature 21°C/19°C. Seedlings were transferred to 10-cm diameter pots (five per pot) and grown in the same conditions. At 22 DAS, plants were harvested immediately after dawn (untreated controls) or sprayed to run-off with water or 2% (v/v) ethanol 0.5 h after dawn and harvested 2, 4 and 6 h later. Four biological replicates (each four to five plants from one pot) were quenched in liquid nitrogen, ground and stored at −80°C.

### Metabolites

Metabolites were extracted with chloroform–methanol (Lunn et al. 2006). Tre6P, phosphorylated intermediates and organic acids were quantified by anion-exchange high-performance liquid chromatography coupled to tandem mass spectrometry (Lunn et al. 2006, modified as in Figueroa et al. 2016). Sugars were assayed enzymatically (Stitt et al. 1989). Starch was assayed enzymatically in the insoluble residue (Hendriks et al. 2003).

### RNA extraction

Total RNA was extracted with RNeasy Plant Mini-Kit (Qiagen, Hilden, Gemany; www.qiagen.com) according to manufacturer's protocol. RNA concentration and purity were estimated using a NanoDrop ND-1000 UV-VIS spectrophotometer (Thermo Fisher Scientific Inc. Waltham, MA., USA; www.thermofisher.com). RNA was diluted with water to 2 *µ*g/µL. RNA integrity was confirmed by agarose (1% w/v) gel electrophoresis and microfluidic electrophoresis (Bioanalyzer 2100, Agilent Technologies, Santa Clara, CA, USA: www.agilent.com). DNA was removed using Turbo DNA-free DNase I enzyme (Applied Biosystems, Waltham, MA, USA; www.thermofisher.com) according to the manufacturer's instructions. Absence of DNA contamination was confirmed by RT-qPCR using intron-spanning primers for the *MADS AFFECTING FLOWERING 5* (*MAF5*; At5g65080) gene: *MAF5*-For: 5´-TTTTTTGCCCCCTTCGAATC-3´; *MAF5*-Rev: 5´-ATCTTCCGCCACCACATTGTAC-3´.

### Global expression profiling

For ATH1 arrays (Affymetrix ATH1 GeneChip probe array), quality control and normalization were performed using Robin software (Lohse et al. 2012). The AlcR response was subtracted as follows. The difference in transcript abundance between water- and ethanol-treated plants was calculated separately for both iTPS lines, and plotted against differences in the AlcR empty vector control samples. A simple linear model was generated with the ethanol-minus-water-difference in the iTPS lines as response variable, and the difference in the AlcR as predictive variable. The coefficient relating the two variables was used to weight data from the iTPS lines. Differences after normalization and subtraction of the background ethanol effect were ascribed to induced TPS.

RNAseq analysis was performed on 2 *µ*g total RNA by BGI Genomics (Shenzhen, China; www.bgi.com) on quadruplicate replicates. Service included library construction, sequencing, quality control, and processing of raw sequencing data (including barcode trimming and removal of adaptor sequences, and low-quality reads), generating >20 million paired-end reads (100 bp) for each sample. Gene mapping and statistical analysis were performed in-house using the CLC Genomics Workbench software (QIAGEN Aarhus A/S, www.qiagenbioinformatics.com). Araport10 and Araport11 genome releases (https://www.arabidopsis.org; Cheng et al. 2017) were used for annotation. Expression values (RPKM; reads per kilobase of transcript per million reads mapped) were corrected library size. The CLC Genomics Workbench was used for differential expression analysis and FC calculations using the corrected RPKM values of ethanol- versus water-sprayed samples. FC is given on a transformed log_2_ scale; zero denotes no change and positive and negative values denote an increase and decrease (Robinson and Oshlack 2010). After applying an FDR < 0.05 and a FC ≥ 2 cutoff only a small fraction of genes exhibited significant change in the same direction in the iTPS and in the alcR control (see Results). These were highlighted in the DEG list and no further normalization was carried out (Supplementary Data Set S4). In data analyses in which a more relaxed filter (FDR < 0.05, FC ≥ 0.2) was applied to the iTPS data, more genes changed in the same direction in iTPS29.2 and alcR after ethanol induction. They were omitted from analyses.

### Statistical analysis

Technical replicates were averaged to generate a single value for each biological replicate. For changes in metabolite levels, statistical analysis was performed on biological replicates using Sigma-Plot 14.5 software (Systat Software GmbH, Düsseldorf, Germany; http://www.systat.de). Significance was tested by one-way ANOVA using a pairwise multiple comparison procedure, with post-hoc testing by Holm-Sidak (*P* < 0.05). To identify differential gene expression, the CLC genomics tool implements proportions-based tests (using RPKM values) as described in Baggerly et al. (2003). the test compares the proportions of counts between two groups (ethanol vs water spray) and assigns weights. The weights are obtained by assuming a Beta distribution on the proportions in a group, and estimating these, along with the proportion of a binomial distribution, by the method of moments. The result is a weighted *t*-type test statistic.

### PageMan, gene ontology, and STRING

PageMan analyses (Usadel et al. 2006) used MapMan software (Thimm et al. 2004; Usadel et al. 2006; version 3.6.0RC1; https://mapman.gabipd.org/) and mapping assignments of Ath_AGI_LOCUS_TAIR10_Aug2012. Heat maps show the average changes in all transcripts in a given BIN or sub-BIN. Only significant changes (FDR < 0.05, FC ≥ 0.2) were retained, other genes were assigned a zero value, before averaging all genes in the BIN or sub-BIN.

GO analyses were performed using the GO database (http://geneontology.org/), version PANTHER17.0 (release date 7th February 2021).

STRING (search tool for recurring instances of neighboring genes; Snel et al. 2000; Szklarczyk et al. 2021) analyses were performed at http://www.bork.embl-heidelberg.de/STRING.

### Accession numbers

Microarray data from this article have been deposited in Gene Expression Omnibus database (http://www.ncbi.nlm.nih.gov/geo) under accession number E-MTAB-12783. Accession numbers of genes and proteins are provided in Supplementary Figs. S8 to S10, S12 to S18, S20 and S23, and Supplementary Datasets S4 and S5.

## Supplementary Material

kiae196_Supplementary_Data

## Supplementary data

The following materials are available in the online version of this article.

**Supplementary Figure S1.** Comparison of changes in transcript abundance 12 h after induction of TPS in two independent iTPS lines, 29.2 and 31.3.

**Supplementary Figure S2.** Estimation of the CRF.

**Supplementary Figure S3.** Comparison of the CRF and iTPS in the ED and EN treatments.

**Supplementary Figure S4.** Relation between Tre6P and sucrose, and changes of further metabolites after induction in iTPS29.2 for 4 or 6 h in the light (supplemental to Fig. 1).

**Supplementary Figure S5.** Global analysis of the response of transcript abundance at 4 and 6 h after induction in *iTPS29.1* (supplemental to Fig. 2).

**Supplementary Figure S6.** Changes in expression of selected G_0_ genes in response to various treatments by which CRF was determined, and abundance of transcripts of genes assigned to G_0_.

**Supplementary Figure S7.** Visualization of selected responses in metabolism using PageMan displays (supplemental to Fig. 3).

**Supplementary Figure S8.** Responses of transcripts encoding proteins involved in sucrose transport and metabolism.

**Supplementary Figure S9.** Responses of selected transcripts encoding proteins involved in the regulation of floral induction.

**Supplementary Figure S10.** Responses of transcripts encoding proteins involved in the circadian clock.

**Supplementary Figure S11.** Enrichment analyses using GO.

**Supplementary Figure S12.** Comparison of the changes in transcript abundance after induced increase in Tre6P, and in Arabidopsis lines that constitutively express bacterial TPS (Supplementary to Fig. 6 and Supplementary Table S5).

**Supplementary Figure S13.** Responses of transcripts encoding proteins involved in Tre6P metabolism.

**Supplementary Figure S14.** Responses of transcripts encoding SnRK1 subunits, or implicated as downstream transcriptional targets of SnRK1 signaling.

**Supplementary Figure S15.** TORC subunits, higher level regulators of ribosome biogenesis, other TORC targets including ABA receptors, and changes in global transcript abundance after inhibiting TORC.

**Supplementary Figure S16.** FCS-LIKE ZINC FINGER (FLZ) family proteins.

**Supplementary Figure S17.** Comparison of iTPS with constitutive overexpression of bZIP11.

**Supplementary Figure S18.** TFs that respond to an induced increase in Tre6P levels.

**Supplementary Figure S19.** Analysis of TFs assigned to CRF group G_1_.

**Supplementary Figure S20.** C and S_1_ bZIP transcription factors.

**Supplementary Figure S21.** Transcript for TFs that were assigned to CRF group G_1_ in the iTPS response: comparison of their iTPS response and in their oeAKIN10 response.

**Supplementary Figure S22.** Analysis of TFs assigned to CRF group G_2_.

**Supplementary Figure S23.** Glucosinolate metabolism and its transcriptional regulation.

**Supplementary Figure S24.** Proposed interplay between Tre6P-mediated signaling and other C-signaling pathways in wild-type plants (supplemental to Figs. 5 and 7).

**Supplementary Table S1.** List of transcriptome datasets used in this study.

**Supplementary Table S2.** Changes in transcript abundance 12 h after induction of TPS, investigated at ED or EN (extends Supplementary Figs. S1 and S3).

**Supplementary Table S3.** Summary of the number of DEGs, their assignment to CRF groups and the R2, direction and *P*-value of the relationship between the change in transcript abundance and the CRF value (extends Fig. 2 and Supplementary Fig. S5, A and B).

**Supplementary Table S4.** Pairwise comparison of responses after 4 and 6 h of induction in the light, and in the ED and EN treatments (extends Supplementary Fig. S5C).

**Supplementary Table S5.** Comparison of the responses to constitutive overexpression of TPS (oeTPS) and induced overexpression of TPS (iTPS) (extends Fig. 6 and Supplementary Fig. S12).

**Supplementary Table S6.** Comparison of the tSnRK1α1 and iTPS responses (extends Fig. 8 and Supplementary Fig. S14).

**Supplementary Data Set 1.** ATH1 profiling of ED and EN treatments (underlies Supplementary Fig. S1 and Supplementary Tables S2 and S4).

**Supplementary Data Set 2.** Calculation of CRF (underlies Supplementary Fig. S2).

**Supplementary Data Set 3.** Metabolites iTPS 4 to 6 h (underlies Fig. 1 and Supplementary Fig. S3).

**Supplementary Data Set 4.** RNAseq data (underlies Figs. 2 to 8, Supplementary Figs. S5 to S14, and Supplementary Tables S2 to S6).

**Supplementary Data Set 5.** GO analysis of RNAseq dataset (extends Supplementary Fig. S11).

**Supplementary Data Set 6.** Robust set of Tre6P-regulated genes (underlies Fig. 6, Supplementary Fig. S12 and Supplementary Table S5).

**Supplementary Data Set 7.** GO analysis (underlies Fig. 6, Supplementary Fig. S12 and Supplementary Table S6).

**Supplementary Data Set 8.** Transcription factors (underlies Supplementary Figs. S18 to S24).

## Dive Curated Terms

The following phenotypic, genotypic, and functional terms are of significance to the work described in this paper:

ATH1 Gramene: AT4G32980
ATH1 Araport: AT4G32980

RPS6 Gramene: AT4G31700
RPS6 Araport: AT4G31700

clf Gramene: AT2G23380
clf Araport: AT2G23380

AtTPS1 Gramene: AT1G78580
AtTPS1 Araport: AT1G78580

MAF5 Gramene: AT5G65080
MAF5 Araport: AT5G65080

bZIP25 Gramene: AT3G54620
bZIP25 Araport: AT3G54620

BZIP9 Gramene: AT5G24800
BZIP9 Araport: AT5G24800

TPS1 Gramene: At1g78580
TPS1 Araport: At1g78580

PGM Gramene: AT1G78050
PGM Araport: AT1G78050

TPS10 Gramene: Medtr5g073200
TPS10 Araport: Medtr5g073200

bZIP11 Gramene: AT4G34590
bZIP11 Araport: AT4G34590
