# Supplementary material for: Direct and indirect responses of the Arabidopsis transcriptome to an induced increase in trehalose 6-phosphate
Source: Plant Physiol. 2024 Apr 9;196(1):409–31. doi: 10.1093/plphys/kiae196 (PMC11376379; doi:10.1093/plphys/kiae196)
Supplement: kiae196_Supplementary_Data [file kiae196_supplementary_data.zip › SUPP Figs and Tables editted.pdf]

SUPPLEMENTAL FIGURES AND TABLES

Avidan et al., Direct and indirect responses of the Arabidopsis transcriptome to an induced increase in trehalose 6-phosphate

**Supplemental Figures**	**Page**
Supplemental Figure S1	2
Supplemental Figure S2	3
Supplemental Figure S3	4
Supplemental Figure S4	6
Supplemental Figure S5	8
Supplemental Figure S6	10
Supplemental Figure S7	13
Supplemental Figure S8	24
Supplemental Figure S9	25
Supplemental Figure S10	26
Supplemental Figure S11	27
Supplemental Figure S12	28
Supplemental Figure S13	33
Supplemental Figure S14	33
Supplemental Figure S15	37
Supplemental Figure S16	42
Supplemental Figure S17	43
Supplemental Figure S18	48
Supplemental Figure S19	53
Supplemental Figure S20	55
Supplemental Figure S21	56
Supplemental Figure S22	57
Supplemental Figure S23	59
Supplemental Figure S24	60

Supplemental Tables

Supplemental Table S1	61
Supplemental Table S2	62
Supplemental Table S3	63
Supplemental Table S4	64
Supplemental Table S5	65
Supplemental Table S6	66

Supplemental Figure S1. Comparison of changes in transcript abundance 12 h after induction of TPS in two independent iTPS lines, 29.2 and 31.3.

iTPS lines 29.2 and 31.3 and the alcR control were grown in an equinoctial (12 h light/12 h dark) light regime at 160 µmol quanta m^{-2}-s^{-1} for 28 days, and then either sprayed with 2% ethanol or water at dawn and harvested 12 h later at dusk (ED treatment), or sprayed with 2% ethanol or water at dusk and harvested 12 h later at dawn (EN treatment). Transcriptome analysis was performed using ATH1 arrays and data are supplied in Supplemental Dataset S1. The change in transcript abundance after ethanol treatment of an iTPS line was calculated compared to the water-sprayed iTPS line. There were only minor changes in transcript abundance due to ethanol addition to alcR controls and this was corrected as explained in Materials and Methods. The results are the mean of 3 samples (each sample consisted of 5-10 whole rosettes). All values are plotted on a transformed $\log_2$ scale (see Methods)

(A) Venn diagrams. The numbers represent transcripts that passed the filter. specified in the display (FDR, False Rate Discovery; FC, fold change)..

(B) Reproducibility of response between *iTPS29.2* and *iTPS31.3* at dusk after inducing at dawn; scatter plots are provided for all transcripts and after filtering (FDR < 0.05).

(C) Reproducibility of response between *iTPS29.2* and i*TPS31.3* at dawn after inducing at dusk.

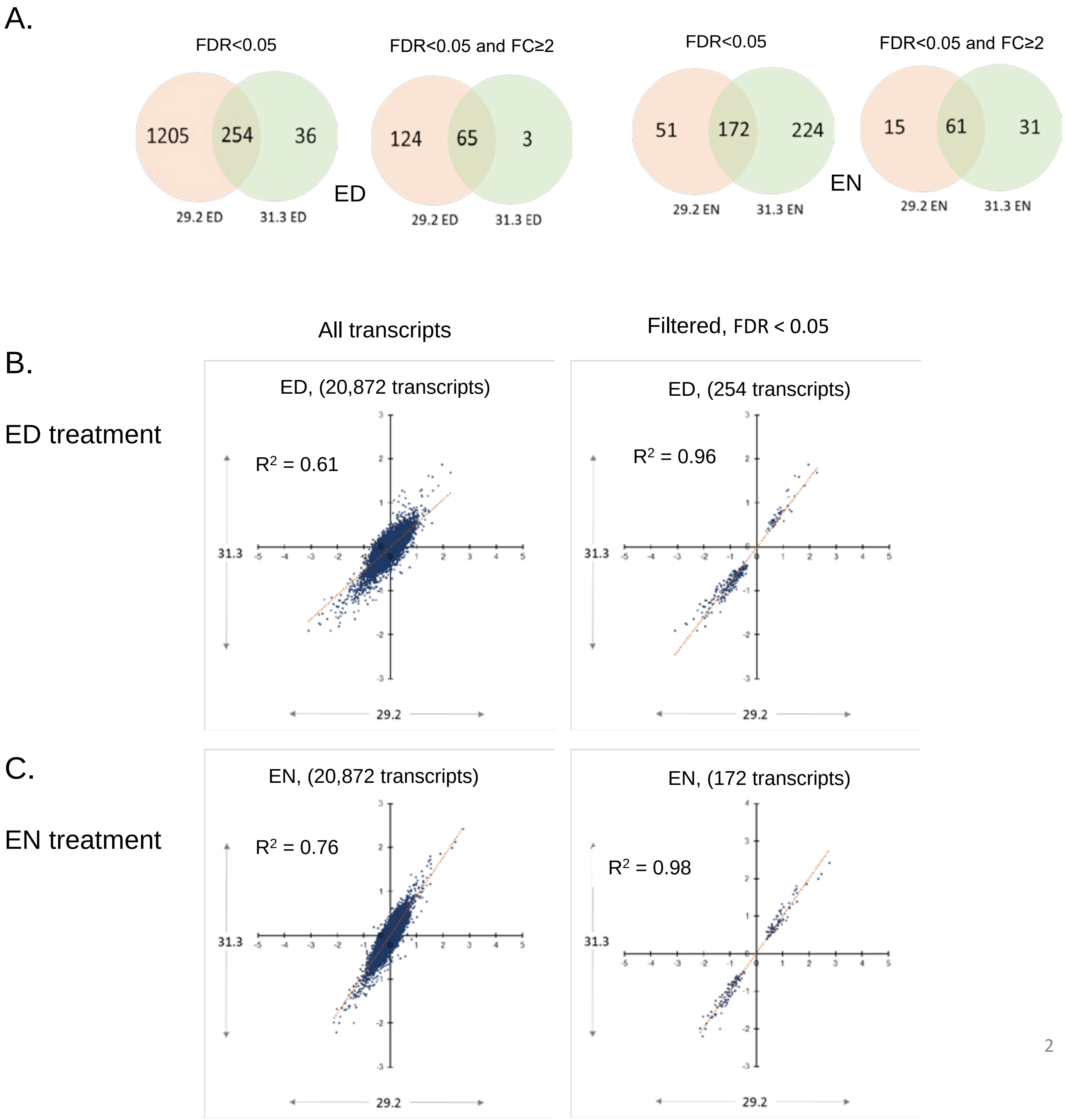

Supplemental Figure S2. **Estimation of the carbon response factor, CRF**. The carbon response factor (CRF) summarizes the response to an increase in sugars across a set of nine treatments. A positive and negative CRF value represents an increase or decrease in transcript abundance in response to elevated sugar, respectively. The CRF was calculated using the response of a given for each transcript in nine treatments that included four treatments in which sugars were supplied to C-starved seedlings, and five treatments that changed endogenous sucrose and reducing sugar levels in rosettes of plants growing in an equinoctial light-dark cycle at 160 µmol quanta m^{-2}-s^{-1}, similar to the conditions used for the iTPS experiments. Especially in diel cycles, transcript abundance is strongly influenced by circadian clock- or light-signaling (Bläsing et al., 2005; Usadel et al., 2008). The treatments were chosen to minimize the impact of circadian clock- or light signaling in transcript abundance.

A B

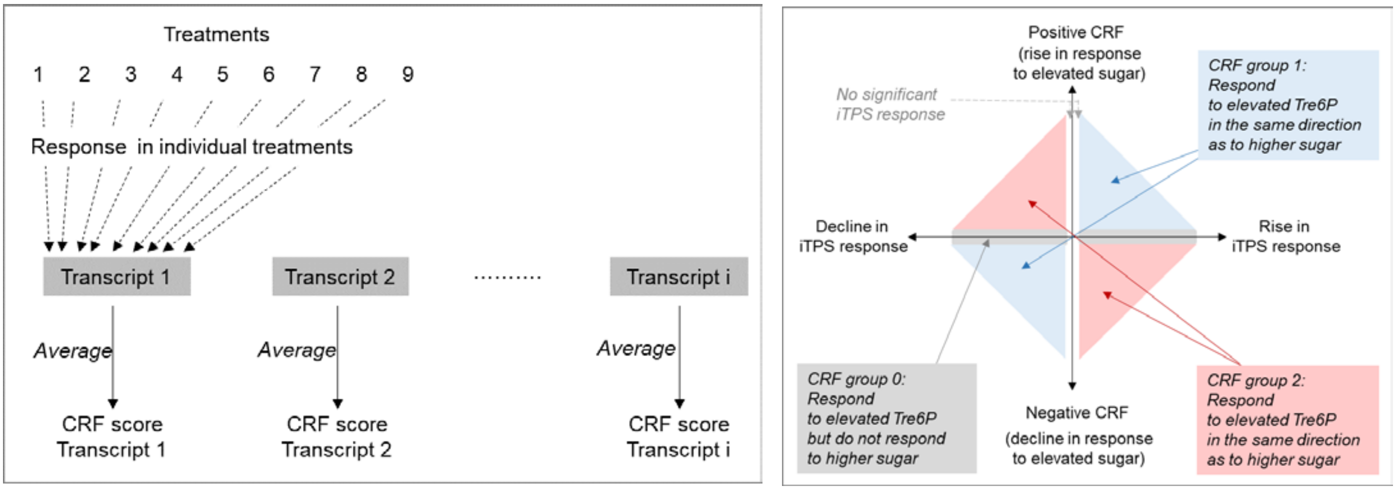

(A) Illustration of the calculation: (1-4) rosettes of *pgm* mutant plants grown at equinoctial light regime compared to wild-type Col-0 at ZT8, ZT12 (ED), ZT20 and ZT24 (EN), respectively (Gibon et al., 2004; Blasing et al., 2005); (5-8) whole seedlings in liquid culture grown under low (50 µmol quanta m^{-2}-s^{-1}) continuous light: (5) full nutrition compared 2 days C-starved seedlings (Osuna et al., 2007); (6) 30 min after adding 15mM sucrose to C-starved seedlings compared to full nutrition (Osuna et al., 2007); (7) 3-h after adding 15mM sucrose to C-starved seedlings compared to full nutrition (Osuna et al., 2007); (8) 30 min after adding 100 mM glucose to C-starved seedlings compared to full nutrition (Blasing et al., 2005); (9) Rosettes of vegetatively growing plants illuminated for 4h after dawn at 350 compared to 50 ppm CO_2 (Blasing et al., 2005). Log_2 FC values (filtered as greater or smaller than $+log_2 0.1$ or $-log_2 0.1$ on the transformed log_2 scale on which the expression value were given, see Methods, respectively) were averaged across the nine treatments taking into account the treatment effect on C status, hence treatments 3-4 were assigned a reverse sign as they led to a decrease in C. The low filter was used to minimize the number of genes that were classified as C-unresponsive (see Supplemental Figure S3A-B and Methods for further details). FC is fold change.

B) Use of the CRF in combination with the observed change in transcript abundance in iTPS (the response of a transcript to induced expression of bacterial TPS) to assign genes to groups. Group 1 (G_1) denotes transcripts where the iTPS response and CRF are qualitatively the same and, by inference, the iTPS response may be a direct response to elevated Tre6P, group 2 (G_2) denotes transcripts where the iTPS response and CRF are qualitatively opposed and by inference the iTPS response is unlikely to be a direct response to elevated Tre6P but is likely to be a independent response to lower sucrose or related metabolites, and group 0 (G_0) denotes transcripts that respond in the iTPS response but cannot be scored because they do not show a consistent response to changes in sugars.

Supplemental Figure S3. Comparison of the carbon response factor (CRF) and iTPS at dusk and dawn. The analysis was performed with the transcript abundance dataset for line 29.2 from Supplemental Figure S1. In the ED treatment, TPS was induced at dawn and plants were harvested at the end of the light period, and in the EN treatment TPS was induced at dusk and plants were harvested at the end of the night.

The Carbon response factor (CRF) summarizes the response of a given transcript to a change in sugars across a set of treatments, with an increasingly positive sign indicating the average increase in abundance and an increasingly negative sign denoting the average decrease in abundance, whilst a value around zero indicates that transcript abundance does not respond to sugar status (for derivation see Supplemental Figure S2, Supplemental Dataset S2 and main text). iTPS denotes the response to induced expression of bacterial TPS.

(A) Total iTPS.29.2 response in the ED treatment ($\log_2$ FC) compared to the CRF.

(B) Total iTPS29.2 response in the EN treatment (($\log_2$ FC) compared to the CRF.

(C) Deconvolution of the iTPS response in the ED treatment, based on the CRF assigned to each transcript. Briefly, transcripts assigned to group 1 (G_1) respond to elevation of Tre6P in the same direction as their response to an increase of sugar supply in a panel of treatments (as defined by the CRF); transcripts assigned to group 2 (G_2) respond to elevation of Tre6P in the opposite direction to their response to an increase of sugar supply in the panel of treatments; transcripts assigned to group 0 (G_0) do not show a notable response to a change in sugar (average response cut off: Log_2 FC ≥ 0.1). For further explanation of the treatments and procedure used to estimate the CF CRF values for each transcript, see Supplemental Figure S2

(D) Deconvolution of the iTPS response in the EN treatment (same procedure as for panel C).

(E) Comparison of the shared deconvoluted responses in the ED and EN iTPS responses at dusk and dawn. Genes were assigned to CRF groups G_1, G_2 and G_0 as described above and in Supplemental Figure S2. Genes assigned to the same CRF group at ED and EN were then regressed against each other.

In all panels, the number in brackets gives the number of transcripts in the analyzed subset.

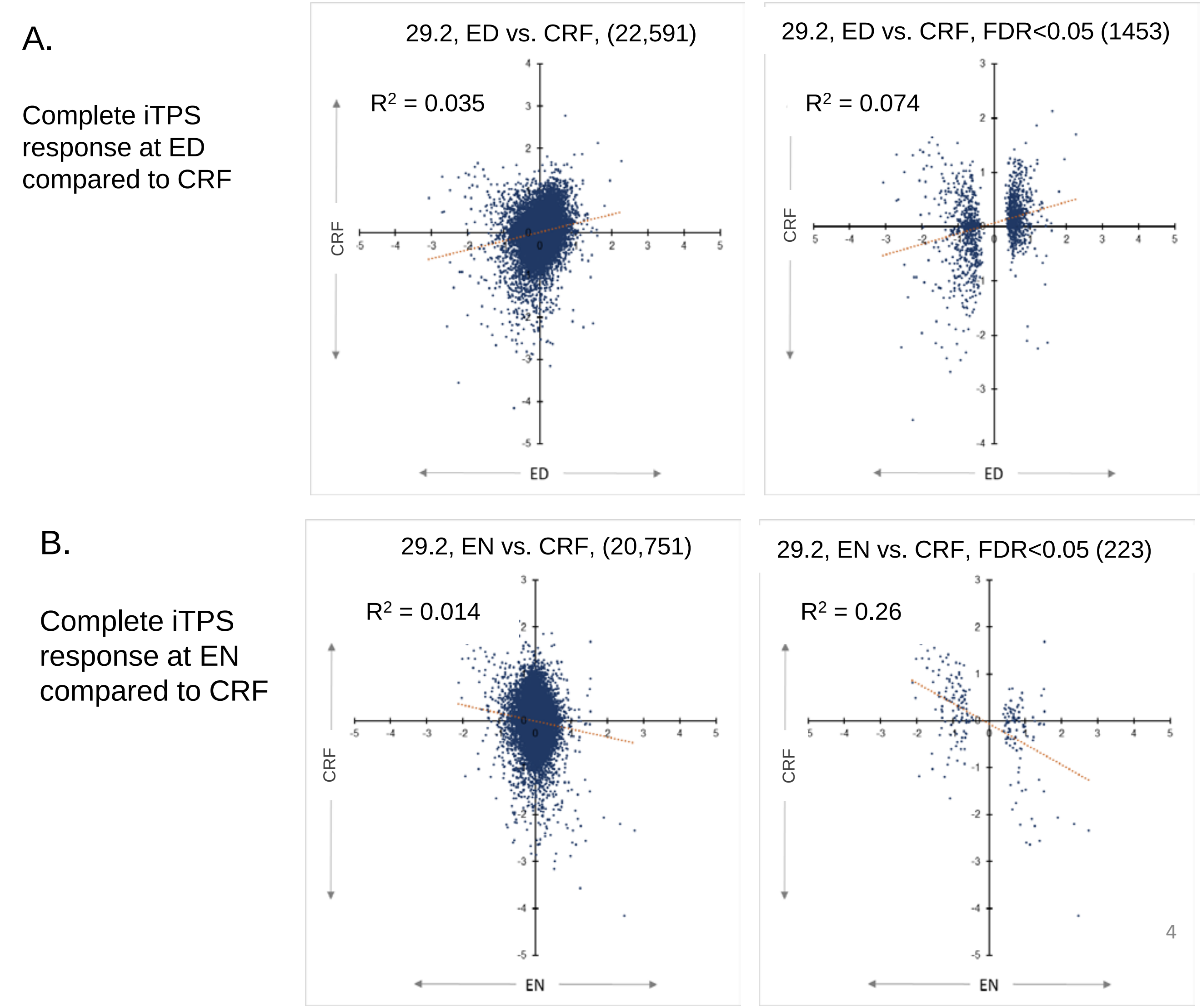

Figure S3 (continued)

C. iTPS response at ED compared to CRF after separation into G_1, G_2, G_0

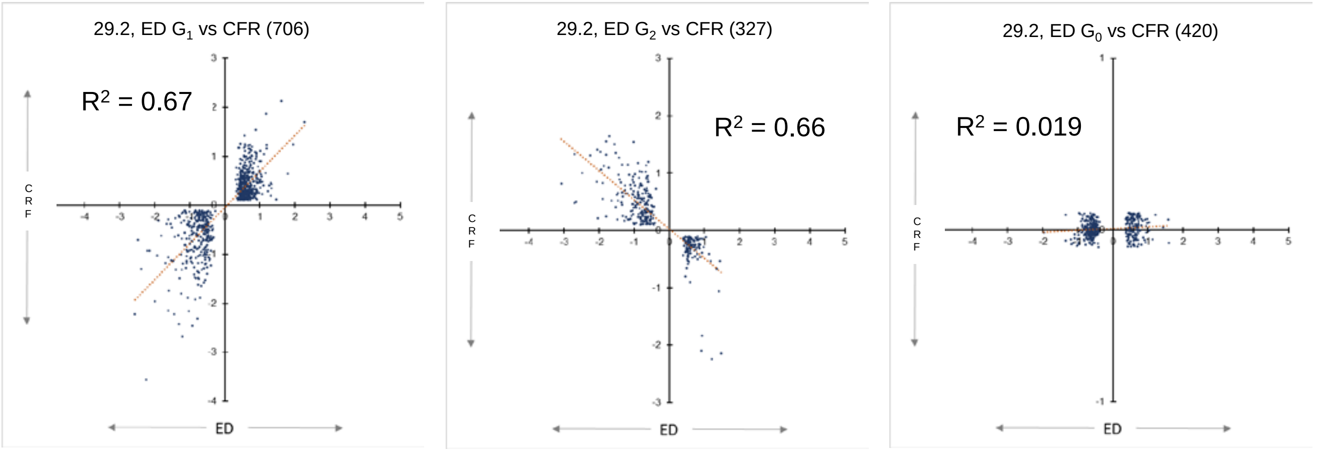

D. iTPS response at EN compared to CRF after separation into G_1, G_2, G_0

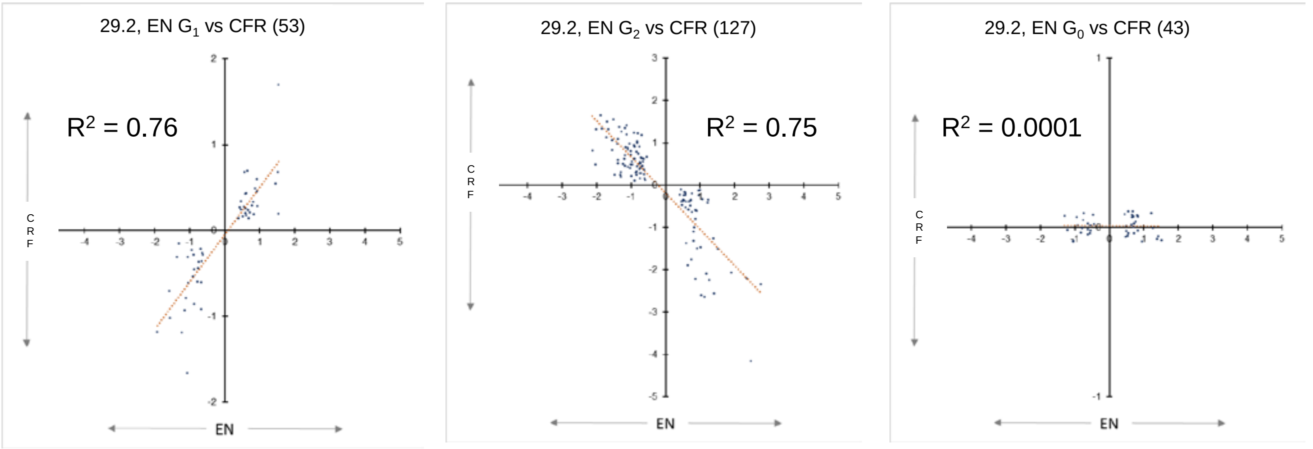

E. Comparison of iTPS responses at ED and EN

Genes assigned to G_1 | Genes assigned to G_2 | Genes assigned to G_0

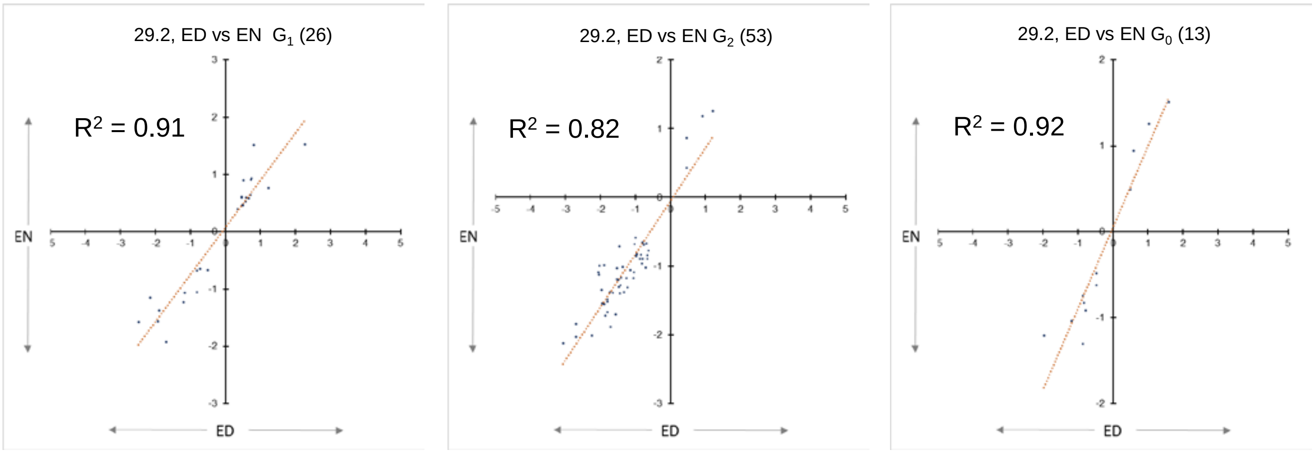

Supplemental Figure S4. Changes of further metabolites after induction in iTPS29.2 for 4 h or 6 h in the light (supplemental to Figure 1). The data are for the experiment shown in Figure 1 and used for RNAseq analyses. The data are provided in Supplemental Dataset S3.

(A) Bacterial TPS protein (otsA) detected by western blots of water controls and ethanol-sprayed (0, 2, 4 and 6-h post-spraying) line *iTPS 29.2*. The right-hand lane shows a wildtype control. The induction of otsA is visible as the gradual increase in intensity of a band at about 57kDa. The anti-otsA antibody cross-reacts weakly with an endogenous protein with a molecular weight just under 57kDa, leading to a weak band in non-induced iTPS and in wild-type controls.

(B) Regression plot of Tre6P against sucrose. The expected positive relationship is seen for the three control treatments (water and ethanol-sprayed alcR, water-sprayed iTPS 29.2. In ethanol-sprayed iTPS29.1, compared to controls and pre-spray ITPS 29.2, there is no marked change in the relationship between Tre6P and sucrose at 2-h post-spraying, and a negative relationship at 4-h and 6-h post-spraying. FW = fresh weight., iTPS is the response to induced expression of TPS. FW is fresh weight.

(C) Further metabolites (see next page)

A

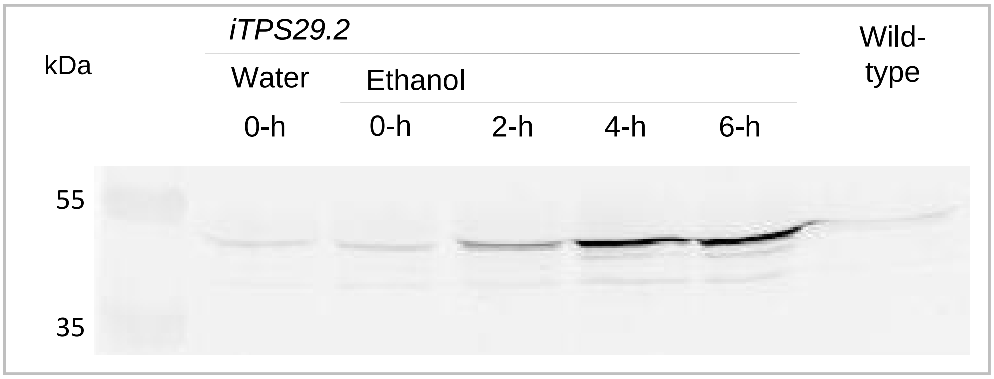

B

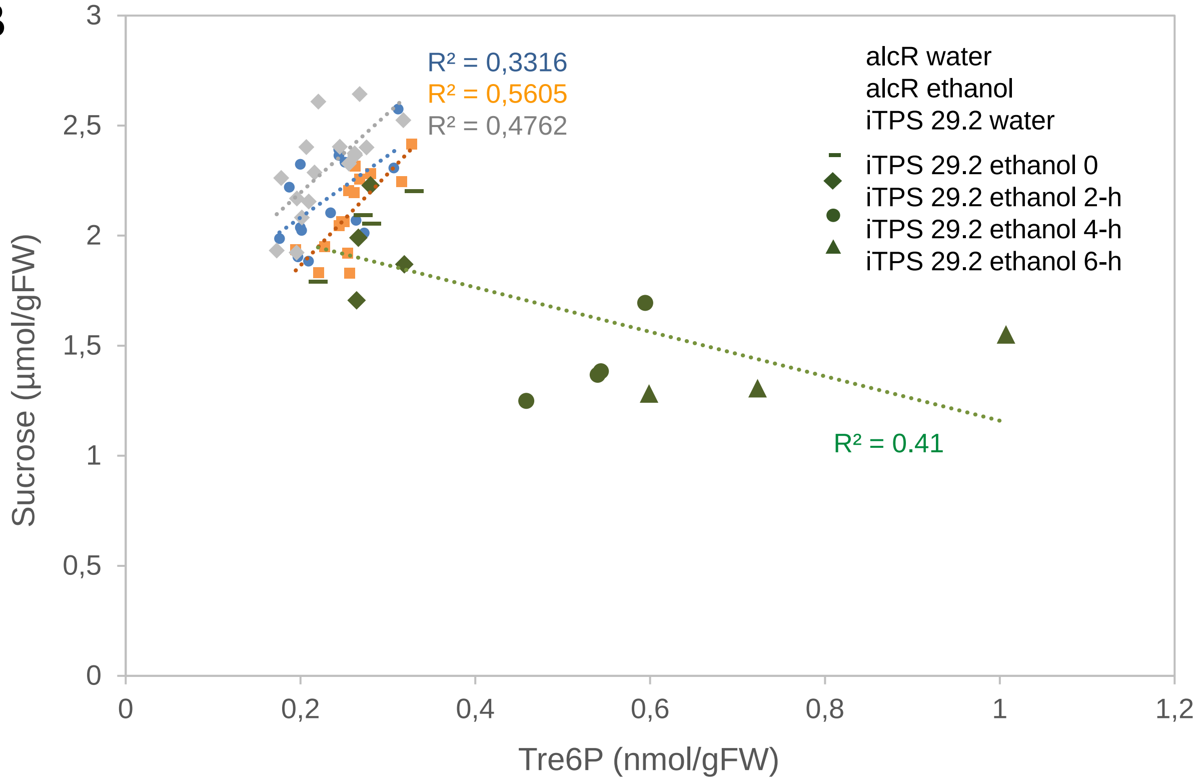

Supplemental Figure S4. continued.

(C) Further metabolites Glc6P, Fru6P, mannose 6-P, PEP, pyruvate, shikimate, malate, fumarate, citrate, aconitate, 2-oxoglutarate. The results are plotted as mean ± S.D. (n = 4) (each replicate contained 4-5 whole rosettes). Significant changes of ethanol-sprayed iTPS 29.2 compared to the three control treatments (water and ethanol-sprayed alcR, water-sprayed iTPS 29.2) are indicated by asterisks (one-way ANOVA, Holm-Sidak, with *, **, *** correspond to P < 0.05, < 0.01, < 0.001). The original data are provided in SI Dataset S3. FW is fresh weight.

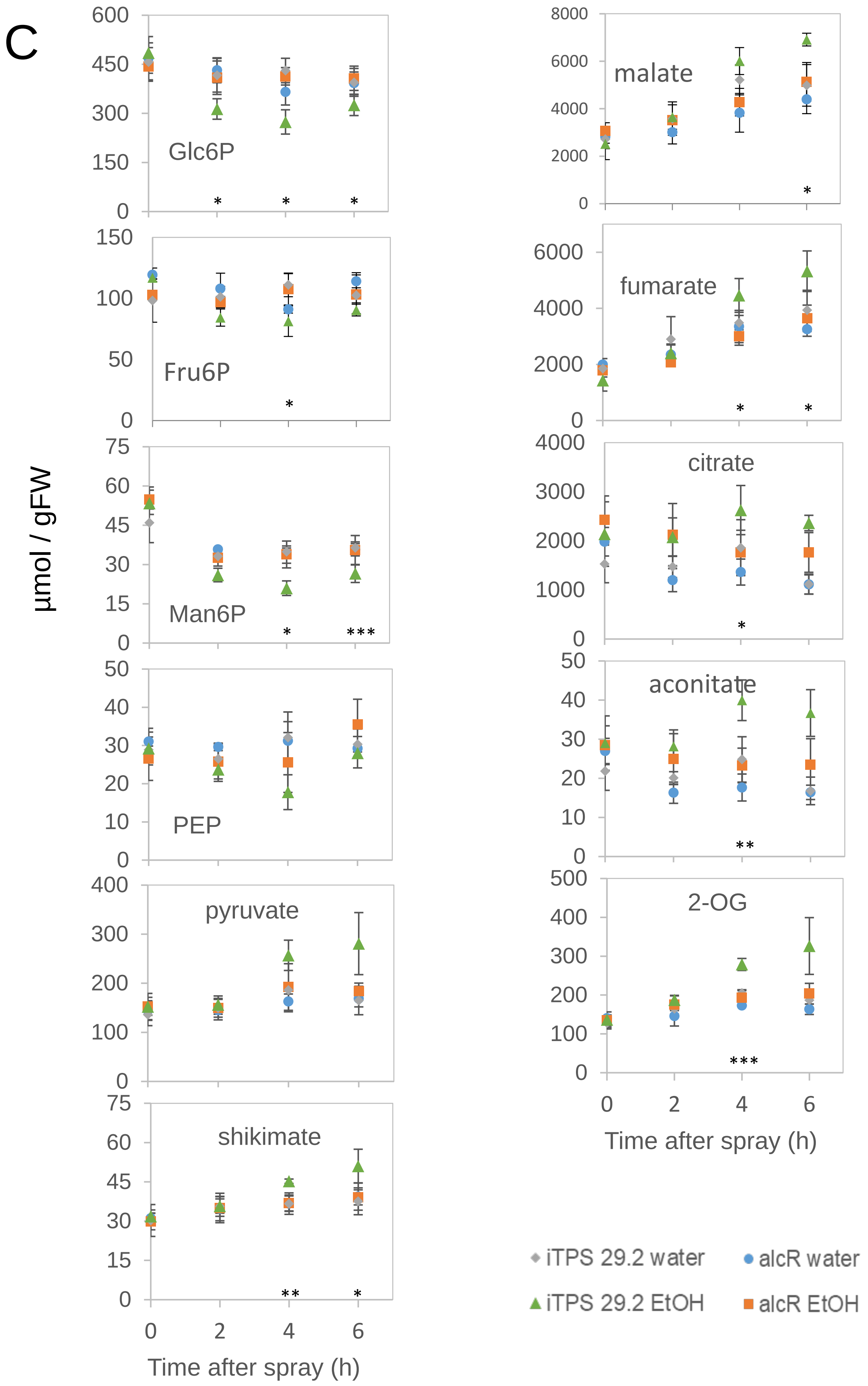

Supplemental Figure S5. Global analysis of the response of transcript abundance at 4 h and 6 h after induction in *iTPS29.1* (supplemental to Figure 2).

(A) Comparison of the response at 4 h and 6h; plots are shown for all transcripts, for all transcript whose responses was significant at FDR <0.05, and for all transcripts whose response was significant at FDR<0.05 and a >2-fold change. iTPS denotes the response after induction of TPS.

(B) Plots of the response against the CRF; plots are shown separately for the response at 4 h (Figure 2D) and at 6h (this panel) for which plots are shown from left to right for all transcripts, for all transcript whose responses was significant at FDR <0.05, and for all transcripts whose response was significant at FDR<0.05 and a >2-fold change. For derivation of the CRF see Supplemental Figure S2.

(C) Reproducibility of the response 4-h or 6-h post-induction in the light compared with to that in the ED and EN treatments (see Supplemental Figure S1). Note that transcript abundance was assessed using different technologies for the 4h and 6h treatments (RNA seq) and the ED and EN treatments (ATH1) with the latter being technically less sensitive for low abundance genes (next page)

In all panels, the number in brackets gives the number of transcripts in the analyzed subset. All values are plotted on a transformed $\log_2$ scale (see Methods)

A

Overall iTPS response versus CRF

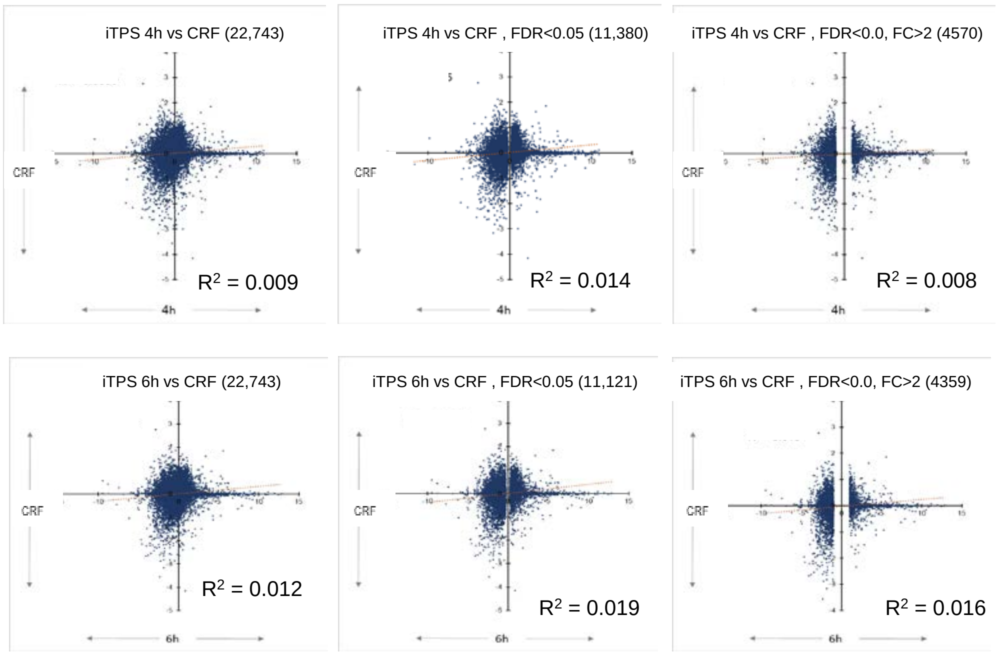

B

iTPS response at 6-h after assignment to G_1, G_2 and G_0 versus CRF

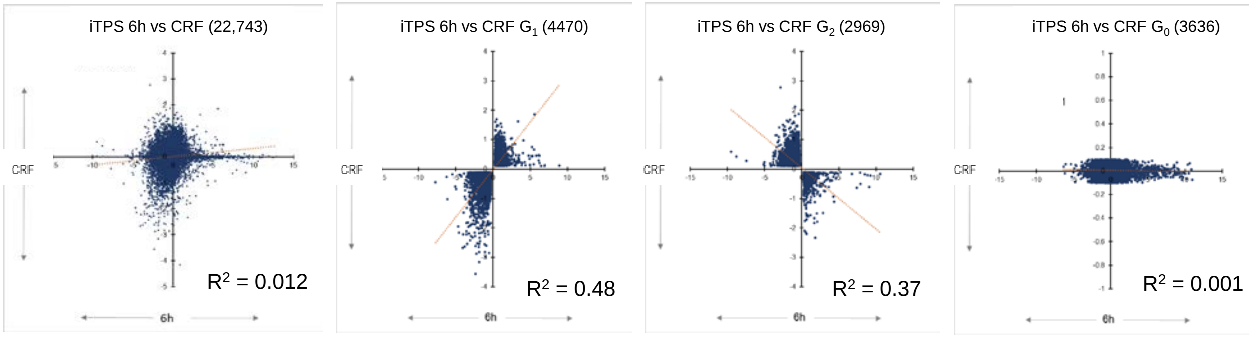

Panel C is on the next page

Figure S5 (continued)

(C) Reproducibility of the response 4-h or 6-h post-induction in the light (Figure 2) compared with to that in the ED and EN treatments (see Supplemental Figure S1). Note that transcript abundance was assessed using different technologies for the 4h and 6h treatments (RNA seq) and the ED and EN treatments (ATH1) with the latter being technically less sensitive for low abundance genes

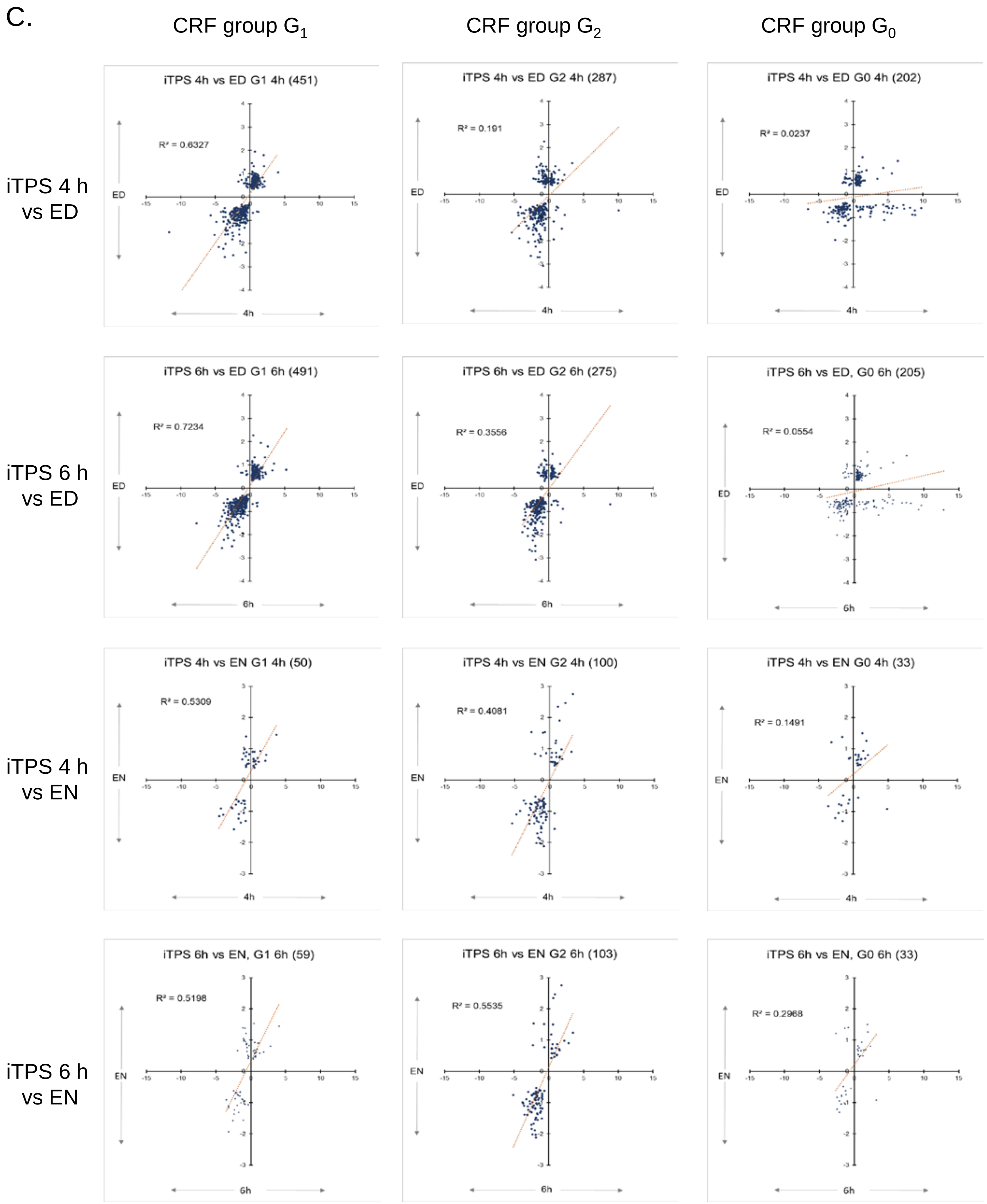

Supplemental Figure S6. changes in expression of top 10-100 G0 genes in response to various treatments, by which CRF was determined.

(A) Response of transcript abundance in each of the nine treatments. The plots show, for genes assigned to G_0, the response of transcript abundance for the 100 and 10 genes whose transcripts showed the largest increase in the iTPS response, and the 100 and 10 genes whose transcripts showed the largest decrease in the iTPS response. The analysis used the shared response between the iTPS 4h and 6h treatments. FC is fold change; iTPS is the response to induced expression of TPS.

(B) Top ten genes in the iTPS G_0 group UP and the iTPS G_O group DOWN and their response in the nine treatments used to estimate the CRF. (see next page)

(C) Impact of transcript abundance on assignment to CRF group G_0. (see over-nest page)

The treatments used were (see also Supplemental Figure S2):

- p8-d8, p12-d12, d20-p20, d24-p24: pgm mutant grown at equinoctial light regime compared to Col-0 at ZT8, ZT12 (ED), ZT20 and ZT24 (EN) (Gibon et al., 2004; Blasing et al., 2005);
- FN-St: full nutrition compared 2 days C-starved seedlings (Osuna et al., 2007);
- 30mSUC: 30 min after adding 15mM sucrose to C-starved seedlings compared to full nutrition (Osuna et al., 2007);
- 3hSUC: 3-h after adding 15mM sucrose to C-starved seedlings compared to full nutrition (Osuna et al., 2007);
- 30mGlc: 30 min after adding 100 mM glucose to C-starved seedlings compared to full nutrition (Blasing et al., 2005);
- 350-50CO_2: rosettes illuminated for 4 h at 350 compared to 50 ppm (Blasing et al., 2005).

A

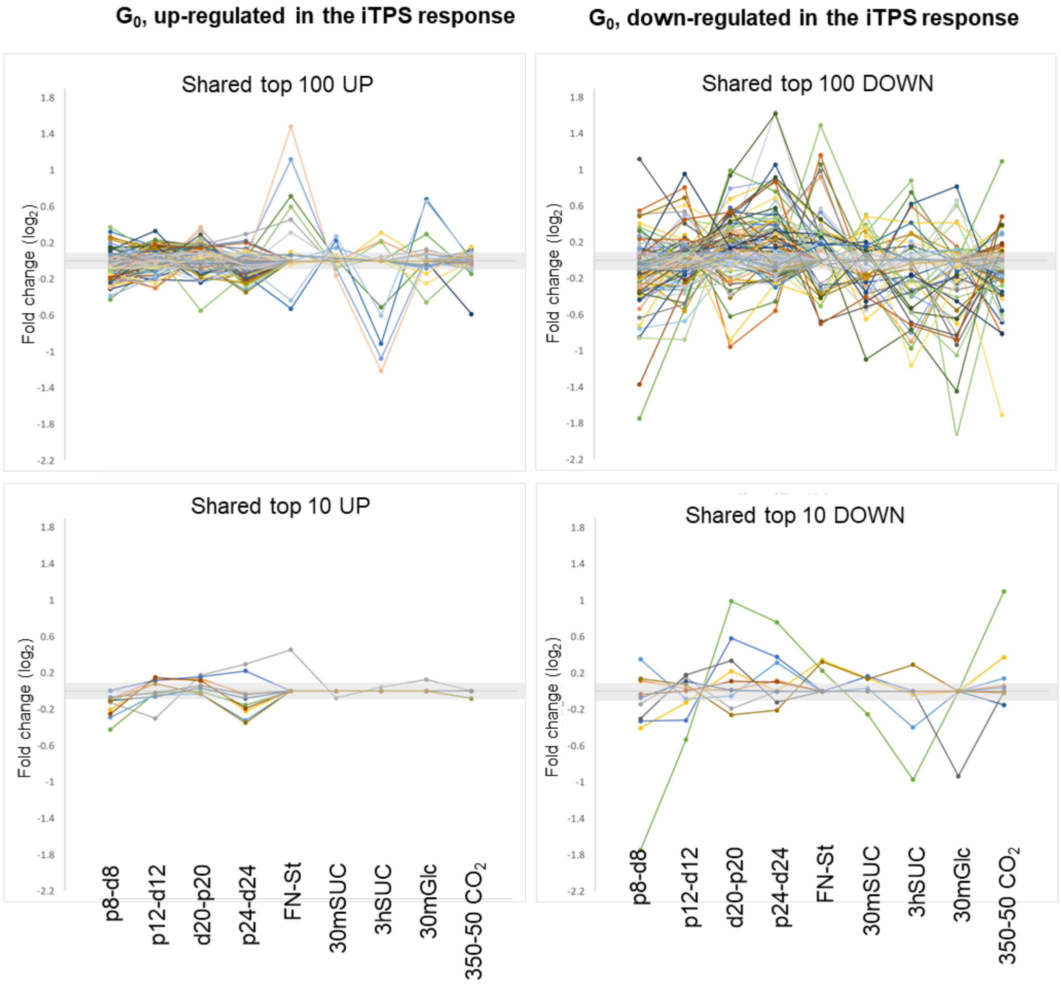

Figure S6: changes in expression of selected G_0 genes in response to various treatments by which the CRF)was determined and abundance of transcripts of genes assigned to G_0 (continued)

(B) The 10 top genes In the iTPS G_0 group UP and the iTPS G_O group DOWN and their response in the nine treatments used to estimate the CRF. The analysis shows the responses in the nine treatments used to estimate the CRF (columns 1-9 from the left) and the 4h and 6h iTPS responses (the 2 right-hand columns). The transcripts are ordered based on the response at 4 h.

B

top 10 SHARED UP in CRF group G_0 (iTPS 4-h and 6-h)

	ID / treatment	p8-d8	p12-d12	p20-d20	p24-d24	FN - St	30mSUC - St	3hSUC - St	30mGlc -St	350 - 50 CO_2	Log_2FC iTPS 4h	Log_2FC iTPS 6h
Glycosyl hydrolase family protein	At3g62710	-0.29	-0.04	-0.03	-0.32	0.00	0.00	0.00	0.00	0.00	10.97	8.07
reverse transcriptase-like protein	At5g19270	-0.09	0.12	0.14	-0.03	0.00	0.00	0.00	0.00	0.00	10.85	12.85
Chloroplast localized GFDP synthase	At3g14530	-0.12	-0.30	0.18	0.30	0.46	-0.08	0.04	0.13	0.00	10.60	9.51
SKU5 similar 11	At3g13390	-0.20	0.13	0.13	-0.22	0.00	0.00	0.00	0.00	0.00	10.52	9.30
member of the ERF (ethylene response factor) subfamily B-6 of ERF/AP2 transcription factor family.	At5g67010	0.01	0.12	0.16	0.22	0.00	0.00	0.00	0.00	0.00	10.38	8.11
hypothetical protein	At5g62330	-0.42	-0.02	0.00	-0.15	0.00	0.00	0.00	0.00	0.00	10.16	9.45
Late embryogenesis abundant (LEA) protein	At4g36600	-0.10	-0.06	0.04	-0.08	0.00	0.00	0.00	0.00	0.00	10.00	4.89
Encodes a defensin-like (DEFL) family protein	At3g63360	-0.25	0.15	0.12	-0.19	0.00	0.00	0.00	0.00	0.00	9.86	11.10
Cytidine/deoxycytidylate deaminase family protein	At4g29620	-0.07	-0.02	0.06	-0.04	0.00	0.00	0.00	0.00	0.00	9.80	6.89
Member of CYP76C family of cytochrome P450 enzymes.Has geraniol 9- or 8-hydroxylase activity	At2g45550	-0.11	0.08	-0.03	-0.35	0.00	0.00	0.00	0.00	-0.08	9.75	8.70

top 10 SHARED DOWN in CRF group G_0 (iTPS 4-h and 6-h)

	ID / treatment	p8-d8	p12-d12	p20-d20	p24-d24	FN - St	30mSUC - St	3hSUC - St	30mGl c-St	350 - 50 CO_2	Log_2FC iTPS 4h	Log_2FC iTPS 6h
hypothetical protein	At3g14340	0.35	-0.08	-0.05	0.32	-0.01	0.03	-0.40	0.00	0.14	-7.90	-9.50
transmembrane protein	At3g13130	0.12	0.03	0.01	0.12	0.00	0.00	0.00	0.00	0.06	-6.31	-3.36
basic helix-loop-helix (bHLH) DNA-binding superfamily	At1g49830	-0.14	0.15	-0.19	0.00	0.00	0.00	0.00	0.00	0.00	-6.19	-3.17
Encodes a root meristem growth factor (RGF). Belongs to a family of functionally redundant homologous peptides that are secreted, tyrosine-sulfated, and expressed mainly in the stem cell area and the innermost layer of central columella cells	At4g16515	-0.40	-0.12	0.22	0.00	0.34	0.14	-0.04	0.00	0.37	-6.08	-5.96
nodulin MtN21-like transporter family protein	At5g64700	-0.33	-0.32	0.58	0.38	0.00	0.17	0.00	0.00	0.04	-5.32	-3.79
member of Alpha-Expansin Gene Family. Involved in the formation of nematode-induced syncytia in roots of Arabidopsis thaliana	At2g40610	**-1.75**	-0.53	0.99	0.76	0.23	-0.25	-0.97	0.00	1.10	-5.23	-4.14
CCT motif family protein	At5g59990	-0.07	0.11	0.01	0.00	0.00	0.00	0.00	0.00	-0.15	-5.01	-2.87
Encodes a Superman (SUP) like protein with zinc finger motifs. Transcript is detected in petal primordia and protein is localized to the nucleus.	At5g06070	-0.03	0.00	0.11	0.10	0.00	0.00	0.00	0.00	0.00	-4.88	-4.92
Cysteine proteinases superfamily protein	At1g29090	-0.30	0.18	0.34	-0.12	0.00	0.00	0.00	-0.93	-0.01	-4.77	-5.11
Encodes MINI ZINC FINGER 1 (MIF1) which has a zinc finger domain but lacks other protein motifs normally present in transcription factors. MIF1 physically interact with a group of zinc finger-homeodomain (ZHD) transcription factors, such as ZHD5 (AT1G75240), that regulate floral architecture and leaf development.	At1g74660	0.14	0.07	-0.26	-0.21	0.32	0.13	0.29	0.00	-0.02	-4.75	-2.04

Figure S6. Changes in expression of selected G0 genes in response to various treatments by which the CRF was determined and abundance of transcripts of genes assigned to G_0 (continued).

(C) **Impact of transcript abundance on assignment to CRF groups G_0.** All transcripts in a given group were ordered on abundance in the 4h water-sprayed 29.2 control (blue). The plots also show the abundance in the 4h ethanol-sprayed treatment. Taking a filter of 10 counts, there is a larger proportion (of low-expressed gene in the G_0 transcript set (15- 20%) than the G_1 and G_2 sets (under 2%) but the majority of transcript assigned to G_0 are present at abundances similar to those in the G_1 and G_2 sets. The analysis was performed with genes where the iTPS was significant at FDR<0.05.

Note that the x-axes are scaled to have the same value for all genes in set and are, per gene, more expanded for the G_2 and G_0 plots than the G_1 plot

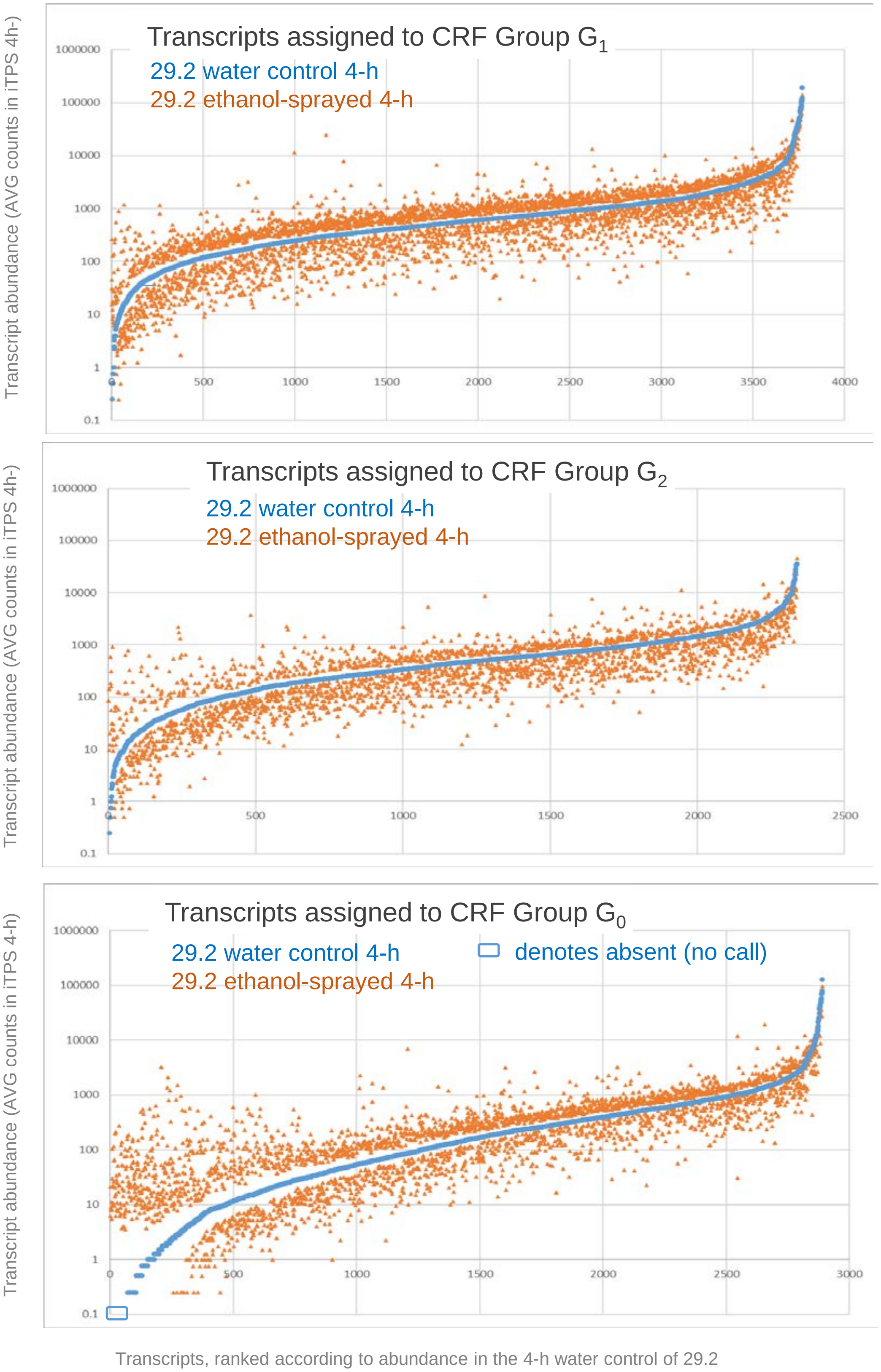

Supplemental Figure S7. Visualization of selected responses in metabolism using PageMan displays (supplemental to Figure 3). (A) Photosynthesis, (B) Gluconeogenesis/glyoxylate cycle, (C) N metabolism, (D) Nucleotide metabolism, (E) Specialized metabolism, (F) Protein, (G) Cell wall. The analysis was conducted using PageMan (Usadel et al., 2006) and MapMan software (version 3.6.0RC1; https://mapman.gabipd.org/; Ath_AGI_LOCUS_TAIR10_Aug2012). The analysis was performed as in Figure 3. The analysis was performed separately for the sets of genes that were assigned to the CRF groups G_1, G_2 and G_0 (see Supplemental Figure S2) and for the responses at 4h and 6h after spraying. The CFR groups are shown from left to right in the block in which the 4h and 6h response is displayed. The analyses used the $\log_2$FC values for all genes in a given category. These were filtered (FDR <0.05, $\log_2$ FC ≥0.2; all values that did not pass the filter were set to zero) and all individual $\log_2$FC values (including the zero values) were then averaged for all genes in that category. The average $\log_2$FC values for each subBIN are displayed as a heat map (for scale see insert). CRF is carbon response factor, FC is fold change, iTPS is the response to induced expression of bacterial TPS.

A. Analysis at the highest level of the MapMan ontology (repeat of the analysis shown in Figure 3, but with a weaker cut-off filter)

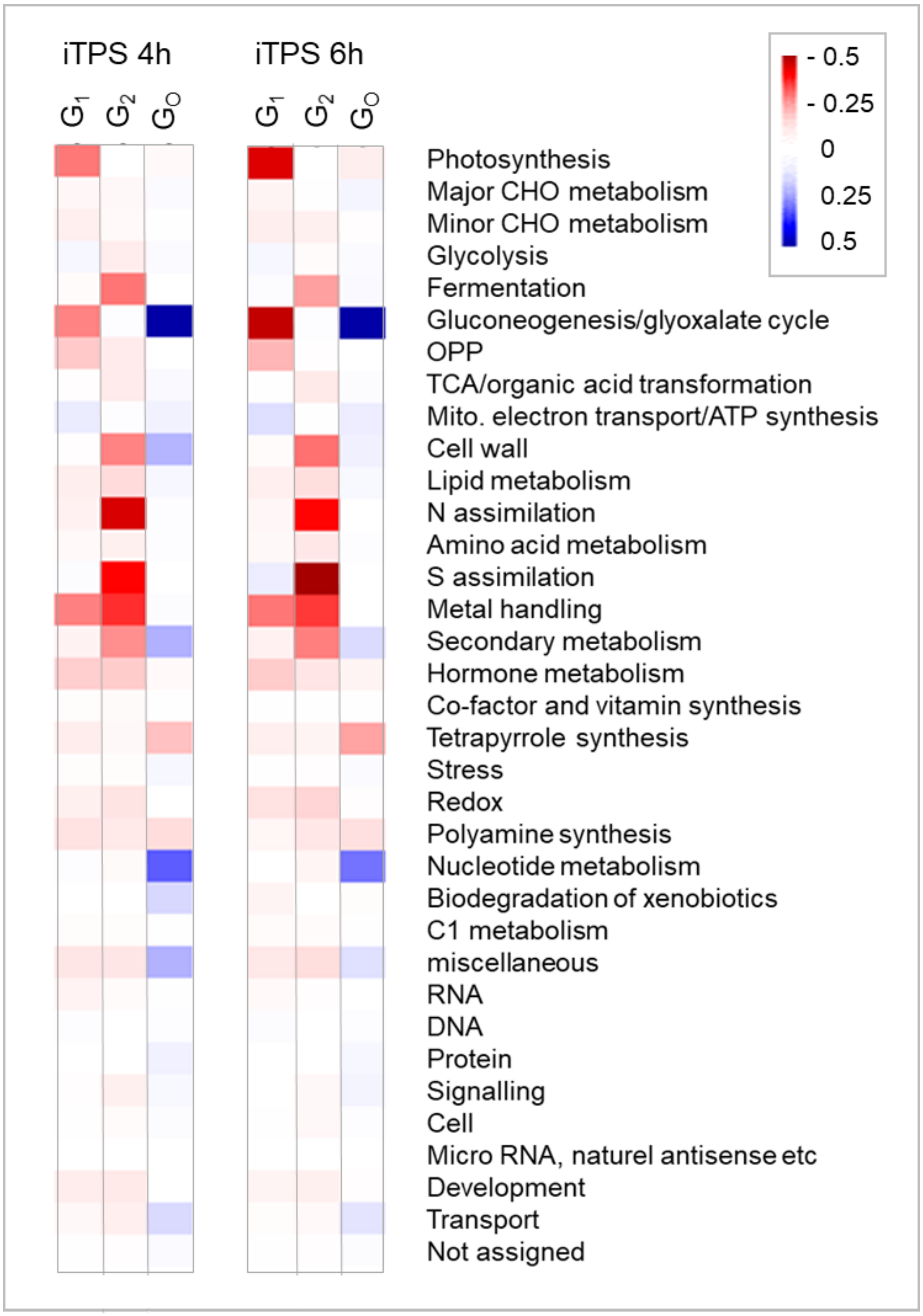

The PageMan tool sets all changes that are below the cut-off filter to zero before averaging responses in a given BIN or subBIN. These average responses are shown as a heatmap in the display. In the case where there is a coordinated response but for many genes the change is below the cutoff filter, this procedure depresses the average change.

This is why the averaged responses in the analysis with a cut-off filter of $\log_2$ FC ≥ 0.2 (this panel) are sometimes larger than in the analysis with a cut-off filter of FC ≥2 (Figure 3).

This is especially the case for genes assigned to G_1 (see, for example, the BINs for photosynthesis, gluconeogenesis /glyoxylate cycle, the OPP pathway, mitochondrial electron transport/ATP synthesis, and tetrapyrrole synthesis).

The similar or stronger average response of genes assigned to G_1 in the analysis with the lower cut-off confirms the analysis of Figure 3. it also shows that genes in the BIN are responding in a coordinated manner to elevated Tre6P.

The detailed analyses of responses within selected BINs that are provided in the following panels B-I were performed with the lower cut-off filter ($\log_2$ FC ≥ 0.2)

Supplemental Figure S7, continued

B. Photosynthesis

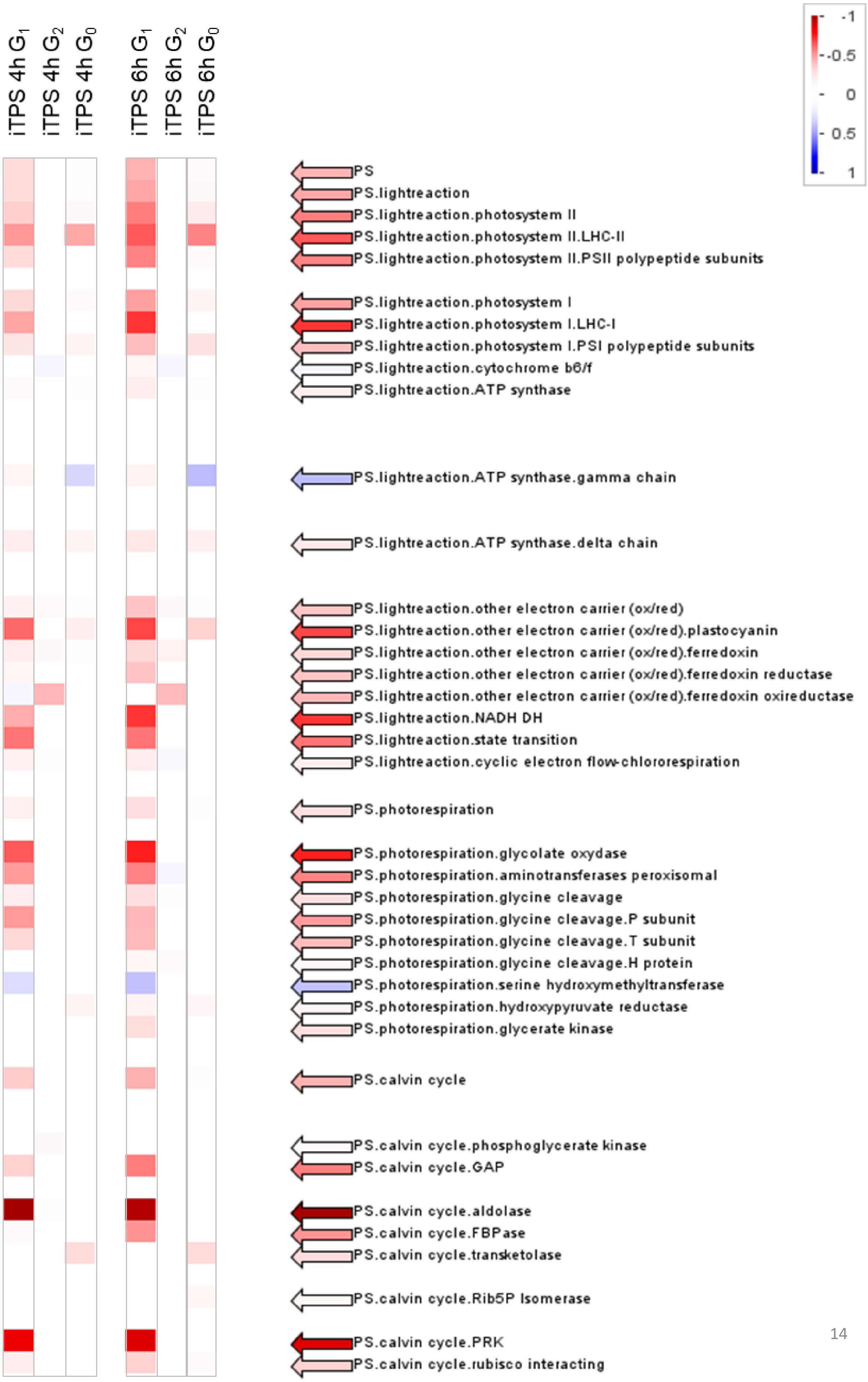

Supplemental Figure S7, continued

C. Gluconeogenesis/glyoxylate cycle

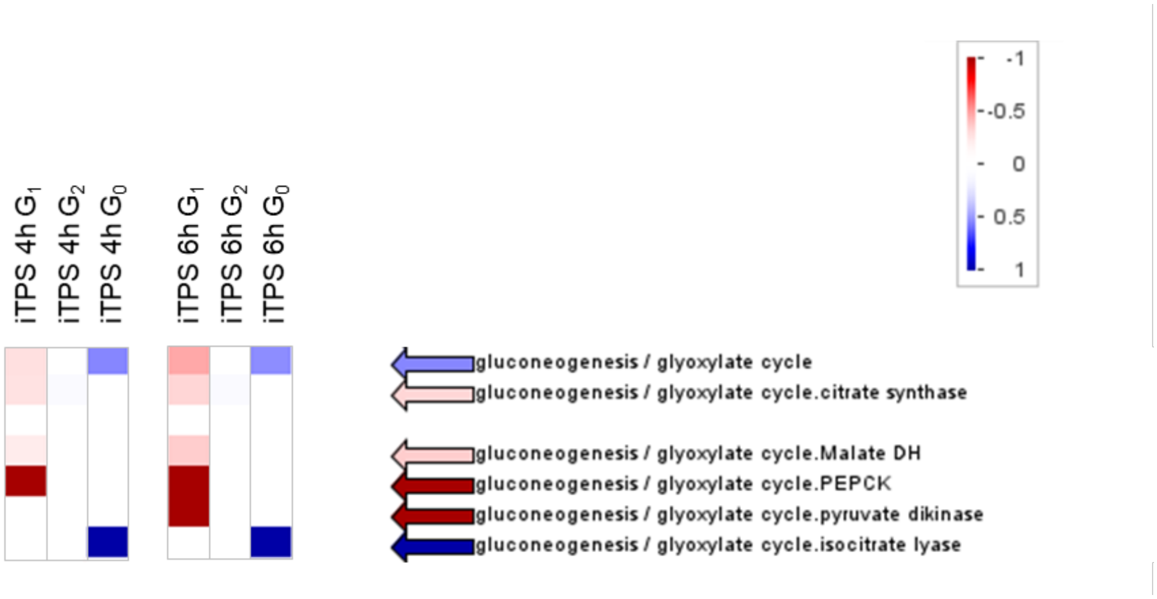

D. N metabolism

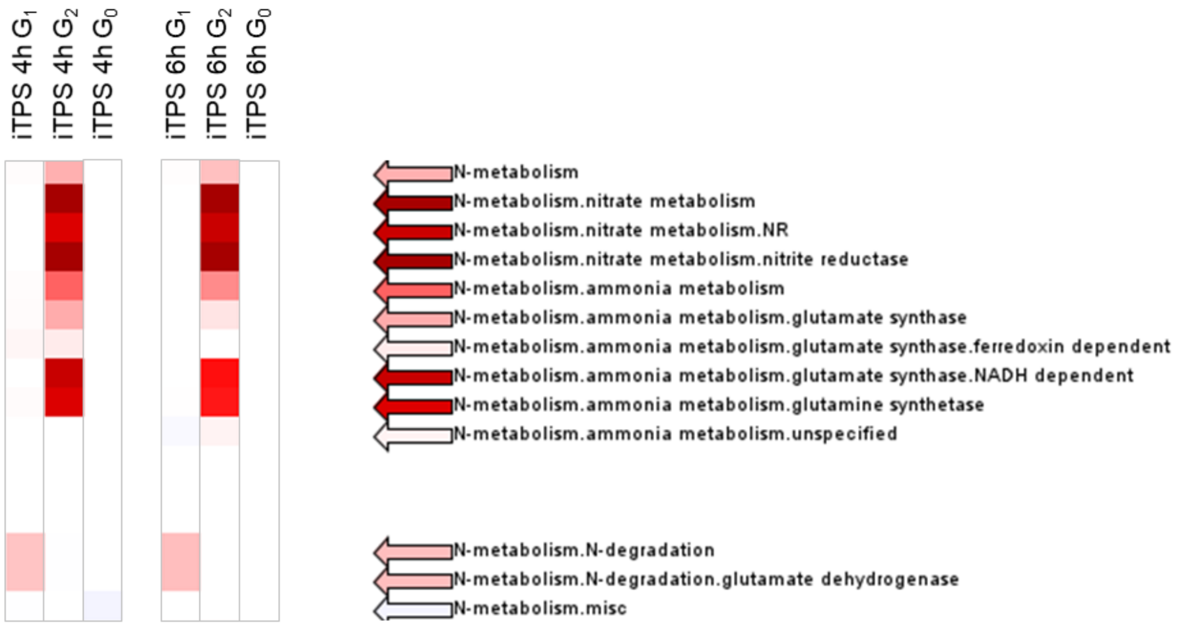

Supplemental Figure S7, continued

E. Nucleotide metabolism

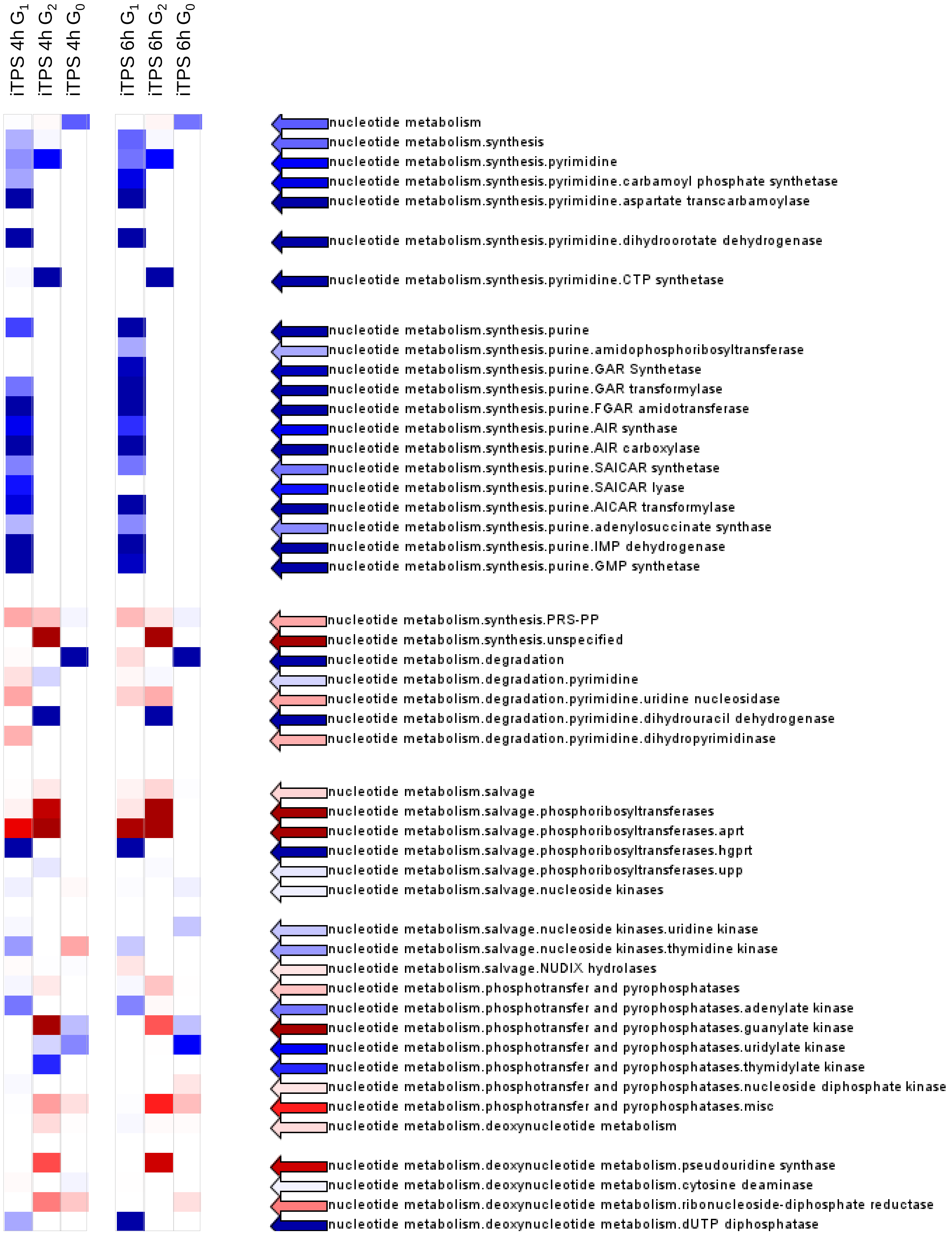

Supplemental Figure S7, continued

F. Specialized metabolism
(continued on next pages)

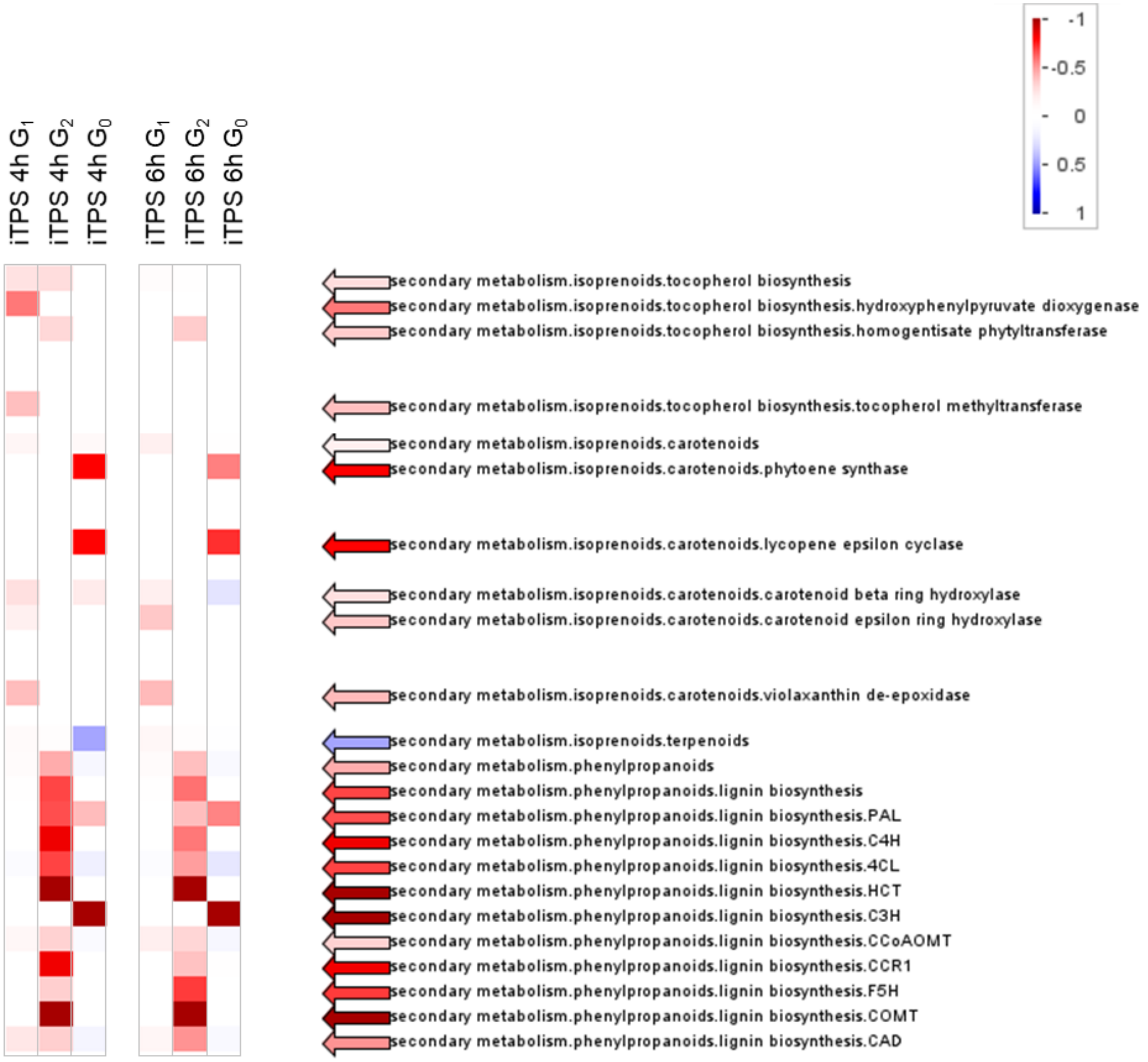

Supplemental Figure S7, continued

F. Specialized metabolism, continued
(mainly S-containing secondary metabolites)

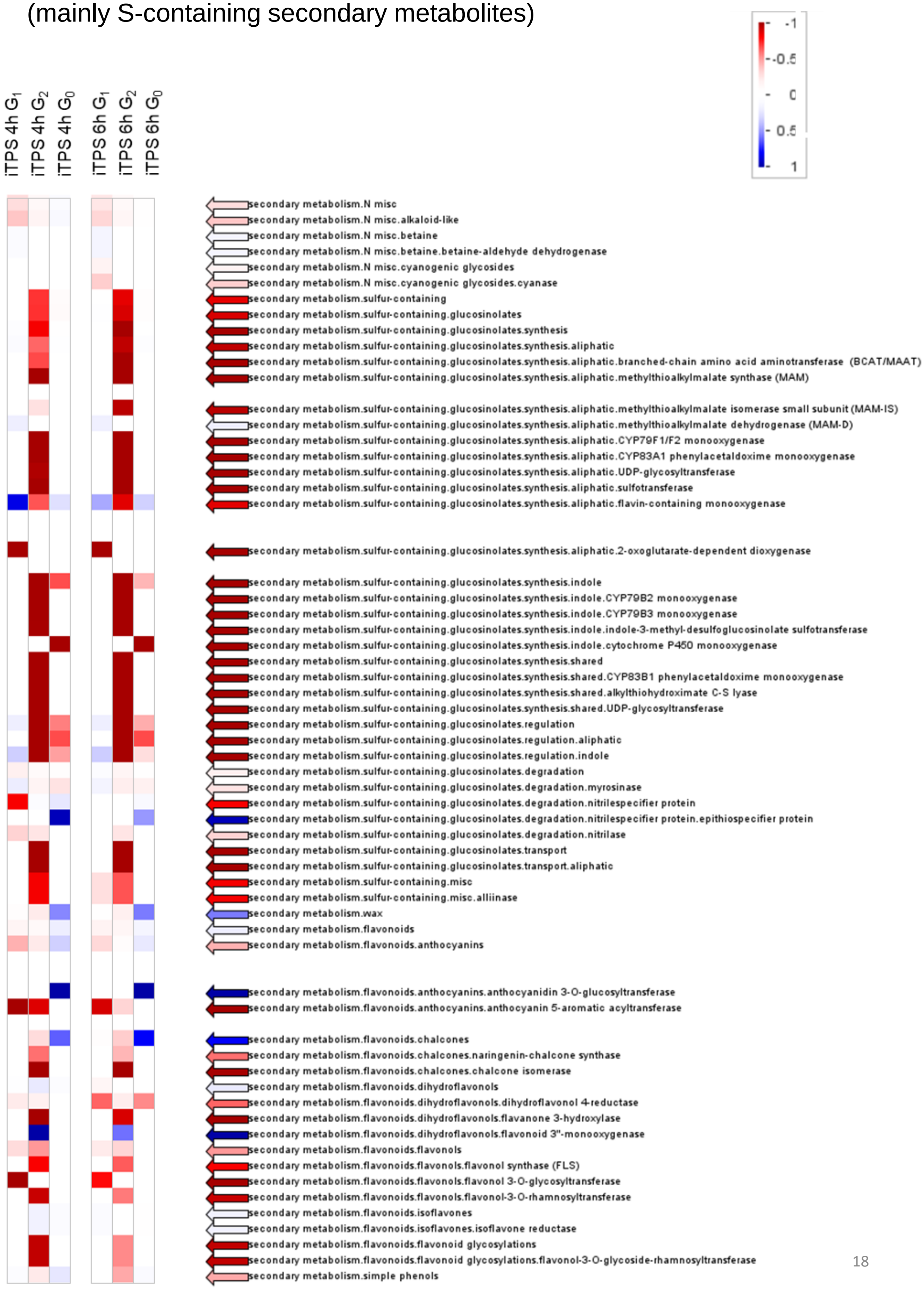

Supplemental Figure S7, continued

G. Protein

Chloroplast and mitochondrial ribosomal proteins

Genes annotated as prokaryotic-type ribosomal proteins.misc. (i.e., not assigned to the plastid or mitochondria) are omitted

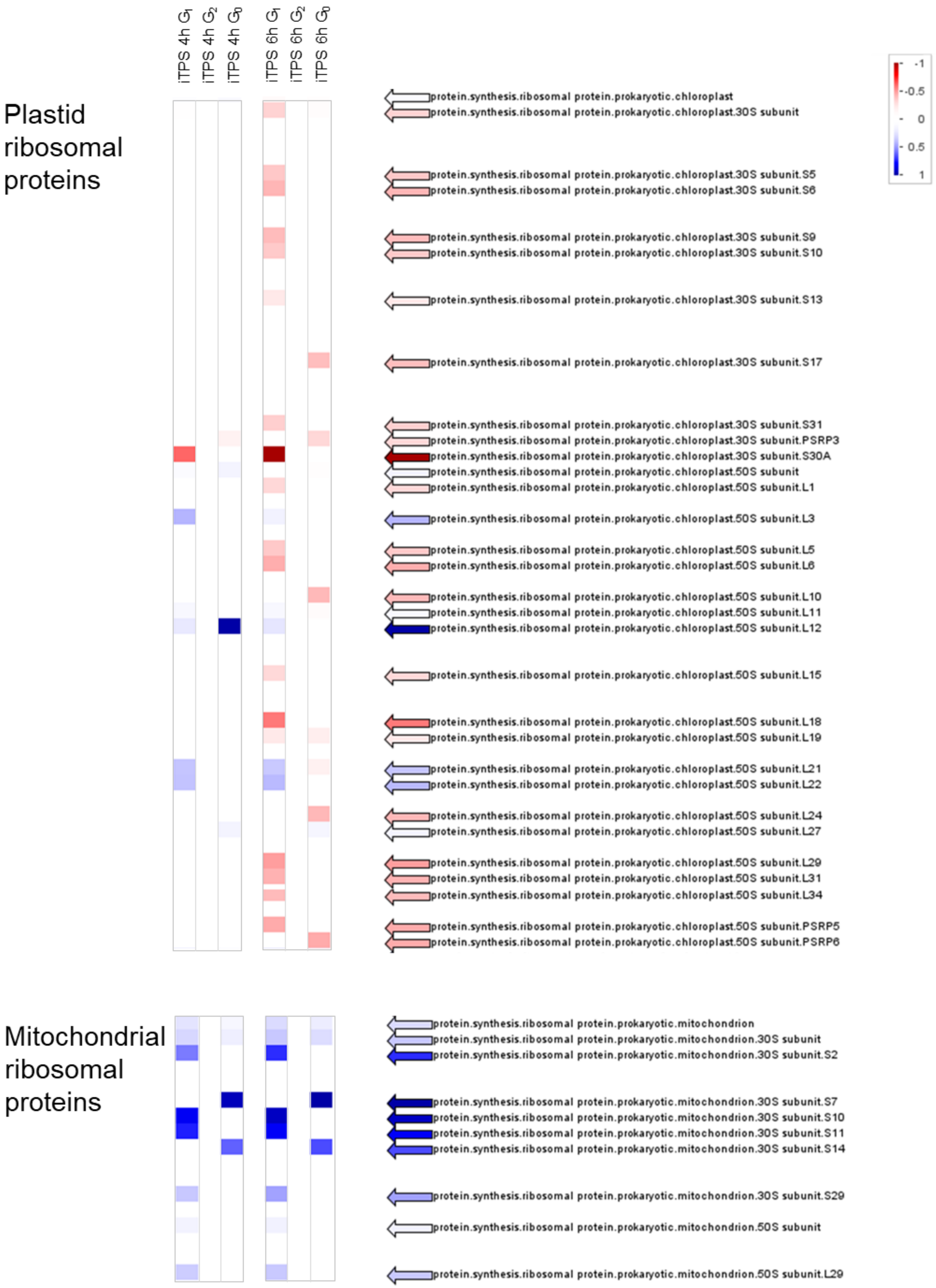

Supplemental Figure S7, continued

G. Protein (continued)

Eukaryotic (cytosolic) ribosomal proteins

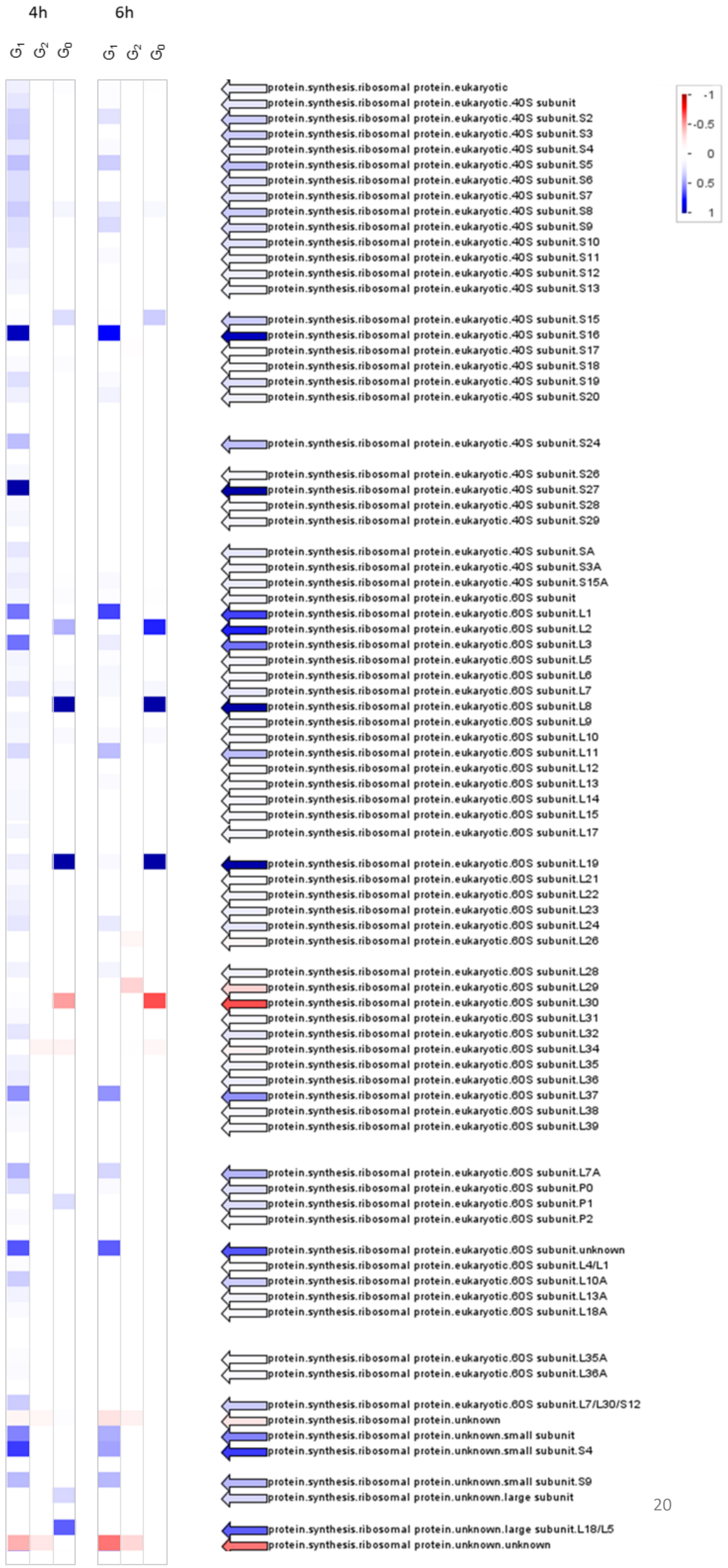

Supplemental Figure S7, continued

G. Protein (continued)

Ribosome biogenesis

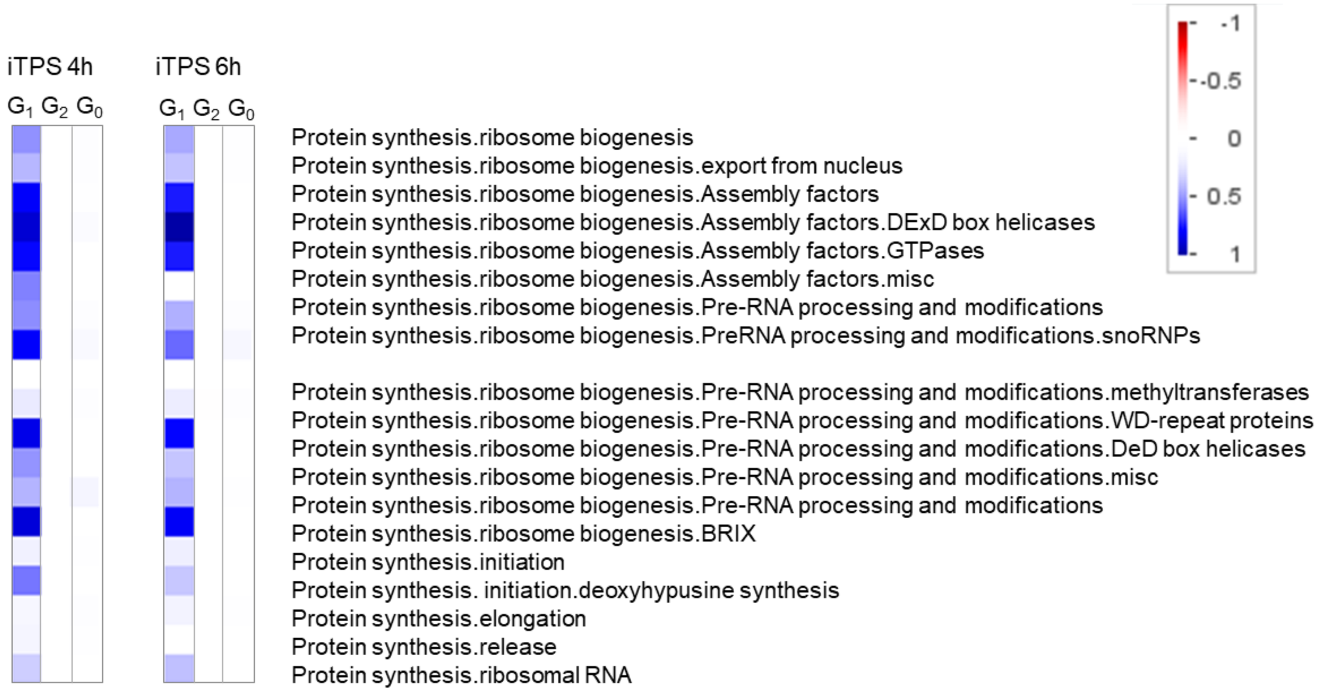

This panel repeats the analysis of Figure 4A but with a weaker cut-off filter. (log_2 FC ≥ 0.2 instead of FC ≥ 2). The analysis confirms that elevated Tr6P induces genes assigned to ribosomal assembly, with larger average changes in the subBINs and with the response extending to more subBINs.

As explained in the legend to Supplemental Figure S7A, when a strong cut-off filter is applied the PageMan analysis depresses the average change because all genes that lie below the cut-off filter are set to zero before calculating the average response in a BIN or subBIN. This can also lead to subBINs not being called even though there is a consistent increase, none of the individual responses are above the cut-off filter.

Overall, this analysis confirms and strengthens the conclusion from Figure 4, that elevated Tre6P leads to coordinated induction of genes involved in ribosome biogenesis

Supplemental Figure S7, continued

G. Protein Continued

Protein targeting

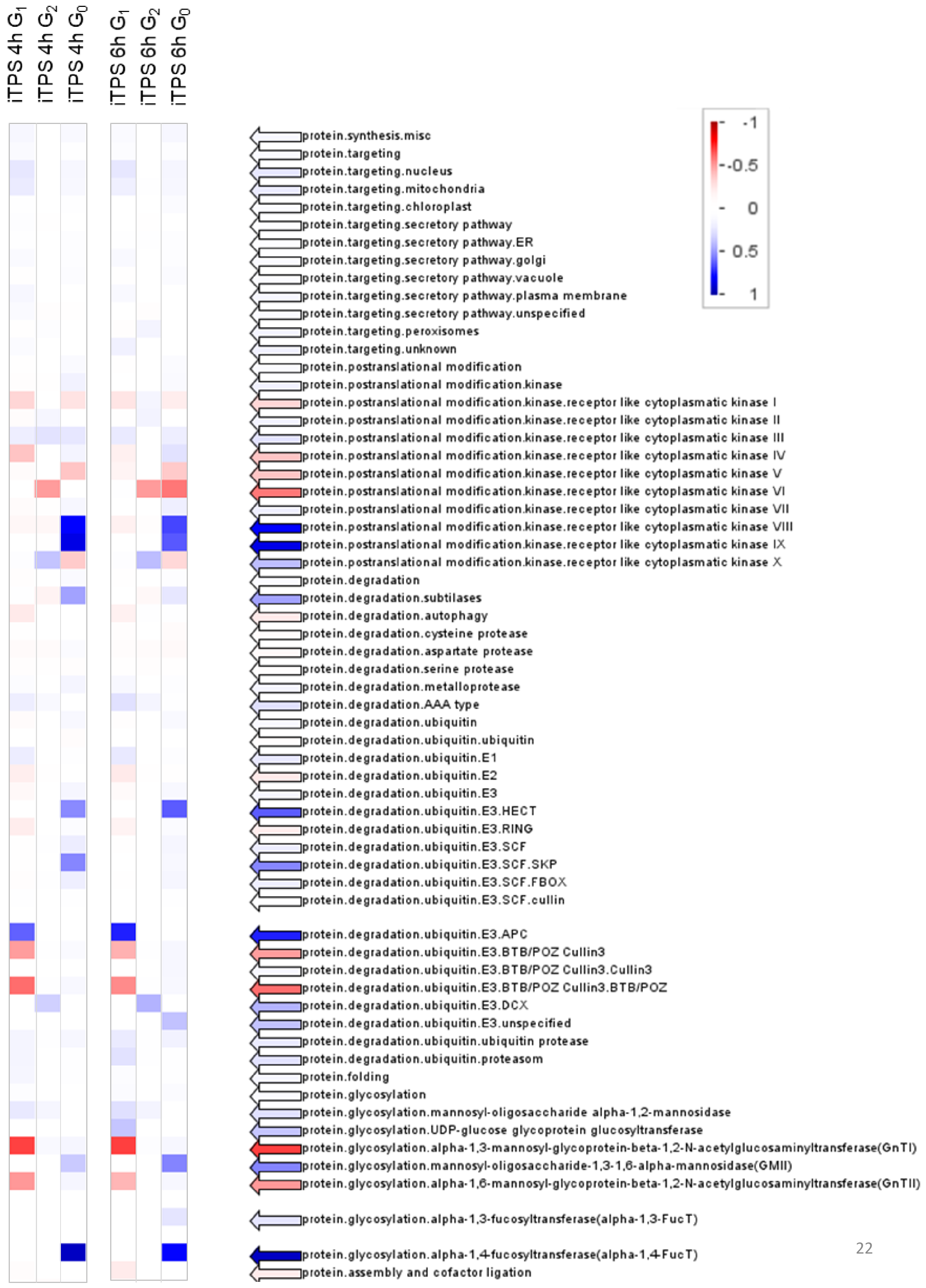

Supplemental Figure S7, continued

H. Cell Wall

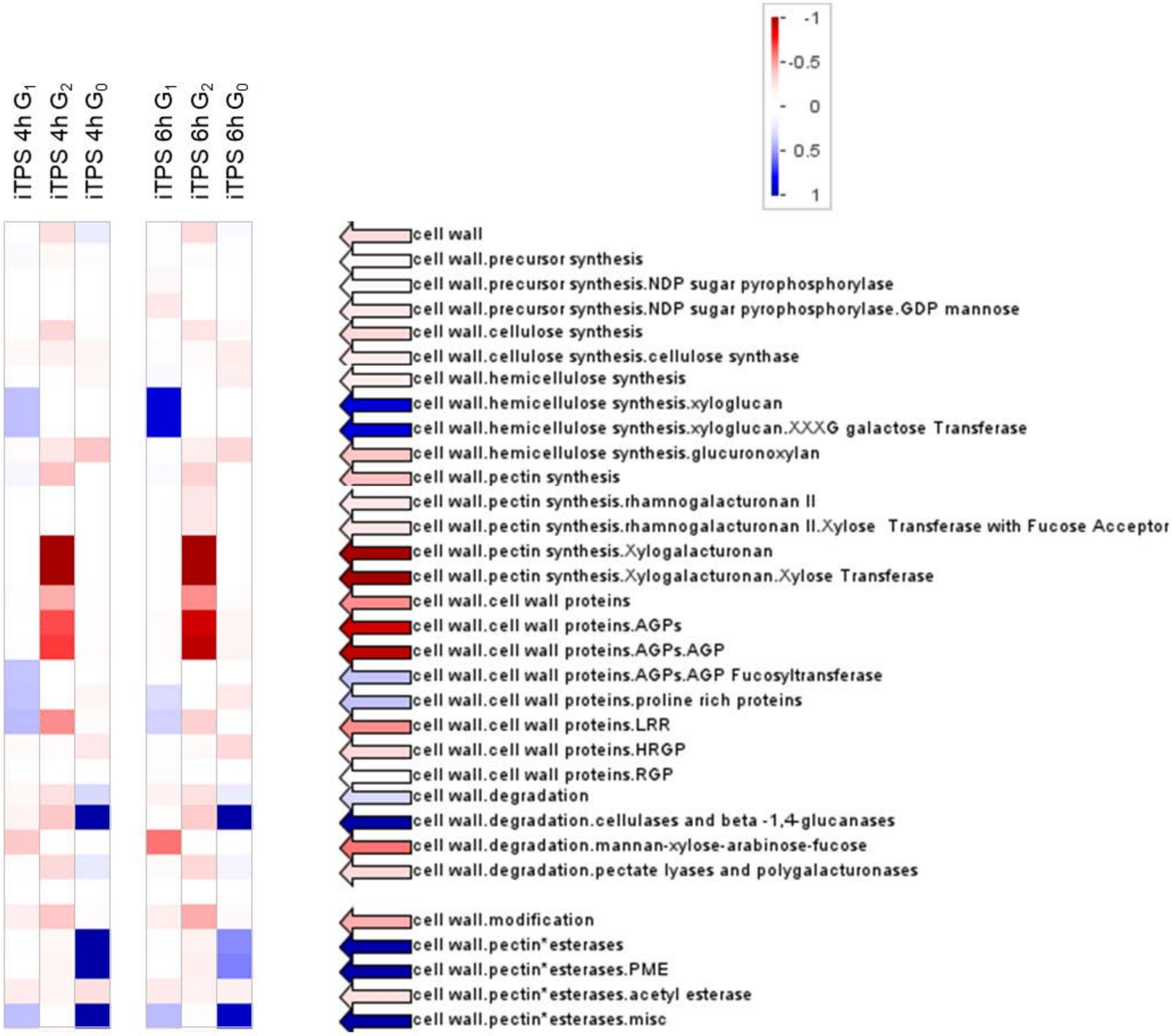

Supplemental Figure S8. Responses of transcripts encoding proteins involved in sucrose transport and metabolism.

Responses are shown separately for the 4-h and 6-h iTPS response, assignment to CRF groups is denoted by shading (G_1 orange, G_2 blue, G_0 white, no significant change white), numbers represent $\log_2$ FC values and the direction of change is depicted by font color (blue for increase and red for decrease). FC denotes fold change, iTPS is the response to induced expression of bacterial TPS.

Information about the expression and function of SWEET family members is from Xue et al. (2022) and Braun (2022)

		iTPS response 4-h	6-h 6h	Clade	Substrate, Expression, Function,
AT1G21460	SWEET1	-1.3	-0.6	I	hexoses, embryo, ABA control of germination
AT3G14770	SWEET2	+0.3	+0.3	I	hexoses, roots, vacuolar storage of hexoses
AT5G53190	SWEET3			I	hexoses,
AT3G28007	SWEET4	-0.8	-1.4	II	hexoses leaf veins, prevents chlorosis
AT5G62850	SWEET5	-1.1	-0.9	II	hexoses, galactose inhibition of pollen germination
AT1G66770	SWEET6			II	hexoses,
AT4G10850	SWEET7			II	hexoses,
AT5G40260	SWEET8			II	hexoses, tapetum, pollen development
AT2G39060	SWEET9			III	sucrose, flower, sucrose release in nectar formation
AT5G50790	SWEET10			III	sucrose, mature vascular tissue and shoot apex in flowering
AT3G48740	SWEET11	-1.9	-2.1	III	sucrose, phloem parenchyma, sucrose export
AT5G23660	SWEET12	-4.5	-3.2	III	sucrose, phloem parenchyma, sucrose export
AT5G50800	SWEET13	-2.8	-1.2	III	sucrose, bundle sheath, sucrose export , also tapetum
AT4G25010	SWEET14			III	sucrose, anther dehiscence and dehydration, GA signalling
AT5G13170	SWEET15	-1.6		III	sucrose, senescing tissues,
AT3G16690	SWEET16	-1.0	-1.6	IV	fructose, tonoplast of leaf cells, vacuolar storage
AT4G15920	SWEET17	-2.9	-2.7	IV	fructose, tonoplast of root cells, vacuolar storage
AT1G71880	SUC1	-0.6			
AT1G22710	SUT1/SUC2		-0.8		
AT2G02860	SUT2/SUC3	+1.0	+1.0		
AT1G09960	SUT4/SUC4		+0.4		
AT1G71890	SUC5	-3.2	-2.0		
AT5G43610	SUC6				
AT1G66570	SUC7				
AT2G14670	SUC8				
AT5G06170	SUC9				
AT5G20830	SUS1	-3.2	-2.0		
AT5G49190	SUS2	+0.8	+0.8		
AT4G02280	SUS3	+1.6	+1.8		
AT3G43190	SUS4	-1.6	-2.7		
AT5G37180	SUS5	-0.8	-1.0		
AT1G73370	SUS6		-0.4		
AT1G62660	VINV1		-0.6		
AT3G13790	CWINV1	+0.9	+1.1		
AT3G52600	CWINV2				
AT2G36190	CWINV4				
AT3G13784	CWINV5	+3.5	+2.5		
AT1G56560	A/N-InvA		+0.6		
AT4G34860	A/N-InvB		+0.5		
AT3G06500	A/N-InvC	-1.4	-0.9		
AT1G22650	A/N-InvD		+0.9		
AT5G22510	A/N-InvE	-0.4	-0.3		
AT1G72000	A/N-InvF				
AT1G35580	A/N-InvG		-0.9		
AT3G05820	A/N-InvH	-1.3			
AT4G09510	A/N-InvI	+1.1	+1.0		
AT5G20280	SPS1		+0.4		
AT5G11110	SPS2		+0.5		
AT1G04920	SPS3	+0.9	+0.7		
AT4G10120	SPS4	-2.2	-2.0		

Suc and SUT is sucrose transporter
SUS is sucrose synthase
VINV is vacuolar invertase
CWINV is cell wall invertase
A/NINV is acid/neutral invertase
SPS is sucrose phosphate synthase

CRF group 1
CRF group 2
CRF group 0
No significant change

Supplemental Figure S9. Responses of selected transcripts encoding proteins involved in the regulation of floral induction.

Responses are shown separately for the 4-h and 6-h iTPS response, assignment to CRF groups is denoted by shading (G_1 orange, G_2 blue, G_0 white, no significant change white), numbers represent $\log_2$ FC values and the direction of change is depicted by font color (blue for increase and red for decrease). CRF is carbon response factor, FC is fold change, iTPS is response to induced expression o fbacterial TPS.

			iTPS response 4-h	iTPS response 6-h
	AT4G20370	TSF		
	AT4G35900	FD	-1.0	-1.4
	AT1G65480	FT		
	AT5G03840	TFL1	-2.0	-2.0
	AT5G06850	FTIP1	-2.0	-2.0
	AT5G10140	FLC		
Floral meristem identity	AT4G22950	AGL19		
Floral meristem identity	AT5G60910	FUL	-1.0	-0.7
Floral meristem identity	AT4G24540	AGL24		
Floral meristem identity	AT5G61850	LFY	+0.9	
Floral meristem identity	AT1G69120	AP1		
	AT2G45660	SOC1	-1.5	-1.0
	AT5G15840	CO	+0.6	
	AT2G33810	SPL3	-1.7	-1.9
	AT1G53160	SPL4	-1.0	-1.0
	AT3G15270	SPL5	-1.0	
	AT2G42200	SPL9	-0.8	-0.8
	AT1G27370	SPL10		
	AT2G22540	SVP	-1.0	-0.7
	AT2G43010	PIF4	-2.4	-2.0
	AT4G31610	REM1		
	AT5G17800	MYB56		
	AT5G65070	MAF4		
	AT5G65080	MAF5		
	AT5G05660	NFXL2	+0.2	
	AT5G57360	ZTL	+0.3	+0.4
	AT5G61380	PRR1		
	AT1G18080	RACK1		
	AT3G22380	TIC	-1.3	-0.6
flowering-time inhibitors – hormonal pathway	AT3G05120	GID1A	-0.8	-0.5
flowering-time inhibitors – hormonal pathway	AT3G63010	GID1B		-0.8
flowering-time inhibitors – hormonal pathway	AT5G27320	GID1C	-1.0	-0.7
flowering-time inhibitors – hormonal pathway	AT1G25560	TEM1	-1.6	-1.5
flowering-time inhibitors – hormonal pathway	AT1G68840	TEM2	-2.3	-2.1
flowering-time inhibitors – hormonal pathway	AT2G01570	RGA1	-0.6	-0.8
flowering-time inhibitors – hormonal pathway	AT1G14920	RGA2	-2.0	-1.7
flowering-time inhibitors – hormonal pathway	AT1G66350	RGL1	-2.5	-2.4
flowering-time inhibitors – hormonal pathway	AT3G03450	RGL2		+0.4
flowering-time inhibitors – hormonal pathway	AT5G17490	RGL3	+0.3	+0.4

CRF group 1
CRF group 2
CRF group 0
No significant change

Supplemental Figure S10. Responses of transcripts encoding proteins involved in the circadian clock.

Responses are shown separately for the 4-h and 6-h iTPS response, assignment to CRF groups is denoted by shading (G_1 orange, G_2 blue, G_0 white, no significant change white), numbers represent log_2 FC values and the direction of change is depicted by font color (blue for increase and red for decrease). CRF is carbon response factor, FC is fold change, iTPS is response to to induced expression of bacterial TPS.

The right-hand display summaries the diel oscillation of these genes in plants grown in similar conditions, showing the fold change between the diel maximum and the diel minimum, and the time of the peak (given as time after dawn, in hours). Transcripts were quantified using qRT-PCR with internal standards to allow absolute quantification. The data are taken from Moraes et al. (2019).

iTPS response (FC, Log_2)

		4-h	6-h
AT1G01060	LHY		+0.7
AT2G46830	CCA1		+0.6
AT2G39180	CCR2		-0.6
AT2G46790	PRR9	-0.3	-0.4
AT5G02810	PRR7		
AT5G24470	PRR5	-1.3	-1.8
AT5G61380	TOC1		
AT1G22770	GI	-0.6	-0.7
AT2G25930	ELF3	-0.4	-0.5
AT2G40080	ELF4	-1.9	-1.5
AT3G46640	LUX	-0.7	-1.2

Diel oscillation

	FC Log_2	Peak time h after dawn
LHYC	9.7	23.7
CCA1	9.4	23.9
PRR9	11.0	4.2
PRR7	7.4	5.9
PRR5	7.9	7.9
TOC1	3.9	10.9
GI	8.2	7.6
ELF3	2.9	16.3
ELF4	8.9	10.3
LUX	4.7	9.7

CRF group 1
CRF group 2
CRF group 0
No significant change

Supplemental Figure S11. Enrichment analyses using Gene Ontology. (A) List of enriched and underrepresented GO terms. The analysis was performed by the Gene Ontology tool (http://geneontology.org/). Entries were restricted to transcripts showed a significant response (FDR <0.05) and showed a FC≥2. An analysis with transcripts that showed a significant change in abundance (FDR < 0.05) irrespective of the size of the FC is provided in Supplemental Dataset S5 .

CRF group	enrichment UP	enrichment DOWN
**G_1**<br>Up- 307<br>Down-840	• mitochondrial RNA modification<br>• mitochondrial gene expression<br>• RNA splicing<br>• ribonucleoprotein complex biogenesis	• negative regulation of cytokinin-activated signaling pathway<br>• trehalose metabolism in response to stress<br>• abaxial cell fate specification<br>• phototropism<br>• entrainment of circadian clock<br>• water transport<br>• response to nitrate<br>• response to far red light<br>• response to light intensity<br>• ethylene-activated signaling pathway<br>• response to red light<br>• response to nematode<br>• response to gibberellin<br>• regulation of stomatal movement<br>• regulation of seedling development<br>• response to auxin<br>• response to chitin<br>• response to water deprivation<br>• response to jasmonic acid<br>• response to abscisic acid<br>• cellular response to oxygen-containing compound<br>• developmental growth<br>• organic substance catabolic process<br>• protein transport<br>• intracellular transport<br>• mRNA processing<br>• DNA repair<br>• translation<br>• meiotic cell cycle process
**G_2**<br>Up –180<br>Down - 756	• regulation of seed dormancy process<br>• hyperosmotic salinity response<br>• response to salicylic acid<br>• response to water deprivation<br>• response to abscisic acid<br>• cellular response to hormone stimulus<br>• signal transduction	• arsenite transport<br>• adenine salvage<br>• indoleacetic acid biosynthetic process<br>• guard mother cell differentiation<br>• nitrate assimilation<br>• regulation of glucosinolate biosynthetic process<br>• syncytium formation<br>• L-phenylalanine biosynthetic process<br>• cutin biosynthetic process<br>• wax biosynthetic process<br>• plant-type cell wall loosening<br>• oligopeptide transport<br>• brassinosteroid biosynthetic process<br>• auxin polar transport<br>• response to nitrate<br>• lateral root formation<br>• amino acid transmembrane transport<br>• flavonoid metabolic process<br>• fatty acid biosynthetic process<br>• response to gibberellin<br>• jasmonic acid mediated signaling pathway<br>• developmental growth<br>• regulation of DNA-templated transcription<br>• protein modification process<br>• translation<br>• rRNA processing<br>• establishment of protein localization to organelle
**G_0**<br>Up – 710<br>Down - 599<br><br>*under-represented processes	• cytidine deamination<br>• geranylgeranyl diphosphate biosynthetic process<br>• regulation of pollen tube growth<br>• pectin catabolic process<br>• RNA modification<br>• protein phosphorylation<br>• gene expression<br>• response to light stimulus<br>• cellular nitrogen compound biosynthetic process	• xylem vessel member cell differentiation<br>• formation of plant organ boundary<br>• asymmetric cell division<br>• cotyledon development<br>• cell wall modification<br>• response to cytokinin<br>• response to auxin<br>• glucosinolate metabolic process<br>• response to red or far red light<br>• meristem development<br>• positive regulation of DNA-templated transcription<br>• response to light intensity<br>• developmental growth<br>• response to abscisic acid<br>• root development<br>• response to wounding<br>• translation<br>• ribosome biogenesis<br>• ncRNA processing

Supplemental Figure S12. Comparison of the changes in transcript abundance an induced increase in Tre6P, and in Arabidopsis lines that constitutively express bacterial TPS.(supplemental to Figure 6 and Supplemental Table S5. The response to constitutive overexpression of TPS (termed oeTPS) is from Zhang et al. (2009) was analyzed using a CATMA array; responsive transcripts were defined using a filter of FDR>0.05 and FC≥2.

(A) Regression plots of oeTPS against the iTPS response of the corresponding gene, either without filtering the iTPS data or after filtering the iTPS data sets with FDR <0.05 or with FDR<0.05 and FC≥2 (left, middle and right side, respectively). Plots are shown separately for the 4h (top row) and the 6h (bottom row) iTPS responses. The number in brackets in the panel heading gives the number of transcripts in the subset plotted in the panel.

(B) Regression plots of oeTPS (Zhang et al., 2009) against the iTPS response after filtering (FDR<0.05) and assigning genes to the CRF groups G_1, G_2 or G_0.

(C) Comparative analysis of the response of genes involved in light–signaling. Shown on next page.

(D) Gene ontology analysis of a robust set of Tre6P-regulated genes that show significant iTPS responses, were assigned to CRF group G_1 and were scored as responsive to oeTPS by Zhang et al. (2009) and showed the same qualitative response as in our iTPS dataset. Shown on next page.

FDR is false rate discovery, FC is fold change, DEG is differentially expressed gene, iTPS is response to induced expression of TPS

A oeTPS versus iTPS, All shared genes, unfiltered or filtered on FDR or FDR plus FC

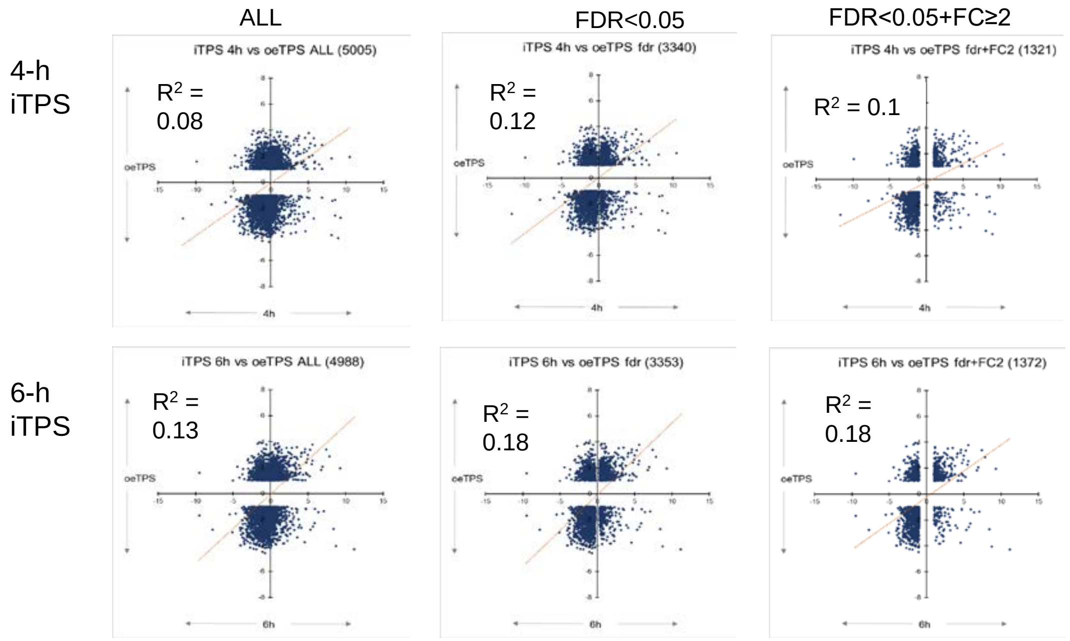

B. oeTPS versus iTPS, after assigning DEGs to G_1, G_2 and G_O

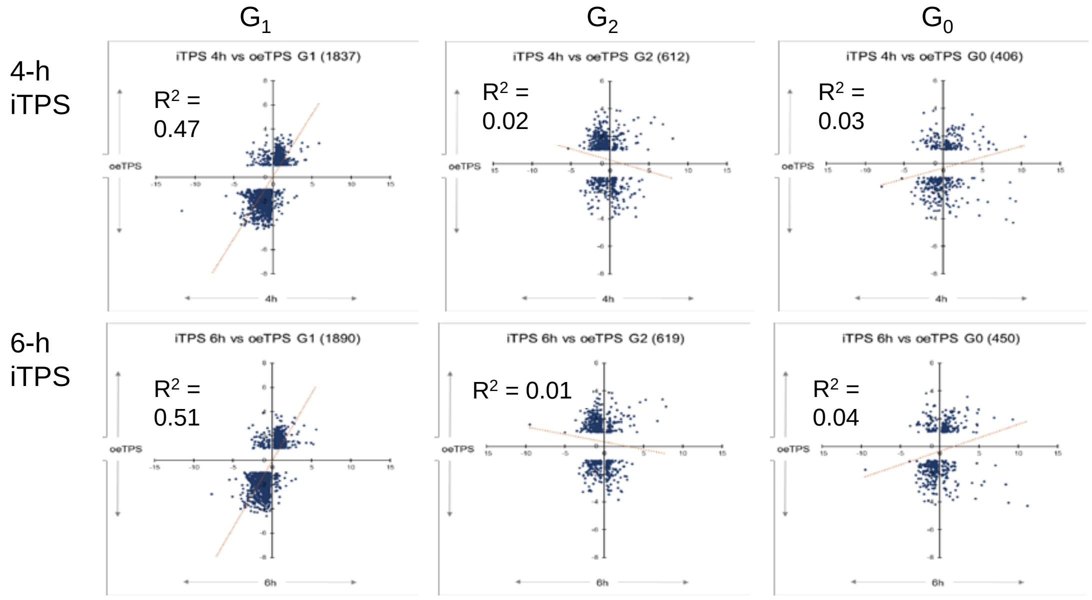

Supplemental Figure S12 (continued)

C

Comparison of the response of genes involved in light–signaling to iTPS and oeTPS. The upper part of the display investigates genes highlighted as involved in light signaling and being repressed by constitutive overexpression of TPS (oeTPS) by Paul et al. (2010) (changes are given for the ratio to control as in Paul *et al* (2010) and the FC as in Zhang et al. (2009). These genes show qualitatively similar responses in the iTPS response, and are almost all assigned to CRF G_1 (orange shaded) implying they are regulated by Tre6P-signaling. Four highlighted genes were absent from the iTPS dataset, and the absence of a CRF assignment denotes a non-significant iTPS response. The lower Table section investigates genes involved in light signaling that showed a strong iTPS response (G_1 or G_0) but were not called in the Zhang et al. (2009) data set, or genes that were strongly induced in iTPS and based on extracted data were also induced in the oeTPS dataset from Zhang et al.(2009). Data for *HY5* was also included. The insert (top right-hand corner) is a scatter plot of the oeTPS versus iTPS for the entire gene set.

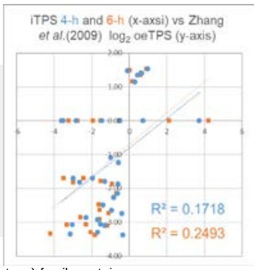

oeTPS response			Qualitative agreement of oeTPS and iTPS	iTPS response				CFR	Transient SnRK1α1	Ontology	
	Table 1, Paul et al. (2020) Ratio to control, direction of change UP DOWN	Zhang et al 2009 oeTPS FC log_2	1= same 0 = opposite na = not available	4-h FC log_2	6-h FC log_2	4h Crf score	6h Crf score		FC log_2	BINNAME_1	DESCRIPTION
At1g49130	0.08		na								
At1g75540 StH2	0.10	-3.30	1	-2.05	-1.69	1	1	-0.26	0.51	RNA.regulation of transcript	(at1g75540):zinc finger (B-box type) family protein
At3g19850	0.10	-3.34	1	-4.19	-3.17	1	1	-0.31	0.97	signalling.light	(at3g19850):phototropic-responsive NPH3 family protein
At5g52250	0.12	-3.06	1	-2.30	-1.54	1	1	-0.95	3.11	development.unspecified	(at5g52250):transducin family protein / WD-40 repeat family protein
At3g26740 ccL	0.12	-3.07	1	-3.34	-3.03	1	1	-1.41	3.02	signalling.light	(at3g26740):CCL (CCR-LIKE)
At3g02380 cOL2	0.12	-3.38	1	-1.83	-0.36	1	1	-0.64	2.47	development.unspecified	(at3g02380):COL2 (CONSTANS-LIKE 2); zinc ion binding
At1g18330 ePr1	0.12	-3.10	1	-1.17	-1.31	1	1	-1.45	0.8	RNA.regulation of transcript	(at1g18330):EARLY-PHYTOCHROME-RESPONSIVE1
At5g37260 cir1	0.13	-2.92	1	-1.58	-1.69	1	1	-1.32	2.54	RNA.regulation of transcript	(at5g37260):myb family transcription factor CIR/RVE2
At3g45780 nPH1	0.14	-2.86	1	-2.61	-2.30	1	1	-0.35	1.86	signalling.light	(at3g45780):PHOT1 (phototropin 1); kinase
At5g17300	0.15	-2.74	1	-1.09	-0.39	1	1	-1.86	2.88	RNA.regulation of transcript	(at5g17300):myb family transcription factor
At3g15570	0.16	-2.64	1	-1.56	-0.94	1	1	-0.14	0.25	signalling.light	(at3g15570):phototropic-responsive NPH3 family protein
At1g14280	0.17	-2.48	1	-1.63	-1.40	1	1	-0.26	-0.2	signalling.light	(at1g14280):PKS2 (PHYTOCHROME KINASE SUBSTRATE 2)
At3g09150	0.21	-2.27	1	-0.85	-0.83	2	2	0.10	0.18	PS.lightreaction.other electr	(at3g09150):HY2 (ELONGATED HYPOCOTYL 2);
At5g66560	0.22	-2.15	1	-0.69	-0.62	2	2	0.18	-0.17	signalling.light	(at5g66560):phototropic-responsive NPH3 family protein
At1g67900	0.25		na								
At5g67385	0.26		na								
At2g43010 PiF4	0.27	-1.7	1	-2.43	-1.98	1	1	-0.31	0.13	signalling.light	(at2g43010):PIF4 (PHYTOCHROME INTERACTING FACTOR 4)
At3g50840	0.27		na								
At3g22104	0.27	-1.87	1	-0.92	-0.60	1	1	-0.10	-1	signalling.light	(at3g22104):phototropic-responsive NPH3 protein-related
At2g02950	0.28	-1.82	1	-2.99	-2.65	1	1	-0.80	-0.23	signalling.light	(at2g02950):PKS1 (PHYTOCHROME KINASE SUBSTRATE 1)
At4g31820 enP	0.29	-1.78	1	-1.53	-1.31	1	1	-0.72	-0.06	signalling.light	(at4g31820):phototropic-responsive NPH3 family protein
At1g18810	0.37	-1.70	1	-3.48	-3.00	1	1	-0.80	2.53	signalling.light	(at1g18810):phytochrome kinase substrate-related
At4g37590 nPY5	0.42	-1.24	1	-0.57	-0.57	1	1	-0.24	1.42	signalling.light	(at4g37590):phototropic-responsive NPH3 family protein
				**Strong in iTPS but not called in Zhang et al. 2010**							
		0	na	-1.93	-1.47	1	1	-0.17	0.81	signalling.light	(at2g40080):ELF4 (EARLY FLOWERING 4)
		0	na	-1.54	-0.55	1	1	-0.29	0.04	signalling.light	(at1g52770):phototropic-responsive NPH3 family protein
		0	na	-3.57	-3.61	0	0	0.01	0.235	signalling.light	(at5g10250):phototropic-responsive protein, putative
		0	na	-3.24	-2.85	0	0	0.05	-0.145	signalling.light	(at3g08660):phototropic-responsive protein, putative
		0	na	-2.75	-3.51	0	0	-0.04	-0.55	signalling.light	(at5g04190):PKS4 (PHYTOCHROME KINASE SUBSTRATE 4)
		0	na	2.13	0.72	0	0	0.09	-0.395	signalling.light	(at2g47860):phototropic-responsive NPH3 family protein
		0	na	4.21	3.71	0	0	0.05	0.03	signalling.light	(at2g23050):phototropic-responsive NPH3 family protein
				**Respond in Zhang et al. (2009) but not in Table in Paul et al. 2010**							
		1.34	1	0.59	0.42	1	1	0.35	-0.425	signalling.light.COP9 signal	(at5g14250):COP13 (CONSTITUTIVE PHOTOMORPHOGENIC 13)
		1.41	1	0.61	0.64	1	1	0.42	-0.575	signalling.light.COP9 signal	(at5g42970):COP8 (CONSTITUTIVE PHOTOMORPHOGENIC 8)
		1.53	1	0.91	0.96	1	1	0.48	-1.085	signalling.light.COP9 signal	(at3g61140):FUS6 (FUSCA 6)
		1.49	na	0.02	-0.11			0.27	-0.6	signalling.light.COP9 signal	(at1g71230):AJH2 (COP9-signalosome 5B); protein binding
		1.16	1	0.17	0.30		1	0.13	-0.365	signalling.light.COP9 signal	(at1g22920):AJH1 (COP9-signalosome 5A)
HY5		-1.09	1	-1.02	-0.99	1	**1**	-0.67	-0.41	RNA.regulation of transcript	(at5g11260):HY5 (ELONGATED HYPOCOTYL 5);

D, E

The assignment of many cell wall modifying genes to G_2 prompted us to inspect the response of the complete *EXPA* and *XTH* families (Supplemental Figure S18). These were already highlighted in the MapMan (Supplemental Figure S7G) and Gene ontology (Supplemental Figure S11, 'plant-type cell wall loosening') enrichment analyses as broadly-responding gene sets in the CRF group G_2 response. The detailed analysis in (Supplemental Figure S18) confirms a broad repression of most of the *EXPA* family, with most responses being assigned to CRF groups G_2 or G_0 (i.e., Tre6P-independent) and a broad repression of *XTH* family members, with some of these being assigned to CRF group G_1 and the others to G_2 and G_0 (i.e., a mix of Tre6P-dependent and Tre6P-independent responses). This could reflect the respective C demand for these different types of cell wall modification. EXPAs allow cell wall expansion without incorporation of new cell wall polymers, whilst XTHs allow this and are also required for insertion of newly synthesized hemicellulose polymers into the cell wall.
Displays are shown on the next page .

Supplemental Figure S12 (continued)

D

Gene	iTPS response						CRF	Other responses	Annotation
Code	4-h adj.p	6-h adj.p	4-h FC log_2	6-h FC log_2	4-h CRF score	6-h CRF score		oeTPS FC log_2	
At2g40610	0,00	0,00	-5,23	-4,14	9	9	-0,05	0,00	(at2g40610):ATEXPA8 (ARABIDOPSIS THALIANA EXPANSIN A8)
At1g69530	0,00	0,00	-4,10	-3,07	9	9	0,00	-2,07	(at1g69530):ATEXPA1 (ARABIDOPSIS THALIANA EXPANSIN A1)
At1g20190	0,00	0,00	-3,71	-3,67	9	9	-0,06	0,00	(at1g20190):ATEXPA11 (ARABIDOPSIS THALIANA EXPANSIN A11)
At2g20750	0,00	0,00	-3,31	-2,35	2	2	0,51	0,00	(at2g20750):ATEXPB1 (ARABIDOPSIS THALIANA EXPANSIN B1)
At4g01630	0,00	0,04	-2,49	-1,71	2	2	0,16	2,01	(at4g01630):ATEXPA17 (ARABIDOPSIS THALIANA EXPANSIN A17)
At2g28950	0,00	0,00	-2,04	-2,06	2	2	0,70	-1,03	(at2g28950):ATEXPA6 (ARABIDOPSIS THALIANA EXPANSIN A6)
At2g39700	0,00	0,00	-1,98	-1,60	2	2	0,92	0,00	(at2g39700):ATEXPA4 (ARABIDOPSIS THALIANA EXPANSIN A4)
At3g29030	0,00	0,00	-1,97	-2,16	2	2	0,99	0,00	(at3g29030):ATEXPA5 (ARABIDOPSIS THALIANA EXPANSIN A5)
At5g56320	0,00	0,00	-1,94	-2,25	0	0	0,05	0,00	(at5g56320):ATEXPA14 (ARABIDOPSIS THALIANA EXPANSIN A14)
At3g45960	0,02	0,00	-1,83	-2,59	0	0	-0,02	0,00	(at3g45960):ATEXLA3 (ARABIDOPSIS THALIANA EXPANSIN-LIKE A3)
At4g17030	0,00	0,00	-1,82	-2,47	2	2	0,20	0,00	(at4g17030):ATEXLB1 (ARABIDOPSIS THALIANA EXPANSIN-LIKE B1)
At3g15370	0,56	#N/A	-1,72	#N/A			0,03	0,00	(at3g15370):ATEXPA12 (ARABIDOPSIS THALIANA EXPANSIN 12)
At3g45970	0,00	0,00	-1,62	-2,36	1	1	-0,50	0,00	(at3g45970):ATEXLA1 (ARABIDOPSIS THALIANA EXPANSIN-LIKE A1)
At2g03090	0,00	0,00	-1,62	-1,43	2	2	0,74	1,28	(at2g03090):ATEXPA15 (ARABIDOPSIS THALIANA EXPANSIN A15)
At2g18660	0,00	0,00	-1,62	-0,68	0	0	-0,02	2,96	(at2g18660):expansin family protein (EXPR3)
At2g37640	0,00	0,00	-1,18	-2,07	2	2	0,18	0,00	(at2g37640):ATEXPA3 (ARABIDOPSIS THALIANA EXPANSIN A3)
At5g02260	0,00	0,00	-1,17	-1,16	2	2	0,31	0,00	(at5g02260):ATEXPA9 (ARABIDOPSIS THALIANA EXPANSIN A9)
At4g28250	0,00	0,00	-0,98	-1,07	2	2	1,03	0,00	(at4g28250):ATEXPB3 (ARABIDOPSIS THALIANA EXPANSIN B3)
At3g03220	0,13	0,01	-0,23	-0,36		2	0,52	0,00	(at3g03220):ATEXPA13 (ARABIDOPSIS THALIANA EXPANSIN A13)
At3g55500	0,48	0,48	-0,17	-0,21			0,11	0,00	(at3g55500):ATEXPA16 (ARABIDOPSIS THALIANA EXPANSIN A16)
At1g26770	0,00	0,00	0,60	1,58	1	1	0,67	0,00	(at1g26770):ATEXPA10 (ARABIDOPSIS THALIANA EXPANSIN A10)
At4g38400	0,00	0,12	0,63	-0,30	2		-0,17	1,32	(at4g38400):ATEXLA2 (ARABIDOPSIS THALIANA EXPANSIN-LIKE A2)
At5g39260	0,09	0,35	0,97	0,79			0,04	0,00	(at5g39260):ATEXPA21 (ARABIDOPSIS THALIANA EXPANSIN A21)
At5g05290	0,30	0,17	1,09	1,08			-0,03	0,00	(at5g05290):ATEXPA2 (ARABIDOPSIS THALIANA EXPANSIN A2)
At1g65680	0,53	0,95	1,11	-0,14			-0,03	0,00	(at1g65680):ATEXPB2 (ARABIDOPSIS THALIANA EXPANSIN B2)
At4g30380	0,09	0,20	2,55	1,98			0,02	0,00	(at4g30380):expansin-related
At4g38210	0,00	0,00	4,59	4,65	0	0	0,01	0,00	(at4g38210):ATEXPA20 (ARABIDOPSIS THALIANA EXPANSIN A20)
At1g12560	0,00	0,00	8,06	9,03	0	0	-0,01	0,00	(at1g12560):ATEXPA7 (ARABIDOPSIS THALIANA EXPANSIN A7)
At1g62980	#N/A	0,75	#N/A	0,60			0,11	0,00	(at1g62980):ATEXPA18 (ARABIDOPSIS THALIANA EXPANSIN A18)
At2g45110	#N/A	#N/A	#N/A	#N/A			0,03	0,00	at2g45110: ATEXPB4 (ARABIDOPSIS THALIANA EXPANSIN B4)
At3g60570	#N/A	#N/A	#N/A	#N/A			-0,01	0,00	(at3g60570):ATEXPB5 (ARABIDOPSIS THALIANA EXPANSIN B5)
At5g39270	#N/A	#N/A	#N/A	#N/A			-0,04	0,00	at5g39270: ATEXPA22 (ARABIDOPSIS THALIANA EXPANSIN A22)
At5g39280	#N/A	#N/A	#N/A	#N/A			0,00	0,00	at5g39280: ATEXPA23 (ARABIDOPSIS THALIANA EXPANSIN A23)
At5g39310	#N/A	#N/A	#N/A	#N/A			-0,03	-2,54	ARABIDOPSIS THALIANA EXPANSIN A24

E

Gene	iTPS response						CRF	Other responses	Annotation
Code	4-h adj.p	6-h adj.p	4-h FC log_2	6-h FC log_2	4-h CRF score	6-h CRF score		oeTPS FC log_2	
At5g65730	0,00	0,00	-3,04	-3,03	1	1	-1,12	1,55	(at5g65730):xyloglucan:xyloglucosyl transferase, putative
At1g10550	0,00	0,00	-2,69	-3,87	2	2	0,24	0,00	(at1g10550):XTH33 (xyloglucan:xyloglucosyl transferase 33);
At1g32170	0,00	0,00	-2,67	-1,98	1	1	-0,70	0,00	(at1g32170):XTR4 (XYLOGLUCAN ENDOTRANSGLYCOSYLASE 4);
At5g57560	0,00	0,00	-1,81	-2,26	1	1	-1,65	1,07	(at5g57560):TCH4 (TOUCH 4); hydrolase, acting on glycosyl bonds
At1g11545	0,00	0,00	-1,80	-3,04	2	2	0,80	0,00	(at1g11545):xyloglucan:xyloglucosyl transferase, putative
At3g25050	0,56	#N/A	-1,72	#N/A			0,04	0,00	(at3g25050):XTH3 (XYLOGLUCAN ENDOTRANSGLUCOSYLASE/HYDROLASE 3)s
At4g25820	0,56	#N/A	-1,72	#N/A			-0,04	3,42	(at4g25820):XTR9 (XYLOGLUCAN ENDOTRANSGLYCOSYLASE 9);
At5g57550	0,00	0,00	-1,70	-1,99	1	1	-1,66	1,03	(at5g57550):XTR3 (XYLOGLUCAN ENDOTRANSGLYCOSYLASE 3); hydrolase,
At3g45970	0,00	0,00	-1,62	-2,36	1	1	-0,50	0,00	(at3g45970):ATEXLA1 (ARABIDOPSIS THALIANA EXPANSIN-LIKE A1)
At4g03210	0,00	0,00	-1,58	-2,15	0	0	0,07	-1,32	(at4g03210):XTH9 (XYLOGLUCAN ENDOTRANSGLUCOSYLASE/HYDROLASE 9); s
At4g25810	0,00	0,00	-1,33	-1,93	1	1	-0,36	2,31	(at4g25810):XTR6 (XYLOGLUCAN ENDOTRANSGLYCOSYLASE 6); hydrolase
At3g23730	0,00	0,00	-1,25	-1,59	2	2	0,30	0,00	(at3g23730):xyloglucan:xyloglucosyl transferase, putative /e
At1g14720	0,00	0,00	-1,20	-1,14	1	1	-0,14	0,00	(at1g14720):XTR2 (XYLOGLUCAN ENDOTRANSGLYCOSYLASE RELATED 2);
At2g01850	0,00	0,00	-1,03	-1,06	1	1	-0,74	0,00	(at2g01850):EXGT-A3 (endo-xyloglucan transferase A3); hydrolase,
At4g37800	0,00	0,00	-1,03	-1,64	2	2	0,82	0,00	(at4g37800):xyloglucan:xyloglucosyl transferase, putative
At5g48070	0,64	0,06	-0,85	-4,55			0,32	0,00	(at5g48070):ATXTH20 (XYLOGLUCAN ENDOTRANSGLUCOSYLASE/HYDROLASE 20);
At1g65310	0,22	0,00	-0,61	-2,26		2	0,21	2,75	(at1g65310):ATXTH17 (XYLOGLUCAN ENDOTRANSGLUCOSYLASE/HYDROLASE 17);
At4g18990	0,33	0,31	-0,58	-0,65			-0,09	0,00	(at4g18990):xyloglucan:xyloglucosyl transferase, putative /
At3g48580	0,34	0,04	-0,45	-1,18		0	0,02	0,00	(at3g48580):xyloglucan:xyloglucosyl transferase, putative /
At2g14620	0,17	0,12	-0,39	-0,38			-0,08	0,00	(at2g14620):xyloglucan:xyloglucosyl transferase, putative /
At2g06850	0,01	0,00	-0,36	-1,69	1	1	-0,15	0,00	(at2g06850):EXGT-A1 (ENDO-XYLOGLUCAN TRANSFERASE);
At4g14130	0,77	0,00	-0,13	-1,64		1	-0,57	-1,18	(at4g14130):XTR7 (XYLOGLUCAN ENDOTRANSGLYCOSYLASE 7);
At4g30280	0,93	0,00	0,05	-1,59		0	-0,02	3,03	(at4g30280):ATXTH18/(XYLOGLUCAN ENDOTRANSGLUCOSYLASE/HYDROLASE 18)
At2g36870	0,00	0,00	0,33	0,44	1	1	0,60	0,00	(at2g36870):xyloglucan:xyloglucosyl transferase, putative /
At5g13870	0,00	0,00	1,95	1,63	2	2	-0,16	0,00	(at5g13870):EXGT-A4 (ENDOXYLOGLUCAN TRANSFERASE A4);
At2g45110	#N/A	#N/A	#N/A	#N/A			0,03	0,00	at2g45110: ATEXPB4 (ARABIDOPSIS THALIANA EXPANSIN B4)
At4g13080	#N/A	#N/A	#N/A	#N/A			0,00	0,00	(at4g13080):xyloglucan:xyloglucosyl transferase, putative /
At4g13090	#N/A	#N/A	#N/A	#N/A			-0,01	2,10	(at4g13090):xyloglucan:xyloglucosyl transferase, putative /
At4g28850	#N/A	#N/A	#N/A	#N/A			0,02	0,00	(at4g28850):xyloglucan:xyloglucosyl transferase, putative /
At5g57530	#N/A	0,62	#N/A	1,41			0,26	0,00	(at5g57530):xyloglucan:xyloglucosyl transferase, putative /
At5g57540	#N/A	#N/A	#N/A	#N/A			0,28	-2,65	(at5g57540):xyloglucan:xyloglucosyl transferase, putative /

F

Gene ontology analysis of a robust set of Tre6P-regulated genes that show significant iTPS responses, were assigned to CRF group G_1 and were also scored as responsive to oeTPS by Zhang et al. (2009).

Shared up

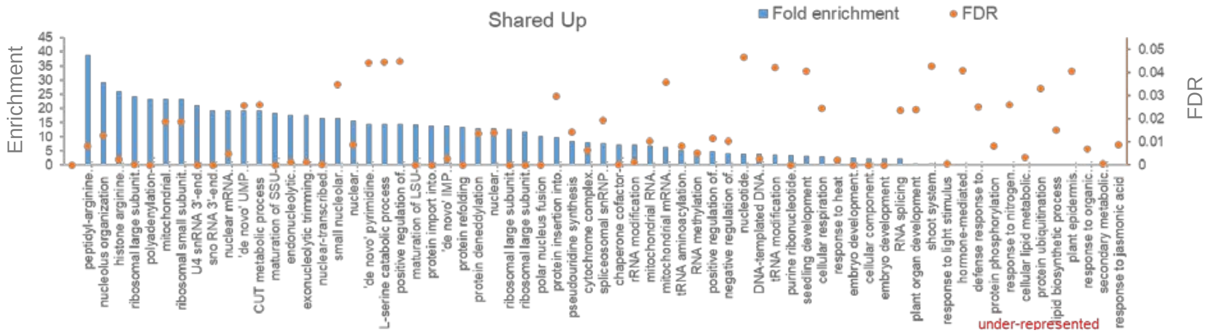

Shared down

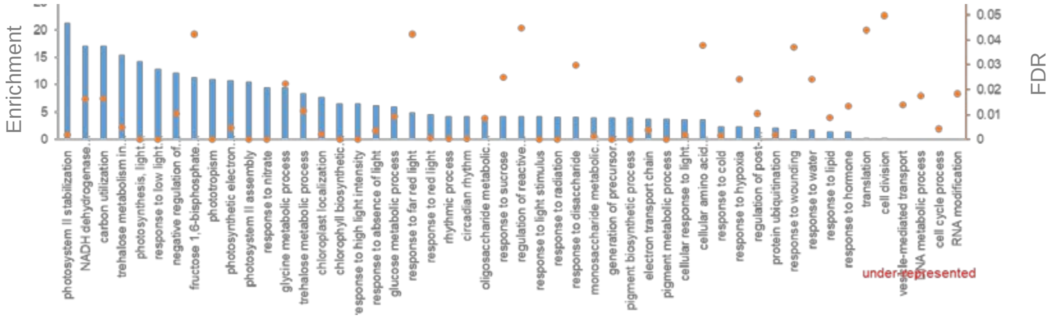

Supplemental Figure S13. Responses of transcripts encoding proteins involved in Tre6P metabolism. Responses are shown separately for the 4-h and 6-h iTPS response, assignment to CRF groups is denoted by shading (G_1 orange, G_2 blue, G_0 white, no significant change white), numbers represent log_2 FC values and the direction of change is depicted by font color (blue for increase and red for decrease). A blank denotes that the transcript did not show a significant change (FDR<0.05). The panel shows the response of 11 trehalose phosphate synthase (TPS) and 10 trehalose phosphate phosphatase (TPP) gene family members. Note that TPS1 is responsible for Tre6P synthesis, TPS2-4 are expressed only in seeds and are in part inactive, and TPS5-11 are so-called TPS class II proteins that have no catalytic activity (see Introduction). FC is fold change, CRF is carbon response factor, iTPS is the response to induced expression of bacterial TPS.

		iTPS response	
		4-h	6-h 6h
AT1G78580	TPS1	-1.1	-1.2
AT1G16980	TPS2		
AT1G17000	TPS3		
AT4G27550	TPS4		
AT4G17770	TPS5	-0.9	-0.6
AT1G68020	TPS6	-0.4	-0.4
AT1G06410	TPS7		
AT1G70290	TPS8	-1.5	-1.1
AT1G23870	TPS9	-1.3	-1.4
AT1G60140	TPS10	-1.2	-1.0
AT2G18700	TPS11	-1.9	-1.8
AT5G51460	TPPA	-2.0	-1.6
AT1G78090	TPPB	-3.0	-2.4
AT1G22210	TPPC		
AT1G35910	TPPD	-1.4	-1.2
AT2G22190	TPPE	-3.0	-2.3
AT4G12430	TPPF		
AT4G22590	TPPG	+0.8	+0.9
AT4G39770	TPPH	-3.9	-2.5
AT5G10100	TPPI		
AT5G65140	TPPJ	+0.7	+0.5

CRF group 1
CRF group 2
CRF group 0
No significant change

Supplemental Figure S14. Responses of transcripts encoding SnRK1 subunits, or implicated as downstream transcriptional targets of SnRK1 signaling.

(A) Responses of transcripts of genes encoding for SnRK1 structural subunits including catalytic subunits (SnRL1α1, SnRK1α2) and regulatory subunits (snf4 βγ, SnRK1β1, SnRK1β2, SnRK1β3).

(B) Responses of transcript for genes reported to be top 30 downstream targets of SnRK1 signaling (based on transient overexpression of SnRL1α1 in protoplasts, Baena-Gonzales *et al.*, 2007)

In panels A and B, responses are shown separately for the 4-h and 6-h iTPS response, assignment to CRF groups is denoted by shading (G_1 orange, G_2 blue, G_0 white, no significant change white), numbers represent log_2 FC values and the direction of change is depicted by font color (blue for increase and red for decrease).

(C) Comparison of the response to transient overexpression of SnRL1α1 in protoplast (tSnRL1α1 response) and the iTPS response of transcripts assigned to CRF groups G_2 and G_0, shown exemplarily for the iTPS 6h response (response for transcripts assigned to G_1 is shown in Figure 8B). (Supplemental to Figure 8 and to Supplemental Table S6).

CRF is carbon response factor, FC is fold change, FCR is false rate discovery, iTPS is the response to induced expression of bacterial TPS

See next pages for panels D-J

A.

		iTPS response 4-h	iTPS response 6-h
AT3G01090	SnRK1 α1	+0.2	+0.3
AT3G29160	SnRK1 α2	+0.2	+0.5
AT1G09020	snf4 βγ	+0.5	+0.6
AT5G21170	SnRK1 β1	-0.9	-1.1
AT4G16360	SnRK1 β2	+0.3	+0.3
AT2g28060	SnRK1 β3	+0.3	

CRF group 1
CRF group 2
CRF group 0
No significant change

B.

			iTPS response 4-h	iTPS response 6-h
Induced by transient overexpression of KIN10	AT3G06850	DIN3		
	AT3G47340	DIN6	+2.2	+0.8
	AT5G20250	DIN10	-1.0	-1.8
	AT3G15450	SEN5		-1.0
	AT5G28770	bZIP63	-1.5	-1.6
	AT2G33830	AXP	-1.5	-4.1
	AT4G35770	DIN1		-2.3
	AT5G49450	bZIP1	-1.6	-1.9
	AT5G18630	LIP	-1.3	-0.8
	AT4G15530	PPDK		-1.2
	AT1G12240	VIn2	-2.4	-2.8
	AT1G70290	TPS8	-1.5	-1.1
	AT1G12780	UDG	-1.9	-2.0
	AT5G22920	PGPD14	-1.2	-2.3
repressed by transient overexpression of KIN10	AT4G34590	bZIP11	-0.4	
	AT1G26770	EXP10	+0.6	+1.6
	AT3G50660	DWF4	-2.4	-1.8
	AT1G19640	JMT	+1.3	+0.5
	AT1G58360	AAP1	+0.4	+0.4
	AT2G36910	MDR1	-0.7	
	AT2G20610	SUR1	-1.3	-1.4
	AT3G09820	ADK1		
	AT3G44750	HDT1	+0.6	+0.5
	AT5G60690	IFL1	-0.9	-0.6
	AT3G28910	MYB30	-1.7	-1.2
	AT1G56650	MYB75	+0.7	+1.5
	AT3G21500	DPS		
	AT3G28900	L34		
	AT1G15250	L37	+1.3	+1.6

C.

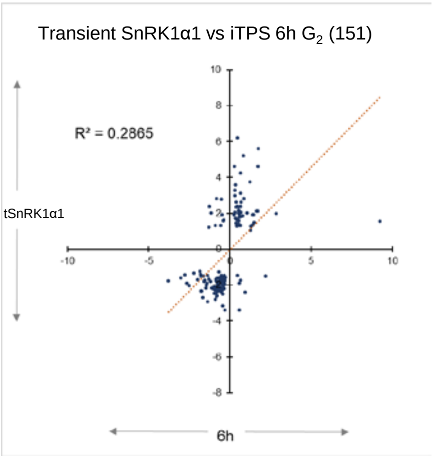

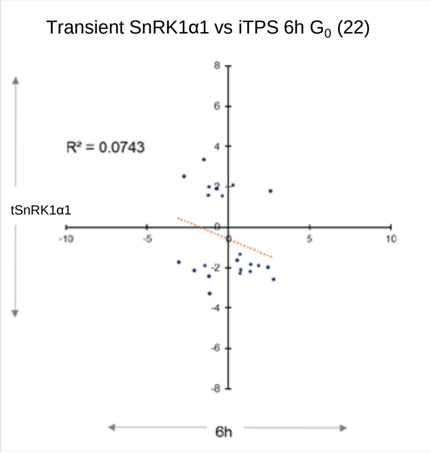

Supplemental Figure S14. Responses of transcripts encoding SnRK1 subunits, or implicated as downstream transcriptional targets of SnRK1 signaling (continued)

(C) Further comparison of the tSnRK1α1 response and the iTPS response. The analysis uses the top 100 (50 most strongly upregulated, 50 most strongly down-regulated) transcripts in the tSnRK1α1 dataset. The left-hand panels show the regression between the published tSnRK1α1 response and the4-h (top) and 6-h (bottom) iTPS responses for all 100 transcripts. Of the 100 transcripts in the tSnRK1α1 dataset, 52 and 72 were retrieved in the 4-h and 6-h iTPS responses, respectively, after filtering at FDR<0.05. Regressions of the published tSnRK1α1 response against the iTPS responses of these transcripts are shown in the middle panels). The 100 transcripts were also filtered based on their iTPS response (FDR>0.05, $\log_2$FC ≥ 0.2) and compared with the CRF (Supplemental Figure S2) to assign transcript to the GRF group G_1 (i.e.., transcripts whose iTPS response qualitatively similar to their CRF, and is probably due to a direct effect of elevated Tre6P). A total of 52 and 49 transcripts in the filtered tSnRL1α1 response were assigned to G_1 in the 4-h and 6-h iTPS datasets, respectively. Regression plots against CRF are shown in the right hand panels..

(D) Regression plots of CRF against the tSnRK1a1 responses for genes assigned (following the procedure in panel C) to CRF groups G_2 and G_0. More information about these analyses and correlations between the tSnRL1α1 response and the iTPS response of transcripts assigned to CRF groups G_2 and G_O is provided in Supplemental Table S6.

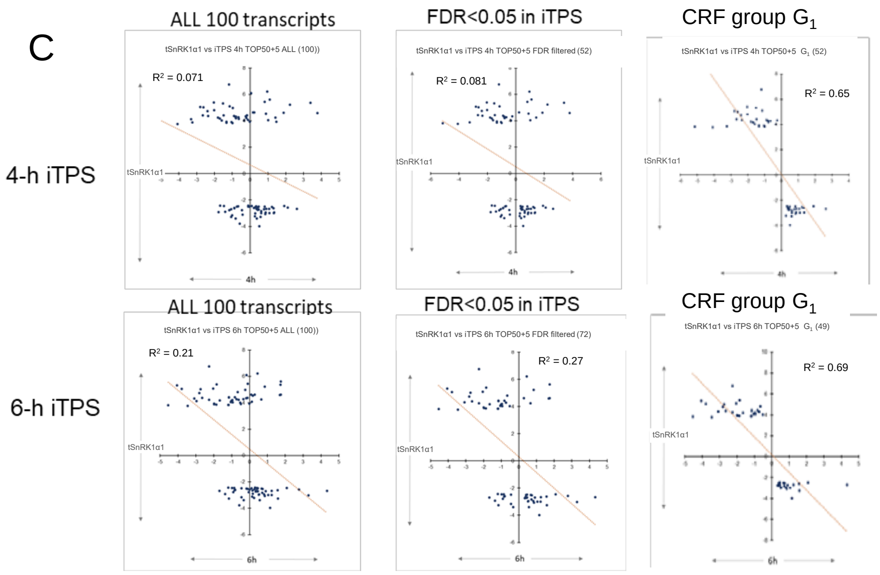

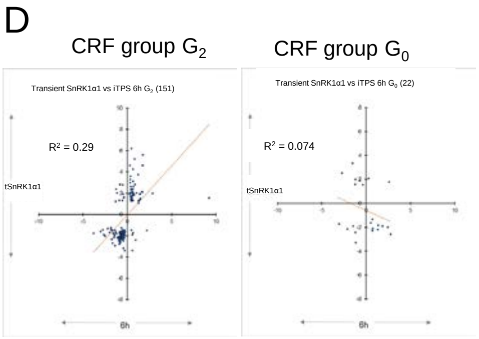

(E) The 1001 genes that were listed by Baena-Gonzalez et al. (2007) as downstream target of SnRK1 were divided into two sets to separate genes that were induced and repressed in the tSnRL1α1 response. In each set, the qualitative response in iTPS was determined, after filtering the iTPS response with an FDR<0.05 and FC<2 filter to allow comparison of two comparably filtered data sets. The table lists, for genes that were induced and repressed in the tSnRL1α1 response, the numbers of transcripts that increased and decreased in the iTPS response. The analysis was done separately for the 4-h and 6-h iTPS responses. The display also shows (in red font) for tSnRL1α1-induced and -repressed genes, what percentage showed a qualitatively opposite response in the tSnRL1α1 response and the iTPS response. A qualitatively opposite response is consistent with a mechanism in which SnRK1 signaling is inhibited by Tre6P. A qualitatively similar response is inconsistent with this mechanism, and consistent with a mechanism in which sugars or other factors regulate SnRK1 activity.

E

		**4-h iTPS response**		**6-h iTPS response**	
Gene set (tSnRK1α1, iTPS filter)		in tSnRK1a1 UP gene set	In tSnRK1α1 DOWN set	In tSnRK1α1 UP gene set	In tSnRK1α1 DOWN set
ALL FDR < 0.05 + FC2	UP in iTPS	**32**	45	**19**	65
	DOWN in iTPS	193	**58**	197	**35**
	**Qualitatively opposite response**	**85.8%**	**43.7%**	**83.6%**	**65%**

Supplemental Figure S14. Responses of transcripts encoding SnRK1 subunits, or implicated as downstream transcriptional targets of SnRK1 signaling (continued)

(F-H) Comparison of the iTPS response and the tSnRL1α1with the for three sets of genes related to metabolism, growth and signaling. These gene sets showed a iTPS response with a large CRF G_1 component indicating that many of them they are responses to elevated Tre6P. The analysis asks if the observed response in iTPS may be due to inhibition of SnRK1 by Tre6P. (G) Photosynthesis, (H) ribosomal proteins and ribosome assembly,(J) light signaling. In the following three panels, the left-hand display shows the response for all transcript assigned to the category, and the right-hand panel shows the response of those assigned to CRF group G_1.

F Comparison of the response of genes involved in **photosynthesis** to iTPS and to tSnRK1α1.

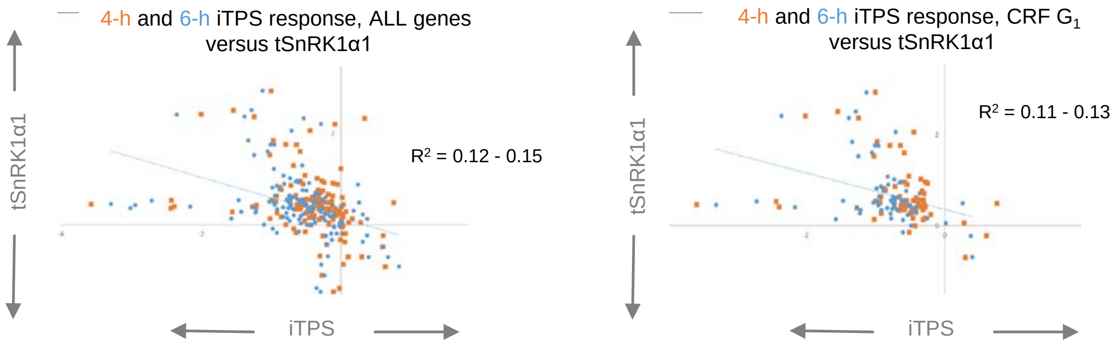

G Comparison of the response of genes involved in **light signaling** to iTPS and to tSnRK1α1. Genes are taken from Supplemental Figure S12D..

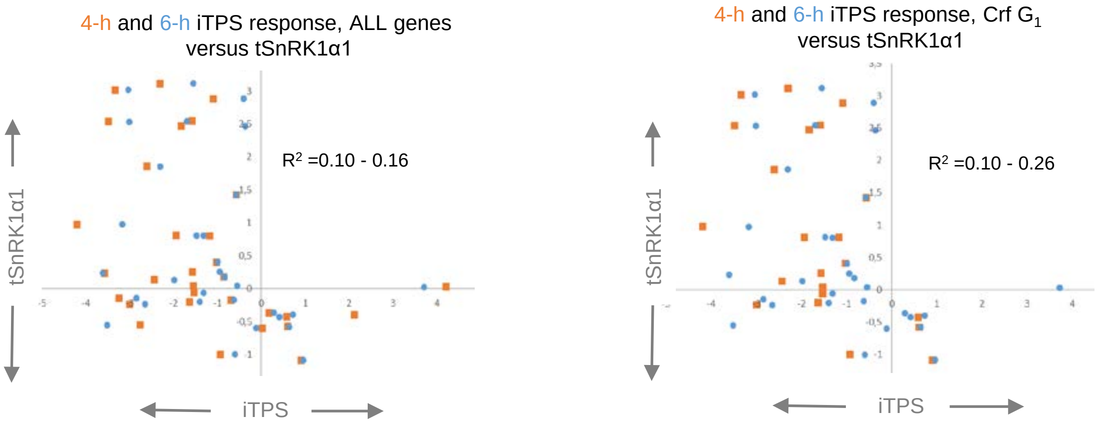

H Comparison of the response of genes encoding **cytosolic ribosomal proteins** to iTPS and to tSnRK1α1. For the responses of genes involved in ribosome assembly see Figure 8C.

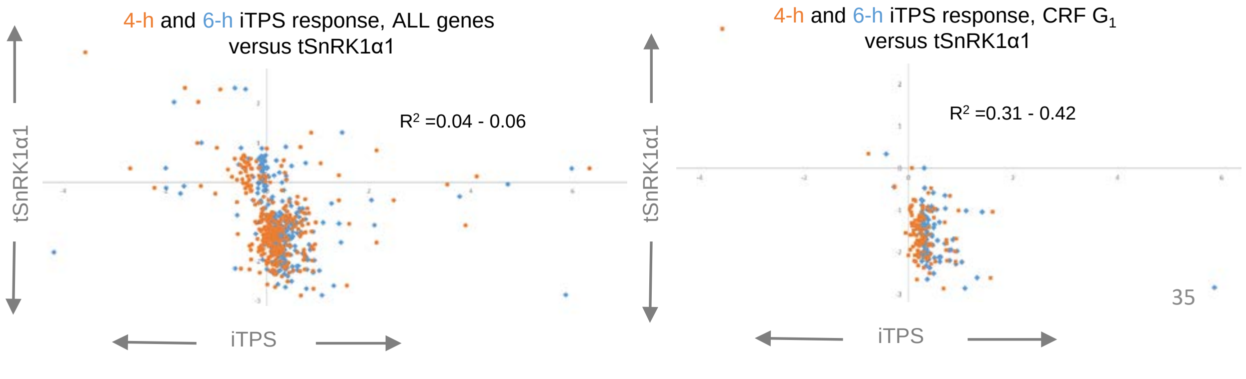

Supplemental Figure S14. Responses of transcripts encoding SnRK1 subunits, or implicated as downstream transcriptional targets of SnRK1 signaling (continued)

I Comparison of the tSnRK1α1 response and the CRF of genes annotated as ribosome assembly factors. Most of these genes are induced by sugar and, as reported for a subset by Baena-González et al. (2007), are repressed by tSnRK1a1. The responses of these genes to iTPS and oeTPS are shown in Figure 8C, revealing broad induction by elevated Tre6P.

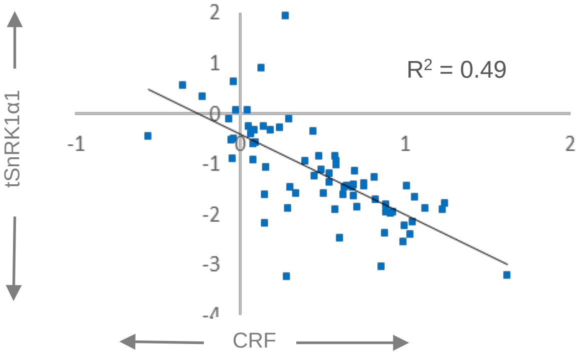

J

Gene		iTPS response						CRF	Other responses				Direction
Name	Code	iTPS 4-h adj.p	iTPS 6-h adj.p	29.2 4-h log2	29.2 6-h log2	iTPS 4-h CRF score	iTPS 6-h CRF score		oeTPS log2	SnRK1 TARGET GENES Log2	tSnRK1a1 log 2	oe bZIP11 log2	iTPS versus tSnRK1α1
TPS1	At1g78580	**0,00**	**0,00**	**-1,07**	**-1,19**	**2**	**2**	0,48	0,00		-0,70		
TPS2	At1g16980	0,36	0,55	-2,45	-1,76			0,01	0,00		0,36		
TPS3	At1g17000							0,04	0,00		0,65		
TPS4	At4g27550	0,37	0,15	-1,25	-3,62			0,07	0,00		0,64		
TPS5	At4g17770	0,00	0,00	-0,92	-0,57	2	2	1,24	3,00		-1,79		Same
TPS6	At1g68020	**0,00**	**0,00**	**-0,37**	**-0,39**	**1**	**1**	-0,62	-1,70		0,60		OPP
TPS7	At1g06410	0,59	0,83	-0,08	-0,04			-0,10	0,00		0,31		
TPS8	At1g70290	**0,00**	**0,00**	**-1,54**	**-1,08**	**1**	**1**	-2,30	-2,33	2,94	2,94		OPP
TPS9	At1g23870	**0,00**	**0,00**	**-1,30**	**-1,35**	**1**	**1**	-1,77	-2,49	3,32	3,32		OPP
TPS10	At1g60140	**0,00**	**0,00**	**-1,17**	**-1,03**	**1**	**1**	-1,63	-2,27	1,79	1,79		OPP
TPS11	At2g18700	**0,00**	**0,00**	**-1,88**	**-1,76**	**1**	**1**	-2,46	-3,44	2,21	2,21		OPP
TPPA	At5g51460	**0,00**	**0,00**	**-1,98**	**-1,56**	**2**	**2**	0,51	2,87		-0,22		
TPPB	At1g78090	**0,00**	**0,00**	**-2,99**	**-2,43**	**2**	**2**	0,37	0,00		-0,01		
TPPC	At1g22210	0,56	0,63	-1,72	-0,87			0,06	0,00		-0,49		
TPPD	At1g35910	**0,00**	**0,00**	**-1,41**	**-1,16**	**0**	**0**	0,06	0,00		0,07		
TPPE	At2g22190	**0,00**	**0,00**	**-3,04**	**-2,27**	**2**	**2**	0,67	0,00		-0,27		
TPPF	At4g12430	0,38	0,54	0,16	0,12			-0,09	-4,03		-0,17	1,26	
TPPG	At4g22590	**0,00**	**0,00**	**0,83**	**0,94**	**0**	**0**	0,09	-1,21		-1,21	1,77	
TPPH	At4g39770	**0,00**	**0,00**	**-3,93**	**-2,48**	**0**	**0**	0,02	-2,91		-0,29		
TPPJ	At5g65140	**0,00**	**0,00**	**0,74**	**0,52**	**0**	**0**	0,07	0,00		0,26		
TPPI	At5g10100	0,56	0,87	-0,39	0,11			0,06	0,00		-0,05		
TRE	At4g24040	**0,00**	**0,00**	**-2,14**	**-2,30**	**1**	**1**	-1,04	-2,44		1,31	3,43	OPP
AKIN10	At3g01090	**0,02**	**0,04**	**0,24**	**0,29**	**2**	**2**	-0,16	0,00	4,63	4,63		
AKIN11	At3g29160	**0,01**	**0,00**	**0,25**	**0,48**	**2**	**2**	-0,35	0,00		0,39	1,79	
SNF4/βγ	At1g09020	**0,00**	**0,00**	**0,48**	**0,58**	**1**	**1**	0,11	0,00		0,12		
SnRK1β1	At5g21170	**0,00**	**0,00**	**-0,92**	**-1,07**	**1**	**1**	-2,19	-2,25	3,23	3,23		OPP
SnRK1β2	At4g16360	**0,01**	**0,01**	**0,28**	**0,34**	**1**	**1**	0,18	1,10		-0,52		OPP

Comparison of the iTPS response with the tSnRL1α1 response for TPSs, Class II TPSs, TPPs and TRE (upper part of panel) and for SnRK1 subunits (lower part of panel). The display shows, from left to right,

- the gene
- the iTPS response: p-value, log_2 fold change and CRF assignment (genes assigned to CRF group G_1 are shaded in light orange). Significant changes in the iTPS response are highlighted in bold font.
- the CRF value (green shading, see Supplemental Figure S2).
- the response to constitutive overexpression of bacterial TPS (oeTPS, from Zhang et al., 2009)
- the response to transient overexpression of SnRK1α1 (t SnRK1α1, two columns, the first showing changes called significant and the second then log_2 FC for all, from Baena-Gonzalez et al., 2007).
- the response to constitutive overexpression of bZIP11 (oebZIP11, from Ma et al., 2011).

The right-hand column compares, for genes assigned to CRF group G_1 in the iTPS response, whether the direction of change in the iTPS response an the tSnRK1α1 response was in the same direction ('same') or in opposite directions ('OPP' , shaded orange). An opposite response is consistent with it being due to Tre6P-inhibition of SnRK1.

Supplemental Figure S15. TORC subunits, higher level regulators of ribosome biogenesis, other TORC targets including ABA receptors, and changes in global transcript abundance after inhibiting TORC.

This Supplemental Figure investigates the possible interaction of Tre6P in regulation of TOR expression and TOR signaling. See Supplemental Figure S14K for a description of the column headings.

(A) TOR subunits.

(B-E) Proteins identified as targets of TOR phosphorylation (Scarpin et al., 2020, Meng et al., 2022) divided into (B) protein kinases that are direct targets of TOR, (C) further genes implicated in ribosome biogenesis, (D) the family of ABA receptors that were highlighted as TOR phosphorylation targets in the analysis of Meng et al. (2022) and (E) further proteins with diverse functions, the upper block is from Meng et al. (2022), the middle block from Scarpin et al. (2020) and the lower block from Liao et al. (2022). The displays show the response in iTPS at 4h and 6h, assignment of the iTPS response to CRF groups, the CRF value (defined as in Supplemental Figure S1) and the response to constitutive overexpression of TPS (oeTPS, Zhang et al., 2009) and transient overexpression of SnRL1α1 in protoplasts (tSnRL1α1, Baena-Gonzalez et al., 2007).

Significant changes in the iTPS response are shown with bold font. Assignment to CRF groups is performed only for significant responses and only CRF1 assignments are shaded. No entry for oeTPS or tSnRL1α1 indicates the response did not pass the filter in the publication.

CRF is carbon response factor, FC is fold change, iTPS is the response to induced expression of TPS, oeTPS is the response to constitutive overexpression of TPS, TORC is TARGET OF RIFAMYCIN COMPLEX, AZD is AZD-8055, 5-{2,4-Bis [(3 S)-3-methylmorpholin-4-yl]pyrido [2,3-d]pyrimidin-7-yl}-2-methoxyphenyl)methanol, an inhibitor of TORC; ABA is abscisic acid

A

Gene		iTPS response						CRF	Other responses	
Name	Code	4-h adj. p	6-h adj. p	**4-h FC log_2**	**6-h FC log_2**	4-h CRF score	6-h CRF score		oeTPS FC log_2	tSnRK1α1 FC Log_2
TOR	At1g50030	0,75	0,55	-0,06	0,15			-0,17	0,00	0,51
RAPTOR2	At5g01770	**0,00**	0,36	**-0,45**	0,19	2		0,18	0,00	-0,24
RAPTOR1	At3g08850	**0,01**	**0,02**	**0,37**	**0,45**	2	2	-0,14	0,00	0,44
LST1 / 2	At3g18140/ At	na	na	na	na			-0,16	na	na

B

Gene		iTPS response						CRF	Other responses	
Name	Code	4-h adj. p	6-h adj. p	**4-h FC log_2**	**6-h FC log_2**	4-h CRF score	6-h CRF score		oeTPS FC log_2	tSnRK1α1 FC Log_2
S6K1	At3g08720	0,11	**0,06**	0,19	**0,33**		2	-0,34	1,16	0,56
S6K2	At3g08730	0,24	0,31	-0,16	-0,15			-0,04		-0,51
LARP1a	At5g21160	**0,00**	**0,00**	**0,43**	**0,48**	2	2	-0,44		1,08
LARP1b	At5g66100	0,36	**0,00**	0,10	**0,36**		1	0,20		
LARP1c	At4g35890	**0,02**	**0,00**	**0,23**	**0,60**	1	1	0,33		
YAK1	At5g35980	0,11	0,01	0,21	**0,44**		0	-0,05		

C

Gene		iTPS response						CRF	Other responses	
Name	Code	4-h adj. p	6-h adj. p	**4-h FC log_2**	**6-h FC log_2**	4-h CRFs core	6-h CRF score		oeTPS FC log_2	tSnRK1α1 FC Log_2
NAP1;1	At4g26110	**0,00**	**0,06**	**0,34**	0,26	1		0,46		-1,37
RPS6	At4g31700	**0,01**	0,36	**0,26**	0,15	1		0,62	1,52	-2,23

Supplemental Figure S15. TORC subunits, higher levels regulators of ribosome biogenesis and other TORC targets including ABA receptors, and changes in global transcript abundance after inhibiting TORC (cont.)

D

The family of ABA receptors that were highlighted as TOR phosphorylation targets in the data set of Meng *et al*. (2022).

Gene		iTPS response						CRF	Other responses	
Name	Code	4-h adj.p	6-h adj.p	4-h FC log_2	6-h FC log_2	4-h Crf score	6-h Crf score		oeOTSA FC log_2	tSnRK1α1 FC Log_2
**PYL1**	At5g46790	**0.00**	**0.00**	**-2.26**	**-1.67**	**2**	**2**	0.30	0.00	0.33
**PYL2**	At2g26040	**0.00**	**0.00**	**-0.89**	**-1.45**	**0**	**0**	0.08	0.00	**1.03**
**PYL3**	At1g73000	0.20	0.62	-1.99	1.41			-0.03	0.00	**0.97**
**PYL4**	At2g38310	**0.00**	**0.00**	**-3.39**	**-2.79**	**2**	**2**	0.11	0.00	-0.32
**PYL5**	At5g05440	**0.00**	**0.00**	**-4.11**	**-3.56**	**1**	**1**	-0.53	0.00	**1.92**
**PYL6**	At2g40330	**0.00**	**0.00**	**-3.94**	**-3.84**	**0**	**0**	0.04	0.00	0.51
**PYL7**	At4g01026	**0.00**	**0.00**	**-1.49**	**-1.26**	**1**	**1**	-0.95	**-3.24**	**2.57**
**PYL8**	At5g53160	**0.00**	**0.00**	**-1.61**	**-1.39**	**1**	**1**	-0.73	**-2.55**	**0.58**
**PYR9**	At1g01360	**0.01**	**0.01**	**-0.38**	**-0.49**	**1**	**1**	-0.60	**-1.35**	**0.93**
**PYR1**	At4g17870	**0.00**	**0.00**	**-1.36**	**-1.96**	**0**	**0**	-0.05	0.00	0.44

E

Further proteins that were highlighted as downstream TOR phosphorylation targets in the data sets of Meng *et al.* (2022) and Brunkold *et al*. (2020), as well as the autophagysome assembly factor ATG13a that is considered a target of TOR (Liao *et al*., 2022)

Gene		iTPS response						CRF	Other responses		Annotation
Name	Code	4-h adj.p	6-h adj.p	4-h FC log_2	6-h FC log_2	4-h CRF score	6-h CRFscore		oeTPS FC log_2	tSnRK1α1 FC Log_2	
E2Fa	At2g36010	**0,03**	0,38	**0,32**	0,15	1		0,41	0,00	0,32	E2F TRANSCRIPTION FACTOR-3; cell cycle, components of cyclin D/retinoblastoma/E2F pathway
E2Fb	At5g22220	0,31	0,31	0,17	0,20			-0,24	0,00	1,36	E2F1; transcription factor, component of cyclin D/retinoblastoma/E2F pathway. RBR1 proteins
ATG1B	At3g53930	0,66	0,34	-0,07	-0,16			-0,24	0,00	0,73	Protein kinase superfamily protein
ATG13	At3g49590	**0,01**	**0,00**	**0,26**	**0,48**	2	2	-0,59	0,00	2,40	Autophagy protein
EIN2	At5g03280	0,08	**0,00**	0,26	**0,66**		0	0,00	0,00	-0,08	ETHYLENE INSENSITIVE 2; transporter, ethylene signal transduction acting downstream of CTR1
PIN2	At5g57090	**0,00**	**0,03**	**1,89**	**0,98**	1	1	0,10	1,61	0,74	EIR1 (ETHYLENE INSENSITIVE ROOT 1); auxin:hydrogen symporter/ transporter
S40-7	At3g15040	0,32	0,11	-0,13	-0,24			-0,21	0,00	0,47	Senescence regulator
hyp. prot.	At3g50370	0,24	0,09	0,19	0,36			0,02	0,00	-0,22	similar to cupin family protein [Arabidopsis thaliana] (TAIR:AT2G18540.1)
eIF2B-δ1	At2g44070	0,10	**0,02**	-2,43	**3,24**		1	0,26	na	na	eukaryotic translation initiation factor 2B family protein / eIF-2B family protein
TAP46	At5g53000	**0,00**	**0,02**	**0,37**	**0,30**	1	1	0,18	0,00	0,34	PP2A-associated protein with a possible function in the chilling response
AML5	At1g29400	0,20	**0,00**	-0,13	**0,51**		2	-0,50	0,00	0,26	member of the *mei2-like* gene family, encoding RNA -binding proteins
eS6b	At5g10360	**0,00**	0,10	**0,33**	0,25	1		0,19	#N/A	-1,72	EMB3010 | EMB3010 (embryo defective 3010); structural constituent of ribosome | chr5
eIF4B1	At3g26400	**0,02**	0,17	**0,25**	0,16	1		0,17	#N/A	#N/A	EIF4B1 | EIF4B1; translation initiation factor | chr3
eIF4B2	At1g13020	**0,05**	**0,01**	**0,20**	**0,36**	1	1	0,31	#N/A	#N/A	eIF4B, mRNA unwinding factor
eIF3m	At3g0220(	**0,00**	**0,00**	**0,58**	**0,488**	2	2	-0,13	#N/A	#N/A	eIF3 mRNA-to-PIC binding complex.eIF3m component
VLN2	At2g41740	**0,00**	0,24	**-0,34**	-0,19	2		0,12	#N/A	#N/A	VILLIN 2; actin binding | chr2
VLN3	At3g57410	**0,00**	**0,00**	**-0,75**	**-0,78**	1	1	-0,25	#N/A	#N/A	VILLIN 3; actin binding | chr3
TOPLESS	At1g15750	**0,00**	**0,00**	**-1,14**	**-0,90**	1	1	-0,25	#N/A	#N/A	TOPLESS, transcription repressor | chr1
CBE1	At4g01290	0,25	**0,04**	0,16	**0,37**		9	0,05	#N/A	#N/A	CONSERVED BINDING OF EIF4E
PUX5	At4g15410	0,06	0,14	0,24	0,21			0,36	#N/A	#N/A	plant ubiquitin regulatory X domain-containing protein
ATG13a	At3g49590	**0,01**	**0,00**	**0,26**	**0,48**	2	2	-0,59	#N/A	2,4	Vesicle trafficking.autophagosome formation, ATG13 accessory component

Supplemental Figure S15. TORC subunits, higher levels regulators of ribosome biogenesis and other TORC targets including ABA receptors, and changes in global transcript abundance after inhibiting TORC (cont.)

F Comparison of the response of transcript abundance (FC on a $\log_2$ scale) to inhibition of TORC by application of AZD8055 (Dong et al., 2015) or by estradiol-induction of ami-*tor* (Caldana et al., 2012) .

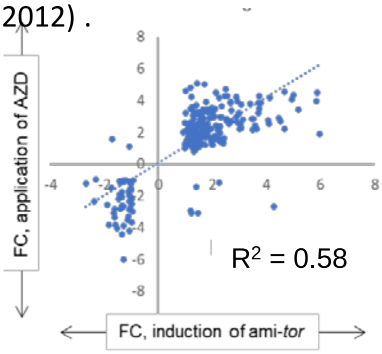

Both experiments were carried out on seedlings growing on nutrient agar supplemented with 1% sucrose. The study of Caldana et al (2015) induced ami-*tor* at 14 days and sampled at 14 and 20 days for transcript analysis, and reported 515 (359 up- and 156 down-regulated) that passed a filter of FC >2 in at least two of the three lines; for further analyses we averaged the response of all three lines. The study of Dong et al (105) applied 2µM AZD8055 after 10 days and harvested seedlings 24 h late and reported 24K transcripts of which 2984 that showed significant changes and are plotted.

G Comparison of the CRF (see Supplemental Figure S2) with the response to inhibition of TORC by AZD8055 application (left-hand display) or induced ami-*tor* (middle display). Plots show FC compared to control (filters applied as in panel F). The right-hand display compares the CRF with the response of ~1000 transcripts that showed significant changes after transient overexpression of SnRK1α1

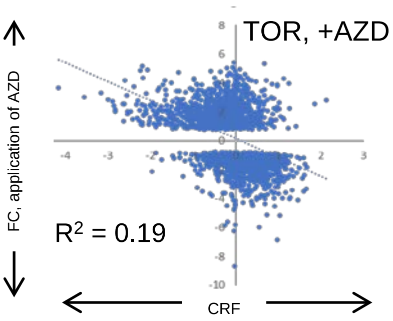

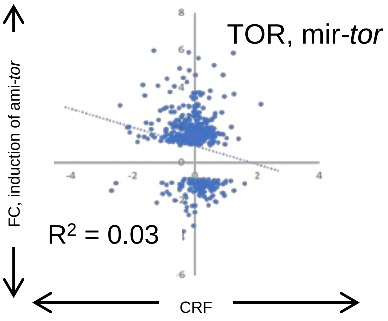

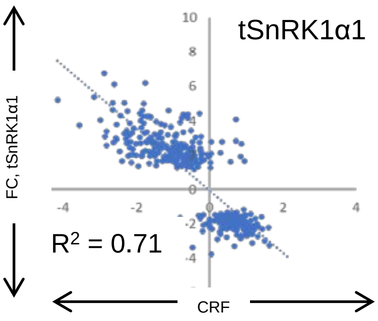

H Comparison of the response to tSnRK1α1 and the response to inhibition of TORC by AZD8055 (right hand display) and ami-*tor* (red = called significant in both treatments, blue = significant in ami-*tor* and all in tSnRK1α1)

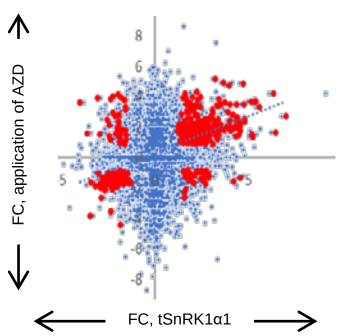

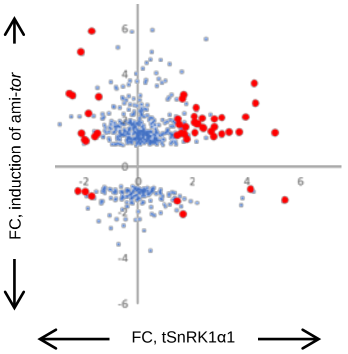

I Comparison of the overall response 4h after induction of TPS (iTPS 4h) with the response to inhibition of TORC by AZD8055 (left-hand display) or induced ami-*tor* (middle display). The plots show the FC compared to control. Filters were applied as in panel F. Similar results were obtained in an analysis with the iTPS 6h data set (not shown). For comparison, the right-hand display show compares the iTPS 4h response with the response to tSnRK1α1 (copy of Figure 8A)

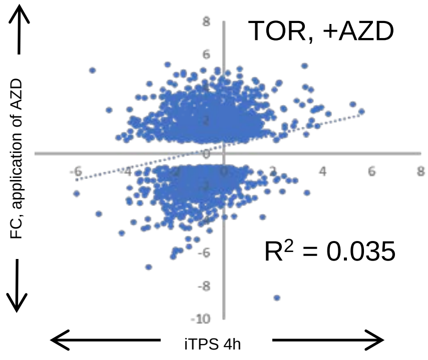

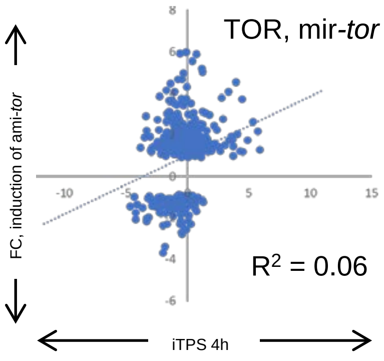

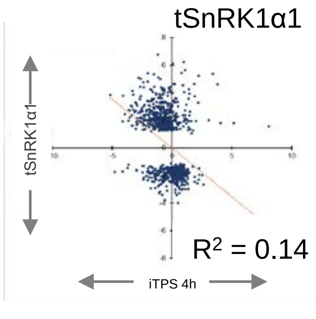

Copy of Figure 8A

Supplemental Figure S15. TORC subunits, higher levels regulators of ribosome biogenesis and other TORC targets including ABA receptors, and changes in global transcript abundance after inhibiting TOR (cont.)

J Deconvolution of the iTPS response into sets of transcripts that respond directly (CRF group G_1) and indirectly (CRF groups, G_2, G_0). For each set of transcripts, the plots compare the response 4h after induction of TPS with the response to inhibition of TORC by AZD8055 application (left-hand display) or induced ami-*tor* (middle display). The plots show the FC compared to control treatments. Filters were applied as in panel F. Similar results were obtained in an analysis with the iTPS 6h data set (not shown). For comparison, analyses of the relation to transient overexpression of SnRK1α1 are shown in the inlays on the right of the display (copies of previous figures as stated)

CRF group G_1

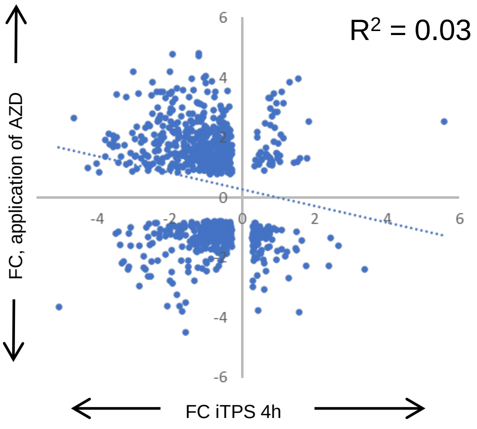

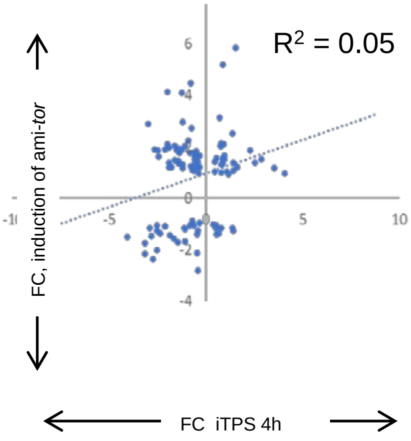

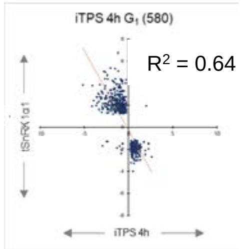

Copy of Figure 8B

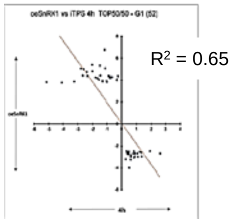

Copy of Supplemental Figure 14C

CRF group G_2

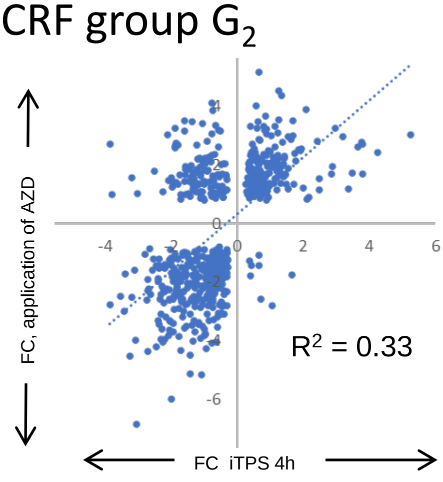

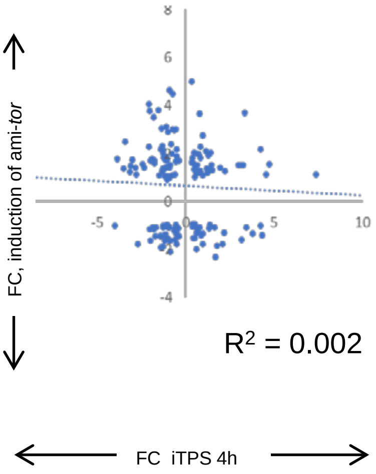

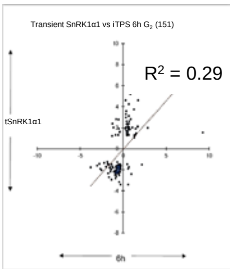

Copy of Supplemental Figure 14D

CRF group G_0

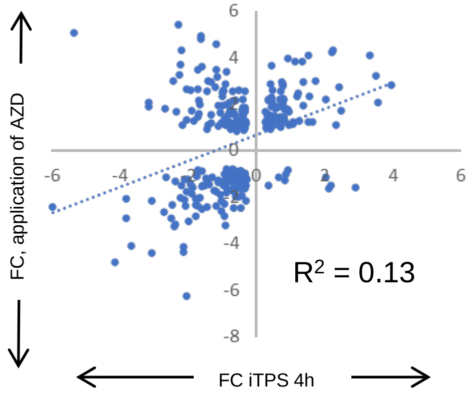

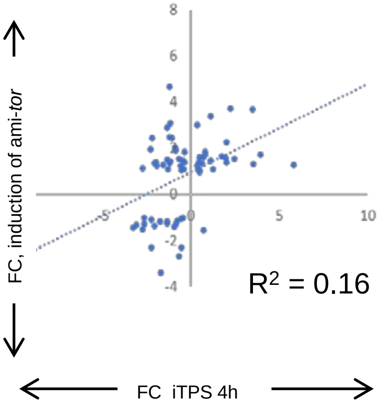

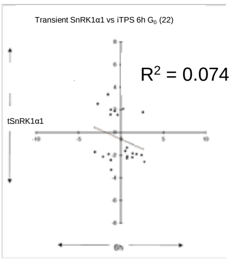

Copy of Supplemental Figure 14D

Supplemental Figure S15. TORC subunits, higher levels regulators of ribosome biogenesis and other TORC targets including ABA receptors, and changes in global transcript abundance after inhibiting TORC (cont.)

K Comparison of the tSNrK1α1 response and response to inhibition of TOR, focusing on the iTPS G_1 gene set, which respond directly to elevated Tre6P probably acting via inhibition of SnRK1. The left-hand plot shows the relationship between the AZD8055-TORC response and the tSNrK1α1 response. The right-hand plot shows the relationship between the ami-*tor* response and the tSNrK1α1 response for all genes in the iTPS data set /blue symbols) and for genes that passed the >2 FC filter (red symbols).

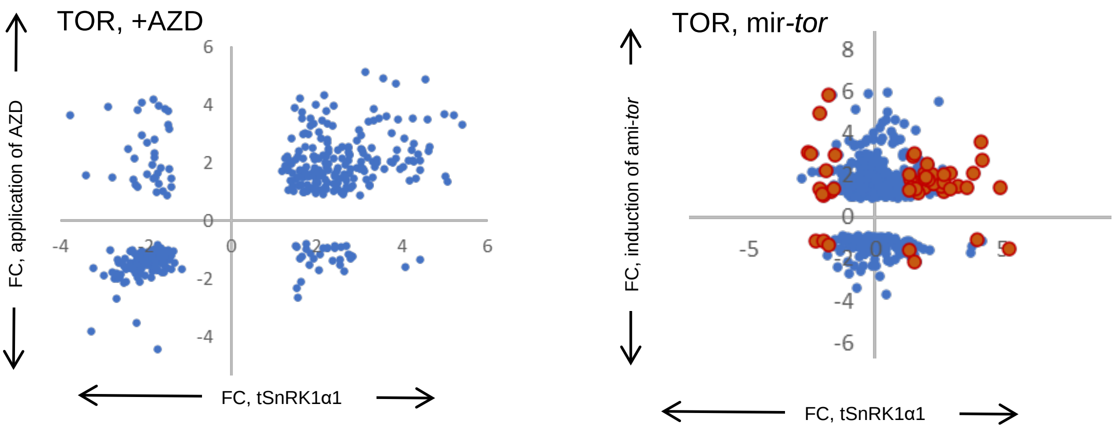

L Comparison of the iTPS response and the response to TORC inhibition for gnes in the MapMan BINs 'Ribosome biogenesis' and 'Photosynthesis' To maximize the number of data points available, the analysis was performed with AZD8055-treatment data of Dong et al. (2015) and used all 24K reported transcripts

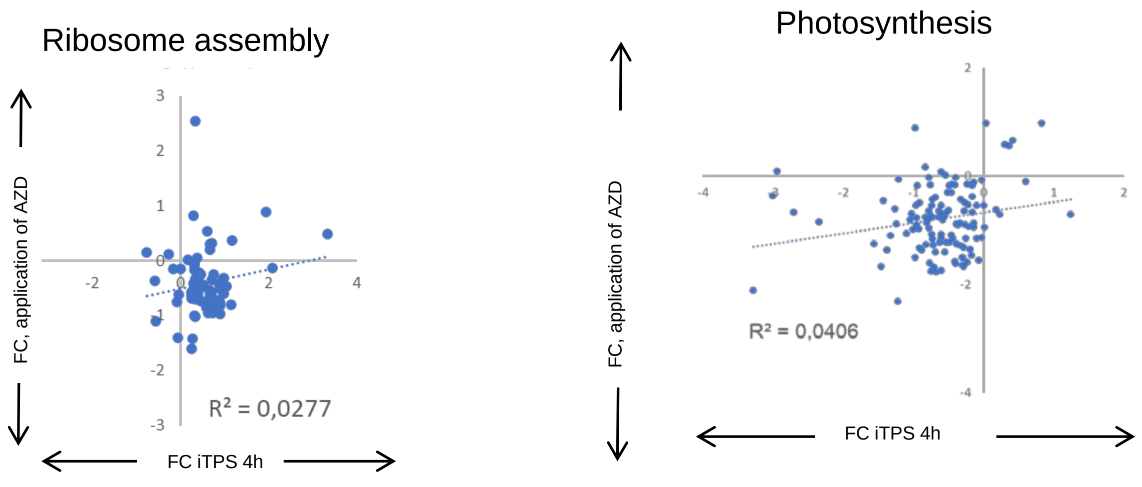

Supplemental Figure S16. FCS-Like Zinc finger (FLZ) family proteins.

The annotation is taken from Jamsheer et al. (2015). All of these proteins are annotated in TAIR11 as ‘SnRK1 metabolic regulator system. FLZ SnRK1-interacting factors’.

They have been reported to respond differently to sugar addition and other treatments like hormone addition and environmental stress (Nietzsche et al., 2014; Jamsheer et al., 2015). The responsiveness to sugar as reported by Jamsheer and Laxmi (2015) is given in the left-hand column, with genes allocated to three sets that that were defined in this publication: set 1 (repressed by >10fold), set 2 (repressed by 2-10-fold) or set 3 (inconsistent responses or are induced). FLZ6 (like FLZ10) was subsequently found to be starvation-induced (Jamsheer *et a*l., 2018).

The display shows the 4-h and 6-h iTPS response, the assignment of the iTPS response to CRF groups, the CRF value (defined as in Supplemental Figure 1 and the response to constitutive overexpression of TPS (oeTPS, from Zhang et al., 2009)) and transient overexpression of SnRK1α1 in protoplasts (tSnRL1α1, from Baena-Gonzalez et al., 2007). Significant changes in the iTPS response are shown with bold font. Assignment to CRF groups is performed only for significant responses and only CRF G_1 assignments are shaded.
No entry for oeTPS or tSnRL1α1 indicates that the response did not pass the filter in the original publication.

Significant changes in the iTPS response are shown with bold font. Assignment to CRF groups is performed only for significant responses, and only CRF G_1 assignments are shaded

Many FLZ family members are significantly repressed in the iTPS response including four of five in set 1 (FLZ1, FLZ5, FLZ8, FLZ14), two of four in set 2 (FLZ3, FLZ15), and four of five in set 3 (FLZ6, FLZ9, FLZ13, FLZ17) of Jamsheer and Laxmi (2015). One was induced in set 2 (FLZ10) of Jamsheer and Laxmi (2015) However, internal analysis in the iTPS data set and especially comparison of the iTPS response with the Crf indicated that most of the responses were indirect (Crf groups 2 or 0) and therefore unlikely to be due to Tre6P signalling. Those assigned to a possible Tre6P-response ((CRF group G1) were FLZ9, FLZ13 and FLZ19, all of which had been previously classed as non-sugar responsive by Jamsheer and Laxmi (2015).

Of the sugar-responsive FLZs, experimental evidence has been presented for an inhibitory effect on SnRK1 for FLZ3 (Bortlik et al., 2022), FLZ8 (Jamsheer et al., 2022) and FLZ6 and FLZ10 (Jamsheer et al., 2018). All of these are inhibited in the iTPS response but all are assigned in CRF groups G_2, indicating that the response is indirect, probably due to the decrease in sugars.

FLZ4 (At4g65040), FLZ7 (At4g39795) and FLZ18 (At1g53903) were not present in the filtered iTPS response dataset, and FLZ16 was not scored in the iTPS response.

CRF is carbon response factor, FC is fold change, FCR is false rate discovery, iTPS is the response to induced expression of bacterial TPS

	Gene		iTPS response						CRF	Other responses		Ontology and annnotation
Grouping in Jamsheer and Laxmi (2015)	Name	Code	4-h adj.p	6-h adj.p	4-h FC log_2	6-h FC log_2	4-h CRF score	6-h CRF score		oeTPS FC log_2	tSnRK1α1 FC Log_2	
1	FLZ1	At5g47060	**0,00**	0,91	**-0,55**	0,02	**2**		1,23	**3,10**	#N/A	Multi-process regulation.SnRK1 metabolic regulator system.FLZ SnRK1-interacting factor
1	FLZ2	At4g17670	0,14	0,81	-0,27	-0,04			1,19	#N/A	#N/A	Multi-process regulation.SnRK1 metabolic regulator system.FLZ SnRK1-interacting factor
1	FLZ5	At1g22160	**0,00**	**0,00**	**-1,16**	**-1,20**	**2**	**2**	0,21	#N/A	#N/A	Multi-process regulation.SnRK1 metabolic regulator system.FLZ SnRK1-interacting factor
1	FLZ8	At3g22550	**0,00**	**0,00**	**-1,50**	**-0,75**	**2**	**2**	0,71	#N/A	#N/A	Multi-process regulation.SnRK1 metabolic regulator system.FLZ SnRK1-interacting factor
1	FLZ14	At5g20700	**0,00**	**0,00**	**-1,16**	**-0,90**	**2**	**2**	0,84	#N/A	-2,78	Multi-process regulation.SnRK1 metabolic regulator system.FLZ SnRK1-interacting factor
2	FLZ3	At2g44670	**0,00**	**0,00**	**-1,30**	**-0,64**	**2**	**2**	0,29	#N/A	#N/A	Multi-process regulation.SnRK1 metabolic regulator system.FLZ SnRK1-interacting factor
2	FLZ10	At5g11460	**0,00**	**0,00**	**0,47**	**0,96**	**0**	**0**	0,09	#N/A	#N/A	Multi-process regulation.SnRK1 metabolic regulator system.FLZ SnRK1-interacting factor
2	FLZ11	At2g25690	0,19	0,17	0,23	0,26			0,23	#N/A	#N/A	Multi-process regulation.SnRK1 metabolic regulator system.FLZ SnRK1-interacting factor
2	FLZ15	At5g49120	**0,00**	**0,00**	**-2,01**	**-1,29**	**0**	**0**	0,03	#N/A	#N/A	Multi-process regulation.SnRK1 metabolic regulator system.FLZ SnRK1-interacting factor
2	FLZ16	At3g63230	#N/A	#N/A	#N/A	#N/A			0,01	#N/A	#N/A	Multi-process regulation.SnRK1 metabolic regulator system.FLZ SnRK1-interacting factor
3	FLZ6	At1g78020	**0,00**	**0,00**	**-1,53**	**-0,72**	**2**	**2**	0,28	#N/A	#N/A	Multi-process regulation.SnRK1 metabolic regulator system.FLZ SnRK1-interacting factor
3	FLZ9	At3g63210	**0,00**	**0,00**	**-3,52**	**-3,00**	1	1	-0,53	#N/A	#N/A	Multi-process regulation.SnRK1 metabolic regulator system.FLZ SnRK1-interacting factor
3	FLZ12	At1g19200	0,94	0,18	-0,03	-0,35			-0,01	#N/A	#N/A	Multi-process regulation.SnRK1 metabolic regulator system.FLZ SnRK1-interacting factor
3	FLZ13	At1g74940	0,59	**0,00**	-0,09	**-0,40**		**1**	-0,17	#N/A	#N/A	Multi-process regulation.SnRK1 metabolic regulator system.FLZ SnRK1-interacting factor
3	FLZ17	At1g53885	**0,01**	**0,00**	**-0,93**	**-1,39**	**1**	**1**	-1,49	#N/A	#N/A	Multi-process regulation.SnRK1 metabolic regulator system.FLZ SnRK1-interacting factor

Supplemental Figure S17. Comparison of iTPS with constitutive overexpression of zBIP11.

Sucrose acts at an uORF to inhibit translation of bZIP11, and falling sucrose allows synthesis of this S_1 type bZIP protein that then interacts with C-type bZIPs to transcriptionally activate starvation responses and inhibit growth (Hanson et al., 2009; Ma et al., 2011, Dröge-Laser and Weiste, 2018).

Ma *et al.* (2011) reported compared to wild-type Arabidopsis, 232 transcripts that showed a significant change and passed a FC > 2 filter in lines with constitutive overexpression of *bZIP11* (oebZIP11). This published response to oebZIP11 was compared with the iTPS response.

CRF is carbon response factor, FC is fold change, iTPS is response to induced expression of bacterial TPS, oeTPS is constitutive overexpression of TPS.

Panels A-C are below, Panels D-G are on the following pages

A

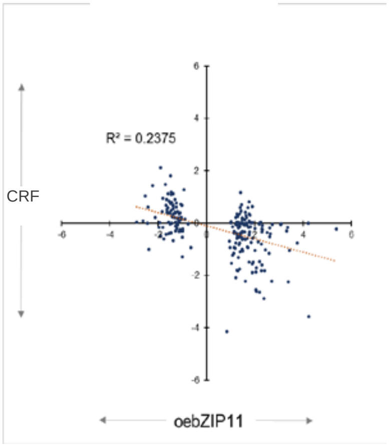

Relationship between the oebZIP11 response and the carbon response factor (CRF) (estimated for each gene from nine treatments in which sugar levels were changed (Supplemental Figure S2).

B

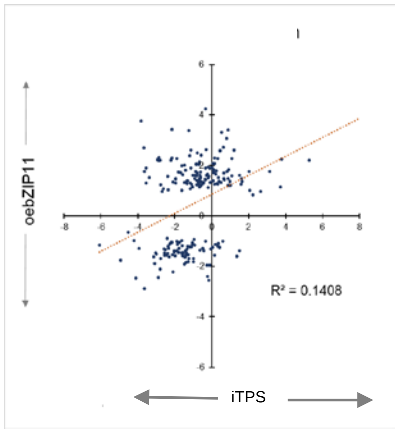

Relationship between the oebZIP11 response and the overall iTPS response. The iTPS data set was not filtered, in order to show not only the response of transcripts that changed but also of any that did not change. The iTPS response is the average of the 4-h and 6-h post-induction responses.

C **Relationship between oebZIP11 and the iTPS response after deconvolution to assign transcripts to CRF groups G_1, G_2 and G_0 (from left to right).** The iTPS response is the average of the 4-h and 6-h post-induction response. The number in brackets is the number of genes in each subset

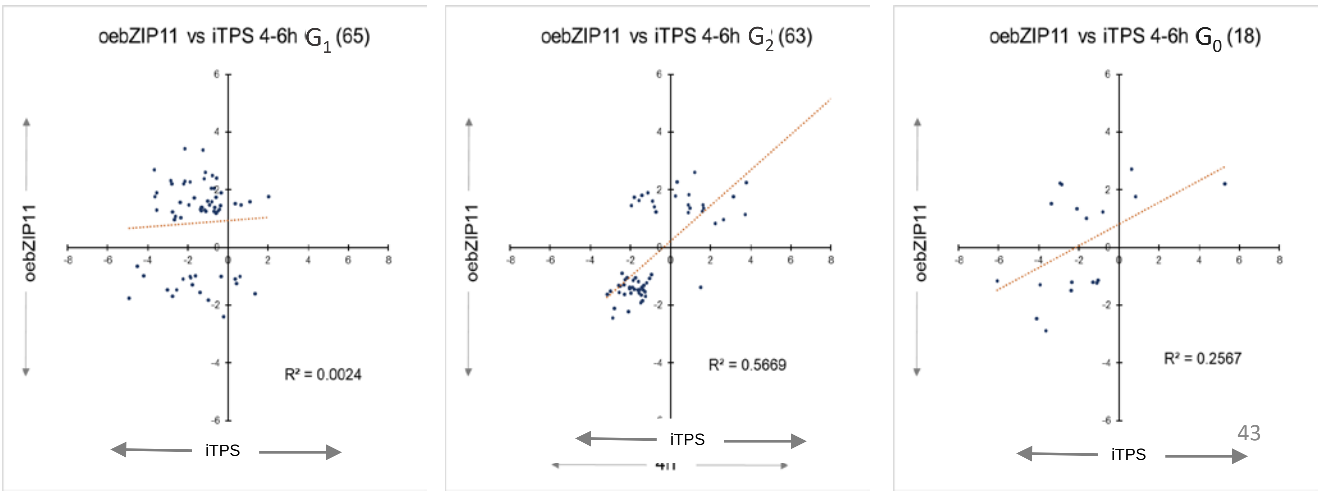

D **oebZIP11-responsive genes: comparison of the iTPS response and the response to constitutive oeTPS** (Zhang et al., 2009). The data set of Zhang et al. (2009) was filtered by the authors (FDR<0.05, FC>2) and contained 94 of the 232 genes that Ma et al. (2009) identified as oebZIP11-responsive. The upper panel shows the relationship between iTPS and oeTPS for all 94 shared genes, the middle panel shows the relationship between iTPS and oeTPS for the 43 genes that were assigned to CRF G_1 in the iTPS response, and the lower panel shows the relationship between iTPS and oeTPS for the 30 genes that were assigned to CRF G_2 in the iTPS response. The iTPS response is the average of the 4-h and 6-h post-induction iTPS responses.

.

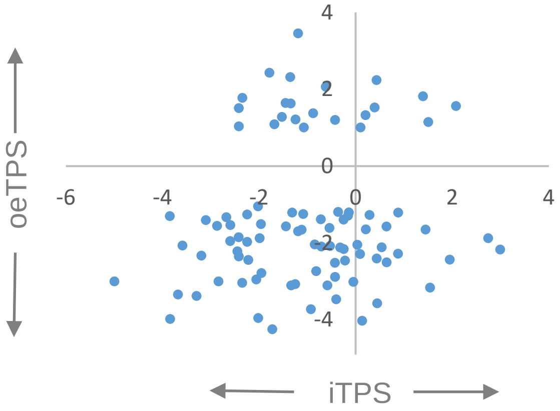

All genes in the constitutive oeTPS response

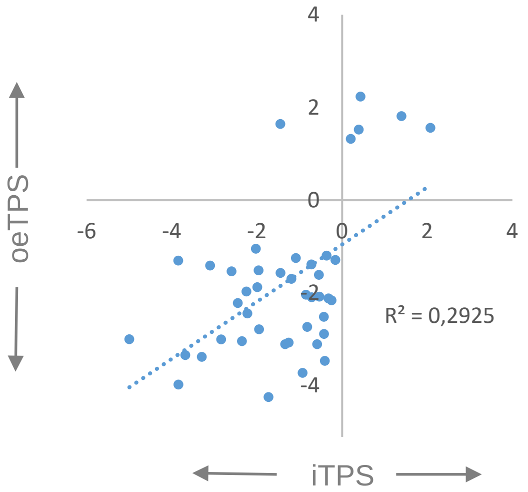

Genes assigned to CRF group G_1 in the iTPS response

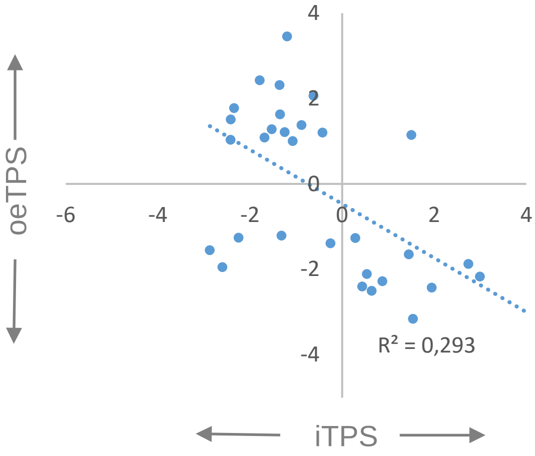

Genes assigned to CRF group G_2 in the iTPS response

Supplemental Figure S17. Comparison of iTPS with constitutive overexpression of zBIP11 (continued)

(E) **List of oebZIP1 responsive genes that are included in genes assigned to CRF group G_1 in the iTPS response.** The genes are separated into those whose transcripts show a qualitatively similar response in oebZIP11 and iTPS (upper part, blue shaded) and those that show an opposite response (lower part, pink-shaded). The display shows the 4-h and 6-h iTPS responses, assignment of the iTPS response to CRF groups, the CRF value (defined as in Supplemental Figure S2), the response to constitutive overexpression of TPS (oeTPS, from Zhang et al., 2009) and to transient overexpression of tSnRL1α1 in protoplasts (tSnRL1α1, from Baena-Gonzalez et al., 2007) and to oebZIP11. Significant changes in the iTPS response are shown with bold font. Assignment to CRF groups is performed only for significant responses and only CRF G_1 assignments are shaded. No entry for oeTPS or tSnRL1α1 indicates that the response did not pass the filter in the original publication

E

Gene		iTPS response						CRF	Other responses				Ontology and annnotation
Name	Code	4-h adj.p	6-h adj.p	**4-h FC log_2**	**6-h FC log_2**	4-h CRF score	6-h CRF score		oeTPS FC log_2	tSnRK1α1 FC Log_2	**oebZIP11**	**direction bZIP11 compared to iTPS**	
GAS6	At1g74670	0,00	0,00	-4,92	-5,07	1	1	-0,60	-3,00		-1,76	same	GA-STIMULATED 6 Cell wall protein downstream of RGL2, integrating GA, ABA, and Glc signaling
SWEET12	At5g23660	0,00	0,00	-4,53	-3,16	1	1	-0,93	-1,30		-0,66	same	SWEET 12, sucrose expoert from leaf
NPH3 protein	At3g19850	0,00	0,00	-4,19	-3,17	1	1	-0,31	-3,34		-0,98	same	Phototropic-responsive **NPH3** family protein
WRKY17	At5g24570	0,00	0,00	-3,02	-2,80	1	1	-0,36			-1,47	same	WRKY DNA-BINDING PROTEIN 17
HSP20-like	At4g21870	0,00	0,00	-2,76	-2,92	1	1	-0,42	-3,00		-1,68	same	HEAT SHOCKr PROTEIN20 like
NPF6.2	At2g26690	0,00	0,00	-2,55	-2,63	1	1	-0,38	-1,54		-1,47	same	NRT1/PTR anion transporter, Major facilitator superfamily protein
	At5g62280	0,00	0,00	-1,76	-2,56	1	1	-0,12			-1,29	same	DUF1442 family protein
SAUR14	At4g38840	0,00	0,00	-2,25	-1,73	1	1	-0,25	-1,88		-1,09	same	SMALL AUXIN UPREGULATED RNA 14
NAC2/ORESARA1	At5g39610	0,00	0,01	-1,89	-1,16	1	1	-1,30			-1,01	same	NAC20 TF, positively regulates leaf senescence, also abiotic stress responses, affects primary root
TN13	At3g04210	0,00	0,00	-1,86	-1,04	1	1	-0,30	1,64		-1,05	same	TIR-NBS protein, contributes to RPS5-triggered immunity.
ARR9	At3g57040	0,00	0,00	-1,40	-1,49	1	1	-0,38	-1,57	1,54	-1,56	same	Response regulator ARR9, cytokin signalling
Zn Finger	At5g60710	0,00	0,00	-1,63	-1,26	1	1	-0,23			-0,98	same	Zinc finger (C3HC4-type RING finger) family protein, UBQ-ligase E3
JAZ1	At1g72450	0,00	0,00	-0,97	-0,75	1	1	-0,16			-1,82	same	jasmonate-zim-domain protein 6, TIFY DOMAIN PROTEIN 11B
	At5g25840	0,00	0,00	-0,34	-0,87	1	1	-0,54			-1,00	same	DUF1677 family protein
FLZ13	At1g74940	0,59	0,00	-0,09	-0,40		1	-0,17			-1,96	same	
ALDH6B2	At2g14170	0,04	0,04	-0,21	-0,28	1	1	-1,00	-2,16	2,05	-2,39	same	Methylmalonate-semialdehyde dehydrogenase
	At2g39130	0,00	0,47	0,34	0,11	1		0,34			1,26	same	Transmembrane amino acid transporter family protein; C
	At2g14835	0,00	0,01	0,36	0,34	1	1	0,14			1,53	same	
	At5g17760	0,73	0,00	0,09	0,78		1	0,58	2,23	-1,98	1,72	same	P-loop containing nucleoside triphosphate hydrolases superfamily protein
APC2	At2g04660	0,00	0,00	0,66	0,80	1	1	0,20			1,47	same	ANAPHASE-PROMOTING COMPLEX/CYCLOSOME 2, cell cycle
ALKBH10A	At2g48080	0,01	0,00	1,09	1,11	1	1	0,14			1,58	same	2OG-Fe (II) oxygenase family protein; mRNA demethylation
SAUR30	At5g53590	0,00	0,00	2,02	2,13	1	1	0,35	1,56	1,95	1,76	same	SMALL AUXIN UPREGULATED RNA 30
BT2	At3g48360	0,00	0,00	-3,66	-4,02	1	1	-2,22	-3,98	1,97	2,70	opposite	BT2 is an essential component of the TAC1-mediated telomerase activation pathway.
	At2g25200	0,00	0,00	-3,54	-3,43	1	1	-1,82		2,20	1,31	opposite	
BXL1	At5g49360	0,00	0,00	-2,80	-3,78	1	1	-2,64	-3,38	5,06	2,21	opposite	BETA-XYLOSIDASE 1, rhamnogalacturonan I.modification and degradation.
	At3g26510	0,00	0,00	-3,55	-2,65	1	1	-1,57	-1,41	2,71	1,89	opposite	Octicosapeptide/Phox/Bem1p family protein;
NAC2	At3g15510	0,00	0,00	-3,65	-2,27	1	1	-0,46			1,75	opposite	NAC transcription factor
	At1g62510	0,00	0,00	-2,62	-2,78	1	1	-1,22		3,74	1,09	opposite	Expressed in root cortex
GRXS6	At3g62930	0,00	0,00	-2,39	-2,50	1	1	-0,61	-2,22		1,57	opposite	CC-type glutaredoxin (ROXY) family, interacts wth TF TGA2 and suppress ORA59 promoter activity
	At5g11070	0,00	0,00	-2,34	-2,36	1	1	-1,53	-3,04		1,03	opposite	hypothetical protein
XTH30	At1g32170	0,00	0,00	-2,67	-1,98	1	1	-0,70		2,60	0,96	opposite	XYLOGLUCAN ENDOTRANSGLUCOSYLASE/HYDROLASE 30, cell wall modification
PRX47	At4g33420	0,00	0,00	-2,77	-1,80	1	1	-0,37			1,23	opposite	Peroxidase superfamily protein
BBE18	At4g20820	0,00	0,00	-2,84	-1,65	1	1	-0,24	-1,97		2,32	opposite	FAD-binding Berberine family protein
TRE1	At4g24040	0,00	0,00	-2,14	-2,30	1	1	-1,04	-2,44		3,43	opposite	TREHALASE 1
PLL12	At5g04310	0,00	0,00	-1,88	-2,17	1	1	-0,23	-1,05		2,28	opposite	PECTATE LYASE LIKE12, cell wall modification
OTU1	At2g28120	0,00	0,00	-1,96	-1,96	1	1	-1,00	-1,51		1,47	opposite	OVARIAN TUMOR DOMAIN (OTU)-CONTAINING DUB (DEUBIQUITILATING ENZYME) 1
	At5g08350	0,00	0,00	-2,22	-1,68	1	1	-1,48	-2,79		2,21	opposite	
DOF1.8	At1g64620	0,00	0,00	-2,16	-1,57	1	1	-0,10			2,30	opposite	DOF TF which regulates vascular cell differentiation and lignin biosynthesis
RZPF34,	At5g22920	0,00	0,00	-1,19	-2,26	1	1	-2,88	-4,25	6,76	2,38	opposite	CHY ZINC-FINGER AND RING PROTEIN 1,
HB-12	At3g61890	0,00	0,00	-1,68	-1,64	1	1	-0,93		2,66	1,72	opposite	HOMEOBOX 12
COR47	At1g20440	0,00	0,00	-1,31	-1,78	1	1	-0,51			1,38	opposite	COLD-REGULATED 47
	At4g27657	0,00	0,00	-1,24	-1,63	1	1	-0,17			3,37	opposite	hypotheticl protein
CHX17	At4g23700	0,00	0,00	-1,47	-1,39		1	-0,18			1,32	opposite	CATION/H+ EXCHANGER 17,
SCPL31,	At1g11080	0,00	0,00	-1,36	-1,35	1	1	-0,50			1,33	opposite	SERINE CARBOXYPEPTIDASE-LIKE 31
PPDK	At4g15530	0,00	0,00	-1,48	-1,23		1	-1,06		2,24	2,00	opposite	PYRUVATE ORTHOPHOSPHATE DIKINASE
SEN1	At4g35770	0,22	0,00	-0,33	-2,34		1	-3,56	-3,11	3,76	4,25	opposite	Senescence-associated gene that is strongly induced by phosphate starvation
BTB/POZ	At2g30600	0,00	0,00	-1,33	-1,17	1	1	-1,81	-3,07	3,85	1,27	opposite	BTB/POZ domain-containing proteins
	At4g18340	0,00	0,00	-1,32	-1,06	1	1	-1,08	-1,70		1,38	opposite	Glycosyl hydrolase superfamily protein;
	At1g12080	0,00	0,00	-0,80	-1,37	1	1	-0,33	-1,25		1,58	opposite	Vacuolar calcium-binding protein-like protein;
NPY3	At5g67440	0,00	0,00	-1,15	-0,77	1	1	-0,52			1,30	opposite	NAKED PINS IN YUC MUTANTS Family (1-5) involved in auxin-mediated organogenesis.
PP2-A13	At3g61060	0,00	0,00	-0,70	-1,15	1	1	-2,56	-3,73	2,99	2,06	opposite	PHLOEM PROTEIN 2-A13, PP2-A13
NAC047	At3g04070	0,00	0,00	-1,11	-0,60	1	1	-0,59			1,26	opposite	NAC DOMAIN CONTAINING PROTEIN 47, SPEEDY HYPONASTIC GROWTH
UGT88A1	At3g16520	0,00	0,00	-1,14	-0,56	1	1	-0,19	-2,04		2,61	opposite	UDP-GLUCOSYL TRANSFERASE 88A1
XMPP	At2g32150	0,00	0,00	-1,01	-0,63	1	1	-2,21	-2,74	2,75	1,60	opposite	xanthosine monophosphate (XMP) phosphatase. Initial step in purine nucleotide catabolism
HMP37	At4g27590	0,03	0,98	-1,62	0,02	1		-0,20			1,99	opposite	HEAVY METAL ASSOCIATED PROTEIN 37 Heavy metal transport/detoxification superfamily
HAT2	At5g47370	0,00	0,00	-0,60	-0,98	1	1	-0,16			1,75	opposite	Homeobox-leucine zipper genes induced by auxin, but not by other phytohormones.
CHX20	At3g53720	0,00	0,00	-0,70	-0,85	1	1	-0,41			1,26	opposite	Member of putative Na+/H+ antiporter family. Omoregulation through K^+ flux, possibly pH modulation
SDP1	At5g04040	0,00	0,00	-0,94	-0,51	1	1	-1,01	-1,39	2,49	1,48	opposite	SUGAR-DEPENDENT1 Triacylglycerol lipase involved in storage lipid breakdown in seed germination
BAM9	At5g18670	0,00	0,00	-0,84	-0,59	1	1	-1,87	-2,10	3,59	2,06	opposite	BETA-AMYLASE 9, positive regulation of starch breakdown
PDH2	At5g38710	0,02	0,01	-0,65	-0,76	1		-0,76			1,36	opposite	Methylenetetrahydrofolate reductase family protein, prolone catabolism
GOLS	At1g56600	0,04	0,51	-1,03	-0,31	1		-0,31			1,14	opposite	galactinol synthase
	At4g30490	0,00	0,00	-0,79	-0,47	1	1	-0,37			2,50	opposite	AFG1-like ATPase family protein
	At1g21680	0,19	0,00	-0,20	-0,97		1	-1,49	-3,11		1,95	opposite	DPP6 N-terminal domain-like protein
CMCU	At5g66650	0,01	0,09	-0,65	-0,48	1		-0,72		3,35	2,14	opposite	CHLOROPLAST-LOCALIZED MITOCHONDRIAL CALCIUM UNIPORTER,
	At3g47000	0,00	0,00	-0,66	-0,43	1	1	-0,51	-1,61		1,40	opposite	Glycosyl hydrolase family protein;
ZIF2	At2g48020	0,00	0,00	-0,58	-0,48	1	1	-0,76	-2,08		1,22	opposite	ZINC-INDUCED FACILITATOR transport protein
SIP2	At2g30360	0,00	0,11	-0,68	-0,37	1		-0,54			1,45	opposite	CBL-interacting protein kinase. Regulates H^+ transporter.Also ABI5, phosphorylation induced by ABA,
	At3g04010	0,00	0,68	-1,15	0,11	1		-0,15			1,07	opposite	O-Glycosyl hydrolases family 17 protein
ACX2	At5g65110	0,00	0,01	-0,59	-0,35	1	1	-0,97			1,20	opposite	ACYL-COA OXIDASE 2
DIN4	At3g13450	0,03	0,00	-0,34	-0,52	1	1	-1,89	-2,52	4,02	1,90	opposite	branched chain alpha-keto acid dehydrogenase E1 beta
ACR9	At2g39570	0,00	0,03	-0,57	-0,28	1	1	-1,83	-2,89	1,98	2,40	opposite	ACT DOMAIN REPEATS 9. ACT domain is an amino acid-binding site in feedback-regulation of amino acid metabolic enzymes
	At2g36310	0,00	0,00	-0,48	-0,35	1	1	-0,48			1,29	opposite	Cytoplasmic nucleoside hydrolase, Inosine/uridine-preferring nucleoside hydrolase
ACHT5	At5g61440	0,67	0,00	-0,09	-0,72		1	-1,31	-3,47		1,32	opposite	Encodes a member of the thioredoxin family protein, chloroplast located
SHL	At4g39100	0,00	0,02	-0,43	-0,30	1	1	-0,57	-1,19		1,33	opposite	SHORT LIFE. Plant-specific histone reader capable of recognizing both H3K27me3 and H3K4me3
XER	At2g04240	0,02	0,04	-0,37	-0,34	1	1	-0,39			1,46	opposite	XERICO
LNK2	At3g54500	0,00	0,62	-0,55	-0,08	1		-0,82	-2,12		1,34	opposite	LINK2 Member of a small family. Along with LNK1 integrates light signaling and circadian clock.
	At1g63840	0,00	0,49	-0,66	0,14	1		-0,38			1,15	opposite	RING/U-box superfamily protein
RMR2	At1g71980	0,00	0,54	-0,31	-0,10	1		-0,40			2,64	opposite	Secretory pathway protein localized to the trans-golgi network. family of vacuolar sorting receptors.
	At3g43430	0,02	0,25	-0,59	0,28	1		-0,35	-1,29		1,55	opposite	RING-H2-type E3 ligase
LSH6	At1g07090	0,79	0,01	0,04	0,36		1	0,60	1,32		-1,25	opposite	LIGHT SENSITIVE HYPOCOTYLS 6,
TWN2	At1g14610	0,00	0,01	0,37	0,41	1	1	0,39	1,52		-1,10	opposite	VALYL TRNA SYNTHETASE
TBL27	At1g70230	0,00	0,00	0,43	0,47	1	1	0,76		-1,84	-1,25	opposite	ALTERED XYLOGLUCAN 4, AXY4, TRICHOME BIREFRINGENCE-LIKE 27
EXPA3	At1g26770	0,00	0,00	0,60	1,58	1	1	0,67		-3,27	-1,01	opposite	EXPANSIN A3
HSP70-4	At3g12580	0,00	0,00	1,36	1,43	1	1	0,58	1,81		-1,59	opposite	HEAT SHOCK PROTEIN 70-4

Supplemental Figure S17. Comparison of iTPS with constitutive overexpression of zBIP11 (continued)

F **Transcripts that are present in the oebZIP11 gene set of Ma et al. (2009) and are assigned to the iTPS Crf group G_1: comparison of the response to oebZIP11 with the responses to iTPS or to constitutive oeTPS (from Zhang et al., 2009).**

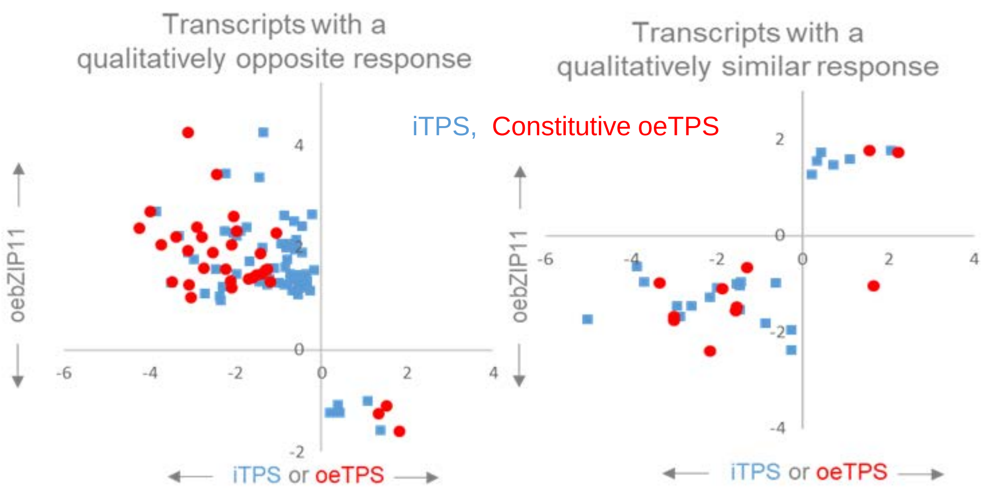

Transcripts with a qualitatively opposite response to iTPS and oebZIP11 are regulated by Tre6P and bIP11 in a mutually reinforcing manner, whilst transcripts that show qualitatively similar responses to these treatments are regulated by Tr6P and bZIP11 in a antagonistic manner. For examples see Supplemental text, section 'Overlap with bZIP11 signaling'.

G **Transcripts that are present in the oebZIP11 gene set of Ma et al. (2009): Comparison of their iTPS and their tSnRK1α1 response. (from Baena-Gonález et al. 2007).** This is done from left to right) or all transcripts and for transcripts assigned to CRF group G_1 and group G_2

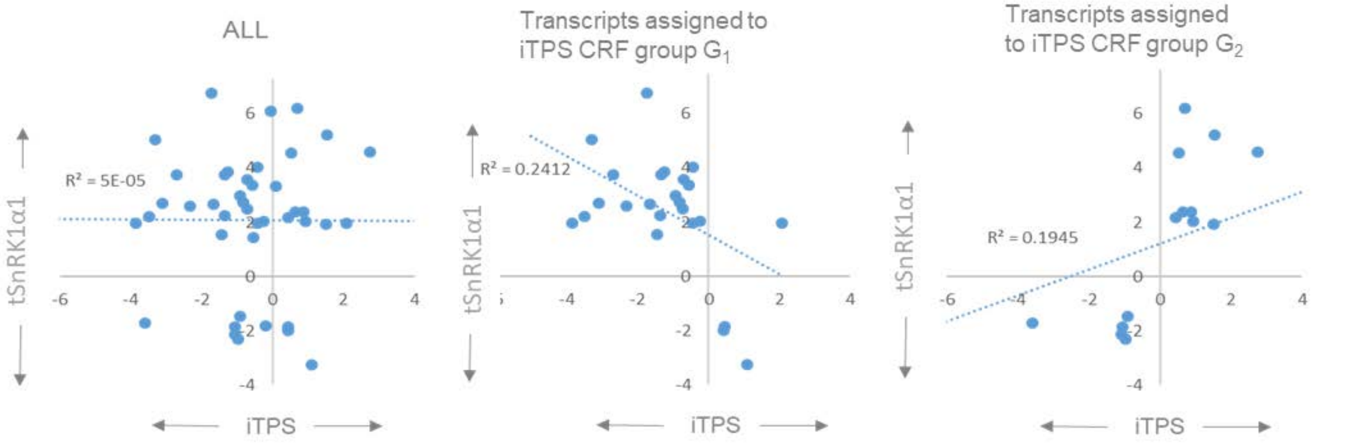

H **Transcripts that are present in the oebZIP11 gene set of Ma et al. (2009): comparison of their tSnRK1α1 response and their oebZIP11 response**. This is done from left to right) or all transcripts and for transcripts assigned to CRF group G_1 and group G_2

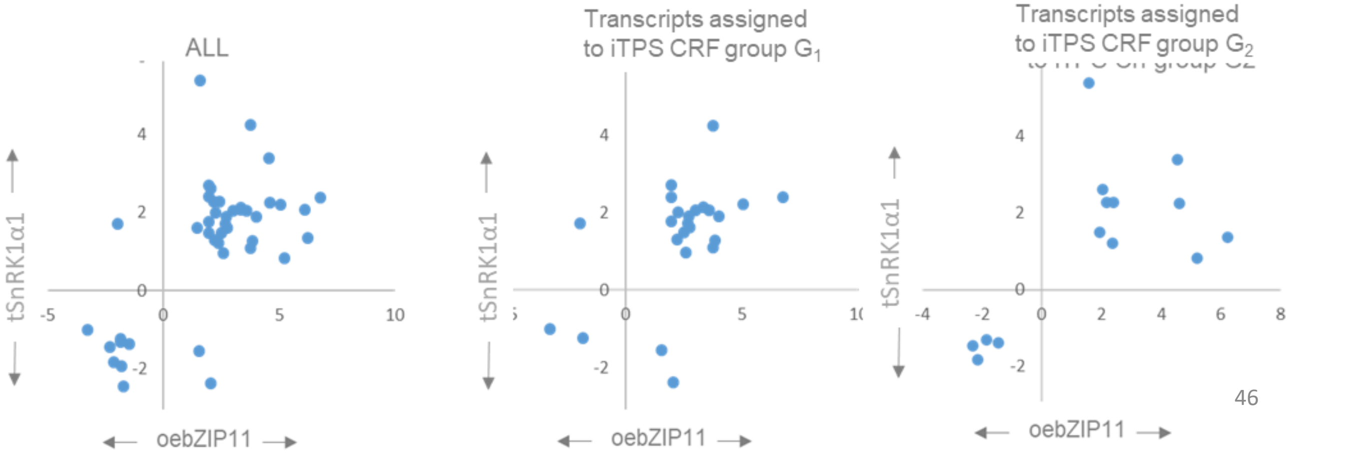

J **Response of trehalose-metabolism related genes.** The display shows the 4-h and 6-h iTPS response, assignment of the iTPS response to CRF groups, the CRF value (defined as in Supplemental Figure S12), the response to constitutive overexpression of TPS (oeTPS, from Zhang et al., 2009) and to transient overexpression of tnRL1α1 in protoplasts (tSnRL1α1, from Baena-Gonzalez et al., 2007) and to oebZIP11. Significant changes in the iTPS response are shown with bold font. Assignment to CRF groups is performed only for significant responses and only CRF G_1 assignments are shaded. No entry for oeTPS or tSnRL1α1indicates that the response did not pass the filter in the publication.

Gene		iTPS response						CRF	Other responses				Comment
Name	Code	4-h adj.p	6-h adj.p	**4-h FC log_2**	**6-h FC log_2**	4-h CRF score	6-h CRFscore		oeTPS FC log_2	tSnRKα1 FC Log_2	**oe bZIP11**	**direction, bZIP11 compared to iTPS**	
TRE1	At4g24040	0,00	0,00	-2,14	-2,30	1	1	-1,04	-2,44		**3,43**	opposite	responses in iTPS and constituve oeTPS are in same direction
TPPG	At4g22590	0,00	0,00	0,83	0,94	0	0	0,09	-1,21		**1,77**	same	responses in iTPS and constituve oeTPS in opposed directions
TPPF	At4g12430	0,38	0,54	0,16	0,12			-0,09	-4,03		**1,26**	none	no response in iTPS and large decrease in oeTPS

Supplemental Figure S18. Transcription factors that respond to an induced increase in Tre6P levels.

(A) Examples of transcription factors (TFs) that showed a strong response and were assigned to CRF G group G1.

(B-E) TFs listed according to their family: (B) bZIPS, (C) WRKYs, (D) AP2/ERF, ED) MYBs. These panels show the 4-h and 6-h iTPS response. All changes were significant (FDR<0.05) and a filter was set for FC≥2 for at least one time point. Responses are listed separately for different CRF groups. For comparison, the published responses to constitutive overexpression of TPS in Arabidopsis is provided (oeTPS, from Zhang et al., 2009; a missing value means that the data is not available). The FC is given on a log_2 scale.

FC is fold change, iTPS is response to induced expression of bacterial TPS, oeTPS is constitutive overexpression of TPS.

Displays are shown on the next page

.

A

ID	iTPS 4h	iTPS 6h	Name	Description
At5g57520	-4.6	-2.7	ZFP2	negative regulation of floral organ abscission
At5g59780	-4.3	-3.5	MYB59	cellular response to potassium ion. In roots it is involved in K+/NO3- transport and expression of the NPF7.3 transporter
At3g46130	-4.2	-3.3	MYB48	regulate flavonol biosynthesis primarily in cotyledons
At2g18300	-4.1	-3.1	HBI1	bHLH protein involved in positive regulation of cell elongation and proliferation and, negative control of plant immunity. BR signaling.
At1g69490	-3.8	-1.9	NAP	It is expressed in floral primordia and upregulated by AP3 and PI
At5g61590	-3.7	-3.9	DEWAX	ERF107. preferentially expressed in the epidermis and induced by darkness and negatively regulates cuticular wax AND anthocyanins biosynthesis
At3g15510	-3.7	-2.3	ANAC2	putative regulator of phloem parenchyma wall ingrowth deposition
At3g29035	-3.2	-2.4	ANAC059	contains CCT domain (CONSTANS like)
At3g54990	-3.2	-2.0	SMZ	Encodes a AP2 domain transcription factor that can repress flowering. SMZ and its paralogous gene, SNARCHZAPFEN (SNZ), share a signature with partial complementarity to the miR172 microRNA, whose precursor is induced upon flowering
At4g00050	-3.2	-2.8	PIF8	Encodes a phytochrome interacting factor that inhibits phytochrome A-mediated far-red light responses and binds to promoter regions of AT2G46970 and AT3G62090.
At5g15830	-3.1	-2.3	bZIP3	bZIP3) is a novel sugar-responsive transcription factor in Arabidopsis plants. The expression of bZIP3 was rapidly repressed by sugar. Genetic analysis indicated that bZIP3 expression was modulated by the SNF1-RELATED KINASE 1 (SnRK1) pathway
At5g56860	-3.1	-2.2	GATA21	GATA, nitrate-inducible, carbon metabolism-involved
At2g20180	-2.7	-2.4	PIF1	Inhibited by light, gibberellin biosynthesis. key negative regulator of phytochrome-mediated seed germination and acts by inhibiting chlorophyll biosynthesis, light-mediated suppression of hypocotyl elongation and far-red light-mediated suppression of seed germination, and promoting negative gravitropism in hypocotyls
At1g79700	-2.7	-2.4	WRI4	specifically controls cuticular wax biosynthesis. functions to activate transcription of genes involved fatty acid biosynthesis during seed and flower development as well as stem wax biosynthesis
At4g17460	-2.7	-2.7	HAT1	regulates meristematic activity
At1g66230	-2.6	-2.4	MYB20	directly activates lignin biosynthesis genes and phenylalanine biosynthesis genes during secondary wall formation. down-regulates the expression of PP2Cs, the negative regulator of ABA signaling, and enhances salt tolerance
At1g71030	-2.2	-1.5	MYBL2	anthocyanin-containing compound biosynthetic process
At1g25550	-2.1	-2.0	HHO3	Transcriptional repressors that functions with other NIGT genes as an important hub in the nutrient signaling network associated with the acquisition and use of nitrogen and phosphorus.
At5g65210	-1.9	-1.3	TGA1	important regulatory factors of the nitrate response in Arabidopsis roots via NRT2.1 and NRT2.2
At5g37260	-1.6	-1.7	CIR1	RVE2, Involved in circadian regulation
At5g49450	-1.6	-1.9	bZIP1	A positive regulator of plant tolerance to salt, osmotic and drought stresses, crucial transcriptional regulators in Pro, Asn, and branched-chain amino acid metabolism. Expression is repressed by sugars, mediated by HEX.
At5g28770	-1.5	-1.6	bZIP63	Phosphorylated by SnRK1. composes a regulatory interface between the metabolic and circadian control of starch breakdown to optimize C usage and plant growth. Hexokinase 1 is required for glucose-induced repression of bZIP63
At4g01120	-1.5	-1.6	bZIP54	glucosinolate metabolic process, response to blue light
At2g18160	-1.5	-1.5	bZIP2	GBF5, FLORAL TRANSITION AT THE MERISTEM3
At1g79430	-1.2	-1.4	APL	required for several aspects of phloem development in the root: (1) the specific divisions organizing the phloem pole, (2) sieve element differentiation and (3) the expression of a companion-specific gene
At1g18330	-1.2	-1.3	EPR1	RVE7, EARLY-PHYTOCHROME-RESPONSIVE1
At5g11260	-1.0	-1.0	HY5	photomorphogenesis (through UVR8 signaling pathway)
At1g56650	0.7	1.5	PAP1	involved in anthocyanin metabolism and radical scavenging, Essential for the sucrose-mediated expression of the dihydroflavonol reductase gene
At1g62150	1.5	1.4	mTERF20	Mitochondrial transcription termination factor family member, active in chloroplasts
At1g17460	1.7	1.4	TRFL3	TRFL3, negative regulation of gene expression

Supplemental Figure S18. Transcription factors that respond to an induced increase in Tre6P levels (continued).

B. bZIPs

G_1

ID	iTPS 4h	iTPS 6h	oeTPS	Name
At5g15830	-3.13	-2.31	n.d	bZIP3
At5g65210	-1.91	-1.28	n.d	TGA1
At2g22850	-1.76	-1.97	-2.16	bZIP6
At3g51960	-1.75	-2.12	n.d	bZIP24
At5g49450	-1.55	-1.88	-3.35	bZIP1
At5g28770	-1.53	-1.65	-2.86	bZIP63
At4g01120	-1.52	-1.64	n.d	bZIP54
At2g18160	-1.49	-1.45	-2.09	bZIP2
At1g13600	-1.29	-1.72	n.d	bZIP58
At5g24800	-1.18	-0.67	n.d	bZIP9
At5g11260	-1.02	-0.99	-1.09	HY5
At2g40950	0.73	1.12	N.D	bZIP17

G_2

ID	iTPS 4h	iTPS 6h	oeTPS	Name
At2g42380	-3.78	-3.19	-1.97	bZIP34
At3g58120	-3.22	-3.10	n.d	bZIP61
At5g10030	-2.42	-1.93	-1.26	OBF4
At1g06850	-1.46	-1.44	n.d	bZIP52
At1g68640	-1.03	-1.39	n.d	TGA8
At4g34590	-0.42	0.16	1.68	bZIP11
At1g77920	-0.76	-0.44	n.d	TGA7
At1g75390	-0.49	-0.55	n.d	bZIP44
At2g31370	-0.30	-0.24	n.d	bZIP59
At5g06960	0.38	0.57	n.d	OBF5
At3g10800	0.69	0.92	n.d	bZIP28
At2g40620	1.65	1.28	n.d	bZIP18
At2g41070	1.70	1.40	-1.03	bZIP12

G_0

ID	iTPS 4h	iTPS 6h	oeTPS	Name
At3g30530	-2.24	-0.12	n.d	bZIP42
At2g17770	-1.51	-1.72	n.d	bZIP27
At2g36270	-1.09	-1.45	n.d	ABI5
At4g35900	-0.99	-1.45	-1.14	bZIP14 (FD)
At4g38900	-0.39	-0.24	n.d	bZIP29
At5g06950	0.43	0.30	n.d	TGA2
At3g56850	0.61	0.50	n.d	DBPF3

C. WRKY

G_1

ID	iTPS 4h	iTPS 6h	oeTPS	Name
At5g07100	-2.93	-2.96	n.d	WRKY26
At4g01720	-2.75	-2.00	n.d	WRKY47
At2g03340	-1.59	-1.14	n.d	WRKY3
At4g01250	-1.28	-0.99	n.d	WRKY22
At3g58710	-0.75	-1.03	1.08	WRKY69
At2g46400	0.65	0.70	2.04	WRKY46
At5g22570	1.80	1.83	n.d	WRKY38

G_2

ID	iTPS 4h	iTPS 6h	oeTPS	Name
At1g29860	-2.82	-0.62	-1.39	WRKY71
At5g52830	-2.59	-2.02	n.d	WRKY27
At4g23810	-1.35	-0.50	n.d	WRKY53
At2g24570	-1.15	-0.76	1.16	WRKY17
At2g25000	-0.73	-0.53	n.d	WRKY60
At4g12020	0.34	0.59	n.d	WRKY19
At3g01970	0.58	0.79	n.d	WRKY45
At2g40750	0.62	0.72	n.d	WRKY54
At3g56400	1.36	1.29	n.d	WRKY70
At4g31800	1.41	0.88	n.d	WRKY18

G_0

ID	iTPS 4h	iTPS 6h	oeTPS	Name
At4g39410	-3.46	-2.54	n.d	WRKY13
At2g34830	-2.51	-1.77	n.d	WRKY35
At2g44745	-2.21	-1.99	n.d	WRKY12
At5g28650	-1.90	-2.12	n.d	WRKY74
At4g24240	-1.38	-1.18	n.d	WRKY7
At1g29280	-1.35	-2.52	n.d	WRKY65
At2g40740	-0.89	-0.35	n.d	WRKY55
At1g30650	-0.86	-0.71	n.d	WRKY14
At1g69310	-0.81	-0.44	n.d	WRKY57
At4g30935	0.66	0.78	n.d	WRKY32
At2g21900	1.59	0.72	n.d	WRKY59
At4g26440	6.02	4.97	n.d	WRKY34

down
up

Supplemental Figure S18. Transcription factors that respond to an induced increase in Tre6P levels Continued

D. AP2/ERF

G_1

ID	iTPS 4h	iTPS 6h	oeTPS	Name
At1g06160	-4.45	-3.57	n.d	ERF59
At5g61590	-3.72	-3.95	-3.17	DEWAX
At2g20880	-3.54	-1.92	n.d	ERF53
At3g54990	-3.21	-1.96	-1.15	SMZ
At1g79700	-2.68	-2.41	-1.49	WRI4
At3g16770	-2.53	-2.60	n.d	ERF72
At4g28140	-2.12	-0.51	n.d	ERF54
At3g60490	-1.85	-1.30	n.d	ERF35
At1g46768	-1.81	-1.98	n.d	RAP2.1
At5g67190	-1.68	-1.54	n.d	DEAR2
At5g47220	-1.56	-1.27	n.d	ERF2
At4g39780	-1.50	-0.72	n.d	ERF60
At4g17490	-1.24	-1.05	n.d	ERF6
At1g72360	-1.12	-0.71	n.d	ERF73
At1g36060	-1.06	-0.21	n.d	WIND3
At5g61600	-0.99	-1.43	n.d	ERF104
At1g22190	-0.97	-0.63	n.d	WIND2
At4g17500	-0.85	-0.38	n.d	ERF1
At3g50260	-0.68	-0.48	n.d	DEAR1
At1g53910	-0.56	-0.58	n.d	ERF74
At1g50640	-0.53	-0.41	n.d	ERF3
At1g25470	-0.34	-0.39	n.d	CRF12
At5g22770	0.34	0.54	n.d	AP2A1
At2g31230	0.40	0.62	n.d	ERF15
At2g23340	0.42	0.19	n.d	DEAR3
At4g23750	0.80	0.63	n.d	CRF2

G_2

ID	29.2 4h	29.2 6h	oeOTSA	name
At5g07580	-2.81	-1.68	-1.26	DEWAX2
At2g39250	-2.55	-1.97	n.d	SNZ
At5g44210	-1.81	-1.50	n.d	ERF9
At4g37750	-1.56	-1.69	n.d	DRG
At4g32800	-1.55	-1.27	n.d	ERF43
At4g36920	-1.43	-1.07	n.d	FLO2
At5g53290	-0.68	-0.52	1.66	CRF3
At1g16060	-0.61	-1.14	n.d	WRI3
At5g60120	-0.60	-0.53	n.d	EAT2
At1g68550	-0.56	-0.31	1.19	CRF10
At2g44940	-0.53	-0.31	n.d	ERF34
At2g28550	-0.41	-0.30	n.d	EAT1
At4g36900	1.14	1.21	n.d	DEAR4
At5g13330	1.18	2.43	n.d	related to AP2 6l
At2g38340	1.31	1.00	3.86	DREB19
At3g11020	1.94	0.73	n.d	DREB2
At1g43160	2.92	4.33	n.d	related to AP2 6

G_0

ID	29.2 4h	29.2 6h	oeOTSA	name
At4g25490	-3.28	-2.37	n.d	CBF1
At5g25390	-3.25	-2.79	n.d	SHN3
At5g25190	-3.19	-2.65	-1.49	ESE3
At1g33760	-3.18	-2.27	n.d	ERF022
At4g25470	-2.60	-2.96	n.d	CBF2
At2g22200	-2.42	-2.33	n.d	T26C19.14
At5g57390	-2.33	-1.81	n.d	AIL5
At1g77200	-2.11	-0.81	n.d	T14N5.6
At4g11140	-1.79	-2.21	n.d	CRF1
At4g25480	-1.75	-0.95	n.d	DREB1A
At4g16750	-1.54	-1.68	n.d	ERF39
At1g78080	-1.32	-0.62	n.d	WIND1
At5g47230	-1.11	-0.42	1.16	ERF5
At5g65510	-1.11	-1.51	-2.58	AIL7
At3g54320	-0.98	-0.85	1.17	WRI1
At3g23230	-0.94	-0.77	n.d	TDR1
At5g67180	-0.75	-1.19	-1.41	TOE3
At3g59990	0.35	0.40	n.d	MAP2B
At3g15210	0.99	1.04	n.d	ERF4
At1g74930	1.76	1.17	-1.69	ORA47
At5g25810	3.04	2.72	n.d	TNY
At1g71450	3.23	3.33	n.d	FUF1
At2g47520	3.69	3.64	n.d	ERF71
At1g12630	4.51	4.53	n.d	T12C24.16
At5g52020	4.95	4.78	n.d	MSG15.10
At1g72570	5.95	5.86	n.d	F28P22.24
At5g67000	6.20	3.54	n.d	K8A10.7
At1g12610	8.86	6.79	n.d	DDF1
At1g63030	9.22	8.64	n.d	DDF2

down
up

Supplemental Figure S18. Transcription factors that respond to an induced increase in Tre6P levels Continued.

E. MYBs

G_1

ID	iTPS 4h	iTPS 6h	oeTPS	Name
At5g59780	-4.33	-3.52	n.d	MYB59
At3g46130	-4.18	-3.33	-1.40	ATMYB48
At5g53200	-3.51	-2.35	n.d	TRY
At1g66230	-2.63	-2.40	-2.66	MYB20
At2g30420	-2.37	-1.98	n.d	ETC2
At1g22640	-2.35	-2.55	n.d	ATMYB3
At5g57620	-2.31	-2.65	n.d	MYB36
At4g37260	-2.28	-2.68	n.d	MYB73
At1g71030	-2.15	-1.53	-1.36	ATMYBL2
At1g19510	-2.13	-3.18	n.d	ATRL5
At1g25550	-2.12	-1.98	n.d	HHO3
At5g44190	-2.00	-1.29	n.d	GLK2
At4g09460	-1.94	-1.85	-2.23	AtMYB6
At2g23290	-1.75	-1.83	n.d	AtMYB70
At5g42630	-1.72	-2.07	n.d	KAN4
At2g47190	-1.58	-2.11	n.d	ATMYB2
At5g37260	-1.58	-1.69	-2.92	CIR1
At5g15310	-1.54	-1.41	n.d	ATMYB16
At1g70000	-1.47	-0.42	n.d	MYBD
At5g08520	-1.46	-1.18	n.d	MYBS2
At1g75250	-1.41	-1.91	n.d	ATRL6
At1g79430	-1.24	-1.38	n.d	APL
At3g06490	-1.23	-3.04	n.d	MYB108
At1g49010	-1.21	-1.17	n.d	MYBS1
At1g18330	-1.17	-1.31	-3.10	EPR1
At3g47600	-1.10	-0.83	n.d	MYB94
At5g17300	-1.09	-0.39	-2.74	RVE1
At5g06800	-1.06	-1.76	n.d	MPH15.16
At5g59430	-1.04	-0.52	n.d	ATTRP1
At2g46410	-1.03	-0.54	n.d	CPC
At1g19000	-1.02	-0.89	-1.08	F14D16.15
At1g74840	-0.99	-1.13	-1.01	F25A4.19
At3g12730	-0.98	-1.63	n.d	MBK21.11
At1g56650	0.72	1.54	n.d	PAP1
At3g55730	1.52	1.56	n.d	MYB109
At1g17460	1.69	1.44	n.d	TRFL3

down
up

G_2

ID	iTPS 4h	iTPS 6h	oeTPS	Name
At5g60890	-4.33	-3.52	n.d	MYB34
At2g38090	-3.11	-2.74	n.d	F16M14.2
At2g16720	-2.92	-2.24	n.d	MYB7
At5g07690	-2.86	-3.05	n.d	MYB29
At1g13300	-2.82	-2.64	n.d	HRS1
At5g65790	-2.61	-1.99	n.d	MYB68
At1g79180	-2.60	-1.16	n.d	MYB63
At5g05790	-2.59	-2.38	n.d	MJJ3.20
At4g38620	-2.57	-1.98	n.d	MYB4
At5g16600	-2.39	-1.82	n.d	MYB43
At2g36890	-2.36	-0.73	n.d	MYB38
At5g58900	-2.17	-1.90	1.58	DIV1
At3g48920	-2.13	-0.69	n.d	MYB45
At2g02820	-1.93	-1.85	n.d	MYB88
At4g17695	-1.81	-0.99	n.d	KAN3
At5g61420	-1.74	-1.86	n.d	MYB28
At4g12350	-1.65	-1.42	n.d	MYB42
At2g03500	-1.51	-1.38	n.d	EFM
At1g14350	-1.49	-1.63	n.d	MYB124
At2g37630	-1.43	-1.27	-1.36	MYB91
At4g22680	-1.14	-0.70	n.d	MYB85
At5g49330	-0.98	-0.26	2.06	MYB111
At1g58220	0.93	1.09	n.d	DRMY1
At4g01280	1.06	1.06	n.d	RVE5
At1g01520	1.12	1.65	n.d	REV3

G_0

ID	iTPS 4h	iTPS 6h	oeTPS	Name
At1g18710	-2.84	-2.11	n.d	MYB47
At1g74080	-2.60	-0.85	n.d	MYB122
At3g12820	-2.16	-3.15	n.d	MYB10
At1g63910	-2.08	-1.30	n.d	MYB103
At3g50060	-2.05	-1.69	n.d	MYB77
At1g66390	-2.04	-1.62	1.68	MYB90
At5g07700	-2.03	-2.03	n.d	MYB76
At1g66370	-1.98	-1.08	n.d	MYB113
At1g18570	-1.97	-1.16	2.01	MYB51
At5g10280	-1.73	-2.05	n.d	MYB92
At3g28910	-1.68	-1.18	n.d	MYB30
At5g67300	-1.65	-1.46	n.d	MYBR1
At3g60110	-1.62	-1.66	n.d	T2O9.90
At4g01680	-1.60	-1.66	n.d	MYB55
At2g02060	-1.52	-1.52	n.d	F14H20.13
At5g23000	-1.51	-2.16	n.d	MYB37
At5g14750	-1.49	-1.45	n.d	MYB66
At1g06180	-1.48	-0.94	n.d	MYB13
At2g40260	-1.35	-1.15	n.d	T7M7.13
At1g66380	-1.28	-1.54	n.d	MYB114
At2g47460	-1.26	-2.29	n.d	MYB12
At3g04030	-1.13	-1.27	n.d	MYR2
At5g58340	0.83	1.04	n.d	MCK7.21
At5g40350	1.69	0.35	n.d	MYB24
At3g28470	1.74	2.63	n.d	MYB35
At5g16770	2.39	0.94	n.d	AtMYB9
At2g42150	3.65	3.46	n.d	T24P15.6
At3g02940	4.91	2.04	n.d	MYB107
At5g55020	5.94	4.01	n.d	MYB120
At5g61620	7.91	6.86	n.d	K11J9.15

Supplemental Figure S19. Analysis of transcription factors assigned to CRF group G_1.

(A Enrichment analysis using Gene Ontology terms. Blue denotes fold enrichment and orange the FDR value. The nine categories at the bottom of the display were underrepresented.
FDR is false discovery rate

(B) STRING analysis (see next slide)

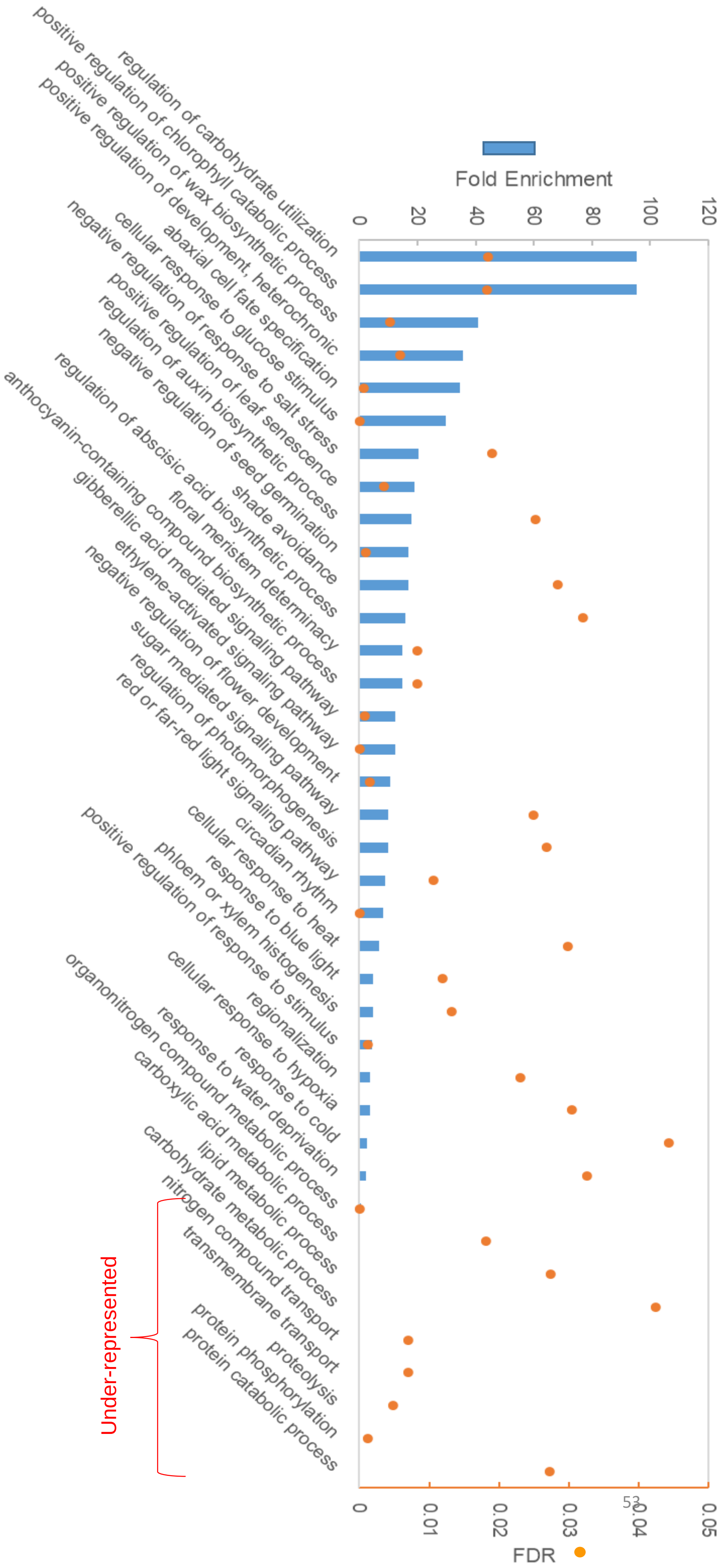

Supplemental Figure S19. Analysis of transcription factors assigned to Crf group G_1 (continued) .

(B) STRING analysis. The tool utilizes available datasets, published work, automated text mining and computational predictions from various organisms to score the likelihood of association between proteins, taking into account physical (direct) and functional (indirect) interactions (Szklarczyk et al., 2021). We used the more relaxed filter for expression (FDR < 0.05 and $\log_2$ FC ≥ 0.2; 290 TFs in total) and set a high interaction confidence score filter (interaction score ≥ 0.7). The thickness of the line connecting two nodes indicates the strength of data support, with thicker lines representing higher interaction score

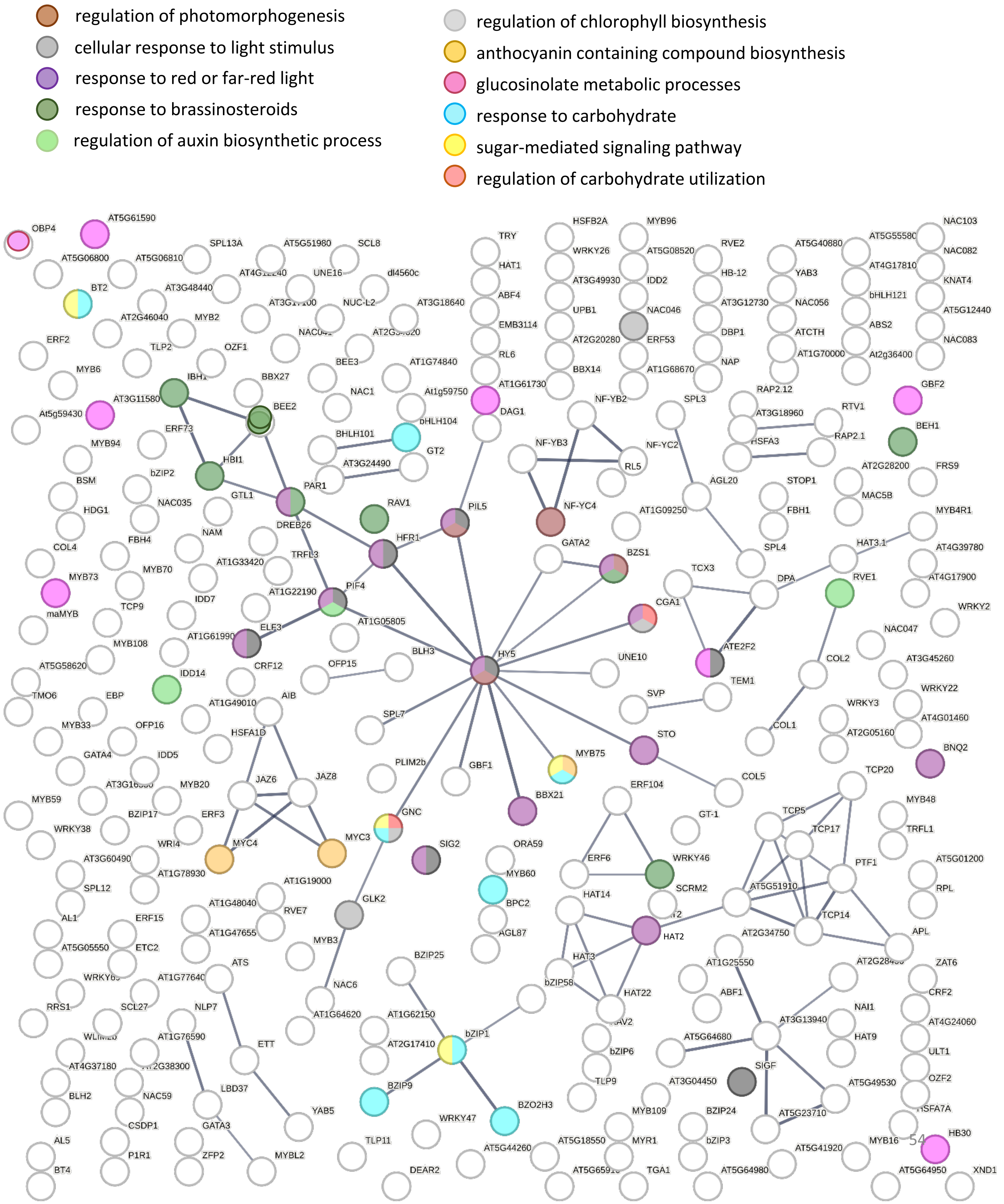

Supplemental Figure S20. C and S_1 bZIP transcription factors

(A) Response of members of the C and S_1 bZIP families. The display shows 4-h and 6-h iTPS response, assignment of the iTPS response to CRF groups, the CRF value (defined as in Supplemental Figure S2) and the response to constitutive overexpression of TPS (oeTPS, from Zhang et al., 2009) and transient overexpression of SnRL1α1 in protoplasts (tSnRL1α1 response, from Baena-Gonzalez et al., 2007). Significant changes in the iTPS response are shown with bold font. Assignment to CRF groups is performed only for significant responses and only CRF G_1 assignments are shaded. No entry for oeTPS or tSnRL1α1 indicates that the response did not pass the filter in the original publication.

CRF is carbon response factor, FC is fold change, iTPS is response to induced expression of bacterial TPS, oeTPS is constitutive overexpression of TPS.

A

Gene		iTPS response						CRF	Other responses		
Name	Code	4-h adj.p	6-h adj.p	4-h FC log_2	6-h FC log_2	4-h CRF score	6-h CRF score		oeTPS FC log_2	tSnRK1α1 FC Log_2	tSnRK1α1 compared to iTPS
bZIP9	At5g24800	**0.00**	**0.00**	**-1.18**	**-0.67**	1	1	-1.54		-1.32	Same
bZIP10	At4g02640	0.80	0.70	-0.03	0.06			0.01		-0.60	No iTPS response
bZIP25	At3g54620	**0.00**	**0.00**	**-0.59**	**-0.41**	1	1	-0.21		0.38	**Reciprocal**
bZIP63	At5g28770	**0.00**	**0.00**	**-1.53**	**-1.65**	1	1	-1.68	1.35	2.87	**Reciprocal**
bZIP1	At5g49450	**0.00**	**0.00**	**-1.55**	**-1.88**	1	1	-2.64	1.46	1.57	**Reciprocal**
bZIP2	At2g18160	**0.00**	**0.00**	**-1.49**	**-1.45**	1	1	-0.49			Same
bZIP11	At4g34590	**0.00**	0.27	**-0.42**	0.16	2		1.16	1.68	-3.11	Inconsistent
bZIP44	At1g75390	**0.03**	**0.02**	**-0.49**	**-0.55**	2	2	0.34		-0.03	No oe SnRK1 response
bZIP53	At3g62420	0.52	0.95	0.07	0.01			-0.58		0.44	No iTPS response

(B) Relationship between the iTPS response and the tSnRK1α1 response for bZIPs, separated into transcripts assigned to CRF group G_1, G_2 and G_0 (from left to right). The display shows the response of transcript abundance 4-h and 6-h post-induction

B

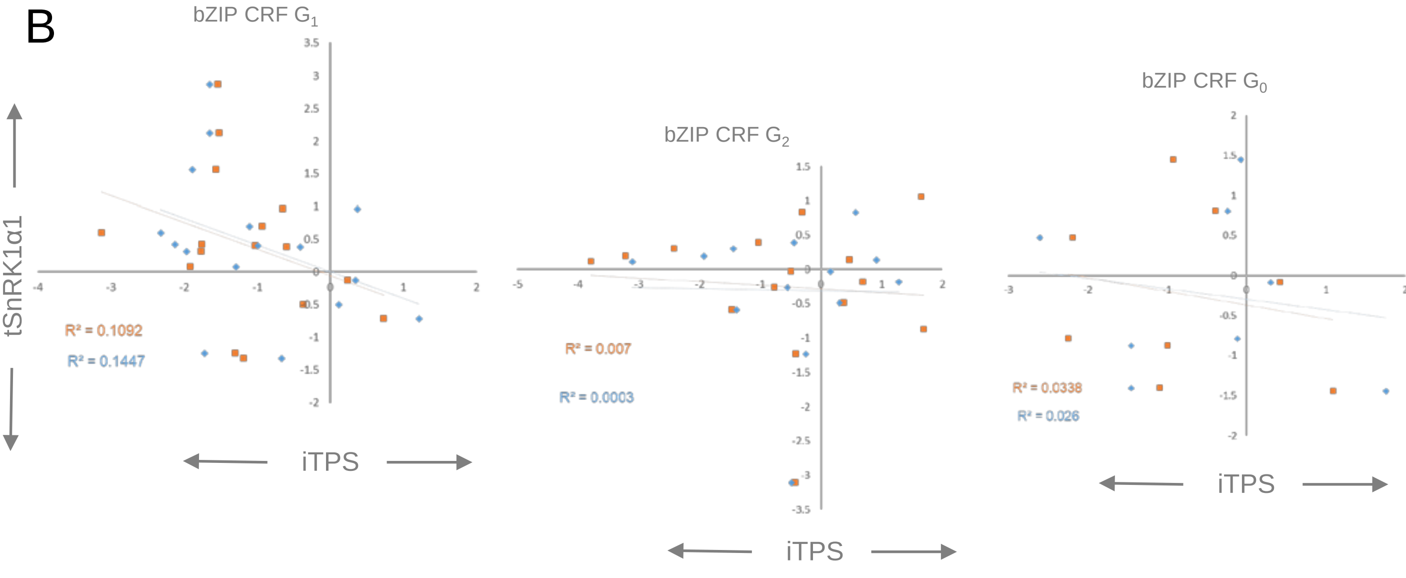

iTPS on x-axis, tSnRK1α1 on y-axis

Supplemental Figure S21: TFs that were assigned to CRF group G_1 in the iTPS response: comparison of their iTPS response and in their tSnRK1α1 response. Responses are shown for genes in different TF families (see displays) focusing on TF families with at least 5 TFs that showed a G_1 type response.
CRF is carbon response factor, FC is fold change, iTPS is response to induced expression of bacterial TPS, AP2/EREBP is APETALA2/ethylene-responsive element binding proteins; AUX/IAA is Auxin/Indole-3-Acetic Acid; DOF is DNA binding with One Finger; GATA denotes containing this amino acid sequence.

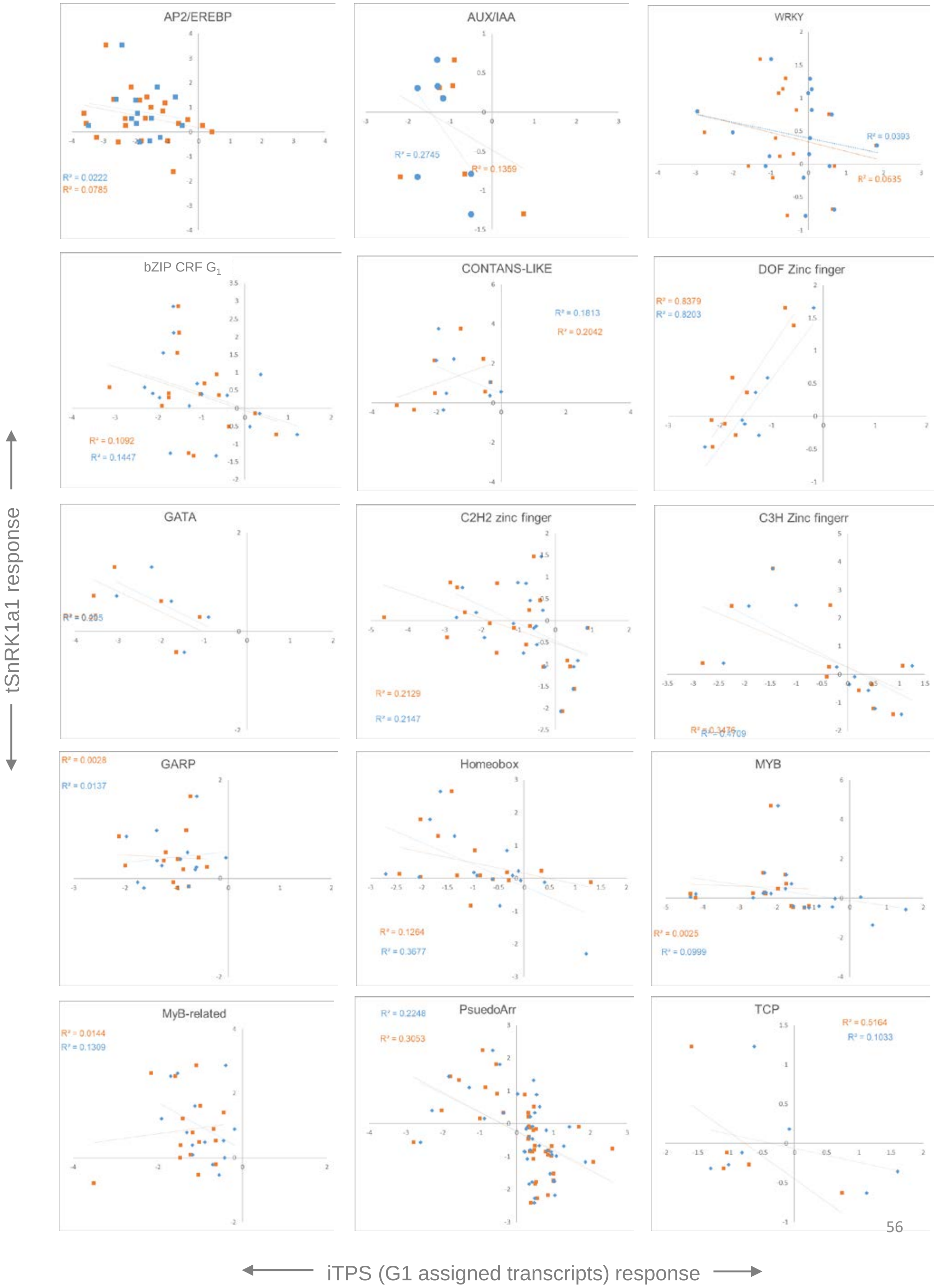

Supplemental Figure S22. Analysis of transcription factors assigned to CRF group G_2.

(A) Enrichment analysis using Gene Ontology terms. . Blue denotes fold enrichment and orange the FDR value. The nine categories at the bottom of the display were underrepresented.

FDR is false rate discovery

(B) STRING analysis, see next slide

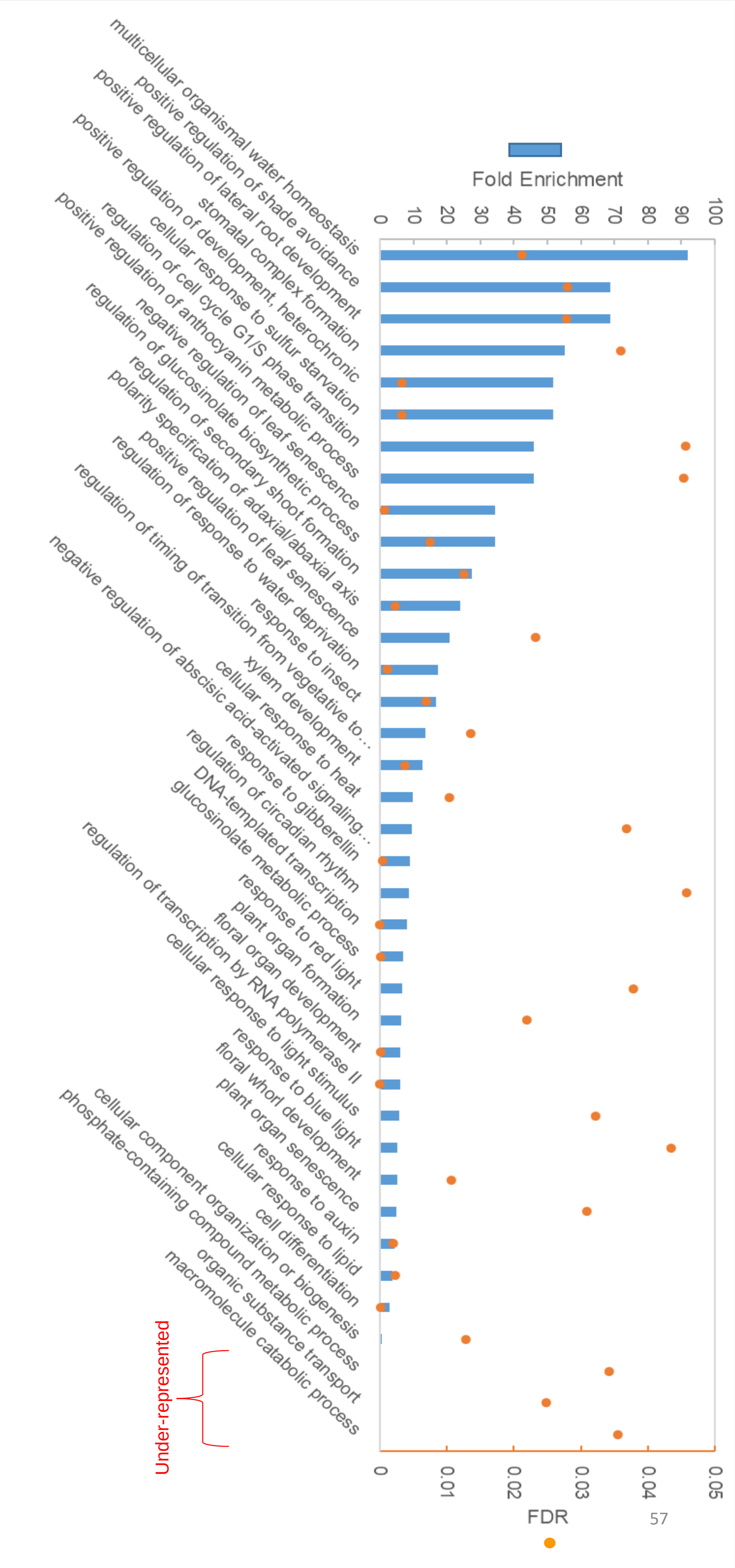

Supplemental Figure S22. Analysis of transcription factors assigned to Crf group G_2 (continued)

(B) STRING analysis. The tool utilizes available datasets, published work, automated text mining and computational predictions from various organisms to score the likelihood of association between proteins, taking into account physical (direct) and functional (indirect) interactions (Szklarczyk et al., 2021). We used the more relaxed filter for expression (FDR < 0.05 and $\log_2$ FC ≥ 0.2; 780 TFs in total) and set a high interaction confidence score filter (interaction score ≥ 0.7). The thickness of the line connecting two nodes indicates the strength of data support, with thicker lines representing higher interaction score

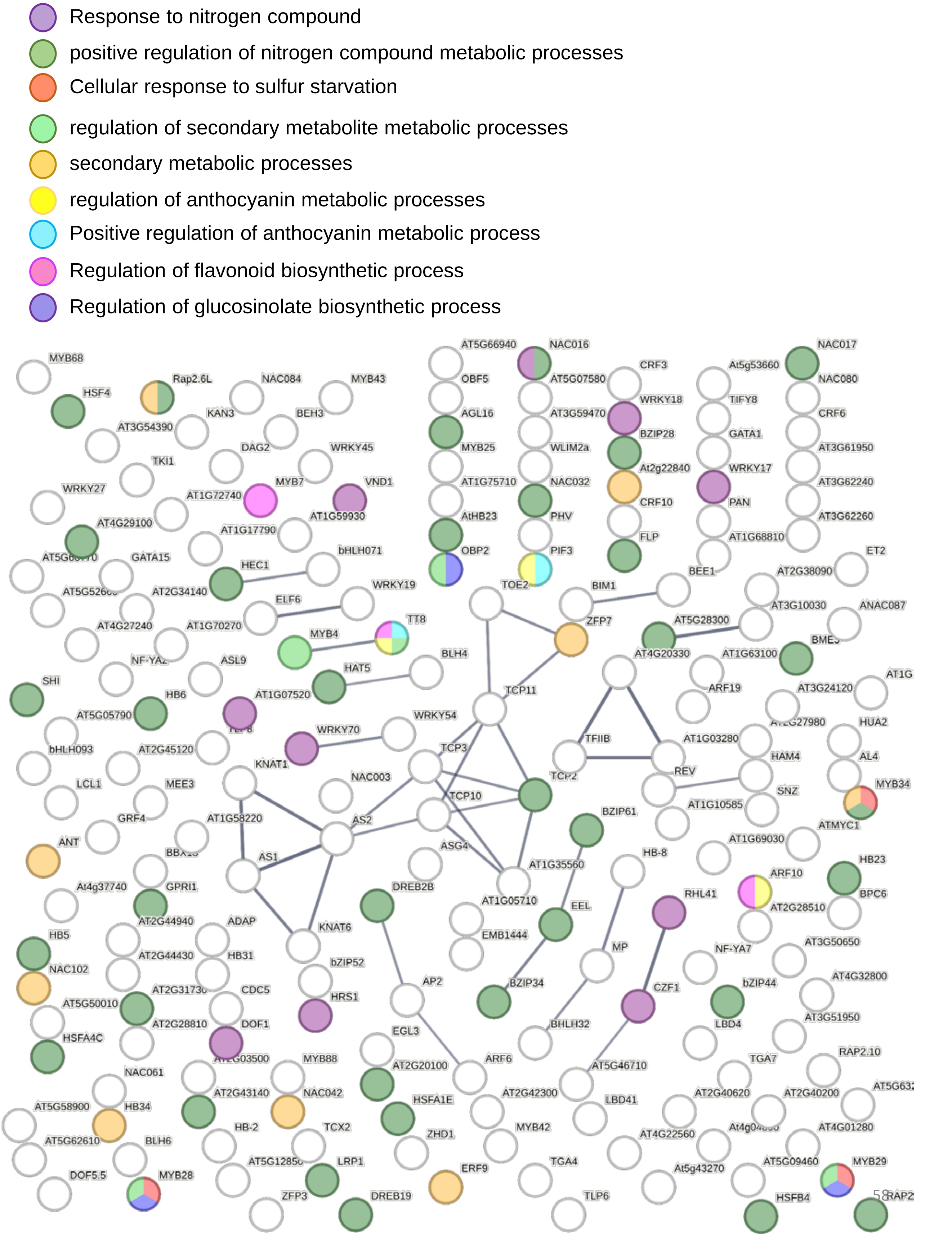

Supplemental Figure S23 Glucosinolate metabolism and its transcriptional regulation

(A) Genes involved in glucosinolate biosynthesis

(B) Transcription factors involved in the regulation of glucosinolate biosynthesis

The displays show the4-h and 6-h iTPS response, assignment of the iTPS response to CRF groups, the CRF value (defined as in Supplemental Figure S2) and the response to constitutive overexpression of TPS (oeTPS, from Zhang et al., 2009) and transient overexpression oftSnRL1α1 in protoplasts (tSnRL1α1, from Baena-Gonzalez et al., 2007). Significant changes in the iTPS response are shown with bold font. Assignment to CRF groups is performed only for significant responses and only CRF G_1 assignments are shaded. No entry for oeTPS or tSnRL1α1 indicates that the response did not pass the filter in the original publication.

CRF is carbon response factor, FC is fold change, iTPS is response to induced expression of bacterial TPS, oeTPS is constitutive overexpression of TPS

A

Gene	iTPS response						CRF	Other responses		Annotation
Code	4-h adj.p	6-h adj.p	**4-h FC log_2**	**6-h FC log_2**	4-h CRF score	6-h CRF score		oeTPS FC log_2	tSnRK1α1 FC Log_2	
At4g39950	0,00	0,00	**-2,98**	**-3,02**	2	2	1,41	2,57	-1,59	CYP79B2 (cytochrome P450, family 79, subfamily B, polypeptide 2); oxygen binding
At5g57220	0,00	0,00	**-2,69**	**-1,72**	0	0	-0,02	2,32	0,26	CYP81F2 (cytochrome P450, family 81, subfamily F, polypeptide 2); oxygen binding
At2g22330	0,00	0,00	**-2,59**	**-2,78**	2	2	0,50		0,85	CYP79B3 (cytochrome P450, family 79, subfamily B, polypeptide 3); oxygen binding
At1g65880	0,11	0,06	**-2,02**	**-4,55**			0,05		-1,53	AMP-dependent synthetase and ligase family protein
At1g65860	0,00	0,00	**-1,91**	**-2,35**	2	2	1,32		-1,06	flavin-containing monooxygenase family protein / FMO family protein
At1g18590	0,00	0,00	**-1,79**	**-2,53**	2	2	1,51		-0,82	sulfotransferase family protein
At4g03050	0,56	0,55	**-1,72**	**-1,76**			0,00		-0,99	AOP3 (2-oxoglutarate?dependent dioxygenase 3); oxidoreductase, a
At5g23020	0,00	0,00	**-1,58**	**-1,66**	2	2	1,37		-0,17	MAM-L (METHYLTHIOALKYMALATE SYNTHASE-LIKE); 2-isopropylmalate synthase
At1g62560	0,00	0,00	**-1,40**	**-2,02**	2	2	1,65		-0,59	flavin-containing monooxygenase family protein / FMO family protein
At5g23010	0,00	0,00	**-1,32**	**-1,92**	2	2	0,48		0,70	MAM1 (2-isopropylmalate synthase 3); 2-isopropylmalate synthase
At2g20610	0,00	0,00	**-1,26**	**-1,43**	2	2	0,79	1,85	-2,01	UR1 (SUPERROOT 1); transaminase
At4g13770	0,00	0,00	**-1,23**	**-1,63**	2	2	0,83	0,00	0,57	CYP83A1 (CYTOCHROME P450 83A1); oxygen binding
At1g74100	0,00	0,00	**-1,00**	**-1,03**	2	2	0,63	2,03	-1,06	sulfotransferase family protein
At1g74090	0,00	0,00	**-0,96**	**-1,65**	2	2	0,60		-0,32	sulfotransferase family protein
At2g43100	0,00	0,00	**-0,55**	**-0,91**	2	2	1,35		-0,71	aconitase C-terminal domain-containing protein
At3g58990	0,09	0,00	**-0,17**	**-1,01**		2	1,56	1,41	-0,08	aconitase C-terminal domain-containing protein
At4g31500	0,00	0,00	**-0,14**	**-0,86**	2	2	0,66	2,28	-1,69	CYP83B1 (CYTOCHROME P450 MONOOXYGENASE 83B1); oxygen binding
At4g13430	0,37	0,36	**-0,09**	**-0,15**			0,39		-0,25	aconitase family protein / aconitate hydratase family protein
At4g12030	0,00	0,00	**-0,07**	**-0,82**	2	2	1,07		0,07	ile acid:sodium symporter family protein
At1g80560	0,63	0,28	**0,05**	**-0,16**			0,29		-1,30	3-isopropylmalate dehydrogenase, chloroplast, putative

B

Gene		iTPS response						CRF	Other responses		Annotation
Name	Code	4-h adj.p	6-h adj.p	**4-h FC log_2**	**6-h FC log_2**	4-h CRF score	6-h CRF score		oeTPS FC log_2	tSnRK1α1 FC Log_2	
MYB29	At5g07690	0,00	0,00	**-2,86**	**-3,05**	2	2	0,97		-0,30	myb domain protein 29); regulates aliphatic GLS biosynthesis
MYB28	At5g61420	0,00	0,00	**-1,74**	**-1,86**	2	2	1,00		-1,31	myb domain protein 28; regulates aliphatic GLS biosynthesis
MYB76	At5g07700	0,00	0,00	**-2,03**	**-2,03**	0	0	-0,05		-0,39	myb domain protein 76; regulates aliphatic GLS biosynthesis
MYB34	At5g60890	0,00	0,00	**-4,33**	**-3,52**	2	2	1,15		-0,16	ATR1/MYB34 (ALTERED TRYPTOPHAN REGULATION); regulates indolic GLS synthesis
MYB122	At1g74080	0,00	0,20	**-2,60**	**-0,85**	0		0,01		-1,65	myb domain protein 122); regulates indolic GLS synthesis
MYB51	At1g18570	0,00	0,00	**-1,97**	**-1,16**	0	0	-0,10	2,01	-1,63	myb domain protein 51; regulates indolic GLS synthesis
MYC2	At1g32640	0,00	0,00	**-1,57**	**-1,82**	1	1	-0,56		-0,26	ATMYC2 (JASMONATE INSENSITIVE 1); DNA binding / transcription factor
MYC3	At5g46760	0,00	0,04	**-0,66**	**-0,30**	1	1	-0,39		1,34	JAZ-interacting transcription factor
MYC4	At4g17880	0,00	0,00	**0,64**	**0,75**	1	1	0,32			JAZ-interacting transcription factor thatwith MYC2 and MYC3 activatse JA-responses.
MYC5	At5g46830	0,02	0,24	**-0,93**	**-0,44**	0		0,05		-0,89	(at5g46830):basic helix-loop-helix (bHLH) family protein
OBP2	At1g07640	0,00	0,00	**-1,00**	**-1,01**	2	2	0,23		0,64	OBF BINDING PROTEIN 2; upstream regulator of indolic GLS
SR1	At2g22300	0,23	0,94	**-0,16**	**0,02**			-0,54		0,56	(at2g22300):ethylene-responsive calmodulin-binding protein, putative (SR1)
WRKY33	At2g38470	0,37	0,00	**-0,16**	**0,63**		2	-0,56	1,48	-0,12	WRKY DNA-binding protein 33; directly regulates indolic GSL biosynthesis

Supplemental Figure S24. Proposed interplay between Tre6P-mediated signaling and other C-signaling pathways in wild-type plants.

The proposed scheme is derived from Figures 5 and 7. It is based on difference between the observed overall response to induction of TPS, in which Tre6P rises but sucrose and many related other metabolites decline, and the known response in wild-type plants to increased C availability, in which Tre6P, sucrose and related metabolites rise (see Figure 1, Supplemental Figure S4, also Martins et al., 2013 and Figueroa et al., 2016, Avidan et al., 2023). For transcripts assigned to CFR group G_1, the response to rising C availability in wild-type plants is predicted to qualitatively similar to their iTPS response. in contrast, for transcripts assigned to CFR group G_2, the response to rising C availability in wild-type plants is predicted to be qualitatively opposite to their iTPS response (and similar to their CRF). Analyses summarized in Figures 5 and 7 identified processes where there was a marked bias for the underlying genes to be assigned to either CRF group G_1 or group G_2. The projected scenarios were generated by reversing the direction of change for processes that were linked to CRF group G_2 and whose observed iTPS response is therefore likely to be driven by the decease of sucrose and related metabolites.

A. Responses of metabolism and growth- and defense-related processes to a rising C supply in wild-type plants., and the contribution of Tre6P and other C-signaling pathways to this response.

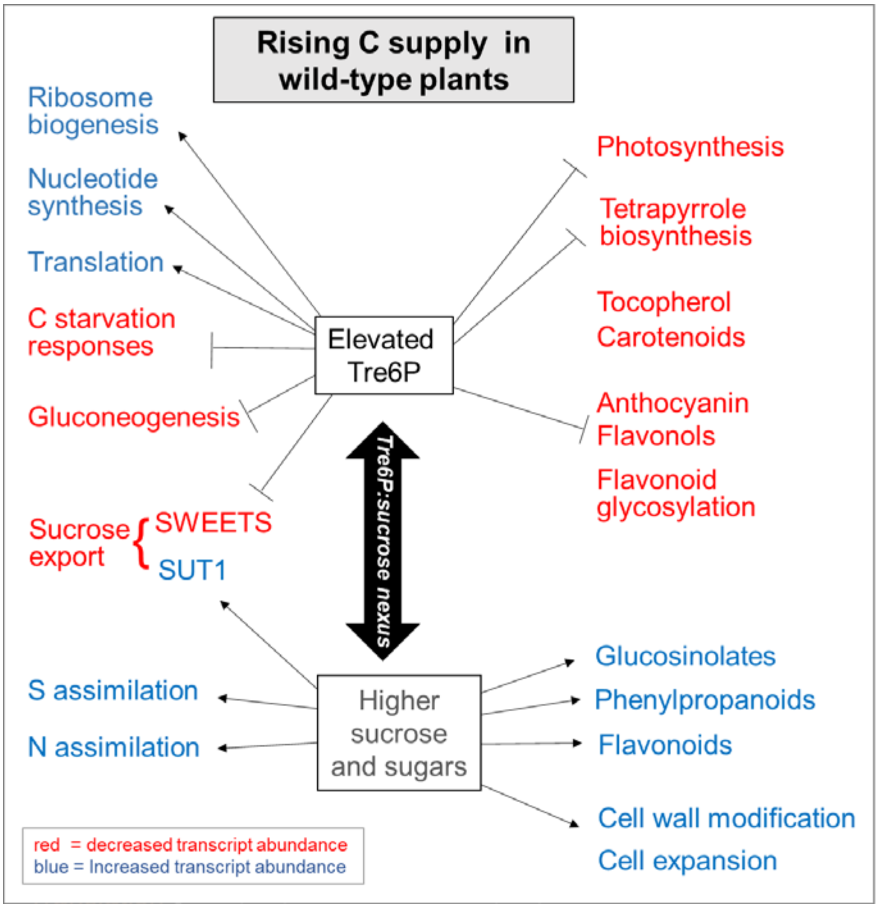

B. Responses of genes involved in Tre6P metabolism and signaling and in the SnRK1 complex. Genes assigned to CRF group G_0 are unlikely to respond to rising C availability, and if they do, not in a predicable way, and are colored black

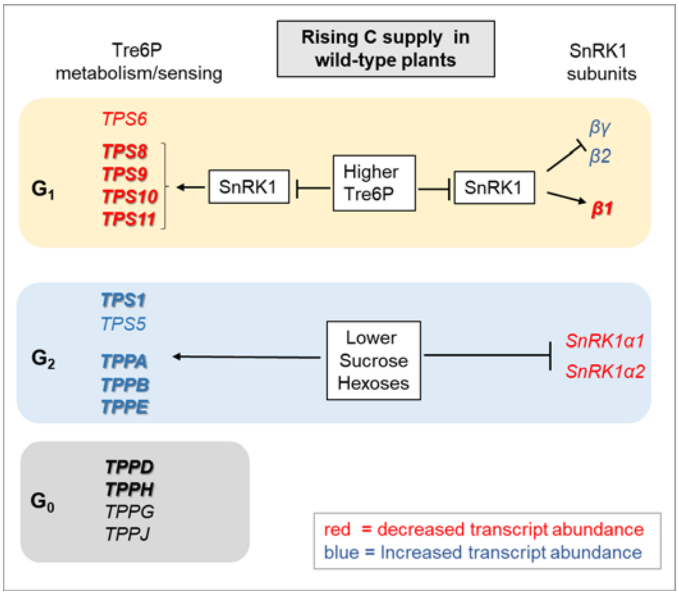

Supplemental Table S1. List of transcriptome data sets. The Table summarizes microarray and RNA-sequencing (RNAseq) datasets used in this study, with a description of treatments and transgenic lines with number of differentially expressed genes (DEGs) in each. The ATH1 datasets for iTPS 29.2, iTPS 31.3 lines and alcR lines are from this study. They are for treatments similar to those described in Martins *et al*., (2013) and were either sprayed at dawn and harvested 12 h later at the end of the day (ED) or were sprayed at dusk and harvested 12 h later at the end of the night (EN). These data are provided in Supplemental Dataset S1, and analyzed in Supplemental Figures S1 and S3 and Supplemental Tables S2-S4. The RNA seq data using iTPS 29.2 are for iTPS 29.2 and alcR lines from this study. They are for plants sprayed 0.5 h after dawn and harvested 4 or 6 h later, respectively. Whilst a maximum of about 33K transcripts can be detected, the number of transcripts detected across lines and treatments in our study was about 23.8K. These data are provided in Supplemental Dataset S4 and analysed in Figures 2-8, Supplemental Figures S5-S24, and Supplemental Tables S4-S6. oeTPS refers to a constitutive overexpression of bacterial TPS, published in Zhang et al., (2009). The downloaded data are analyzed in Figure 6, Supplemental Figure S12 and Supplemental Table S5. tSnRK1α1 efers to transient overexpression in protoplasts, published in Baena-Gonzales et al. (2007), respectively. The downloaded data are analyzed in Figure 8, Supplemental Figure S14 and Supplemental Table S6. oebZIP11 refers to transient over expression of bZIP11 for 2 h in 7 days old published in Hanson et al. (2008) and Ma et al. (2011). For the published datasets, data was provided only for transcript that showed a significant change, as scored in the publication. The value under ‘total genes detected’ refers to the number of features on the array, and is given as NN* as the actual number detected will have been lower. h – hours, d – days.

Line	Dataset	Tissue analyzed	Age or time after induction	Total genes detected	DEGs FDR<0.05	Up (%)	Down (%)	DEGs FDR<0.05 and FC>2	Up (%)	Down (%)
iTPS 31.3	ATH1	whole rosette	ED	22.7K	290	62 (21.4)	228 (78.6)	68	9 (13.2)	59 (86.8)
iTPS 31.3	ATH1	whole rosette	EN	22.7K	396	198 (50)	198 (50)	76	24 (31.6)	52 (68.4)
iTPS 29.2	ATH1	whole rosette	ED	22.7K	1459	684 (46.9)	775 (53.1)	189	43 (22.8)	146 (77.2)
iTPS 29.2	ATH1	whole rosette	EN	22.7K	223	104 (46.6)	119 (53.4)	92	30 (32.6)	62 (67.4)
iTPS 29.2	RNA seq	whole rosette	4 h	23.8K	13400	6530 (48.7)	6870 (51.3)	5618	2066 (36.8)	3552 (63.2)
iTPS 29.2	RNA seq	whole rosette	6 h	23.8K	13109	10934 (83.4)	2175 (16.6)	5437	2017 (37.1)	3420 (62.9)
oeTPS	ATq3.6.2	seedling	7 d	29K*	-	-	-	5272		
tSnRK1α1	ATH1	leaf protoplasts		24K*	-	-	-	1021		
oebZIP11	ATH1	seedling	7 d, 2 h	24K*	-	-	-	236		

Supplemental Table S2. Changes in transcript abundance 12 h after induction of TPS, investigated at either ED or EN.

iTPS 29.2, iTPS 31.1 and alcR were either sprayed at dawn and harvested 12 h later at the end of the day (ED) or were spayed at dusk and harvested 12 h later at the end of the night (EN).

The upper part summarizes the total number of shared DEGS (Filter: FDR<0.05 only, no fold-change cutoff between line 29.2 and line 31.3 at ED and EN), and the correlation between the response in the two lines at ED and at EN.

In the middle part, the assignment of transcript to GRF groups G_1, G_2 and G_O (see Supplemental Figure S2) are shown separately for line 29.2 and line 31.3, and the correlation between the change in transcript abundance and the CRF value in the sets of transcripts assigned to G_1 G_2 and G_0

The lower part summarizes the % of DEGS assigned to $G_1$and G_2 at ED and EN.

Correlation between iTPS 29.2 and iTPS 31.3 for the response of all shared DEGs

Line	Lines	CRF	treatment	Number of DEGs	R^2	Slope direction	p value
iTPS	29.2 vs 31.3	-	ED	254	0.9565	+	1.35x10 $^{-173}$
iTPS	29.2 vs 31.3	-	EN	172	0.9821	+	2.23x10 $^{-150}$
iTPS	29.2 vs 31.3	G_1	ED	138	0.969	+	4.12x10 $^{-104}$
iTPS	29.2 vs 31.3	G_2	ED	88	0.925	+	4.46x10 $^{-49}$
iTPS	29.2 vs 31.3	G_0	ED	30	0.977	+	1.47x10 $^{-24}$
iTPS	29.2 vs 31.3	G_1	EN	43	0.9781	+	1.16x10 $^{-35}$
iTPS	29.2 vs 31.3	G_2	EN	108	0.9837	+	1.22x10 $^{-96}$
iTPS	29.2 vs 31.3	G_0	EN	21	0.9895	+	2.99x10 $^{-20}$

Correlation between the C response factor (CRF) and the iTPS response of a given line after assigning transcripts to G_1, G_2 or G_0

Line	Line	CRF	tireatment	Number of DEGs	R^2	Slope direction	p value
iTPS	29.2	G_1	ED	706	0.67	+	5.4x10 $^{-172}$
iTPS	29.2	G_2	ED	327	0.656	-	1.73x10 $^{-77}$
iTPS	29.2	G_0	ED	420	0.019		0.005
iTPS	29.2	G_1	EN	53	0.76	+	2.13x10 $^{-17}$
iTPS	29.2	G_2	EN	127	0.754	-	6.2x10 $^{-40}$
iTPS	29.2	G_0	EN	43	0.0001		0.95
iTPS	31.3	G_1	ED	166	0.645	+	8.9510x10 $^{-39}$
iTPS	31.3	G_2	ED	91	0.435	-	1.1x10 $^{-12}$
iTPS	31.3	G_0	ED	33	0.004		0.732
iTPS	31.3	G_1	EN	114	0.69	+	2.63x10 $^{-30}$
iTPS	31.3	G_2	EN	200	0.684	-	2.21x10 $^{-51}$
iTPS	31.3	G_0	EN	82	0.035		0.09

	**% of DEGs in G_1**		**% of DEGs in G_2**	
	iTPS 29.2	iTPS 31.1	iTPS 29.2	iTPS 31.1
ED	48		23	
EN	24		57	

Supplemental Table S3. Summary of the number of differentially expressed genes, their assignment to CRF groups and the R^2, direction and p-value of the relationship between the change in transcript abundance and the CRF value (extends Figure 2 and Supplemental Figure S5A-B).

The Table lists the numbers of DEGs (FDR < 0.05) that were assigned to CRF groups G_1, G_2 and G_O for the 4-h and the 6-h data sets, as well as for transcript that showed a shared response and alignment in both the 4-h and 6-h data sets (termed 4-6h shared). The last three columns show the R_2 value, the slope direction and the p-value when, for that set of genes, the change in transcript abundance is plotted against their CRF value.

				Plot of change in transcript abundance against CRF		
**Line**	**CRF**	**Time after induction**	**Number of DEGs**	**R^2**	**Slope direction of plot**	**p value**
iTPS 29.2	G_1	4 h	4576	0.45	+	0
iTPS 29.2	G_2	4 h	2887	0.35	-	2.11×10^{-274}
iTPS 29.2	G_0	4 h	3573	0.002		0.0067
iTPS 29.2	G_1	6 h	4470	0.48	+	0
iTPS 29.2	G_2	6 h	2969	0.37	-	2.17×10^{-296}
iTPS 29.2	G_0	6 h	3636	0.0007		0.11
iTPS 29.2	G_1	4-6 h shared	3769	0.92	+	0
iTPS 29.2	G_2	4-6 h shared	2338	0.90	+	0
iTPS 29.2	G_0	4-6 h shared	2890	0.92	+	0

Supplemental Table S4. Pairwise comparison of responses 4-h and 6-h post-induction in the light, and in the ED and the EN treatments (extends Supplemental Figure S5C).

Pairwise comparisons of genes assigned to different CRF groups are made between all treatments. Data is plotted for transcripts that showed a significant change in abundance in at least one of the treatments (FDR < 0.05, Log_2 FC ≥ 0.2). The two treatments that are compared are shown in the two left-hand columns The next two columns identify which treatment was used to filter, and which CRF group is compared. Transcripts were assigned to CRF group 1 when their response to elevation of Tre6P was in the same direction as their response to an increase of sugar supply in a panel of treatments (as defined by the CRF); transcripts assigned to CRF group 2 respond to elevation of Tre6P in the opposite direction to the response to an increase of sugar supply; transcripts assigned to CRF group 0 do not show any notable response to a change in sugar in the panel of treatments (for details see Supplemental Figure S2).

		Filtered by:	Filtered by :				
**Treatment a**	**Treatment b**	**treatment a**	**treatment b**	**Number of DEGs**	**R^2**	**p-value**	**Slope direction**
iTPS (4h)	iTPS (ED)	G_1		451	0.632	$8.2x10^{-101}$	+
iTPS (4h)	iTPS (ED)		G_1	519	0.413		+
iTPS (4h)	iTPS (ED)	G_2		287	0.191	$1.1x10^{-15}$	+
iTPS (4h)	iTPS (ED)		G_2	244	0.403		+
iTPS (4h)	iTPS (ED)	G_0		202	0.022	0.024	+
iTPS (4h)	iTPS (ED)		G_0	197	0.018		+
iTPS (6h)	iTPS (ED)	G_1		491	0.723	$1.1x10^{-138}$	+
iTPS (6h)	iTPS (ED)		G_1	528	0.574		+
iTPS (6h)	iTPS (ED)	G_2		275	0.355	$7.3x10^{-29}$	+
iTPS (6h)	iTPS (ED)		G_2	240	0.574		+
iTPS (6h)	iTPS (ED)	G_0		205	0.055	0.00068	+
iTPS (6h)	iTPS (ED)		G_0	205	0.055		+
iTPS (4h)	iTPS (EN)	G_1		50	0.530	$5.4x10^{-10}$	+
iTPS (4h)	iTPS (EN)		G_1	48	0.315		+
iTPS (4h)	iTPS (EN)	G_2		100	0.408	$1.3x10^{-13}$	+
iTPS (4h)	iTPS (EN)		G_2	108	0.517		+
iTPS (4h)	iTPS (EN)	G_0		33	0.149	0.026	+
iTPS (4h)	iTPS (EN)		G_0	34	0.169		+
iTPS (6h)	iTPS (EN)	G_1		59	0.519	$1.2x10^{-10}$	+
iTPS (6h)	iTPS (EN)		G_1	49	0.533		+
iTPS (6h)	iTPS (EN)	G_2		103	0.560	$2.2x10^{-19}$	+
iTPS (6h)	iTPS (EN)		G_2	114	0.573		+
iTPS (6h)	iTPS (EN)	G_0		33	0.297	0.001046	+
iTPS (6h)	iTPS (EN)		G_0	33	0.297		+

Supplemental Table S5: Comparison of the responses to constitutive overexpression of TPS (oeTPS) and induced overexpression of TPS (iTPS) (supplemental to Figure 6 and Supplemental Figure S12). The oeTPS response (from Zhang *et al.*, 2009) was filtered by the authors (FDR<0.05, FC≥2), The Tables show the number of DEGs shared DEGS with the iTPS response, after filtering and assigning genes in the iTPS response to CRF groups.

(A) Comparison of the oeTPS response with the overall iTPS response, either unfiltered or after filtering showing numbers of shared genes and p-value of the regression

(B) Comparison of the oeTPS response with the TPS response after filtering it (FDR<0,05) and assigning genes in the iTPS response to the CRF groups G_1, G_2 or G_0. The Table summarizes the % of genes in the oeTPS response that were assigned to a given CRF group (for details of assignment to CRF groups, see Supplemental Figure S2).

A. · Comparison of the oeTPS response with the overall iTPS response, either unfiltered or after filtering showing numbers of shared genes and p-value of the regression

Lines	ALL	p Value	FDR < 0.05	p Value	FDR < 0.05 + FC2	p Value
4h vs oeTPS	5005	1.41×10^{-91}	3340	8.26×10^{-95}	1321	5.59×10^{-32}
6h vs oeTPS	4988	1.41×10^{-149}	3353	1.1×10^{-144}	1372	4.7×10^{-59}

B. Comparison of the oeTPS response with the TPS response after filtering it (FDR<0,05) and assigning genes in the iTPS response to the CRF groups G_1, G_2 or G_0. The tables summaries the % of genes in the oeTPS response that were assigned to a given CRF group (for details of assignment to CRF groups, see Supplementary Figure S2)

G1		DEGs assigned to G_1 in iTPS and are present also in oeTPS	
**# shared iTPS**	**Comparisons**	**#**	**%**
	4h vs oeTPS	1837	40.1
	6h vs oeTPS	1890	42.3
3769	4-6h vs oeTPS	1596	42.3

G2		DEGs assigned to G_2 in iTPS and are present also in oeTPS	
**# shared iTPS**	**Comparisons**	**#**	**%**
	4h vs oeTPS	612	21.2
	6h vs oeTPS	619	20.8
2338	4-6 vs oeTPS	484	20.7

G0		DEGs assigned to G_0 in iTPS and are present also in oeTPS	
**# shared iTPS**	**Comparisons**	**#**	**%**
	4h vs oeTPS	406	14.1
	6h vs oeTPS	450	15.2
2890	4-6h vs oeTPS	347	12.0

Supplemental Table S6. Comparison of the tSnRK1α1 and iTPS responses (extends Figure 8 and Supplemental Figure S14).

The Table shows the relationship between all 1005 genes from the tSnRK1α1 data set (FDR < 0.05, FC ≥ 2) that were retrieved in the unfiltered iTPS data set (ALL), and the relationship for the subsets of genes from the tSnRK1α1 response that were retained in the filtered iTPS response (FDR < 0.05, Log_2 FC ≥ 0.2) and assigned to the CRF groups G_1, G_2 and G_0 (for details of assignments to CRF groups, see Supplemental Figure S2).

Comparison	CRF in the iTPS response	Number of DEGs	R^2	p Value
iTPS 4h vs tSnRK1α1	ALL	1005	0.140	7.63×10^{-35}
	G_1	580	0.644	7×10^{-132}
	G_2	144	0.415	2.9×10^{-18}
	G_0	22	0.042	0.36
iTPS 6h vs tSnRK1α1	ALL	1005	0.212	6.97×10^{-54}
	G_1	541	0.677	1.64×10^{-134}
	G_2	151	0.286	1.44×10^{-12}
	G_0	22	0.074	0.22
