# Supplementary material for: Direct and indirect responses of the Arabidopsis transcriptome to an induced increase in trehalose 6-phosphate
Source: Plant Physiol. 2024 Apr 9;196(1):409–31. doi: 10.1093/plphys/kiae196 (PMC11376379; doi:10.1093/plphys/kiae196)
Supplement: kiae196_Supplementary_Data [file kiae196_supplementary_data.zip › Supplemental Text.pdf]

Avidan et al. Direct and indirect responses of the Arabidopsis transcriptome to an induced increase in trehalose 6-phosphate

Supplemental Text. Analysis of selected areas of metabolism, signaling and development

Subsection	Page
AlcR inducible expression system	***2***
Initial experiment with harvest 12 hours post-induction	***2***
MapMan BINS related to metabolism	***3***
Photosynthesis	*3*
Gluconeogenesis	*3*
N metabolism	*3*
Nucleotide metabolism	*4*
Specialized metabolism	*4*
Sucrose metabolism and transport	***5***
Protein synthesis, ribosomal proteins and ribosome biogenesis	***6***
Cell wall synthesis and modification; cell expansion	*7*
Flowering induction	***8***
Circadian clock components	***9***
Light signaling	***11***
Expansins and Xyloglucan endotransglucosylases	***11***
TPSs and TPPs	***12***
Expression of TOR subunits	***12***
Shared targets with post-translational TOR signaling	***12***
Comparison with the transcriptional response to inhibition of TORC	***14***
Finger-Like Zinc (FLZ) family proteins	***18***
Overlap with bZIP11 signaling	***19***
Transcription factors	***22***
Direct and indirect impact on photosynthesis, the C/N balance and growth	***26***
Direct and indirect impacts on light, circadian and ABA signaling	***27***
References	***29***

AlcR inducible expression system

The *Escherichia coli otsA* gene was expressed under the control of the *Aspergiluus nidulans* alcR/alcA promoter system developed by Caddick et al. (1998) for ethanol-inducible gene expression in plants, as described in Martins et al. (2013). The transgenic locus in the iTPS plants includes a *pro-35S:alcR:t-nos* construct for constitutive expression of the AlcR ethanol-binding transcription factor, and a *pro-alcA:otsA:t-ocs* construct for expression of the *E. coli* OtsA (TPS) protein when the AlcR transcription factor binds to the *alcA* promoter in the presence of ethanol. The AlcR control plants (Martins et al., 2013) contain the *pro-35S:alcR:t-nos* gene construct and an empty *pro-alcA:[...]:t-ocs* cassette, resulting in constitutive expression of the AlcR protein that should bind unproductively to the *alcA* promoter in the presence of ethanol. In both iTPS and AlcR plants, the transgenic locus contains the selection marker *nptII.*

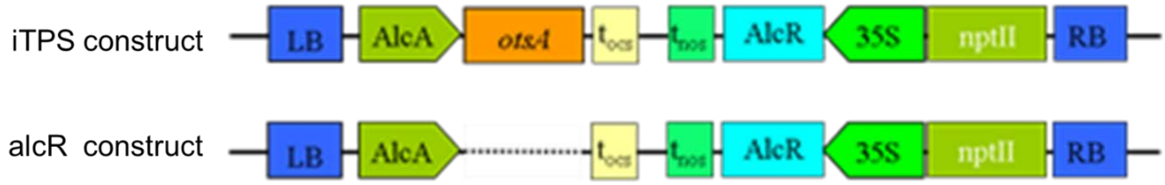

Initial experiment with harvest 12 hours post-induction

In a first experiment, we investigated if the response was similar in two separate iTPS lines, iTPS29.1 and iTPS321.3. This initial experiment was carried out using 12-h induction and also served to show that shorter treatments were needed and that induction should be carried out in the light period, because indirect effects predominated, especially during the night.

iTPS lines 29.2 and 31.3 and control alcR line plants were sprayed with 2% ethanol or water at the beginning of the day and harvested 12 h later at the end of the day (ED treatment) or were sprayed at dusk and harvested 12 h later at the end of the night (EN treatment). In published data for metabolite levels in the same plant material, after ethanol induction Tre6P levels increased 3- to 4-fold at ED and 2-to 3-fold at EN compared to the control treatments (Martins et al., 2013, see also Figueroa *et al.,* 2016 for separate experiments). Transcript abundance was analyzed using the Affymetrix ATH1 array (Supplemental Dataset S1). The ethanol-sprayed iTPS line was compared to the corresponding water-sprayed iTPS control to calculate the fold-change (FC) for each transcript. These were corrected for minor responses to alcR (see Methods). We termed the resulting change in transcript abundance the ‘iTPS response’. Line 29.1 showed a stronger response than line 31.3 in the ED treatment, and a similar response to line 31.3 in the EN treatment (Supplemental Figure 1A, Supplemental Table S1). For shared genes, the responses in the two lines were strongly correlated

(Supplemental Figure S1A-C, Supplemental Table S2, R^2 = 0.96 and 0.98 for FDR<0.05 filtered data sets at ED and EN, respectively).

Further data analyses were performed with the data for line 29.1. When the iTPS response was compared with CRF values (a measure for how the transcript responds to an increase in the sugar supply; for explanation and calculation, see main test and Supplemental Figure S2) across all transcripts there was little similarity in the ED treatment and even less in EN treatment (Supplemental Figure S3A-B). Transcripts assigned to G_1 showed a strong positive correlation between their CRF values and iTPS response (R^2 = 0.67 and 0.76 at ED and EN, respectively) consistent with them responding to elevated Tre6P (Supplemental Figure S3C-D, Supplemental Table S2). Transcripts assigned to G_2 showed a strong negative correlation between their CRF values and iTPS response, consistent with them responding to the decrease in sugars (Supplemental Figure S3C-D, Supplemental Table S2). There was a strong correlation between the response at ED and EN of transcripts that were assigned to G_1, or to G_2, or to G_0 (Supplemental Figure S2E).

A larger proportion of transcripts was assigned to G_1 and a smaller proportion to G_2 at ED than at EN (Supplemental Table S2). This might reflect the larger decrease of sugars in the EN than the ED treatment. At night, the sucrose pool is strongly dependent on starch mobilization and falls because elevated Tre6P leads to a strong inhibition of starch mobilization (Martins et al. 2013; dos Anjos et al. 2018). In the light period, elevated Tre6P leads to diversion of more of the fixed C to organic acid and amino acid synthesis, but the sucrose pool is partly stabilized by photosynthetic C fixation and possibly by changes in sucrose export (Figueroa et al. 2016, see also below).

This initial experiment with harvest 12-h post induction led us to focus on the iTPS response in the light period, to harvest at earlier times post-harvest and to focus on line 29.2.

The following sections provide more information for the experiment in which iTPS29.1 and the control alcR line were sprayed with ethanol or water 0.5h after dawn and sampled 4h and 6h later for RNAseq analysis (see main text, Figures 1-2 for an analysis of metabolite levels, global changes in gene expression and assignment to CRF groups G_1, G_2 and G_0). We compared the responses of transcripts assigned to G_1, G_2 and G_0 in the RNAseq experiment with those in the ED and EN treatments (Supplemental Figure S5C, Supplemental Table S4). There was relatively good agreement, especially for transcripts that were repressed. Discrepancies may reflect genes that respond either transiently or slowly to an induced rise in Tre6P

MapMan BINS related to metabolism

As highlighted in the main text and Figure 3, analysis of the iTPS response in the RNAseq experiment at the highest level of the MapMan ontology (BINs) and using a gene set that was filtered to focus on genes that showed a ≥2-fold change in transcript abundance revealed that genes assigned to CRF groups G_1, G_2 and G_0 were often associated with different functions.

MapMan BINs group genes that participate in a given metabolic sector or cellular function, irrespective of whether they are involved in biosynthesis or catabolism. Some BINs group different processes (e.g., 'Cell wall' includes biosynthesis of cell wall components, but also their modification and degradation; 'Protein' includes protein synthesis, protein modification and protein degradation). We therefore inspected the responses in selected BINs at higher resolution (Supplemental Figure S7) to provide further support for the separate identify of the G_1 response (Tre6P-mediated) and G_2 response (mediated by the decline in sucrose or other indirect effects). This analysis was also performed to learn if the G_0 response (respond to iTPS but not to changes in sugar availability) was associated with specific functions. We performed these additional PageMan analyses with a lower cut-off ($\log_2$ FC ≥ 0.2) than that used for Figure 3 (FC ≥ 2). This was done because a lower filter can increase the sensitivity to detect coordinated responses.

We first re-analyzed the response at the highest level of the MapMan ontology, using the weaker cut-off filter (Supplemental Figure S7A). The re-analysis confirmed the responses seen in Figure 3. In several cases like photosynthesis, gluconeogenesis/glyoxylate cycle, the OPP pathway, mitochondrial electron transport/ATP synthesis, tetrapyrrole synthesis) the responses of genes assigned to CRF group G_1 (i.e., genes responding to elevated Tre6P) was even stronger. As explained in the legend of Supplemental Figure S7A, application of a strong FC filter before performing the PageMan analysis can mask or attenuate coordinated responses. This is because all individual values that lie below the cutoff are set to zero before calculating the average response for the BIN or subBIN. This procedure will depress the average change when a set of genes is showing a qualitatively coordinate response but some of the responses are quantitatively below the imposed cut-off. The similar or stronger average response of genes assigned to G_1 in the analysis with the lower cut-off confirms that genes involved in photosynthesis, gluconeogenesis/glyoxylate cycle, mitochondrial electron transport/ATP synthesis, tetrapyrrole synthesis are repressed, and that this is occurring in a coordinated manner by elevated Tre6P. Further BINs that were repressed in a coordinated manner included metal handling, redox, hormone metabolism and polyamine synthesis. These BINs also showed responses in the CRF group G_2, showing that whilst some genes in these BINs are responding to elevated Tre6P many others are affected in an indirect manner.

Photosynthesis: Resolving the photosynthesis BIN into subBINS revealed that many genes encoding proteins involved in the light reactions, electron transport, photorespiration and the Calvin-Benson cycle were assigned to G_1 and repressed (Supplemental Figure S7B). This indicates that elevated Tre6P drives a broad transcriptional repression of photosynthesis. In the section ‘Direct and indirect impact on photosynthesis, the C/N balance and growth’ of the Supplemental text, we argue that this provides strong evidence that the well-known repression of photosynthetic gene expression by sugar is not just due to changes in the N status and N-signaling but includes an input from C-signaling, mediated at least partly by Tre6P.

Gluconeogenesis: Genes involved in the glyoxylate cycle and gluconeogenesis were assigned to G_1 and repressed, with the exception of isocitrate lyase that was assigned to G_0 and induced (Supplemental Figure S7C). It is not clear why isocitrate lyase shows such a divergent response.

N metabolism: A more complex picture was found for N metabolism (Supplemental Figure S7D). This BIN includes genes involved in nitrate and ammonium assimilation, which are typically induced by sugar (Vincentz et al. 1993; Krapp et al. 1995; Wang et al. 2000; Coruzzi 2003; Usadel et al. 2008, Vidal et al. 2020, see also the CRF values in Supplemental Dataset S4). These genes were assigned to G_2 and repressed in the iTPS response, implying that repression is not mediated by Tre6P (or at least, not primarily mediated by Tre6P) but is, instead, an indirect response to low sugar.

Glutamate dehydrogenases (GDHs) are induced by low sugar (Osuna et al. 2007; Jean-Xavier et al. 2012; Cookson et al. 2016) and contribute to recycling of C skeletons from amino acids (Coruzzi 2003; Fontaine et al. 2012). They were repressed in iTPS and assigned to G_1, indicating that they are repressed by Tre6P, resembling genes involved in gluconeogenesis (see above). Indeed, the major function of GDHs may be in recycling of C skeletons under low-C conditions.

Nucleotide metabolism: In nucleotide metabolism (Supplemental Figure S7E), genes involved in biosynthesis and, to a greater extent, genes involved in purine biosynthesis were mainly assigned to CRF group G_1 and induced, whereas genes involved in nucleotide salvage or breakdown, in equilibration of nucleotides or in synthesis of deoxyribonucleotides were assigned to various CRF groups and showed mixed responses.

Secondary metabolism: In the secondary metabolism BIN (Supplemental Figure S7F), genes involved in tocopherol and carotenoid biosynthesis were partly assigned to G_1 and repressed (resembling the repression of genes encoding photosynthesis proteins, see Supplemental Figure 7B) and partly to G_0

and repressed (resembling the response for tetrapyrrole biosynthesis, see Figure 3 and Supplemental Figure S7A).

This contrasted with genes involved in phenylpropanoid biosynthesis, flavonoid biosynthesis and, even more strikingly, glucosinolate biosynthesis that were assigned to G_2 and repressed, presumably as a response to the decline in sugars. The responses of transcription factors involved in the regulation of glucosinolate biosynthesis are presented later in the supplemental text, section 'Transcription factors'.

Sucrose metabolism and transport

Given that Tre6P is known to regulate sucrose metabolism (see Introduction, also Figure 1), we inspected the responses of individual genes involved in sucrose metabolism and transport (Supplemental Figure S8).

There were complex changes in expression of members of the sucrose phosphate synthase (SPS) family that is involved in sucrose synthesis, and members of the sucrose synthase (SUS) and invertase (INV) families that are involved in sucrose degradation. Responses in G_1 included a weak induction of *SPS1* and stronger repression of *SPS4*, induction of *SUS1* and repression of *SUS2*, and repression of *VINV1*, *A/N-InvC*, *A/N-InvE*, and *A/N-invH*.

There were widespread changes for sucrose-H^+ transporter (SUT) and the sugar efflux SWEET family members (for background on their functions see Xue et al. 2022; Braun 2022). *SUT1/ SUT/At1g 22710*, the sucrose/H^+ transporter that catalyzes active uptake of sucrose from the apoplast into companion cells in leaves, was weakly repressed at 6h. There were mixed responses for other *SUT* family members. There was a large decrease in transcript abundance for *SWEET11* and *SWEET12*, which mediate passive efflux of sucrose from the phloem parenchyma cells into the apoplast, and *SWEET13*, which mediates passive export of sucrose from bundle sheath cells into the apoplast, with *SWEET11* and *SWEET12* being assigned to G_1 indicating they are repressed by Tre6P-signaling, and *SWEET13* being assigned to G_2 indicating an indirect response.

It might be noted that *SWEET12* was previously reported to be induced by constitutive overexpression of bacterial TPS in Arabidopsis (Zhang et al. 2009). However, it was reported to be repressed by vascular tissue-specific overexpression of TPS in Arabidopsis (Fichtner et al. 2021) and by supplying a caged Tre6P precursor to wheat shoots (Oszwald et al. 2018). Later in the Supplemental text, possible reasons for these divergent responses are given (section 'Overlap with bZIP11 signaling') and a possible biological function is proposed (section 'Direct and indirect impact of an induced increase in Tre6P on photosynthesis, the C/N balance and growth').

Many other *SWEETs* were also repressed, including *SWEET16* and *SWEET17*, with the former responding in a way consistent with it being repressed by Tre6P. *SWEET16* and *SWEET17* are located in the tonoplast and implicated in vacuolar storage of fructose (Guo et al. 2013; Chardon et al. 2013; Klemens et al2013). Their repression might decrease recycling of fructose from the vacuole in C-replete conditions.

SWEET proteins have been implicated in the regulation of sink-source interactions by FLOWERING LOCUS T (FT) homologs in potato (Aberlanda et al. 2019) and SWEET10 has been implicated in the induction of flowering by FT in Arabidopsis (Andrés et al. 2022) *SWEET10* transcript abundance was not significantly altered in the iTPS response (Supplemental Figure S8)

Protein synthesis, ribosomal proteins and ribosome biogenesis

The BIN 'Protein' represents a very large set of genes with diverse functions, including not only genes for protein synthesis but also for post-translational modification and for protein degradation. To focus better on the process of protein synthesis, we inspected the subBINs for amino acid activation and the translation process, ribosomal proteins and ribosome biogenesis

Genes involved in amino acid activation and translation (initiation, elongation, release) were mainly assigned to G_1 and induced (Supplemental Figure S7G). Genes encoding cytosolic and mitochondrial ribosomal proteins were assigned to G_1 and broadly induced (Figure 4A, Supplemental Figure S7G), whereas genes encoding chloroplast ribosomal proteins were assigned to G_1 and often repressed at 6h (Supplemental Figure S7G), reminiscent of the response of genes involved in photosynthesis.

The response of genes involved in ribosome biogenesis is shown in Figure 4, and Supplemental Figure S7G. Figure 4A shows a PageMan analysis with a cut-off of FC ≥ 2, and Supplemental Figure S7G shows a PageMan analysis with a cut-off of $\log_2$ FC ≥ 0.2. Genes assigned to ribosome assembly were assigned to G_1 and showed a broad induction. This was already apparent in the analysis shown in Fig 4A. It was confirmed in the analysis with a relaxed FC cut-off in Supplemental Figure S7G, where the estimated average changes became larger and the response spread to more subBINS. As explained above and in the legend to Supplemental Figure S7A and S7G, this is because for all genes with a change below the cut-off filter the response is set to zero before calculating an average response for the subBIN. Incidentally, ribosome biogenesis genes generally showed a positive correlation between their iTPS response and their CRF value (Figure 4B) and a broadly reciprocal response to that after transient overexpression of SnRK1 in protoplasts (Baena-González et al. 2007) (Figure 8C).

Cell wall synthesis and modification; cell expansion

We also inspected the responses of genes involved in cell wall biosynthesis, which is a major consumer of C during cell growth, and cell wall modification, which is required for expansion growth (Supplemental Figure S7H).

In the BIN 'Cell wall', many genes assigned to cellulose, hemicellulose and pectin synthesis were assigned to G_2 and repressed, as were several genes for various classes of cell wall proteins, especially arabinogalactan proteins (AGPs). Overall, there was a trend to repression of genes involved in cell wall synthesis but much of this response is indirect.

Some genes involved in cell wall modification were assigned to G_1 and even more genes to G_2, with both sets being repressed. This repression affected both expansins (*EXPA*s) and xyloglucan endotransglucosylases (*XTH*s) (see also Supplemental text section 'Expansions and Xyloglucan endotransglucosylases', below). Pectin esterases were assigned to G_1, G_2 and G_0, with an overall trend to induction.

This indicates a mixed impact of an induced elevation of Tre6P on cell wall modification with some direct effects but many indirect effects, which tend to be repressive.

Flowering induction

Flowering time is regulated by a large number of pathways that provide information about photoperiod, temperature, plant maturity and metabolic status (Jin and Ahn 2020; Quiroz et al. 2021; Izawa, 2021). Tre6P has been implicated in the regulation of floral initiation (Schleupmann et al., 2003; Wahl et al. 2013; Fichtner et al. 2020), acting to promote the CONSTANS/FLOWERING TIME (CO/FT) photoperiod pathway (for background see Turck et al. 2007; Shim et al. 2017) and the miR156 maturity pathway (Wang 2014, Ponnu et al. 2020). Further, the delay in the floral transition in mutants with low TPS abundance is partly reverted in mutants with modified SnRK1 function (Zacharaki et al. 2022). We therefore inspected how genes involved in the various floral induction pathways respond to transient elevation of Tre6P (Supplemental Fig S9).

Many floral induction genes were assigned to G_1 and repressed by elevated Tre6P. This includes S*UPPRESSOR OF CONSTANS1* (*SOC1*), *SQUAMOSA PROMOTER-BINDING PROTEIN-LIKE* (*SPL*) *SPL3* and *SPL4*, *SHORT VEGETATIVE PHASE* (*SVP*) and *PHYTOCHROME INTERACTING FACTOR 3* (*PIF3)* and *PIF4*, which are inhibitory genes in the temperature-dependent flowering pathway (Yin and Ahn 2021; Brightbill and Sung 2022). *PIFs* are also involved in the maturity pathway (Wang, 2014). In addition to this repression of *PIF3* and *PIF4*, *PIF5* was also repressed, although its assignment to CRF G_2 indicates this is not due to Tre6P-signaling.

Other genes repressed by elevated Tre6P and assigned to CRF G_1 included members of the *GIBBERELLIN-INSENSITIVE DWARF* family (*GID1A*, *GID1C*), and *TEMPRANILLO* family (*TEM1*, *TEM2*)

that are involved in the hormonal regulation of flowering under long days (Griffiths et al. 2007; Takeshi et aI. 2021). Gibberellin signaling interacts negatively with DELLA proteins (Li et al. 2018; Takeshi 2021; Takeshi et al. 2021). The DELLA protein *REPRESSOR OF GA1-3* 1 (RGA1) was assigned to G_1 and weakly repressed. Three further DELLAs (*RGA2/GAI* and *RGA-LIKE 1* (*RGL1*) and *RGL3*) were assigned to G_2, with *RGA2* and *RGL1* being strongly repressed and RGL_3 being weakly induced.

Many genes involved in meristem identity were repressed but assigned to G_2 indicating that the responses may be indirect. Several genes were assigned to G_O indicating that they may be regulated by Tre6P in a manner that is cryptic in wild-type plants; this included the *FT-INTERACTING PROTEIN 1* (*FTIP1*) that is required for FT transport from the leaf to the shoot apex (Liu et a*l*., 2013) and *FD/BZIP14* that interacts with FT at the shoot apex to promote flowering (Abe et al. 2005). Other genes assigned to G_0 included the LOV-domain blue light receptor *ZEITLUPE* (*ZTL*) as well as *SPL5* and *SPL9* which, like *SPL4*, were repressed. *SPL* family genes are downstream of miR156 in the age-dependent floral induction pathway (Wang et al. 2014), which has previously been implicated in the regulation of flowering by Tre6P under short-day conditions (Wahl et al. 2013)

It has previously been reported that constitutively elevated Tre6P leads to increased expression of *FT* (Wahl et al*.* 2013; Fichtner et al*.* 2020) and accelerates flowering in long days. *FT* expression was not significantly increased in the iTPS data set but this may be because the plants were harvested early in the light period, before *FT* transcript rises.

Circadian clock components

Sugar-signaling can modulate the circadian clock (Haydon et al*.* 2013; Frank et al. 2018; Webb et al. 2019; Viani et al. 2022). We Inspected the iTPS response for core components of the circadian clock (Supplemental Fig S10), to learn if these responses were in part due to Tre6P-mediated signaling.

There were widespread changes in transcript abundance of clock components including *PSEUDO RESPONSE REGULATOR 5* (*PRR5)* and all three components of the Evening Complex (*EARLY FLOWERING 3* (*ELF3*), *ELF4* and *LUX ARRHYTHMO*), all of which were assigned to G_1 and repressed by iTPS. *PRR7* has been reported to be induced by low C (Haydon et al. 2013; Moraes et al. 2019), but no significant change was found in the iTPS response (see later in this section for a possible explanation). Relatively few circadian clock genes were assigned to G_2, but some were assigned to G_0 including *GIGANTEA* (*GI*) and the clock output *CONSTANS* (*CO*) that induces *FT* in a light-dependent and clock-gated manner from about 12-14 h after dawn onwards (Mikael and Dorothee 2014; Jae et al. 2017) to induce flowering in long days.

There was a general trend to increased abundance of transcripts (especially at 6h post-induction) for dawn clock components that peak at dawn and subsequently decline, and decreased

abundance of components for day, dusk and especially evening components that are low at dawn and rise during the light period (Supplemental Figure S10). This observation is consistent with a rise in Tre6P early in the light period leading to a delay in the subsequent progression of the clock. The magnitude of these changes (FC on a $\log_2$ scale) was up to 0.7 for dawn genes, and to -0.4 to -1-9 for day, dusk and evening genes with the largest observed decrease being observed for the *ELF4* transcript. This was small relative to the amplitude of the diel changes of transcript abundance in plants growing in the conditions used for the RNAseq experiment (FC $\log_2$ 8-11; Flis et al. 2019; Moraes et al. 2019). These observations nevertheless point to elevation of Tre6P at 4-6 h into the light period having a measurable impact on core clock transcripts, possibly due to a delay in clock progression leading to slower decay of dawn transcripts and slower rise of transcripts that peak later in the diel cycle. Indeed, 'Entrainment of the circadian clock' was highlighted as an over-enriched category in the GO analysis of the iTPS CRF G_1 response (Supplemental Figure S11).

The absence of a significant response of *PRR7* transcript is puzzling, as this gene has been reported to be a target of low sugar signaling (Haydon et al. 2013; Moraes et al. 2019) mediated by SnRK1-dependent phosphorylation of bZIP11 (Frank et al*.* 2019; Viani et al. 2021). It is possible that the absence of a response of *PRR7* is because it reaches its diel peak at 4-6 h after dawn and is relatively insensitive to a transient elevation of Tre6P at this time. Alternatively, the absence of a response of *PRR7* might reflect the complexity of upstream signaling with both Tre6P and sugars providing input, that in wild-type plants would change in parallel but in the iTPS response change in a reciprocal manner. For example, it has been proposed that sucrose acts via ZTL (which is repressed in the iTPS response and assigned to CRF G0) to stabilize GI protein (Dalchau et al. 2011; Haydon et al. 2017). An additional factor may be that light modulates action of SnRK1 on the clock (Shin et al*.* 2017).

Overall, the rapid response of transcripts for core clock completes to elevated Tre6P underlines the sensitivity of clock dynamics to changes in the C status. It in broad agreement with earlier studies showing that sugars regulate the expression of many clock components (Dalchau et al. 2011; Haydon et al. 2017; Shin et al. 2017; Webb et al. 2019). That said, the response to elevated Tre6P differs in details from that seen in previous studies. For example, low C and Tre6P promote SnRK1-dependent action of bZIP63, leading to increased *PRR7* expression and lengthening of clock period (Haydon et al. 2013; Frank et al2018; Viana et al. 2021) and sudden low-light perturbations lead to lower sucrose and Tre6P, increased *PRR7* expression and a small delay in clock progression (Moraes et al. 2019). In contrast, *PRR7* did not respond in our study (see above) and elevated Tre6P delayed rather than advancing clock progression (see above). This may be because we investigated the response to elevated Tre6P, whereas most previous studies addressed C-starvation. Furthermore, metabolic- and light signaling interact to modify clock gene expression (Shin et al. 2017; Shor et al.

2017; 2018) and Tre6P may modify this interaction (see next section and section 'Direct and indirect impacts on light, circadian and ABA signaling', later in the Supplemental text).

Light signaling

Paul et al. (2010) identified 23 genes that are involved in light signaling and were repressed in response to constitutive overexpression of bacterial TPS (oeTPS). Of these genes, 19 were present in the iTPS data set after fusing it with the set of genes for which CRF could be scored (see Supplemental Figure S2). We inspected the responses of these and further genes involved in light signaling (Supplemental Figure S12C).

Transcript abundance for all 19 genes was decreased in the iTPS response, with 17 being assigned to CRF G_1. This included *CCR-LIKE* (*CCL*), *EARLY PHYTOCHROME RESPONSIVE 1* (*EPR1*), *REVEILLE2* (*CIR/RVE2*), *PHOTOTROPIN1* (*PHOT1*), *PHYTOCHROME KINASE SUBSTRATE 1* and *2* (*PKS1, PKS2*), *PIF4,* which has previously been shown to be sugar regulated during diel cycle (Moraes et al. 2019) and some phototrophic response proteins. Further genes involved in light signaling that were strongly repressed in the iTPS data set included the clock component *ELF4* (see also above), *PKS4*, and further phototropic response proteins, and *ELONGATED HYPOCOTYL 5* (*HY5*). The latter is involved in transcriptional regulation of many processes including photomorphogenesis, ABA signaling and anthocyanin biosynthesis inhibition (for more details see section 'Transcription factors' below and the main text Discussion). Several of these genes, including *HY5*, were assigned to CRF group G_1. Analysis of the iTPS response also uncovered induction of several genes involved in CONSTITUTIVE PHOTOMORPHOGENESIS 9 (COP9) signaling that were assigned to CRF G_1. These were also induced in the oeTPS dataset of Zhang et al. (2009). Overall, this comparison reveals remarkable agreement between the response of light signaling genes to transiently elevated Tre6P and a constitutive increase in Tre6P, and provides evidence that this response was largely due to signaling down stream of Tre6P.

Overall, as suggested by Paul et al. (2010), Tre6P interacts with and inhibits light signaling. This provides a mechanism whereby light-induced morphogenesis and growth responses can might be modified and tuned by carbon availability (see also section 'Direct and indirect impacts on light, circadian and ABA signaling' later in the Supplemental text).

Expansins and Xyloglucan endotransglucosylases

Paul et all. (2010) reported repression of many genes involved in cell wall modification in lines with constitutive e overexpression of TPS. These responses and those after induction of TPS are summarized in Supplemental Figure S12D-E. iTPS led to a broad repression of most of the *EXPA* family, however most were assigned to CRF groups G_2 or G_0 (i.e., Tre6P-independent). It led to broad

repression of *XTH* family members, with some being assigned to CRF group G_1 and the others to G_2 and G_0 (i.e., a mix of Tre6P-dependent and Tre6P-independent responses). This could reflect the respective C demand for these different types of cell wall modification. EXPAs allow cell wall expansion without incorporation of new cell wall polymers, whilst XTHs allow this and are also required for insertion of newly synthesized hemicellulose polymers into the cell wall. Overall, our analyses of the iTPS responses indicate that much of the repression of cell wall-modifying genes may be indirect.

Trehalose phosphate synthases and trehalose phosphate phosphatases

The transient elevation of Tre6P was achieved by induced expression of a heterologous bacterial TPS. We asked how the endogenous Tre6P pathway responds to this sudden imposed increase in Tre6P (Figure 7, Supplemental Figure S13). Tre6P is synthesized by TPS1, whilst TPS2-4 are catalytically active but only expressed at a specific stage of seed development (Delorge et al., 2014; Fichtner and Lunn, 2021). TPS1 was assigned to G_2 and repressed, possibly due to the decrease in sugar. TPS5-11 are termed ‘class II’ TPSs and lack catalytic activity (Ramon et al., 2009; Fichtner and Lunn, 2021). Except for *TPS7*, all class II *TPS*s were repressed and all of these except *TPS5* were assigned to G_1. The repression was strongest for *TPS8-11,* whilst *TPS5-7* showed smaller and less consistent changes. Arabidopsis possesses ten diverse TPPs (Vandesteene et al., 2012). These were assigned to G_2 or G_0 with two being induced and six repressed.

These observations point to large-scale rewiring of Tre6P metabolism in response to an imposed increase in Tre6P (see also the Discussion section in the main manuscript).

Expression of TOR subunits

There was an increase in transcript abundance for many genes that encode ribosome biogenesis factors and structural components of the cytosolic and mitochondrial ribosomes (see main Manuscript and above, Figure 4, Supplemental Figure S7G). TORC is known to positively control ribosome assembly in mammals, fungi and plants, acting in a broadly opposed manner to SnRK1 (Sabatini 2017; Ryabova et al. 2018; Wu et al. 2019; Meng et al. 2022; Scarpin et al. 2020; 2022). The molecular relationship between SnRK1 and TORC is not well understood, and probably complex. However, the observation that SnRK1α1 (the catalytic subunit of SNRK1) and RAPTOR1B proteins interact in the cytosol (Nukarinin et al. 2016) and that SnRK1α1 phosphorylates RAPTOR1B protein *in vitro* (Nukarinen et al*.* 2016) indicates that the mutual interactions may include direct action of SnRK1 on TORC. Another possible connection is that ABA-activated SnRK2s can directly phosphorylate RAPTOR or release activated SnRK1 to phosphorylate RAPTOR, thereby repressing TOR-signaling (Wang et al.

2018; Belda-Palazon et al. 2020). We asked if the iTPS response provided any evidence for an interaction between Tre6P-signaling and TOR-signaling.

The TARGET OF RAPAMYCIN (TOR) complex (TORC) is a trimer consisting of the TOR catalytic subunit, REGULATORY-ASSOCIATED PROTEIN OF TOR (RAPTOR) and LETHAL WITH SEC THIRTEEN PROTEIN 8 (LST8), whereby both RAPTOR and LST8 are encoded by two genes. Supplemental Figure S15A summarizes the impact of iTPS on expression of the TORC subunits. There was little response, apart from a weak induction of RAPTOR 1, which the assignment to CRF G_2 indicated was unlikely to be a response to Tre6P-signaling.

Shared targets with post-translational TOR signaling

TORC acts by phosphorylating direct targets like S6Kinase, YET ANOTHER KINASE 1(YAK1) and LA-RELATED PROTEIN 1 (LARP1) that in turn phosphorylate diverse downstream targets (Sabatini, 2017; Ryabova et al. 2018; Wu et al. 2019; Scarpin et al. 2020; 2022). S6Kinase and LARP1 promote growth by stimulating ribosome biogenesis, protein translation and other processes (Scarpin et al. 2022), whereas inactivation of YAK1 promotes growth (Barrada et al. 2019; Forzani et al. 2019). The LARP1 protein is involved in a TOR-LARP1-50'TOP signaling axis that is conserved in plants and animals and regulates expression of 50'TOP mRNAs, including transcripts encoding ribosome assembly factors and ribosomal proteins (Scarpin et al. 2020; 2022). The resulting increase in ribosome abundance is one of the ways in which TOR orchestrates an increase in protein synthesis and growth.

We investigated whether elevated Tre6P increases expression of S6Kinases (S6PKs), LARP1s and YAK1 (Supplemental Figure S15B). Whilst Tre6P did not consistently alter transcript abundance for S6Kinases, *S6K1* was weakly induced 6h after induction and was also strongly induced in the constitutive oeTPS data set of Zhang et al. (2009). Transcript abundance for *LARP1* family members increased, especially at 6h. The responses of *LARPb* and *LARP1c* were assigned to CRF G_1, consistent with signaling downstream of Tre6P enhancing their expression, whereas *LARP1a* was assigned to G_2. Our analysis also revealed a weak induction of *YAK1*, but this response was assigned to CRF G_0 indicating it may be an indirect effect.

We also inspected the response of further genes implicated in the upstream regulation of ribosome biogenesis (Supplemental Figure S15C). There was weak induction of *NUCLEOSOME ASSEMBLY PROTEIN 1;1* (*NAP1;1*) and *RIBOSOMAL PROTEIN S6* (*RS6)* which jointly promote transcription of rRNA (Son et al. 2015). The response was significant for *NAP1;1* at 4h and 6h, and for *RS6* at 4h. *RS6* was also strongly induced in the constitutive oeTPS dataset of Zhang et al. (2009). The iTPS responses were assigned to CRF group G_1 and reciprocal responses were seen in tSnRK1α1 data

set of Baena-Gonzalez et al. (2007) indicating that Tre6P might act to enhance expression of *NAP1;1* and *RSP,* and hence potentially increase RNA transcription, by inhibiting SnRK1 activity.

We next inspected whether elevated Tre6P might promote transcription of downstream TORC targets listed in Scarpin et al. (2020) and Meng et al. (2022). The *PYR*/*PYL* gene family encodes ABA receptors, which are phosphorylated and inhibited by TORC (Wang et al*.* 2018a). Remarkably, all eight members of the gene family were repressed in the iTPS response (Supplemental Figure 15D, significant in all cases except *PYL3*), and four were assigned to CRF group G_1, indicating that their repression is mediated by Tre6P signaling rather than indirect effects. Further, three of the family (*PYL7, PYL8, PYR9*) were repressed in the constitutive oeTPS data set of Zhang et al. (2009). These findings point to a concerted inhibition of ABA signaling by TORC and by Tre6P, acting post-translationally and transcriptionally, respectively. Interestingly, most of these genes were significantly induced by tSnRK1α1 *(PYL2, PYL3, PYL5, PYL7, PYL8, PYR5,* data from Baena-Gonzaléz et al. 2007) consistent with Tre6P acting via inhibition of SnRK1 to repress ABA receptors.

Inspection of other TOR phosphorylation targets shortlisted by Scarpin et al. (2020) and Meng et al. (2022) (Supplemental Figure S15E) pointed to several being transcriptionally regulated by Tre6P-signaling (i.e., significant iTPS response, assigned to CRF group G_1, including weak induction of several elongation initiation factors (*elF4B1*, *elF4B2*, e*lF2B-δ1*), of *E2F TRANSCRIPTION FACTOR-3* that is involved in the cell cycle, of *ETHYLENE INSENSIVE 2* and of the auxin transporter *PIN2*, as well as inconsistent effects at 4h and 6h on transcripts encoding further translation initiation factors, *CONSERVED BINDING of eIFE* (*CBE1*), the developmental regulator *TOPLESS* and *VILLIN* actin-binding proteins. Many of these genes were also assigned to CRF group G_1. There was a significant increase in expression of autophagosome assembly factor *ATG13a*, but this was assigned to CRF group G_2.

Overall, this analysis revealed a potential synergy between the post-translational regulation of ribosome assembly, translation and other processes by TORC, and the transcriptional regulation of these same processes by signaling downstream of Tre6P. There was an especially clear synergy between TORC and Tre6P for ABA receptors (see also section 'Direct and indirect impacts on light, circadian and ABA signaling', later in the Supplemental text).

Comparison with the transcriptional response to inhibition of TORC

We also compared the response of the global transcriptome to iTPS with the response to modified TORC activity. To do this, two data sets were used in which TORC activity was decreased by estradiol-induction of three different amiR-*tor* (Caldana et al. 2012) or by application of the TORC-inhibitor AZD8055 (Dong et al. (2015). These treatments allowed a transient inhibition of TORC using complementary approaches, one via a genetic intervention and the other via an inhibitor. Both studies

analyzed plant material at a not dissimilar stage to that used in our iTPS treatments, but did differ from our study in that the plants were grown on nutrient agar supplemented with 1% sucrose. The study of Caldana et al. (2015) induced ami-*tor* at 14 days and sampled at 3 and 6 days later for transcript analysis. The responses were similar at both time points, but larger at 6 days post-induction when 515 (359 up- and 156 down-regulated) transcripts passed a filter of log change >2 in at least two lines; for further analyses we used the 6 days post-induction dataset and averaged the response of all three lines. The study of Dong et al. (2015) applied 2 µM AZD8055 after 10 days and harvested seedlings 24 h later, in this case a dataset was available for >24K transcripts, of which 2780 showed significant changes. In both studies, longer treatments led to growth inhibition and bleaching indicating repression of photosynthesis. Comparison of the responses revealed good agreement between the global changes in transcript abundance after inhibiting TOR by induction of ami-*tor* or by application of AZD8055 (R^2 = 0.58, only eight transcripts showed reciprocal changes, Supplemental Figure S15F). Good agreement was also obtained when the ami-*tor* dataset was compared with the response of all 24K transcripts in the Dong et al. (2015) data set (R^2 = 0,51, less than 40 transcripts showed reciprocal changes, analysis not shown).

We first compared the CRF (see Supplemental Figure S2) with the response to inhibition of TORC (Supplemental Figure S15G). In principle, if inputs from C-signaling make a substantial contribution to the regulation of TORC activity and/or if TORC plays a major role in C-signaling, a negative relationship would be expected between the CRF and the response to inhibition of TORC. Plots of the AZD-TOR and the ami-*tor* response against CRF showed weak and negligible negative slopes (R^2 = 0.19 and 0.03, respectively). Further, many transcripts that had a CFR close to zero were strongly induced or strongly repressed in response to inhibition of TORC. A similar but damped pattern emerged when the CRF was compared the unfiltered AZD-TOR and tSnRK1α1 responses (negative slopes, R^2 = 0.07 and 0.21, respectively, analyses not shown).

This contrasts with the tSnRK1α1 response, which was strongly and negatively correlated to the CRF (R^2 = 0.71). These analyses indicate that at a global scale, SnRK1 signaling is closely related to the transcriptional response to the C supply. In contrast, TORC-signaling responds to other unrelated inputs; whilst there may be an input form the C supply, at a global level this is largely masked.

Supplemental Figure S15H compares the global transcriptional response to inhibition of TORC and to overexpression of SnRK1a1 (which mimic the response to a low-TORC-mediated restriction of growth and to a SnRK1-dependent starvation response, respectively). Based on the idea that TORC and SnRK1 act antagonistically (see main Manuscript and above), these treatments would be expected to lead to qualitatively similar responses. Analysis of the filtered data sets (red symbols) revealed that many transcripts did show a qualitatively-similar response, but a substantial subset responded in a

reciprocal manner. Analysis of the unfiltered data sets (faint blue symbols) revealed that many transcripts showed large changes in response to tSnRK1α1 but not to inhibition of TORC, and that many transcripts showed a large response to inhibition of TOR but not to overexpression of SnRK1α1, underlining that the two transcriptional responses are rather different. This may be partly due to different biological systems (transient overexpression in mesophyll protoplasts versus inhibition of TORC in seedlings grown on sucrose-supplemented medium) and to side effects of the chemical and genetic interventions. Nonetheless, these analyses indicate that TORC and SnRK1 do not always act in a strictly reciprocal manner.

This encouraged us to analyze the TORC fingerprint in the iTPS response and, in particular, to compare it with the strong SnRK1fingerprint in the iTPS response.

We first compared the response to inhibition of TORC with the overall iTPS response (Supplemental Figure S15I). Here, a negative relationship might be expected if TORC is activated by rising C-supply and plays a major role in C-signaling. When the overall iTPS response was compared with the response to inhibition of TORC, there was no relationship (weak positive slope; R^2 = 0.035 and 0.059, for the AZD-TOR and ami-*tor* response, respectively). As discussed in the main Results text, the overall iTPS response was negatively but weakly related to the tSnRK1α1 response (see Figure 8A, Supplemental Figure S14C; copies are show in Supplemental Figure S15I).

As already mentioned, the response to elevated Tre6P includes both direct and indirect effects (Results text, Figure 2, Supplementary Figures S2, S3, S5). To separate these, the iTPS response was deconvoluted to assign transcripts to the CRF group G_1, which captures direct responses to elevated Tre6P, and the CRF groups G_2 and $G_{0,}$. The responses of these subsets of genes were then compared to the response to TORC inactivation (Supplemental Figure S15J). The response to AZD- and ami-*tor* inhibition of TORC was unrelated to the iTPS G_1 response (slightly negative and positive slopes, R^2 = 0.03 and 0.05, respectively). Although a subset of transcripts exhibited the expected reciprocal behavior, many responded in the same direction to elevated Tre6P and to inhibition of TORC. This is in striking contrast to the very strong and consistent negative correlation between the tSnRK1α1 response and the iTPS G_1 response (Figure 8B and Supplemental Figure 14C; to aid comparison, copies of these displays are provided in Supplemental Figure S15J). This analysis indicates that in contrast to the strong involvement of SnRK1 in the direct response to elevated Tre6P (see also above), any involvement of TORC is more complex (see next but one paragraph for more discussion).

There was no consistent relationship between the response to inhibition of TORC and the iTPS G_2 response, (positive and slight negative and positive slopes, R_2 = 0.33 and 0.002, respectively). This contrasts with the marked positive correlation between the tSnRK1α1 response and the iTPS G_2 response (R^2 = 0,29, Supplemental Figure S14D; copies of these displays are provided in Supplemental

Figure S15J). The differing responses in the AZD-TOR and ami-*tor* responses may reflect differences in the experimental design, including the later sampling time in the latter. There was a weak positive relationship between inhibition of TORC and the iTPS G_0 response (R^2 = 0,13 and 0.16 for the AZD-TOR and ami-*tor* treatments, respectively).

The analysis of the iTPS G_1 gene set indicated that the direct response to elevated Tre6P involves, in some cases, responses of transcript abundance that resemble those that would also be initiated by increased TORC activity and, in other cases, responses that are opposite to those that are initiated by increased TORC activity (see Supplemental Figures S15J). This mirrors the complex relationship between the response TORC-inhibition and SnRK1α1 overexpression seen in Supplemental Figure S15H. This is also illustrated by Supplemental Figure 15K, where the responses to tSnRK1a1 and to TOR-inactivation are compared for the subset of genes in the iTPS groups G_1. If SnRK1 and TORC act antagonistically, we would expect a qualitatively similar response. Whilst some genes show qualitatively similar responses, many others show reciprocal responses.

This mixed response was explored further for genes in the MapMan BINs 'Ribosome biogenesis' and 'Photosynthesis'. For ribosomal biogenesis, many transcripts showed a qualitatively reciprocal response to iTPS and to inhibition of TORC. It should be noted that this does not show that Tre6P is acting via TORC; it is equally likely that Tre6P acts via another pathway that produces the same outcome as stimulation of TORC, e.g., by inhibiting SnRK1. There are also many outliers. For photosynthesis, many genes showed a qualitatively similar response to iTPS and inhibition of TORC, being largely repressed in both treatments, although with outliers. Repression of photosynthesis has been noted previously after inhibition of TORC (Caldana et al. 2012; Dong et al. 2015; Dobronel et al. 2016), in agreement with the bleaching phenotype that is seen after longer term inhibition of TORC activity. Although TORC is usually viewed as acting in a reciprocal manner to SnRK1, in this case elevated Tre6P (probably acting via inhibition of SnRK1) and TORC-inactivation lead to a qualitatively similar response.

Overall, these analyses underline the central role of SnRK1 in the direct transcriptional response to elevated Tre6P, and indicate that the action of Tre6P on TORC plays a small or subsidiary role. They also indicate that, whereas Tre6P- and SnRK1-signalling play a major role in orchestrating the transcriptional response to the C supply, for TORC-signaling the C supply is only one of a large number of inputs; further inputs probably include nutritional status and hormonal signaling (Wu et al. 2019; Liu et al. 2022; Meng et al. 2022). Further, they indicate that in some cases Tre6P- and SnRK1-signaling interact in a complex manner with TORC-signaling (i.e., there is no simple reciprocal relationship), for example, TORC signaling induces photosynthesis and growth, but photosynthesis is repressed by elevated Tre6P. It is possible that Tre6P- and SnRK1-signaling may act to coordinate and

fine-tune different parts of the broad stimulation of photosynthesis and growth processes that is unleashed by TORC. For example, they might act to repress photosynthesis when it is producing more fixed C than is needed for growth. That said, this analysis has been carried out with data sets collected from differing biological material. Rigorous examination of this idea will require parallel perturbation of Tre6P, SnRK1 and TORC in the same biological material.

FCS-LIKE ZINC FINGER (FLZ) family proteins

FLZ family proteins (Jamsheer et al. 2015) are emerging as newly identified negative regulators of SnRK1 and, possibly, as being involved in interactions between SnRK1 and TORC (Nietzsch et al. 2014; 2016; Jamsheer and Lamxi 2015; Jamsheer et al. 2015; 2018a; 2018b; 2022; Bortlik et al. 2022). We therefore inspected their transcriptional response to iTPS.

As background, the expression of FLZ family proteins is known to be differentially regulated by sugars, cellular energy level and abiotic stress (Jamsheer and Laxmi 2015). Whilst many *FLZs* are induced by high sugars, others are unaffected or repressed. This pattern is broadly reciprocal to their response to constitutive overexpression of SnRK1α1, indicating that SnRK1 is involved in their response to sugars (Jamsheer and Laxmi 2015). Many FLZ proteins interact with and negatively regulate SnRK1 activity (Nietzsch et al. 2014, 2016; Jamsheer et al. 2018a;2018b; 2022) by, at least for FLZ3, interfering with phosphorylation of the T-loop of SnRK1α1 (Bortlik et al. 2022). The finding that TORC activity is attenuated in some *flz* mutants (*flz6*, *flz10*, Jamsheer et al. 2018a; *flz8*, Jamsheer et al., 2022) supports a model in which FLZ proteins promote TOR-signaling in high sugar conditions by inhibiting SnRK1 (Jamsheer et al. 2018a; 2018b; 2022). There may also be a reciprocal interaction between TORC and FLZ function. The finding that several FLZ proteins (FLZ3, FLZ4, FLZ5, FLZ6, FLZ7) are encoded by mRNAs with 5'TOP motifs indicates that their translation may be promoted by the TOR-LARP1-50'TOP signaling axis (Scarpin et al. 2022). The implication is that in conditions where TOR is activated, TOR may fine-tune translation of *FLZ* mRNAs to increase FLZ protein levels, restrict SnRK1 activity and promote growth (Scarpin et al., 2022). This emerging role of FLZ proteins as possible mediators between SnRK1- and TORC-signaling led to us to inspect whether their expression was modified by elevated Tre6P (Supplemental Figure S16).

FLZ family members are ordered in Supplemental Figure S16 according to the three sets that Jamsheer and Laxmi (2015) defined based on the response after exogenously adding sugar to seedlings; set 1 shows a large induction by sugar, set 2 a weaker induction and set 3 shows no response or repression. This assignment was largely confirmed by the CRF that we calculated for each gene response (Supplemental Figure S16). Most of the *FLZ* family members in set 1 (*FLZ1, FLZ5, FLZ8, FLZ14*) and some in set 2 (*FLZ3, FLZ10, FLZ15*) were significantly repressed in the iTPS response, as well as

FLZ6 from set 3 (which was classified as sugar non-responsive by Jamsheer and Laxmi (2015) but which was classed as sugar-responsive using the broader set of treatments that we used to calculate CRF values). The above iTPS responses were all assigned to CRF groups G_2 or G_0, indicating that they are triggered by lower sugar or other indirect effects, rather than by elevated Tre6P itself. At least some of these *FLZ* family members genes were previously reported to be repressed by transient overexpression of SnRK1α1 (*FLZ3, FLZ8* by Jamsheer and Laxmi (2015); *FLZ14* by Baena-Gonzalez et al. (2007), see Supplemental Figure S16). The implication is that, at least in these cases, SnRK1-signaling is not modulated by Tre6P or, at least, that Tre6P is not the major input that regulates SnRK1 activity. Of the remaining four FLZ family members, which are repressed by sugar, three (*FLZ9, FLZ13, FLZ17*) were also significantly repressed in the iTPS response, and these were assigned to CRF group G_1. At least two of them (*FLZ9, FLZ17)* were previously reported to be induced by transient overexpression of SnRK1α1 (Jamsheer and Laxmi 2015). The implication is that SnRK1 induces these *FLZ* family members and that this is counteracted by elevated Tre6P.

Overall, this analysis, firstly, adds to the evidence that expression of the *FLZ* family is highly regulated by C status and, secondly, points to Tre6P inhibition of SnRK1 activity contributing to the regulation of a subset of *FLZ* genes that are induced in high sugar, whereas other signaling pathways are involved in the regulation of family members whose expression increases in low C conditions.

Overlap with bZIP11 signaling

S_1 and C class bZIP transcription factors play an important role in low-energy signaling in plants (Dröge-Laser and Weiste (2018). The C supply regulates expression of the S_1 type transcription factor bZIP11 translationally, with high sucrose acting at upstream open reading frames (uORFs) to stall ribosome progression, this block being removed in low-C conditions to promote translation (Wiese et al, 2004; Rahmani et al. 2009). A similar translational regulation may also apply for other S_1 bZIP proteins (bZIP1, bZIP2, bZIP44, bZIP53) (Juntawong et al*.* 2014). It is also known that growth is inhibited by overexpression of some S_1 class bZIPs including bZIP11 (Hanson et al. 2009; Ma et al. 2011, Dröge-Laser and Weiste2018). Further, constitutive overexpression of *bZIP11* in Arabidopsis led to changes in transcript abundance that partly mimic the response to starvation (Ma et al*.* 2011). Changes included increased expression of genes involved in catabolism, genes involved in the synthesis of minor carbohydrates such as myo-inositol and raffinose, and increased expression of *TPPF* and *TPPG* which are thought to catalyze the dephosphorylation of Tre6P to trehalose. We therefore compared the response of transcript abundance to *bZIP11* overexpression with that after transient elevation of Tre6P (Supplemental Figure S19).

In their study of Arabidopsis lines with constitutive overexpression of *bZIP1,* Ma et al. (2011) reported that 232 transcripts showed a significant change and passed a filter of FC ≥ 2 (hereafter termed the oebZIP11 response). We first compared the oebZIP11 response with the CRF (Supplemental Figure S19A). There was a broadly negative relationship (R^2 = 0.24, negative slope), confirming the conclusion of Ma et al. (2011) that bZIP11 overexpression partly mimics the response to low C. When the oebZIP11 response was compared to the overall iTPS response, there was a weak positive relationship (R^2 = 0.14), but many transcripts showed opposing changes or did not change in the iTPS response (Supplemental Figure S19B). Deconvolution of the iTPS response into CRF groups (Supplemental Figure S19C) revealed no relationship between the bZIP11 and iTPS response for transcripts assigned to G_1, a strong positive relationship for transcripts assigned to G_2, and a weak positive relationship for transcripts assigned to G_0. The absence of a relationship for CRF group G_1 is consistent with Tre6P-signaling being largely independent of bZIP11-signaling, and the positive correlation for CRF groups G_2 is consistent with the decline in sucrose levels in iTPS leading to increased bZIP11-signaling. This might be in part due to lower sucrose relieving the translational arrest of bZIP11. It might be noted that any such effects might mask overlap between the response of genes to bZIP-signaling and to elevated Tre6P.

The dataset for constitutive oeTPS (Zhang et al. 2009) contained 94 of the 232 transcripts identified as bZIP11-responsive by Ma et al. (2009). For these transcripts, we inspected how much agreement there was between their iTPS response and constitutive oeTPS response (Supplemental Figure S19D). Whilst there was little agreement across all 94 transcripts, a clear pattern emerged when they were deconvoluted to separate transcripts that respond directly to Tre6P-signalling from transcripts that show indirect effects. This revealed very good qualitative agreement for the subset assigned to iTPS CRF group G1, and a very divergent behavior for the subset assigned to iTPS CRF group G2. This indicates a relatively robust response for transcripts that lie downstream of Tre6P-signaling, but not for transcripts that change due to indirect effects.

Even though the responses to iTPS (or oeTPS) and oebZIP11 were very different, and especially for transcripts assigned to iTPS CRF group G_1, many transcripts were shared between the iTPS and oebZIP11 data sets. When the transcripts in the oebZIP11 data set were scored for their iTPS response, 84, 79 and 28 transcripts were assigned to G_1, G_2, G_0, corresponding to 36, 34 and 12% of the oebZIP11 set, respectively. Only 18% of the transcripts in the oebZIP11 set showed non-significant changes in the iTPS response. The proportion of the total transcripts that showed significant changes in the iTPS response was higher in the subset that showed an oebZIP11 response (82%) than in the total iTPS response (55%, see Supplemental Table S1), and a strikingly high proportion of these (44%) are assigned to CRF groups G_1. Further, many of these shared transcripts showed a qualitatively similar

response in the constitutive oeTPS data set of Zhang et al. (2009). This indicates that although bZIP11- and Tre6P-signaling are independent of each other, they often affect the same genes.

This led us to inspect the response of the iTPS G_1 subset more closely (Supplemental Figure S19E). This display separates genes whose transcripts changed in the same and in an opposite direction in the responses to iTPS and oebZIP11. Given that Tre6P acts a positive signal for sucrose, and bZIP11 is translationally activated by low sucrose, it would be expected that transcripts would show qualitatively opposite responses to iTPS and in oebZIP11. This was the case for 61 (74%) of the transcripts, indicating that these genes are regulated in a mutually-reinforcing manner by Tre6P-signaling and by sucrose-modulated bZIP11-signaling (Supplemental Figure 19F). Reciprocal responses were also found for the 32 transcripts from this set that were reported by Zhang et al. (2009) (Supplemental Figure 19E-F). Genes regulated in a reinforcing manner bZIP11 and Tre6P-SnRK1 included many genes involved in starvation responses and also *TREHALASE1*, *BETA-AMYLASE9 (BAM9)*, a catalytically inactive β-amylase which together with BAM4 acts to accelerate the degradation of assimilatory starch in leaves during the night (David et al*.,* 2021), and *NIGHT LIGHT-INDUCIBLE AND CLOCK-REGULATED2* (*LNK2*), a member of a small gene family that integrates light signaling with the circadian clock and is required for function of the dawn-phased *REVEILLE4* (*RVE4*) and *RVE8* (Xie et al., 2014).

However, 21 (26%) of the transcripts responded in a qualitatively similar manner in the iTPS and oebZIP11 responses indicating that, for these genes, Tre6P-signaling and bZIP11-signaling may counteract each other (Supplemental Figure 19E-F). Ten of these transcripts were reported in the dataset of Zhang et al. (2009) and nine of them responded in a qualitatively similar manner to their response to oebZIP11. This set of genes included genes involved in sucrose transport and gibberellic acid-, auxin- and jasmonate-signaling. It included genes encoding GA-STIMULATED 6 (a cell wall protein downstream of RGL2 that integrates GA, ABA, and glucose signaling, Zhong et al. 2015), the sucrose effluxer SWEET12, a phototropic-responsive NPH3 family member, two SMALL AUXIN UPREGULATED RNAs (SAURs), and the transcription factors JAZ6/TIFY DOMAIN PROTEIN 11B, WRKY17 and NAC20. Dual regulation of *SWEET12* expression by two sugar-signaling pathways might explain why elevated Tre6P sometime represses *SWEET12* (current study, Zhang et al., 2009) and sometimes induces *SWEET12* (Fichtner et al., 2021; Oszvald et al 2018).

We next investigated how Tre6P and SnRK1 interact in the regulation of targets that are shared with oebZIP11 (Supplemental Figure 19G-H). For the 232 genes shortlisted by Ma et al. (2009) as bZIP11 targets, there was no relationship between the overall response of transcripts to iTPS and to transient overexpression of SnRK1α1. However, after deconvolution into CRF groups, we found a negative relationship for transcripts assigned to CRF group G_1, and a positive relationship for

transcripts assigned to CRF groups G_2 (Supplemental Figure 19G). This mirrors the relationship in the entire iTPS data set (Figure 2, Supplemental Figure S5B). The negative relationship in the subset of bZIP11-regulated transcripts that are assigned to iTPS CRF group G_1 is consistent with them being regulated both by bZIP11 and also by Tre6P-inhibition of SnRK1. We also compared the response of transcripts to tSnRK1α1 and oebZIP11 (Supplemental Figure 19H). The vast majority of the transcripts showed qualitatively similar responses to oebZIP11 and tSnRK1α1, both for the total set shared genes (43) as well as for the subsets that were assigned to iTPS CRF group1 (25 genes) and CRF group 2 (14 genes). This again points to their transcript abundance being regulated in parallel by bZIP11-signaling and by SnRK1-signaling.

Finally, Ma et al. (2009) highlighted the response of two *TPPs* (*TPPG* and *TPPF)* to overexpression of bZIP11. We noticed that *TREHALASE1* (*TRE1*) is also repressed in the oebZIP11 data set. The responses of their transcripts are examined in Supplemental Figure 19J. *TRE1* is repressed in the iTPS response and assigned to CRF group G_1, is also repressed by constitutive oeTPS, and is induced by oebZIP11, indicating that Tre6P and bZIP11 act in parallel to repress *TRE1*. A more complicated picture emerged for the two *TPPs*. These are induced by oebZIP11, but are either weakly induced (but assigned to CRF group G_0) or unaffected in iTPS, and both were repressed by constitutive oeTPS. This indicates that constitutive TPS overexpression leads to complex indirect effects on these *TPPs*.

Transcription factors

We also inspected the response of transcription factors (TFs), as a subset of genes that might give independent insights into processes that are impacted in the iTPS response.

Elevated Tre6P led to changes in transcript abundance for about 780 TFs spread across all three CRF groups (290 G_1, 199 G_2, 291 G_0) using a relaxed filter of FDR < 0.05, FC ≥ 0.2, and about 416 TFs (141 G_1, 108 G_2, 167 G_0) using a stringent filter of FDR < 0.05 and FC ≥ 2 filters and requiring the filter to be passed in both the 4-h and 6-h datasets. Supplemental Figure S18A lists examples of strongly responding TFs and Supplemental Figure S18B-E lists TFs that passed a filter of FDR<0.05 and FC > 2at one or more of the two time points, broken down into TF families and, within each family, the CRF response group.

To provide an overview of the response, we performed Gene Ontology analysis (Supplemental Figure S19A). We focused on TFs assigned to CRF group G_1, which are probably responding to signaling downstream of elevated Tre6P. This analysis highlighted several categories related to carbohydrate metabolism and C-signaling (regulation of carbohydrate utilization, cellular response to glucose stimulus, sugar-mediated signaling process) and biosynthetic pathways (regulation of chlorophyll catabolism, synthesis, regulation of wax biosynthesis, anthocyanin-containing compound biosynthetic

process) as well as signaling pathways related to light (shade avoidance, far or red light signaling pathway, response to blue light) and hormones (regulation of auxin biosynthetic process, gibberellic acid-mediated signaling process, ethylene-activated signaling pathway, abscisic acid biosynthetic process), the clock (circadian rhythm) and development (floral meristem determination, phloem or xylem histogenesis). Many of these processes were highlighted in our preceding analyses of the complete iTPS G_1 response.

We also used the STRING on-line tool (https://string-db.org/) to search for functional associations between the TFs that respond to transient elevation of Tre6P (Supplemental Figure 19B) The tool utilizes available datasets, published work, automated text mining and computational predictions from various organisms to score the likelihood of association between proteins, and takes into account physical (direct) and functional (indirect) interactions (Szklarczyk et al. 2021). We again focused on transcription factors assigned to CRF group G_1 (Supplemental Figure S21B). Among these 290 TFs, there were significantly more interactions than expected by chance (p = 1x10^{-16}). Multiple interactions (≥5) are observed for a small number of TFs, indicating that these may be potential central hubs in the iTPS response. The most strongly linked transcription factor was *HY5*, followed by *PIF4*, *HFR1*, *DREB26*, *JAZ6*, *JAZ8*, *HAT2*, a set of *TCP*s (including *TCP14*, *TCP17*, *TCP5*, *TCP20* and *PTF1*/*TCP13*, *APL* (*ALTERED PHLOEM DEVELOPMENT*) and At3g13940) and *bZIP1*. There was also a small network of *NUCLEAR FACTOR Y* (*NF-YC*) family members. HY5, is a master regulator of thousands of genes and coordinates light, environmental and developmental signaling (Gangappa and Botto 2016; Dröge-Laser et al. 2018). *PIF4* is involved in light signaling, *JAZ6*/*MYC3* and *JAZ8*/*MYC4* in jasmonic acid signaling, *TCP*s in morphogenesis and cell cycle regulation, *HAT*s in regulating auxin-mediated morphogenesis in shoot and root tissues, APL1 in phloem development, *NF-YCs* in floral regulation, and *bZIP11* in responses to low energy, whereby *PIF4* is also implicated in aspects of C-signaling. In Supplemental Figure S21B, TFs assigned to selected categories in signaling and metabolism are colored. This again highlights, as expected, a major impact of elevated Tre6P on TFs involved in responses to C supply, but also on chlorophyll biosynthesis, anthocyanin and glucosinolate biosynthesis, as well as light signaling and hormone signaling (including brassinosteroid and auxin). This recapitulates many of the processes that were highlighted in our analysis of the overall iTPS response.

We next looked in more detail at the C and S_1 subfamilies of bZIP TFs. C-bZIPs are translationally regulated by sucrose that represses translation at upstream open reading frames (uORFs) (see above, section ‘Overlap with bZIP11 signaling’) and heterodimerize with group S_1 members to control, in a redundant manner, large sets of genes involved in C- and energy-signaling (Dröge-Laser and Weiste 2018). They also interact with SnRK1-signaling. For example, phosphorylation

of bZIP63 by SnRK1 alters its dimerization behavior and triggers a transcriptional low energy response (Mair et al. 2015), and SnRK1-regulated C/S1 bZIP signaling transcriptionally activates the starvation response in prolonged darkness (Pedrotti et al. 2018).

Most C and S_1 family members showed significant changes in transcript abundance in the iTPS response (Supplemental Figure S20A), including *bZIP9*, *bZIP25* and *bZIP63* in the C subfamily, and *bZIP1*, *bZIP2*, and *bZIP44* in the S_1 subfamily and, of these, *bZIP9*, *bZIP25*, *bZIP63, bZIP1* and *bZIP2* were assigned to CRF groups G_1 indicating that they may be repressed by signaling downstream of Tre6P. Given the known role of SnRK1 in the post-translational regulation of these bZIPs (Dröge-Laser and Weiste, 2018), we compared their transcriptional response to iTPS response with the published response to transient SnRK1α1 overexpression (Baena-González et al., 2007) (Supplemental Figure S20A). Reciprocal responses were found for *bZIP25*, *bZIP63, bZIP1* transcripts, consistent with the transcriptional repression in iTPS being due to inhibition of SnRK1 by Tre6P. However, qualitatively similar responses were found for *bZIP9* and *bZIP2*, whilst *bZIP10* responded to tSnRK1α1 but not to iTPS, *bZIP11* and *bZIP44* were assigned to the CRF group G_2 indicating that they are responding to lower sugar or other indirect effects, and *bZIP53* did not respond to iTPS. Further, whilst *bZIP11* was significantly repressed at 4h, this effect was reverted at 6h, and in the constitutive oeTPS data set of Zhang et al. (2009) it was induced. Thus, the transcriptional regulation of bZIP11 may be rather complex with Tre6P-SnRK1 signaling playing only a minor role. It is also noteworthy that *bZIP63* and *bZIP1* were induced by constitutive oeTPS, in contrast to them being repressed by iTPS, again underlining the complexity of the signaling networks that regulate their expression and the likelihood that the response to constitutive overexpression of TPS may be overlaid by indirect effects. Overall, transcriptional regulation of the C/S1 bZIPs appears to be rather complicated. It is also likely that translational regulation of by sucrose (see references above) may play a more important role in their response to C than changes in transcription per se.

We next looked more widely at the response of transcripts in the bZIP family. This family consists of many further groups that are implicated in light responses, auxin signaling and transport, pathogen responses and basal resistance (Dröge-Laser et al. 2018; Dröge-Laser and Weiste 2018). iTPS led to changes in expression of many members of this family, with some being assigned to CRF group G_1, and others to G_2 or G_0. For each of these sets of genes, we compared the iTPS response with the response to tSnRK1α1 (Supplemental Figure S20B). There was a weak negative correlation for genes assigned to G_1 (R^2 = 0.11 and 0.14 at 4-h and 6-h, respectively) and no relationship for genes assigned to G_2 or G_0. This is consistent with Tre6P-SnRK1 signaling contributing to the transcriptional regulation the bZIPs assigned to CRF group G_1. The weak correlation might be because Tre6P-SnRK1 signaling makes only a minor contribution for many of the genes, or may reflect limitations in comparing

responses in two different experimental systems. The absence of any relation for the genes assigned to G_2 and G_0 is consistent with their expression being regulated by other indirect effects. We expanded this analysis to investigate further TF families, focusing on genes assigned to CRF group G_1 (Supplemental Figure S21). As a general trend, many of the genes showed a qualitatively similar response to iTPS and tSnRK1α1, especially for TFs in the AP2/EREBP, C3H ZINC FINGER, MYB-related and pseudo-ARR families. One notable response was induction of PAP1, a MYB TF that is involved in the sucrose-mediated induction of genes for anthocyanin and flavonoid biosynthesis (Supplemental Figure S18A).

Finally, we next inspected TFs assigned to CRF group G_2, which may be responding to lower sugar in the iTPS response. As already seen for all transcripts assigned to G_2 (Figure 3, Supplemental Figures S7 and S11), very different sets of TFs were assigned to G_2 compared to G_1. Enrichment analyses (Supplemental Figure S22A) highlighted, for example, TFs assigned to water homeostasis, water deprivation, response to sulfur starvation, regulation of glucosinolate biosynthetic process, glucosinolate metabolic process, maturity transition, negative regulation of ABA signaling. Some categories were found in both CRF groups G_2 and G_1, for example, some categories related to light signaling, indicating that not only Tre6P but also other sugar signaling pathways modulate light responses. STRING analysis (Supplemental Figure S22B) highlighted many TFs involved in the response to nitrogen and in the regulation of nitrogen metabolism, sulfur starvation and specialized metabolism including anthocyanin metabolism, flavonoid biosynthesis and glucosinolate biosynthesis.

Supplemental Figure S23 shows the responses of TFs that might contribute to the widespread repression of genes involved in glucosinolate biosynthesis (see Supplemental Figure S7F and, for more detail, Supplemental Figure S23A for genes in the biosynthesis pathway). Many of the genes in the biosynthetic pathways for these specialized metabolites were repressed, but probably via indirect effects as they were assigned to G_2. Interestingly, a set of six upstream MYB family TFs (*MYB29, MYB28, MYB34, MYB51, MYB76, MYB122*) that have been shown to coordinately induce aliphatic or indolic glucosinolate biosynthetic genes (Mitreiter and Gigolashvili 2021) were repressed and assigned to CRF group G_2, as were *OBP1* and *WRKY33* which have also been implicated in upstream regulation of glucosinolate biosynthesis (Mitreiter and Gigolashvili 2021) (Supplemental Figure S23B). This is consistent with the coordinated repression of glucosinolate biosynthesis genes being an indirect effect, possibly due to low sugar repressing the MYB TFs, *OBP1* and *WTK33*. A separate set of MYC TFs that has been implicated in regulating jasmonic acid signaling also modulates glucosinolate synthesis (Dombrecht et al. 2007; Schweizer et al. 2013, Fernandez-Caldo et al. 2011). These MYC TFs act independently of the MYB TFs to regulate glucosinolate biosynthesis (Schweizer et al. 2013). Curiously, two of the *MYBs* were repressed and one was induced and these were assigned to CRF group G_1,

consistent with them being regulated by Tre6P-signalling. This points to the complex response of glucosinolate biosynthesis to C availability, with low sugar repressing sulfur metabolism and glucosinolate metabolism, and Tre6P modulating jasmonic acid-induced defense responses.

Direct and indirect impact of an induced increase in Tre6P on photosynthesis, the C/N balance and growth

It has long been known that sugars repress genes that are involved in photosynthesis (Sheen 1990, Von Schwaeren et al., 1991, Krapp et al., 1993; Lastdrager et al., 2014). In some cases, this may be linked with changes in the C/N balance (Stitt and Krapp, 1999; Li et al., 2021; Wang et al., 2022) or pathogen signaling (Biemelt and Sonnewald, 2006; Doehlemann et al., 2008; de Haro et al 2019). Our data reveal a rapid, direct impact of sugar-signaling on photosynthesis and related processes like plastid ribosomal protein expression that is mediated by Tre6P, likely acting at least partly via SnRK1.

SWEET11 and SWEET12 are involved in sucrose export from source leaves (Braun, 2022; Xue et al., 2022). It is curious that they are repressed by elevated Tre6P that, in wild-type plants, would be associated with higher sucrose in source leaves. A possible explanation is that, in wild-type plants, high sucrose in source leaves might be associated with limited utilization in sink tissues, with repression of *SWEET*s serving to increase retention of C in source leaves and promote nitrate assimilation and amino acid synthesis (Figueroa et al., 2016). Many genes involved in N assimilation and metabolism are induced by sugar (Vincentz et al., 1993; Krapp et al.,1995; Wang et al., 2000; Stitt et al., 2002; Coruzzi, 2003; Vidal et al., 2020). Our analysis of the iTPS response indicates that this is due to sugar signaling that is independent of Tre6P (Supplemental Figure S7C). As sucrose and Tre6P usually change in parallel in wild-type plants (see Introduction) it can be envisaged that Tre6P and sugar signaling act cooperatively in the transcriptional regulation of sucrose export and N assimilation. In addition, Tre6P acts post-translationally to activate NR and PEPC and stimulate amino acid synthesis (Figueroa et al., 2016) allowing rapid fine-tuning of C and N metabolism. Thus, at a whole-plant level, Tre6P-dependent and -independent signaling act cooperatively to balance C and N metabolism and restore growth in sink organs.

An induced elevation of Tre6P also impacted processes involved in cellular growth, including ribosome biogenesis, and cell wall modification. As outlined in the next two paragraphs, elevated Tre6P acted to increase ribosome biogenesis, whereas there were mainly indirect effects on cell wall modification, possibly due to signaling by low sugar. In a whole plant, when sugars rise, a combination of rising Tre6P and rising sugars may coordinately promote protein synthesis and cell expansion. This may be modified by light signaling (see the earlier section 'light signaling' and the next section) and hormone signaling.

Protein synthesis is positively regulated by sugars, which promote both polysome loading and ribosome biogenesis (Juntawong and Bailey-Serres, Pal et al., 2013; Juntawong et al., 2014; Nelson and Millar, 2015; Flis et al., 2016; Ishihara et al., 2017). Translation initiation is stimulated by TOR (Lastdrager et al*.,* 2014), and ribosome biogenesis is stimulated by TOR (Lastdrager et al., 2014; Scarpin et al., 2022) and inhibited by transient SnRK1 overexpression (Baena-González et al., 2007). Our finding that elevated Tre6P rapidly induces genes for cytosolic and mitochondrial ribosomal proteins and ribosomal assembly factors (Figure 4, Supplemental Figure S7G) reveals a link between Tre6P-signalling and ribosome biogenesis. Induction of nucleotide biosynthesis by Tre6P (see above) may also contribute to increased ribosome biogenesis, as rRNA typically represents about 80% of total RNA (Warner, 1999). Increased ribosome biogenesis will presumably promote protein synthesis and cellular growth. More studies are needed to learn if this response is due to increased ribosomal biogenesis in growing sink leaves, or if it also occurs in mature source leaves where increased protein synthesis would presumably be linked with faster protein turnover. Protein turnover represents a substantial energy cost (Ishihara et al., 2017) and it would be interesting to learn if the rate of protein turnover is regulated in response to energy supply. In contrast, genes for plastidic ribosome proteins were mainly repressed. This presumably contributes to the repression of chloroplast function and photosynthesis in sugar-replete conditions.

On the other hand, as already mentioned (see above, section 'Expansins and Xyloglucan endotransglucosylases'), induction of TPS led to broad repression of *EXPA* and *XTH* family members, (Supplemental Figures S7H, S12), which will presumably decrease cell wall modification and cell expansion (Cosgrove, 2005; Kaewthai et al. 2013). However, most *EXPA* and many *XTH* family members were assigned to CRF group G_2, indicating that their repression might be related to the decline in sucrose after induction of TPS, rather than elevated Tre6P. This provides another example where Tre6P and sugar signaling act cooperatively to regulate a higher-level process; in this case, the balance between protein synthesis and cell expansion, with Tre6P-mediated signaling promoting protein synthesis and cellular growth, whilst Tre6P-independent signaling promotes *EXPANSIN* and *XTH* expression and cell expansion.

Direct and indirect impacts on light, circadian and ABA signaling

Tre6P acts transcriptionally on many signaling functions including the circadian clock, light signaling, ABA signaling, auxin signaling and floral induction. These categories were highlighted both in our analyses of the total transcriptome (Figure 6E-F, Supplemental Figures S9-S12, S15) and TFs (Supplemental Figures S20-21).

Light-signaling pathways were highlighted as downstream targets of elevated Tre6P in GO analyses of the response of the total transcriptome and of TFs (Supplemental Figure S11, S12C-D, S24A). There was a remarkably conserved repression of many light-signaling components between the induced and the constitutive (Zhang et al., 2009; Paul et al., 2010) responses (Supplemental Figure S12D). STRING analysis of the responses of TFs highlighted HY5 as a major hub, and PHYTOCHROME INTERACTING FACTOR4 (PIF4) and LONG HYPOCOTYL IN FAR-RED1 (HFR1) as further hubs. HY5 is a master regulator of thousands of genes and coordinates light, environmental and developmental signaling (Gangappa and Botto, 2016; Dröge-Laser et al., 2018). Sugars are known to inhibit *PIF4* expression (Shor et al, 2017; 2018) and this may contribute to modulation of PIF4 function and growth in varying light regimes (Moraes et al., 2019). Overall, as suggested by Paul et al. (2010), Tre6P interacts with and modulates light signaling, providing a mechanism whereby C availability can modify and tune light-induced morphogenesis and growth responses. Sucrose-dependent hypocotyl elongation was prevented in *tps1* mutants and SnRK1α1 overexpressors (Simon et al*.,* 2018). It will be interesting to learn how the interaction between Tre6P and light signaling impacts on the parallel regulation of protein synthesis by Tre6P and cell wall modification by sugars to control expansion growth and plant composition and morphology.

The circadian clock was highlighted as downstream of Tre6P signaling in GO analyses of the response of the total transcriptome response and of TFs (Figure 6F, Supplemental Figures S11, S21). There were widespread changes in the expression of core clock components, with dawn components being induced and day, dusk and evening components repressed (Supplemental Figures S10, Supplemental text). This rapid response to a 3- to 4-fold increase in Tre6P underlines the sensitivity of clock dynamics to changes in the C status and is in broad agreement with earlier studies showing that sugars regulate the expression of many clock components (Dalchau et al., 2011; Haydon et al., 2017; Shin et al., 2017; Webb et al., 2019). However, the response to elevated Tre6P differs in details from that seen in previous studies. For example, low C and Tre6P promote SnRK1-dependent action of bZIP63, leading to increased *PRR7* expression and lengthening of clock period (Haydon et al., 2013; Frank et al., 2018; Viana et al., 2021) and sudden low-light perturbations lead to lower sucrose and Tre6P, increased *PRR7* expression and a small delay in clock progression (Moraes et al., 2019). In contrast, *PRR7* did not respond in our study and the changes in other clock transcripts were consistent with a delay in clock progression by Tre6P. This may be because our experiments investigated the response to elevated Tre6P, whereas most studies of the impact of sugars on the clock have addressed the impact of C-starvation on clock dynamics. Furthermore, metabolic regulation and light signaling interact to modify clock gene expression (Shin et al., 2017; Shor et al., 2017; 2018). Modified light-signaling (see above) may contribute to the response of the circadian clock to elevated Tre6P.

There is a well-documented and close interaction between sugar signaling and ABA signaling (Rolland et al., 2006; Lastdrager et al., 2014) with the arrest of root growth by high sugar levels in the medium being alleviated in mutants defective in ABA sensing or signaling. There are also interactions between Tre6P and ABA signaling (Avonce et al., 2004; Ramon et al. 2007; Debast et al., 2011; Tian et al., 2019; Belda-Palazón et al., 2020, 2022), and reduced-function mutant alleles of *tps1* are hypersensitive to ABA (Gómez et al., 2010). It is known that TOR phosphorylates and inhibits all eight members of the PYR/PYL ABA receptor family (Meng et al., 2022). Strikingly, elevated Tre6P led to decreased transcript abundance for seven of the eight family members, with four responding in a manner consistent with them being repressed by Tre6P-dependent signaling, three of the four also being repressed by constitutive overexpression of TPS (Zhang et al., 2009) and all four being induced by transient overexpression of SnRKα1 indicating that Tre6P is acting via inhibition of SnRK1(Supplemental Figure S15D). This multilevel regulation of ABA receptors by TOR, Tre6P and SnRK1 may contribute to the cross-sensitization of sugar- and ABA signaling

References

Publications that are cited in the main text can be found in the reference list of the main text. The following lists publications that are cited only in the Supplemental text.

Abe M, KobayashiY, Yamamoto S, Daimon Y, Araki T. FD, a bZIP protein mediating signals from the floral pathway integrator FT at the shoot apex: Science 2005:**309**:1052–1056, doi: 10.1126/science.1115983

Abelanda JA, Bergonzi S, Oortwijn M, Sonnewald S, Du M, Visser RG, Sonnewald S, Bachem CWB. Source-sink regulation is mediated by interaction of an FT homolog with a SWEET protein in potato. Current Biol 2019:**29**:1178-1186

Andrés F, Kinoshita A, Kalluri N, Fernández V, Falavigna VS, Cruz TMD, Jang S, Chiba Y, Seo M, Mettler-Altmann T, Huettel B, Coupland G. The sugar transporter SWEET10 acts downstream of *FLOWERING LOCUS T* during floral transition of *Arabidopsis thaliana*. BMC Plant Biol 2020:**20**:1-14

Avonce N, Leyman B, Mascorro-Gallardo JO, Van Dijck P, Thevelein JM, Iturriaga G. The Arabidopsis trehalose-6-P synthase *AtTPS1* gene is a regulator of glucose, abscisic acid, and stress signaling. Plant Physiol 2004:**136**:3649–3655

Baena-González E, Hanson J. Shaping plant development through the SnRK1–TOR metabolic regulators. Curr Opin Plant Biol 2017:**35**:152–155

Barrada A, Djendli M, Desnos T, Mercier R, Robaglia C, Montané MH, Menand B. A TOR–YAK1 signaling axis controls cell cycle, meristem activity and plant growth in Arabidopsis. Development 2019:**146**: dev171298

Bernard SM, Habash DZ. The importance of cytosolic glutamine synthetase in nitrogen assimilation and recycling. New Phytol 2009:**182**(3):608-320

Biemelt S, Sonnewald U- Plant-microbe interactions to probe regulation of plant carbon metabolism. J Plant Physiol. 2006:163(3):307-318 doi: 10.1016/j.jplph.2005.10.011.

Braun DM. Phloem loading and unloading of sucrose: what a long, strange trip from source to sink. Annu Rev Plant Biol 2022:**73**:553-58

Brightbill CM, Sung S. Temperature-mediated regulation of flowering time in *Arabidopsis thaliana*. aBIOTECH 2022:**3**:78–84

Caldana C, Li Y; Leisse A, Zhang Y, Bartholomaeus L.; Fernie AR; Willmitzer L, Giavalisco P. Systemic analysis of inducible target of rapamycin mutants reveal a general metabolic switch controlling growth in Arabidopsis thaliana. Plant J. 2013:**73**:897**–**909

Chardon F, Bedu M, Calenge F, Klemens PAW, Spinner L, Clement G, Chietera G, Léran S, Ferra M, Lacombe B, et al. Leaf fructose content is controlled by the vacuolar transporter SWEET17 in *Arabidopsis*. Current Biol 2013:**23**:697-792

Coruzzi GM. Primary N-assimilation into Amino Acids in Arabidopsis. Arabidopsis Book, 2003:**2**: e0010

Cosgrove DJ. Growth of the plant cell wall. Nat Rev Mol Cell Biol 2005:**6**(11):850-861 doi: 10.1038/nrm1746

David DC, Lee S-K, Bruderer E, Abt M, Fischer-Stettler M, Tschopp M.A, Solhaug E, Sanchez K, Zeeman SC. BETA-AMYLASE9 is a plastidial non-enzymatic regulator of leaf starch degradation. Plant Physiol 2021:**188:**191-207 DOI:10.1093/plphys/kiab468

de Haro LA, Arellano SM, Novák O, Feil R, Dumón AD, Mattio MF, Tarkowská D, Llauger G, Strnad M, Lunn JE, Pearce S, Figueroa CM, Del Vas M. Mal de Río Cuarto virus infection causes hormone imbalance and sugar accumulation in wheat leaves. BMC Plant Biol 2019:**19**(1):112
doi: 10.1186/s12870-019-1709-y

Doehlemann G, Wahl R, Horst RJ, Voll LM, Usadel B, Poree F, Stitt M, Pons-Kühnemann J, Sonnewald U, Kahmann R, Kämper J. Reprogramming a maize plant: transcriptional and metabolic changes induced by the fungal biotroph *Ustilago maydis*. Plant J. 2008:**56**(2):181-195 doi: 10.1111/j.1365-313X.2008.03590.x.

Dombrecht B, Xue GP, Sprague SJ, Kirkegaard JA, Ross JJ, Reid JB, Fitt GP, Sewelam N, Schenk PM, Manners JM, Kazan K.. MYC2 differentially modulates diverse jasmonate-dependent functions in Arabidopsis. Plant Cell 2007:**19**:2225–2245

Dröge-Laser W, Snoek BJ, Snel B, Weiste C. The Arabidopsis bZIP transcription factor family — an update. Curr Opin Plant Biol 2018:**45**:36-49

Fernández-Calvo P, Chini A, Fernández-Barbero G, Chico J-M, Gimenez-Ibanez S, Geerinck J, Eeckhout D, Schweizer F, Godoy M, Franco-Zorrilla JM, et al. The Arabidopsis bHLH transcription factors MYC3 and MYC4 are targets of JAZ repressors and act additively with MYC2 in the activation of jasmonate responses. Plant Cell 2011:**23**:701-715

Flis A, Mengin V, Ivakov AA, Mugford ST, Hubberten HM, Encke B, Krohn N, Höhne M, Feil R, Hoefgen R, et al. Multiple circadian clock outputs regulate diel turnover of carbon and nitrogen reserves. Plant Cell Environ 2019:**42**:549–573

Fontaine J-X, Tercé-Laforgue T, Armengaud P, Clément G, Renou J-P, Pelletier S Catterou M, Azzopardi M, Gibon Y, Lea PJ et al. Characterization of a NADH-Dependent Glutamate Dehydrogenase Mutant of Arabidopsis Demonstrates the Key Role of this Enzyme in Root Carbon and Nitrogen Metabolism. Plant Cell 2012:**24**(10):4044-4065

Fu Y, Lim S, Urano D, Tunc-Ozdemir M, Phan NG, Elston TC, Jones AM. Reciprocal encoding of signal intensity and duration in a glucose-sensing circuit. Cell 2014:**156**(5):1084-1095 doi: 10.1016/j.cell.2014.01.013

Forzani C, Duarte GT, Van Leene J, Clément G, Huguet S, Paysant-Le-Roux C, Mercier R, De Jaeger G, Leprince A-S, Meyer C. Mutations of the AtYAK1 kinase suppress TOR deficiency in Arabidopsis. Cell Reports 2019:**27**:3696-3708

Frank A, Matiolli CC, Viana JC, Hearn TJ, Kusakina J, Belbin FE, Wells Newman D, Yochikawa A, Cano-Ramirez DL, Chembath A et al. Circadian entrainment in Arabidopsis by the sugar-responsive transcription factor bZIP63. Current Biol 2018:**28:**2597-2606

Gangappa SN, Botto JF. The multifaceted roles of HY5 in plant growth and development. Mol Plant 2016:**9**:1353-1365

Goddijn OJM, van Dun K. Trehalose metabolism in plants. Trends Plant Sci. 1999:**4**(8):315–319

Griffiths J, Murase K, Rieu I, Zentella R, Zhang Z-L, Powers SJ, Gong F, Phillips AL, Hedden P, Sun T-P, Thomas SG. Genetic characterization and functional analysis of the GID1 gibberellin receptors in Arabidopsis. Plant Cell 2007:**18**:3399–3414

Guo WJ, Nagy R, Chen G H-Y, Pfruder S, Yu Y-C, Santiella D, D Frommer W, Martinoia E. SWEET17, a facilitative transporter, mediates fructose transport across the tonoplast of Arabidopsis roots and leaves. Plant Physiol 2013:**164**:777-789

Harthill JE, Meek SEM, Morrice N, Peggie MW, Borch J, Wong BHC, Mackintosh C. Phosphorylation and 14-3-3 binding of Arabidopsis trehalose-phosphate synthase 5 in response to 2-deoxyglucose. Plant J. 2006:**47**(2):211–223

Haydon MJ, Mielczarek O, Robertson FC, Hubbard KE, Webb AAR. Photosynthetic entrainment of the *Arabidopsis thaliana* circadian clock. Nature **2103:502**:689–692

Haydon MJ, Mielczarek O, Frank A, Román Á, Webb AA. Sucrose and ethylene signaling interact to modulate the circadian clock. Plant Physiol 2017:**175**:947-958

Hwang G, Kim S, Cho J-Y, Paik I, Kim J-I, Oh E. Trehalose-6-phosphate signaling regulates thermoresponsive hypocotyl growth in *Arabidopsis thaliana*. EMBO Rep. 2019:**20**(10):47828

Ishihara H, Moraes T, Pyl E-T, Schulze WX, Obata T, Scheffel A, Fernie AR, Sulpice R, and Stitt M. Growth rate correlates negatively with protein turnover in Arabidopsis accessions. Plant J 2017:**91**: 416-429

Jamsheer LM, Sharma M, Laxmi A. FCS-like zinc finger 6 and 10 repress SnRK1 signaling in Arabidopsis. Plant J. 2018a:**94**:232-245

Jamsheer KM, Shukla BN, Jindal S, Gopan N, Mannully CT, Laxmi A. The FCS-like zinc finger scaffold of the kinase SnRK1 is formed by the coordinated actions of the FLZ domain and intrinsically disordered regions. J Biol Chem 2018b:**293**:13134–13150

Johansson M, Staiger D. Time to flower: interplay between photoperiod and the circadian clock. J Exp Bot 2014:**66**(3):719-730

Izawa T. (What is going on with the hormonal control of flowering in plants? Plant J. 2021:**105**:431–445

Jamsheer,KM, Laxmi A. Expression of Arabidopsis FCS-Like Zinc finger genes is differentially regulated by sugars, cellular energy level, and abiotic stress. Front Plant Sci 2015:**6:**746 doi.org/10.3389/fpls.2015.00746

Jin S, Ahn JH. Regulation of flowering time by ambient temperature: repressing the repressors and activating the activators. New Phytol 2021:**230**:938–942.

Juntawong P, Bailey-Serres J. Dynamic light regulation of translation status in *Arabidopsis thaliana*. Front Plant Sci 2012:**3**:66 doi: 10.3389/fpls.2012.00066.

Juntawong P, Girke T, Bazin J, Baily-Serres J. Translational dynamics revealed by genome-wide profiling of ribosome footprints in Arabidopsis. Proc Natl Acad Sci USA 2014:**111**:E203–212

Kaewthai N, Gendre D, Eklöf JM, Ibatullin FM, Ezcurra I, Bhalerao RP, Brumer H. Group III-A XTH genes of Arabidopsis encode predominant xyloglucan endohydrolases that are dispensable for normal growth. Plant Physiol 2013:**161**(1):440-54 doi: 10.1104/pp.112.207308.

Klemens PAW, Patzke K, Deitmer J, Spinner L, Le Hir R, Bellini C, Bedu M, hardon F, Krapp A, Neuhaus HE. Overexpression of the vacuolar sugar carrier AtSWEET16 modifies germination, growth, and stress tolerance in Arabidopsis. Plant Physiol 2013:**163:**1338-1352

Krapp A, Hofmann B, Schäfer C, Stitt M. Regulation of the expression of rbcS and other photosynthetic genes by carbohydrates: a mechanism for the "sink-regulation" of photosynthesis? Plan J. 1993:**3**:817-828

Krapp A. Stitt M. An evaluation of direct and indirect mechanism for the "sink"-regulation of photosynthesis in spinach: changes in gas exchange, carbohydrates, metabolites, enzyme activities and steady state transcript levels after cold girdling source leaves Planta. 1995:**195**:313-323

Li L, Liu KH, Sheen J. Dynamic nutrient signaling networks in plants. Annu Rev Cell Dev Biol 2021:**37**: 341-367 doi: 10.1146/annurev-cellbio-010521-015047

Li S, Tian Y, Wu K, Ye Y, Yu J, Zhang, Liu Q, Hu3, Li H, Tong Y, Harberd NP, Fu X. Modulating plant growth–metabolism coordination for sustainable agriculture. Nature 2018:**560**(7720):595-600 doi.org/10.1038/s41586-018-0415-

Liao CY, Pu YT, Nolan TM, Montes C, Guo HQ, Walley JW, Yin YH, Bassham DC. Brassinosteroids modulate autophagy through phosphorylation of RAPTOR1B by the GSK3-like kinase BIN2 in Arabidopsis. Autophagy 2022:**19(4):** 1293-1310 DOI 10.1080/15548627.2022.2124501

Liu L, Liu Chang, Hou X, Wanyan Xi, Shen L, Tao Z, Wang Y, Yu H. FTIP1 is an essential regulator required for florigen transport. PLoS Biol 2012:**10**:e1001313

Martínez-Barajas E, Delatte T, Schluepmann H, de Jong GJ, Somsen GW, Nunes C, Primavesi LF, Coello P, Mitchell RA, Paul MJ. Wheat grain development is characterized by remarkable trehalose 6-phosphate accumulation pregrain filling: tissue distribution and relationship to SNF1-related protein kinase1 activity. Plant Physiol 2011:**156**:373–381

Mitreiter S, Gigolashvili T. Regulation of glucosinolate biosynthesis. J Exp Bot 2021:**72**:70-91

Moraes TA, Mengin V, Annunziata MG, Encke B, Krohn N, Höhne M, Stitt M. Response of the circadian clock and diel starch turnover to one day of low light or low CO_2. Plant Physiol 2019:**179**: 1457–1478

Moreau M, Azzopardi M, Clément G, Dobrenel T, Marchive C, Renne C, Martin-Magniete M-L, Taconnat L, Renou J-P, Robaglia C, et al. Mutations in the Arabidopsis homolog of LST8/GβL, a partner of the target of rapamycin kinase, impair plant growth, flowering, and metabolic adaptation to long days. Plant Cell 2012:**24**:463–81

Nelson CJ, Millar AH. Protein turnover in plant biology. Nature Plants 2015:**1**15017

Nietzsche M, Schießl I, Börnke F, Griffiths CA. The complex becomes more complex: protein-protein interactions of SnRK1 with DUF581 family proteins provide a framework for cell- and stimulus type-specific SnRK1 signaling in plants. Front Plant Sci 2014:**5**:1–13

Nunes C, O'Hara LE, Primavesi LF, Delatte TL, Schluepmann H, Somsen GW, Silva AB, Fevereiro PS, Wingler A, Paul MJ. The trehalose 6-phosphate/SnRK1 signaling pathway primes growth recovery following relief of sink limitation. Plant Physiol 2013a:**162:** 1720–1732

Pal SK. Liput M. Piques M, Ishihara H, Martins MCM, Sulpice R, van Dongen J, Yadav UP, Lunn JE, Usadel B, Schulze WX, Stitt M. Diurnal changes of polysome loading track sucrose content in the rosette of wildtype Arabidopsis and the starchless *pgm* mutant. Plant Physiol. 2013:**162**:1246-1265

Pedrotti L, Weiste C, Nägele T, Wolf E, Lorenzin F, Dietrich K, Mair A, Weckwerth W, Teige M, Baena-González E, Dröge-Laser W. Snf1-RELATED KINASE 1-controlled C/S 1-bZIP signaling activates alternative mitochondrial metabolic pathways to ensure plant survival in extended darkness. Plant Cell 2018:**30**:495-509

Ponnu J, Wahl V, Schmid M. Trehalose-6-phosphate: Connecting plant metabolism and development. Front Plant Sci 2011:**2:**70

Ponnu J, Schlereth A, Zacharaki V, Dzialo MA, Abel C, Feil R, Schmid M, Wahl V. 2020. The trehalose 6-phosphate pathway impacts vegetative phase change in Arabidopsis thaliana. Plant J. 2020104(3):768-780. doi: 10.1111/tpj.14965

Quiroz S, Yusti JCs, Chávez-Hernández EC, Martínez T, de la Paz Sanchez M, Arroyo AG, Álvarez-Buylla E-R, García-Ponce B Beyond the Genetic Pathways, Flowering Regulation Complexity in Arabidopsis thaliana. J. Mol. Sci.2021:**22**,5716

Ramon M, Rolland F, Thevelein JM, Van Dijck P, Leyman B. ABI4 mediates the effects of exogenous trehalose on Arabidopsis growth and starch breakdown. Plant Mol. Biol.2007:63:195–206

Ramon M, Dang TVT, Broeckx T, Hulsmans S, Crepin N, Sheen J, Rolland F. Default activation and nuclear translocation of the plant cellular energy sensor SnRK1 regulate metabolic stress responses and development. Plant Cell 2019:**31:**1614–1632

Sánchez-Villarreal A, Davis AM, Davis SJ. AKIN10 activity as a cellular link between metabolism and circadian-clock entrainment in Arabidopsis thaliana. Plant Signal Behav. 2018:**13**(3):e1411448

Schweizer F, Fernandez-Calvo P, Zander M, Diez-Diaz M Fonseca S, Glauser G, Lewsey MG, Ecker JR, Solano R, Reymond P. Arabidopsis basic helix-loop-helix transcription factors MYC2, MYC3, and MYC4 regulate glucosinolate biosynthesis, insect performance, and feeding behavior. Plant Cell 2013:**25**: 3117-3132

Shim JS, Kubota A, Imaizumi T. Circadian Clock and Photoperiodic Flowering in Arabidopsis: CONSTANS Is a Hub for Signal Integration. Plant Physiol 2017:**173**:5-15

Shin J, Sánchez-Villarreal A, Davis AM, Du SX, Berendzen KW, Koncz C, Ding Z, Li C, Davis SJ. The metabolic sensor AKIN10 modulates the Arabidopsis circadian clock in a light-dependent manner. Plant Cell Environ 2017:**40**:997–1008

Simon NLS, Jelena K, Fernandez-Lopez A, Chembath A, Belbin FE, Dodd AN. The Energy-Signaling Hub SnRK1 Is Important for Sucrose-Induced Hypocotyl Elongation. Plant Physiol. 2018:**176**:1299-1310 DOI10.1104/pp.17.01395

Shor E, Paik I, Kangisser S, Green R, Huq E. PHYTOCHROME INTERACTING FACTORS mediate metabolic control of the circadian system in Arabidopsis. New Phytol 2017:**215**:217–228

Shor E, Potavskaya R, Kurtz A, Paik I, Huq E, Green R. PIF-mediated sucrose regulation of the circadian oscillator is light quality and temperature dependent. Gene 2018:**9**(12)**:**628 doi:10.3390/genes9120628

Solano R, Reymond P. Arabidopsis basic helix-loop-helix transcription factors MYC2, MYC3, and MYC4 regulate glucosinolate biosynthesis, insect performance, and feeding behavior. Plant Cell 2013:**25**: 3117-3132

Stitt M, Krapp A. The molecular physiological basis for the interaction between elevated carbon dioxide and nutrients. Plant Cell Environ. 1999:**22:**583-622

Stitt M, Müller C, Matt P, Gibon Y, Carillo P, Morcuende R, Scheible W-R, Krapp A. Steps towards an integrated view of nitrogen metabolism. J Exp Bot 2002:**370**:959-970

Takeshi I. What is going on with the hormonal control of flowering in plants? Plant J 2021:**105**:431–445

Takehishi I, Okada K, Fukazawa J, Yohsuke Takahashi Y. DELLA-dependent and -independent gibberellin signaling. Plant Signal Behav 2021:**13**: e1445933.

Tian L, Xie Z, Lu C, Hao X, Wu S, Huan Y, Li D, Chen L. The trehalose-6-phosphate synthase TPS5 negatively regulates ABA signaling in *Arabidopsis thaliana*. Plant Cell Rep 2019:**38**:869-882

Turck F, Fornara F, Coupland G. Regulation and identity of florigen: FLOWERING LOCUS T moves center stage. Annu Rev Plant Biol 2008:**59**:573-594.

Viana AJ., Matiolli CC, Newman DW, Vieira JGP, Duarte GT. Martins MCM, Gilbault E, Hotta CT, Caldana C, Vincentz M. The sugar-responsive circadian clock regulator bZIP63 modulates plant growth. New Phytol 2021:**231**:1875–1889

Vidal EA, Alvarez JM, Araus V, Riveras E, Brooks MD, Krouk G, Ruffel S, Lejay L, Crawford NM, Coruzzi GM, Guriérrez RA. Nitrate in 2020: Thirty years from transport to signaling networks. Plant Cell 2020:**32**:2094-2119 doi: 10.1105/tpc.19.00748.

Vincentz M, Moureaux T, Leydecker M-T, Vaucheret H, Caboche M. Regulation of nitrate and nitrite reductase expression in Nicotiana plumbaginifolia leaves by nitrogen and carbon metabolites. Plant J. 1993:**3**(2):315-324

von Schaewen A, Stitt M, Schmidt R, Sonnewald U, Willmitzer L. Expression of a yeast-derived invertase in the cell wall of tobacco and Arabidopsis plants leads to accumulation of carbohydrate,

inhibition of photosynthesis and strongly influences growth and phenotype of transgenic tobacco plants. EMBO J 1991:**9**:3033-3044

Wang JW. Regulation of flowering time by the miR156-mediated age pathway. J Exp Bot 2014:**65**: 4723-4730

Wang R, Guegler K, LaBrie ST, Crawford NM. Genomic analysis of a nutrient response in Arabidopsis reveals diverse expression patterns and novel metabolic and potential regulatory genes induced by nitrate. Plant Cell 2000:**12**(8):1491-1509 doi: 10.1105/tpc.12.8.1491.

Wang Z, Wang XM, Xie B, Hong ZL, Yang QC. Arabidopsis NUCLEOSTEMIN-LIKE 1 (NSN1) regulates cell cycling potentially by cooperating with nucleosome assembly protein AtNAP1;1. BMC Plant Biol 2018:**18**:99 doi 10.1186/s12870-018-1289-2

Wang H, Han C, Wang JG, Chu X, Shi W, Yao L, Chen J, Hao W, Deng Z, Fan M, Bai MY. Regulatory functions of cellular energy sensor SnRK1 for nitrate signalling through NLP7 repression. Nat Plants 2022:**8**:1094-1107 doi: 10.1038/s41477-022-01236-5

Wang Y, Wang L, Micallef BJ, Tetlow IJ, Mullen RT, Feil R, Lunn JE, Emes MJ. AKINβ1, a subunit of SnRK1, regulates organic acid metabolism and acts as a global modulator of genes involved in carbon, lipid, and nitrogen metabolism. J Exp Bot 2020:**71**:1010–1028

Warner JR. The economics of ribosome biosynthesis in yeast. Trends Biochem Sci: 1999: **24**:437–440

Wiese A, Elzinger N, Wobbes B, Smeekens S. A conserved upstream open reading frame mediates sucrose-induced repression of translation. Plant Cell 2004:**16**:1717–1729

Williams SP, Rangarajan P, Donahue JL, Hess JE, Gillaspy GE. Regulation of Sucrose non-Fermenting Related Kinase 1 genes in *Arabidopsis thaliana*. Front Plant Sci 2014:**5**:324

Xie Q, Wang P, Liu X, Yuan L, Wang L, Zhang C, Li W, Xing H, Zhi L, Yue Z, et al. LNK1 and LNK2 are transcriptional coactivators in the Arabidopsis circadian oscillator. Plant Cell 2014:**26**:2843-2857

Xue X, Wang J, Shukla D, Cheung LS, Chen L-Q. When SWEETs turn tweens: updates and perspectives. Annu Rev. Plant Biology 2022:**73**:379-403

Yin S, Ahn JH. Regulation of flowering time by ambient temperature: repressing the repressors and activating the activators. New Phytol. 2021:30:938-942. DOI10.1111/nph.17217

Zacharaki V, Ponnu J, Crepin N, Langenecker T, Hagmann J, Skorzinski N, Musialak-Lange M, Wahl V, Rolland F, Schmid M. Impaired KIN10 function restores developmental defects in the Arabidopsis *trehalose 6-phosphate synthase1* (*tps1*) mutant. New Phytol 2022:**235**:220-233

Zhang Z, Zhu J-Y, Roh J, Marchive C, Kim S-K, Meyer C, Sun Y, Wang W Wang Z-Y. TOR signaling promotes accumulation of BZR1 to balance growth with carbon availability in Arabidopsis. Current Biol 2016:**26**:1854–1860

Zhang ZZ, Sun Y, Jiang J, Wang WF, Wang, ZY. Sugar inhibits brassinosteroid signaling by enhancing BIN2 phosphorylation of BZR1. PLOS Genet 2021:**17:**e1009540

Zhong C, Y Xu H, Ye S, Wang S, Li L, Zhang S, Wang X. Gibberellic Acid-Stimulated Arabidopsis6 serves as an integrator of gibberellin, abscisic acid, and glucose signaling during seed germination in Arabidopsis. Plant Physiol 2015:**169**:2288-2303
